# Time to treatment disruption in children with HIV-1 randomized to initial antiretroviral therapy with protease inhibitors versus non-nucleoside reverse transcriptase inhibitors

**DOI:** 10.1371/journal.pone.0242405

**Published:** 2020-11-23

**Authors:** Dwight E. Yin, Christina Ludema, Stephen R. Cole, Carol E. Golin, William C. Miller, Meredith G. Warshaw, Ross E. McKinney, on behalf of the PENPACT-1 (PENTA 9 / PACTG 390) Study Team

**Affiliations:** 1 Division of Infectious Diseases and Division of Clinical Pharmacology, Toxicology and Therapeutic Innovation, Department of Pediatrics, Children’s Mercy-Kansas City and University of Missouri-Kansas City, Kansas City, Missouri, United States of America; 2 Division of Infectious Diseases, Department of Pediatrics, Duke University Medical Center, Durham, North Carolina, United States of America; 3 Department of Epidemiology, Gillings School of Global Public Health, University of North Carolina at Chapel Hill, Chapel Hill, North Carolina, United States of America; 4 Department of Epidemiology and Biostatistics, School of Public Health, Indiana University, Bloomington, Indiana, United States of America; 5 Department of Health Behavior, Gillings School of Global Public Health, University of North Carolina at Chapel Hill, Chapel Hill, North Carolina, United States of America; 6 Department of Medicine, School of Medicine, University of North Carolina at Chapel Hill, Chapel Hill, North Carolina, United States of America; 7 Department of Epidemiology, College of Public Health, The Ohio State University, Columbus, Ohio, United States of America; 8 Center for Biostatistics in AIDS Research, Harvard T.H. Chan School of Public Health, Boston, Massachusetts, United States of America; 9 Association of American Medical Colleges, District of Columbia, Washington, United States of America; IAVI, UNITED STATES

## Abstract

**Background:**

Choice of initial antiretroviral therapy regimen may help children with HIV maintain optimal, continuous therapy. We assessed treatment-naïve children for differences in time to treatment disruption across randomly-assigned protease inhibitor versus non-nucleoside reverse transcriptase inhibitor-based initial antiretroviral therapy.

**Methods:**

We performed a secondary analysis of a multicenter phase 2/3, randomized, open-label trial in Europe, North and South America from 2002 to 2009. Children aged 31 days to <18 years, who were living with HIV-1 and treatment-naive, were randomized to antiretroviral therapy with two nucleoside reverse transcriptase inhibitors plus a protease inhibitor or non-nucleoside reverse transcriptase inhibitor. Time to first documented treatment disruption to any component of antiretroviral therapy, derived from treatment records and adherence questionnaires, was analyzed using Kaplan-Meier estimators and Cox proportional hazards models.

**Results:**

The modified intention-to-treat analysis included 263 participants. Seventy-two percent (*n =* 190) of participants experienced at least one treatment disruption during study. At 4 years, treatment disruption probabilities were 70% (protease inhibitor) vs. 63% (non-nucleoside reverse transcriptase inhibitor). The unadjusted hazard ratio (HR) for treatment disruptions comparing protease inhibitor vs. non-nucleoside reverse transcriptase inhibitor-based regimens was 1.19, 95% confidence interval [CI] 0.88–1.61 (adjusted HR 1.24, 95% CI 0.91–1.68). By study end, treatment disruption probabilities converged (protease inhibitor 81%, non-nucleoside reverse transcriptase inhibitor 84%) with unadjusted HR 1.11, 95% CI 0.84–1.48 (adjusted HR 1.13, 95% CI 0.84–1.50). Reported reasons for treatment disruptions suggested that participants on protease inhibitors experienced greater tolerability problems.

**Conclusions:**

Children had similar time to treatment disruption for initial protease inhibitor and non-nucleoside reverse transcriptase inhibitor-based antiretroviral therapy, despite greater reported tolerability problems with protease inhibitor regimens. Initial pediatric antiretroviral therapy with either a protease inhibitor or non-nucleoside reverse transcriptase inhibitor may be acceptable for maintaining optimal, continuous therapy.

## Introduction

Globally, 1.8 million children are living with HIV, and 110,000 die annually due to AIDS-related illnesses [1]. For HIV-infected children, greatest survival outcomes can be achieved only with optimal, uninterrupted treatment on effective antiretroviral therapy (ART). Treatment disruptions, defined as any interruption or alteration of initial ART, may result from patient-level factors (*e*.*g*., poor adherence, drug intolerance), provider-level factors (*e*.*g*., prescription stops, changes, or errors), or systems-level factors (*e*.*g*., stock outs, interruptions in drug delivery). Unfortunately, treatment disruptions may result in treatment failure, acquisition of resistance mutations, and loss of future treatment options—which are particularly consequential in children. Compared with adults, children have greater pharmacokinetic variability and fewer available licensed drugs [2, 3]. Due to longer lifetime antiretroviral exposure, children have more potential for long-term toxicity [4, 5]. Children have greater social vulnerability related to their dependence on others for medical care and medication administration [6, 7]. If inadequately treated, children progress much faster to AIDS and death [8–10]. As children’s initial ART regimens are often their best opportunity for effective, tolerable treatment, optimizing the time on a successful initial regimen may result in greater long-term effectiveness of ART and more lifetime treatment options [11]. Analyzing longitudinal relationships between pediatric ART regimens and time to treatment disruption allows identification of initial ART regimens that pose greater challenges to maintaining optimal, continuous ART.

When deciding which regimen to prescribe to optimize clinical outcomes, clinicians must consider both drug pharmacology and potential adherence to ART regimens [12]. Boosted protease-inhibitor (PI)-based regimens appear more forgiving of treatment disruptions than do non-nucleoside reverse transcriptase inhibitor (NNRTI)-based regimens [13–17]. However, certain PI characteristics decrease adherence and tolerability, particularly in children: poor taste; gastrointestinal toxicity; and regimen complexity, such as pill burden, storage requirements, and dosing frequency [7, 17–22]. Prior pediatric studies that have assessed the ability of children to maintain continuous therapy did not do so in settings in which use of PI- vs. NNRTI-based ART regimens was randomly allocated, nor have prior studies measured treatment disruptions longitudinally. As a result, these previously conducted studies have potential for residual confounding from unmeasured covariates. Furthermore, most studies have isolated analyses of prescription patterns, adherence, and tolerability, rather than evaluating the total effect of the regimen on maintaining optimal, continuous therapy. In the PENPACT-1 study, 266 HIV-1-infected, treatment-naïve children from Europe, North America, and South America were randomized to ART with either a PI or NNRTI and followed longitudinally for at least 4 years [23]. We aimed to assess PENPACT-1 participants for differences in time to treatment disruption across randomized PI vs. NNRTI treatment arms at 4 years and end of study.

## Methods

### Study design and participants

PENPACT-1 (Paediatric European Network for Treatment of AIDS [PENTA] 9 / Pediatric AIDS Clinical Trials Group [PACTG] 390) was an international multicenter phase 2/3, randomized, open-label trial enrolling children living with HIV-1 from 68 clinical centers in 13 countries in Europe and North and South America between September 25, 2002, and September 7, 2005 (S1 Protocol) [23]. Eligible children aged 31 days to less than 18 years were HIV-1-infected and had not received ART or received only antiretrovirals for <56 days to reduce mother-to-child transmission (excluding single-dose nevirapine). All parents or guardians and children, as appropriate, gave written consent for the parent trial; this protocol was conducted in accordance with the Declaration of Helsinki and approved by the relevant ethics committee or institutional review board (IRB) for each participating center. The secondary analysis on time to treatment disruption was performed under a data request and was reviewed only at IRBs where the analysis was performed. The secondary analysis was deemed exempt by the Duke University IRB and approved by the University of North Carolina-Chapel Hill and Children’s Mercy Kansas City IRBs. This study is registered with the International Standard Randomised Controlled Trial Number Registry (ISRCTN73318385) at https://doi.org/10.1186/ISRCTN73318385 and ClinicalTrials.gov (NCT00039741) at https://clinicaltrials.gov/ct2/show/NCT00039741.

Children were randomized 1:1 to start ART with two nucleoside reverse transcriptase inhibitors (NRTIs) plus either a PI or NNRTI. Randomization was stratified by age (<3 years or ≥3 years); receipt of perinatal ART prophylaxis; and research network (PENTA or PACTG), which varied by region; with variable block sizes. The study was open label, and the treating clinician chose the two NRTI drugs combined with a drug from the randomly assigned PI or NNRTI class. Children underwent clinical and HIV-1 RNA viral load assessments at randomization (week 0), weeks 2, 4, 8, 12, 16, 24, and then every 12 weeks until the last child assigned to treatment reached 4 years of follow-up (August 31, 2009). Treatment starts, changes, and stoppages were recorded at these clinical visits and *ad hoc* throughout the study. Trained study personnel administered validated adherence questionnaires every 24 weeks after randomization, or if missed, at the following attended visit [24]. Adherence questionnaires were harmonized across networks to collect key data. Specifically, adherence questionnaires recorded the number of missed doses to all antiretrovirals over the 3 days prior to these 24-weekly visits and barriers to adherence experienced within 2 weeks prior to these visits. Four years of follow-up was defined as the week 192 visit plus a 6-week lag to capture late visits.

### Outcomes

We defined time to treatment disruption as the number of weeks between randomization and the first documented treatment disruption event. We defined treatment disruption as stopping, switching, or reporting missed doses of any component of the initial ART regimen for any reason except recall of nelfinavir (June 2007) or planned treatment interruptions. Stopping was defined as any duration of treatment discontinuation, regardless of whether treatment was restarted or changed in the future, whereas switches were defined as immediate changes of therapy. Information on ART stoppages or switches was derived from participants’ treatment records, and missed doses were defined as any questionnaire-reported missed doses within 3 days prior to the study visit.

Additional analyses included adjustment for stratified randomization factors (age, receipt of perinatal ART prophylaxis, research network), assessed differences in outcome for the primary follow-up time point (4 years) vs. the entire study, and explored reasons for treatment disruptions. Reasons for treatment disruptions were analyzed using (1) the treatment record’s documented rationale for ART stop or change and (2) any questionnaire-reported barriers to adherence within 2 weeks prior to the visit when missed dose(s) were reported. Only one reason for treatment disruption was allowed on the treatment record; thus a single response, such as “caregiver request,” may not exclude additional reasons. Multiple reasons were allowed on adherence questionnaires.

We assessed the sensitivity of our results to our definition of treatment disruption. Our alternative outcome definitions included restricting treatment record-based treatment disruptions (or any questionnaire event) to drug changes or stops lasting more than 3 days or 14 days and restricting treatment record-based treatment disruptions (or any questionnaire event) to only events including the PI or NNRTI drug component.

### Statistical analysis

PI vs. NNRTI treatment groups were assessed according to a modified intention-to-treat (mITT) analysis consistent with the original study [23]. The sole modification was removal of three participants: two who withdrew consent prior to ART initiation, and one with a major eligibility violation. Follow-up began at date of randomization. Participants were right-censored for initial treatment contrary to randomization, planned treatment interruption, death, withdrawal of consent, loss to follow-up, or study end.

For the primary outcome, we estimated the risk of treatment disruptions using the complement of the Kaplan-Meier estimator. We estimated the hazard ratio for treatment disruptions using Cox proportional hazards models. Proportional hazards assumptions were assessed graphically, using time-interaction terms, and with martingale residuals. In adjusted analyses, we stratified by baseline randomized stratification variables: age, exposure to perinatal ART, and research network. Analyses were conducted in SAS^®^ version 9.4 (Cary, NC).

## Results

PENPACT-1 enrolled 266 HIV-1 infected children from 68 centers in 13 countries in Europe, North America, and South America. The mITT analysis was restricted to 263 participants who initiated ART. Participants were a median age of 6.5 years at enrollment (IQR [interquartile range], 1.8–12.9), 52% male, 49% black, and 79% exposed to HIV via vertical transmission (Table 1). Fifty-one percent had moderate to severe clinical symptoms (CDC stage B or C). Median growth parameters were below average (weight-for-age Z score -0.6; height-for-age Z score -0.9). Median CD4 Z-score was -3.5, consistent with predominance of moderate to severe immunosuppression, and median viral load was 5.0 log_10_ copies/mL. Whereas 15% of children had ART exposure for prevention of mother-to-child transmission, 4% had at least one major resistance mutation at baseline. Although treatment groups had differences in racial distribution, baseline characteristics relating to mode of HIV-1 acquisition, clinical and immunological status, and ART resistance were generally balanced across ART regimens, consistent with the randomized design.

**Table 1:** Baseline characteristics of study participants according to initial ART regimen.

		Randomized Group	
Variable		PI	NNRTI	Total
*N*		131	132	263
Age				
<3 years	*n* (%)	34 (26%)	36 (27%)	70 (27%)
3–17 years	*n* (%)	97 (74%)	96 (73%)	193 (73%)
Age in years	Median (IQR)	7.1 (2.8, 13.7)	6.4 (2.7, 11.0)	6.5 (2.8, 12.9)
Sex				
Male	*n* (%)	69 (53%)	67 (51%)	136 (52%)
Race				
Black, Non-Hispanic	*n* (%)	60 (46%)	69 (52%)	129 (49%)
White, Non-Hispanic	*n* (%)	40 (31%)	29 (22%)	69 (26%)
Hispanic/Other	*n* (%)	31 (24%)	34 (26%)	65 (25%)
Research Network^a^				
PENTA	*n* (%)	95 (73%)	93 (70%)	188 (71%)
PACTG/IMPAACT	*n* (%)	36 (27%)	39 (30%)	75 (29%)
Route of Infection				
Vertical	*n* (%)	103 (79%)	106 (80%)	209 (79%)
Other/Unknown	*n* (%)	28 (21%)	26 (20%)	54 (21%)
CDC Clinical Stage				
N	*n* (%)	27 (21%)	29 (22%)	56 (21%)
A	*n* (%)	35 (27%)	37 (28%)	72 (27%)
B	*n* (%)	41 (31%)	43 (33%)	84 (32%)
C	*n* (%)	28 (21%)	23 (17%)	51 (19%)
Weight-for-Age Z-score	Median (IQR)	-0.5 (-1.6, 0.1)	-0.7 (-1.6, 0.2)	-0.6 (-1.6, 0.1)
Height-for-Age Z-score	Median (IQR)	-0.9 (-1.5, -0.2)	-0.9 (-1.8, 0)	-0.9 (-1.7, -0.2)
CD4 Z score	Median (IQR)	-3.6 (-7.2, -1.7)	-3.4 (-6.5, -1.4)	-3.5 (-6.8, -1.6)
Viral Load log_10_ copies/mL	Median (IQR)	5.1 (4.5, 5.7)	5.0 (4.5, 5.6)	5.0 (4.5, 5.7)
Perinatal ART Exposure	*n* (%)	19 (15%)	20 (15%)	39 (15%)
≥1 Major Resistance Mutation^b^	*n/N* (%)	5/116 (4%)	5/123 (4%)	10/239 (4%)
HIV-1 subtype				
B	*n* (%)	52 (42%)	49 (39%)	101 (41%)
C	*n* (%)	13 (11%)	12 (10%)	25 (10%)
F	*n* (%)	25 (20%)	23 (18%)	48 (19%)
A/CRF_AG/D/G	*n* (%)	21 (17%)	31 (25%)	52 (21%)
Unclassified	*n* (%)	12 (10%)	11 (9%)	23 (9%)
Switching Threshold				
1,000 copies/mL	*n* (%)	66 (50%)	68 (52%)	134 (51%)
30,000 copies/mL	*n* (%)	65 (50%)	64 (48%)	129 (49%)
Duration of Follow-Up in weeks	Median (IQR)	263 (217, 313)	260 (219, 316)	261 (217, 313)

ART, antiretroviral therapy; IQR, interquartile range; *N*, total sample size; *n*, subsample size; NNRTI, non-nucleoside reverse transcriptase inhibitor; PACTG, Pediatric AIDS Clinical Trials Group; PENTA, Paediatric European Network for Treatment of AIDS; PI, protease inhibitor.

^a^ PENTA sites were predominantly in Europe, South America, and the Bahamas. PACTG sites were based primarily in the United States.

^b^ Not all patients had successful baseline genotypic resistance assays.

**Fig 1:**
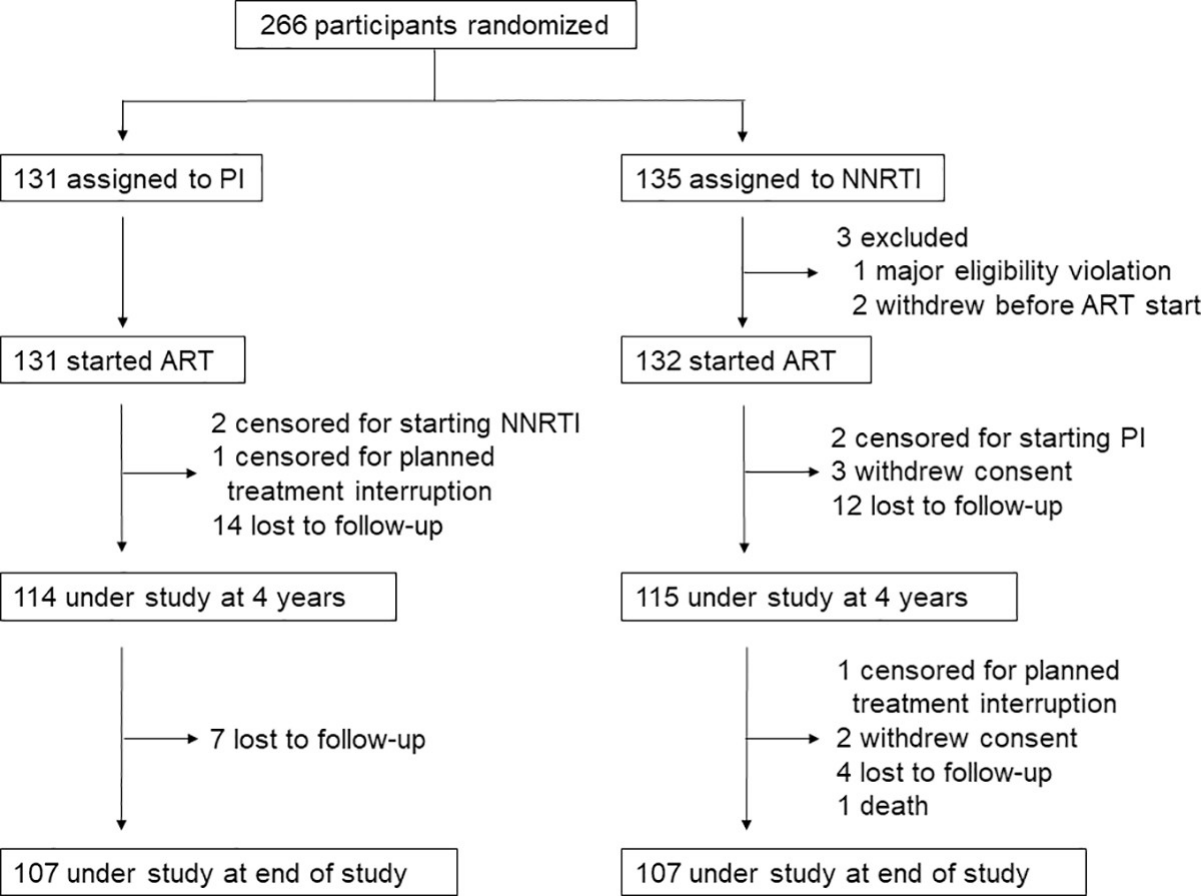
Study profile. ART, antiretroviral therapy; NNRTI, non-nucleoside reverse transcriptase inhibitor; PI, protease inhibitor.

Median follow-up time was 261 weeks (IQR, 217–313). Two participants in each arm were started on a PI or NNRTI contrary to randomization; two underwent planned treatment interruption; five withdrew from study after ART initiation; 37 were lost to follow-up; and one patient died, due to HIV-related complications (Fig 1). Two hundred forty-nine participants ever completed an adherence questionnaire, totaling 2,112 questionnaires over the duration of the study for a mean of 8.5 questionnaires per participant.

**Fig 2:**
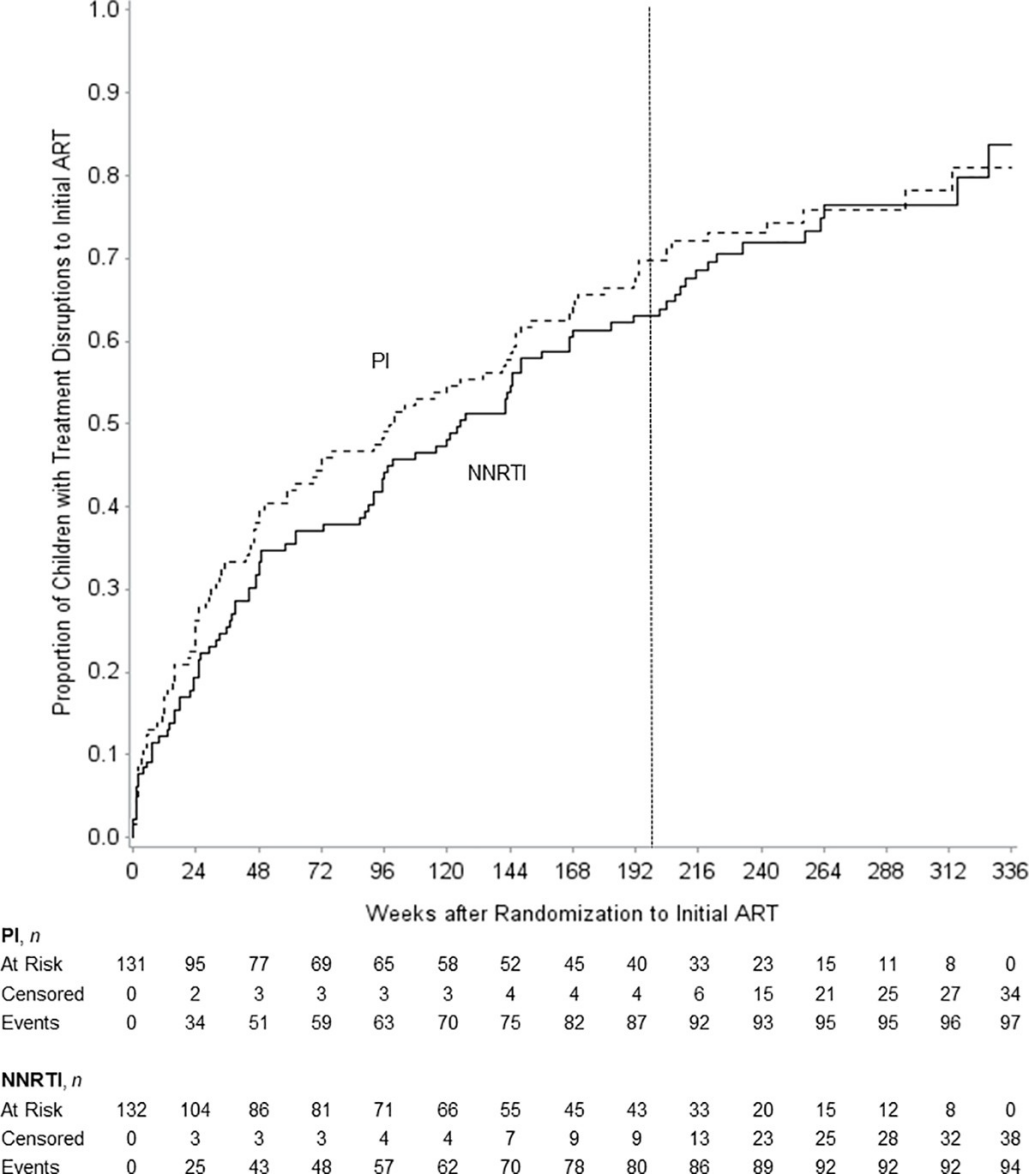
Proportion of children experiencing treatment disruption from initial ART regimen by study week. The vertical line delineates 4 years on study. ART, antiretroviral therapy; *n*, subsample size; NNRTI, non-nucleoside reverse transcriptase inhibitor; PI, protease inhibitor.

Overall, 191 of 263 participants had at least one treatment disruption event during the study, with 66% (95% confidence interval [CI] 61–72%) treatment disruption probability at 4 years (primary follow-up period) and 83% (95% CI 76–91%) treatment disruption probability at study end (6.5 years). At 4 years, probabilities of treatment disruption were 70% (95% CI 62–78%) vs. 63% (95% CI 55–72%) in the PI and NNRTI arms, respectively (Fig 2). Hazards for treatment disruption, however, were similar for PI vs. NNRTI-based regimens (unadjusted hazard ratio [HR] 1.19, 95% CI 0.88–1.61), even after adjustment for stratification factors of age, receipt of perinatal ART, and research network/region (adjusted HR 1.24, 95% CI 0.91–1.68).

After 4 years, treatment disruption probabilities converged, such that treatment disruption probabilities at study end were 81% (95% CI 72–90%) for PI vs. 84% (95% CI 73–94%) for NNRTI arms, but changes over time in the hazard ratio of treatment disruption by treatment arms were non-significant (unadjusted *P* for interaction = 0.33, adjusted *P* = 0.21). Hazards for treatment disruption over the entire study period were similar for PI vs. NNRTI-based regimens, unadjusted (HR 1.11, 95% CI 0.84–1.48) and adjusted (HR 1.13, 95% CI 0.84–1.50).

Of 191 treatment disruption events, 126 events were based on ART regimen stoppages or changes in the treatment record, and 67 events were reported missing doses on adherence questionnaires, with two participants experiencing both event types simultaneously. Of the treatment stops or changes, 25% of events were substitutions of at least one first-line ART drug (PI 32%, NNRTI 16%), 53% were stoppage or suspension of the entire first-line ART regimen (PI 48%, NNRTI 59%), and 22% were switches to a second-line ART regimen (PI 20%, NNRTI 25%). Most frequent reasons documented for ART stops or changes were adverse events (34%), viral failure (22%), caregiver request (18%), non-adherence (7%), and temporary break (6%), with the greatest difference between PIs over NNRTIs for adverse events (Table 2).

**Table 2:** Reasons listed for treatment disruption events.

Reason / Barrier		PI	NNRTI	Total
Treatment Record^a^				
Adverse event	*n* (%)	24 (37%)	19 (31%)	43 (34%)
Viral failure	*n* (%)	13 (20%)	15 (25%)	28 (22%)
Caregiver request	*n* (%)	11 (17%)	12 (20%)	23 (18%)
Non-adherence	*n* (%)	6 (9%)	3 (5%)	9 (7%)
Temporary break	*n* (%)	3 (5%)	5 (8%)	8 (6%)
Unknown	*n* (%)	5 (8%)	1 (2%)	6 (5%)
Drug supply problem	*n* (%)	1 (2%)	2 (3%)	3 (2%)
Intercurrent illness	*n* (%)	0 (0%)	2 (3%)	2 (2%)
Resistance	*n* (%)	1 (2%)	1 (2%)	2 (2%)
Parent forgot	*n* (%)	1 (2%)	0 (0%)	1 (1%)
Simplification	*n* (%)	0 (0%)	1 (2%)	1 (1%)
Treatment record total	*n*	65	61	126
Adherence Questionnaire^b^				
Forgot/lack of support	*n* (%)	10 (29%)	10 (30%)	20 (30%)
Ran out of drug	*n* (%)	8 (24%)	9 (27%)	17 (25%)
Problems taking some of the drugs (*e*.*g*., intolerance, taste, medication volume)	*n* (%)	11 (32%)	5 (15%)	16 (24%)
Fear of disclosure to others	*n* (%)	10 (29%)	5 (15%)	15 (22%)
Patient refused/didn’t want to take drugs	*n* (%)	10 (29%)	4 (12%)	14 (21%)
Scheduling/lifestyle interference	*n* (%)	9 (26%)	3 (9%)	12 (18%)
Drug toxicity concerns	*n* (%)	7 (21%)	4 (12%)	11 (16%)
Supervised by someone else or multiple caregivers	*n* (%)	6 (18%)	5 (15%)	11 (16%)
Patient unwell	*n* (%)	6 (18%)	4 (12%)	10 (15%)
Other	*n* (%)	4 (12%)	5 (15%)	9 (13%)
Different routine/change in living situation	*n* (%)	3 (9%)	4 (12%)	7 (10%)
Fed up giving/taking drugs	*n* (%)	3 (9%)	2 (6%)	5 (7%)
Think medication is not needed or not helping	*n* (%)	2 (6%)	2 (6%)	4 (6%)
Caregiver unwell/depressed	*n* (%)	0 (0%)	0 (0%)	0 (0%)
Total listed problems on questionnaire^b^	*n*	89	62	151
Total participants with questionnaire-reported missed doses	*n*	34	33	67
Total Treatment Disruption Events^c^	*n*	97	94	191

*n*, subsample size or number of events; NNRTI = non-nucleoside reverse transcriptase inhibitor; PI = protease inhibitor.

^a^ One category allowed per treatment record change or stop.

^b^ Participants may have answered in more than one category.

^c^ Some participants had both a treatment record and adherence questionnaire event at the same time.

Reports of missed doses on adherence questionnaires were balanced between PI and NNRTI arms, as 35% of non-adherence events in each arm were from patient or caregiver reports. The most common questionnaire-reported barriers to adherence, forgetting/lacking support (30%) or running out of medications (25%), were balanced between PI and NNRTI regimens. Other common questionnaire-reported adherence problems—including difficulties with administration, such as those attributed to intolerance, taste, patient refusal (24%); fear of disclosure to others (22%); patient refusal (21%); difficulties with scheduling or lifestyle (18%); and concerns about drug toxicity (16%)—were more frequently reported in participants in the PI arm (Table 2).

In sensitivity analyses, modifications of the outcome definition did not result in substantial hazard ratio changes. Point estimates at 4 years remained similar to the primary analysis when restricting events on the treatment record (or any event on questionnaire) to only ART regimen stops or changes lasting >3 days (unadjusted HR 1.16, 95% CI 0.85–1.57; adjusted HR 1.19, 95% CI 0.88–1.63), only ART regimen stops or changes lasting >14 days (unadjusted HR 1.27, 95% CI 0.93–1.74; adjusted HR 1.32, 95% CI 0.96–1.81), or only stops or changes including the PI or NNRTI drug (unadjusted HR 1.14, 95% CI 0.84–1.55; adjusted HR 1.18, 95% CI 0.87–1.61).

## Discussion

In PENPACT-1, our estimates were not compatible with large differences in time to treatment disruption between participants randomized to PIs versus NNRTIs. Point estimates were mildly in the direction of more treatment disruptions in PI-based regimens, particularly in the primary end point of 4 years, but differences were small, possibly due to chance, and appeared to decrease by study end. Exploration of reasons for treatment disruptions suggested that PI-based regimens may be less tolerable, both due to adverse events leading to treatment stoppages or substitutions and to regimen-specific adherence barriers reported on the adherence questionnaire. However, these PI-associated difficulties did not interrupt continuous therapy to the initial PI-based regimens more than they did to NNRTI-based regimens.

Although we did not find a meaningful difference in treatment disruptions in PI vs. NNRTI-based regimens, the secondary analyses exploring reasons for treatment disruptions suggested that administration of a PI-based regimen to a child may be a struggle, even if not resulting in actual missed doses. The treatment record suggested that participants experienced more adverse events to PIs over NNRTIs, but adherence questionnaire responses formed a pattern of difficulties with PI tolerability, whether attributed to taste, medication volume or pill burden, toxicity, or simply patient refusal. This pattern would be consistent with existing literature on PI vs. NNRTI regimens. PIs have higher drug toxicity, especially gastrointestinal side effects, and intolerance, particularly regarding their noxious taste [7, 18–20, 25–27]. Even if children are able to swallow pills, certain PIs are available only as large pills [28, 29]. At the time of PENPACT-1, no PIs were available as complete-regimen combinations for children, whereas single-tablet NNRTI regimens could facilitate adherence through administration of fewer pills [2, 30–34]. More recently, a novel four-in-one fixed-dose combination of abacavir, lamivudine, and LPV/r granule-filled capsules has been under study and submitted to the FDA for approval [35]. Participants reported more barriers to adherence in PIs related to scheduling or lifestyle interference, which may relate to dosing frequency. We hypothesize that increased fear of disclosure to others, as noted in the PI arm, may relate to difficulties concealing drug administration when given more frequently. Higher dosing frequency has been associated with more frequent treatment disruptions [20, 30, 33, 34, 36–39]. Some NNRTIs, most notably efavirenz, have more suitable pharmacokinetics for once daily administration. In our study, most PI-based regimens were administered at least twice daily, whereas some commonly used NNRTI-based regimens allowed once-daily dosing.

Most children in PENPACT-1 experienced a treatment disruption event during the study. Only about one-third of participants remained continuously on their initial ART at 4 years; only one-sixth remained continuously on initial ART at study end. These results are consistent with other pediatric data on the durability of first-line treatment regimens [40]. Maintaining continuous therapy on ART is critical to sustained HIV-related outcomes, as suppressing viral load decreases the probability of HIV sub-populations acquiring antiretroviral resistance mutations and chances of forward infection [41–49]. Although optimal adherence targets vary by PI vs. NNRTI class, adherence has been modest across ART studies, especially patients failing to achieve viral suppression [12, 16, 17, 30, 50–56]. Notably, ART appears to be less successful in producing viral suppression in children, who are more prone to viral failure and resistance due to higher plasma viral loads, less robust antiviral immune responses, greater pharmacokinetic variability, and social dependency [44, 57]. Adolescents have particularly worse viral and immunological outcomes, due to poor ART adherence [48, 49, 58–60]. The large proportion of children in PENPACT-1 with disruptions of their initial ART raises concerns regarding long-term durability, especially as these patients were receiving adherence support on a clinical trial protocol at specialty pediatric HIV centers.

Based on our data, choice of an initial PI- vs. NNRTI-based regimen may not have a major impact on ART treatment disruption. Despite differences in reported regimen-related adherence barriers, participants in both treatment arms persevered in taking their regimens similarly. Moreover, the most common questionnaire-reported barriers were not regimen-specific: forgetting/lack of support and running out of drug. Novel interventions may still be able to improve the experience of drug administration. Pediatric pellets are heat-stable and generally more acceptable than syrups, but palatability and administration problems persist and may increase over time [61–63]. Pediatric granules, especially in the four-in-one combination, may improve palatability and decrease pill burden [35, 64]. Precision medicine related to taste-sensing genotypes may hold promise for prescribing according to individualized palatability [65]. In adult data, integrase strand transferase inhibitors (INSTIs) have been at least as tolerable as PIs or NNRTIs, if not more so, and INSTIs are increasingly preferred drugs in children [66–69]. Nevertheless, a primary goal of optimizing continuous therapy to ART is durable viral suppression, which was comparable across PI vs. NNRTI arms in this study’s parent trial, although similar trials had variable results [23, 70–74]. In this study population, choice of either PI- or NNRTI-based initial ART appears acceptable.

Our estimates of treatment disruption may have had measurement error. First, we had no direct measures of drug exposure, such as therapeutic drug monitoring. Treatment records captured only prescribing events and documented ART disruptions, and the adherence questionnaires relied on accurate reporting by either the child or the caregiver, if present and willing to answer. Although we relied on a questionnaire that has previously been validated [24], reporting biases and unanswered questionnaires may have affected our measures of missed doses. Our combining treatment records and adherence questionnaires into a composite outcome should have decreased measurement error from either instrument individually. Second, adherence questionnaires in this study focused on ART adherence over the 3 days prior to the most recent visit and inquired about adherence barriers encountered over the prior 2 weeks, rather than a daily measure of adherence throughout the study. The time-varying nature of treatment disruption means that patients may have experienced an initial or temporary period of treatment disruption that was subsequently corrected [75, 76], but our analysis presents only data on time to first event of treatment disruption. Third, limited participant report of individual drugs missed on the adherence questionnaire precluded definitive identification of treatment disruptions of individual drugs. Instead, we assessed treatment disruption to any component of the ART regimen. Fourth, heterogeneity of adherence questionnaires across networks, ages, and respondents regarding barriers to therapy should caution against rigorous interpretation of reasons for treatment disruptions. Finally, this study size was not sufficient to distinguish differences on the order of 7%, as was seen at 4 years.

## Conclusions

In conclusion, children in PENPACT-1 had similar time to treatment disruption for initial PI-based regimens and NNRTI-based regimens. Although secondary analyses suggest that PI-based regimens may be more difficult to tolerate and may be less convenient to administer, these difficulties did not result in a large difference in children stopping, changing, or missing doses at 4 years (PI 70%, NNRTI 63%), and any suggested differences diminished by study end (PI 81%, NNRTI 84%). Initial ART with either a PI or NNRTI may be acceptable for maintaining continuous therapy on ART in children.

## Supporting information

S1 Checklist CONSORT checklist. (DOC)

S1 Protocol PENPACT-1 trial protocol. (PDF)

## References

[1] Joint United Nations Programme on HIV/AIDS. UNAIDS Data 2018 Geneva, Switzerland: UNAIDS; 2018 [cited 2019 Jun 21]. Available at: https://www.unaids.org/sites/default/files/media_asset/unaids-data-2018_en.pdf.

[2] Van der Linden D, Callens S, Brichard B, Colebunders R. Pediatric HIV: new opportunities to treat children. Expert Opin Pharmacother. 2009;10(11):1783–91. 10.1517/14656560903012377 19558340

[3] Brichard B, Van der Linden D. Clinical practice treatment of HIV infection in children. Eur J Pediatr. 2009;168(4):387–92. 10.1007/s00431-008-0914-8 19152000

[4] Leonard EG, McComsey GA. Antiretroviral therapy in HIV-infected children: the metabolic cost of improved survival. Infect Dis Clin North Am. 2005;19(3):713–29. 10.1016/j.idc.2005.05.006 16102657

[5] Leonard EG, McComsey GA. Metabolic complications of antiretroviral therapy in children. Pediatr Infect Dis J. 2003;22(1):77–84. 10.1097/00006454-200301000-00018 12544413

[6] Mellins CA, Brackis-Cott E, Dolezal C, Abrams EJ. The role of psychosocial and family factors in adherence to antiretroviral treatment in human immunodeficiency virus-infected children. Pediatr Infect Dis J. 2004;23(11):1035–41. 10.1097/01.inf.0000143646.15240.ac 15545859

[7] Buchanan AL, Montepiedra G, Sirois PA, Kammerer B, Garvie PA, Storm DS, et al Barriers to medication adherence in HIV-infected children and youth based on self- and caregiver report. Pediatrics. 2012;129(5):e1244–51. 10.1542/peds.2011-1740 22508915 PMC3340587

[8] Dabis F, Elenga N, Meda N, Leroy V, Viho I, Manigart O, et al 18-Month mortality and perinatal exposure to zidovudine in West Africa. AIDS. 2001;15(6):771–9. 10.1097/00002030-200104130-00013 11371692

[9] Violari A, Cotton MF, Gibb DM, Babiker AG, Steyn J, Madhi SA, et al Early antiretroviral therapy and mortality among HIV-infected infants. N Engl J Med. 2008;359(21):2233–44. 10.1056/NEJMoa0800971 19020325 PMC2950021

[10] Becquet R, Marston M, Dabis F, Moulton LH, Gray G, Coovadia HM, et al Children who acquire HIV infection perinatally are at higher risk of early death than those acquiring infection through breastmilk: a meta-analysis. PLoS One. 2012;7(2):e28510 10.1371/journal.pone.0028510 22383946 PMC3285615

[11] Edessa D, Sisay M, Asefa F. Second-line HIV treatment failure in sub-Saharan Africa: A systematic review and meta-analysis. PLoS One. 2019;14(7):e0220159 10.1371/journal.pone.0220159 31356613 PMC6663009

[12] Pham PA. Antiretroviral adherence and pharmacokinetics: review of their roles in sustained virologic suppression. AIDS Patient Care STDS. 2009;23(10):803–7. 10.1089/apc.2008.0269 19795999

[13] Gross R YB, Wood E, Bangsberg D, Justice AC, Montaner JS. Boosted PIs are more forgiving of suboptimal adherence than nonboosted PIs or NNRTIs. 13th Conference on Retroviruses and Opportunistic Infections; Denver, CO, USA; 2006.

[14] Harrison L, Melvin A, Fiscus S, Saidi Y, Nastouli E, Harper L, et al HIV-1 Drug Resistance and Second-Line Treatment in Children Randomized to Switch at Low Versus Higher RNA Thresholds. J Acquir Immune Defic Syndr. 2015;70(1):42–53. 10.1097/QAI.0000000000000671 26322666 PMC4556171

[15] Agwu A, Lindsey JC, Ferguson K, Zhang H, Spector S, Rudy BJ, et al Analyses of HIV-1 drug-resistance profiles among infected adolescents experiencing delayed antiretroviral treatment switch after initial nonsuppressive highly active antiretroviral therapy. AIDS Patient Care STDS. 2008;22(7):545–52. 10.1089/apc.2007.0200 18479228 PMC2929158

[16] Bangsberg DR, Acosta EP, Gupta R, Guzman D, Riley ED, Harrigan PR, et al Adherence-resistance relationships for protease and non-nucleoside reverse transcriptase inhibitors explained by virological fitness. AIDS. 2006;20(2):223–31. 10.1097/01.aids.0000199825.34241.49 16511415

[17] Muller AD, Myer L, Jaspan H. Virological suppression achieved with suboptimal adherence levels among South African children receiving boosted protease inhibitor-based antiretroviral therapy. Clin Infect Dis. 2009;48(1):e3–5. 10.1086/595553 19025495

[18] Bain-Brickley D, Butler LM, Kennedy GE, Rutherford GW. Interventions to improve adherence to antiretroviral therapy in children with HIV infection. Cochrane Database Syst Rev. 2011(12):CD009513 10.1002/14651858.CD009513 22161452 PMC6599820

[19] Davies MA, Boulle A, Fakir T, Nuttall J, Eley B. Adherence to antiretroviral therapy in young children in Cape Town, South Africa, measured by medication return and caregiver self-report: a prospective cohort study. BMC Pediatr. 2008;8:34 10.1186/1471-2431-8-34 18771599 PMC2533648

[20] Esteban Gomez MJ, Escobar Rodriguez I, Vicario Zubizarreta MJ, Climente Pollan J, Herreros de Tejada A. [Influence of antiretroviral therapy characteristics on pediatric patient adherence]. Farmacia Hosp. 2004;28(6 Suppl 1):34–9. 15649114

[21] Schlatter AF, Deathe AR, Vreeman RC. The Need for Pediatric Formulations to Treat Children with HIV. AIDS Res Treat. 2016;2016:1654938 10.1155/2016/1654938 27413548 PMC4927993

[22] Lin D, Seabrook JA, Matsui DM, King SM, Rieder MJ, Finkelstein Y. Palatability, adherence and prescribing patterns of antiretroviral drugs for children with human immunodeficiency virus infection in Canada. Pharmacoepidemiol Drug Saf. 2011;20(12):1246–52. 10.1002/pds.2236 21936016

[23] PENPACT-1 (PENTA 9/PACTG 390) Study Team, Babiker A, Castro nee Green H, Compagnucci A, Fiscus S, Giaquinto C, et al First-line antiretroviral therapy with a protease inhibitor versus non-nucleoside reverse transcriptase inhibitor and switch at higher versus low viral load in HIV-infected children: an open-label, randomised phase 2/3 trial. Lancet Infect Dis. 2011;11(4):273–83. 10.1016/S1473-3099(10)70313-3 21288774 PMC3111069

[24] Van Dyke RB, Lee S, Johnson GM, Wiznia A, Mohan K, Stanley K, et al Reported adherence as a determinant of response to highly active antiretroviral therapy in children who have human immunodeficiency virus infection. Pediatrics. 2002;109(4):e61 10.1542/peds.109.4.e61 11927734

[25] Bhattacharya M, Dubey AP. Adherence to antiretroviral therapy and its correlates among HIV-infected children at an HIV clinic in New Delhi. Ann Trop Paediatr. 2011;31(4):331–7. 10.1179/1465328111Y.0000000031 22041467

[26] Elise A, France AM, Louise WM, Bata D, Francois R, Roger S, et al Assessment of adherence to highly active antiretroviral therapy in a cohort of African HIV-infected children in Abidjan, Cote d'Ivoire. J Acquir Immune Defic Syndr. 2005;40(4):498–500. 10.1097/01.qai.0000168180.76405.5f 16280708

[27] Giacomet V, Albano F, Starace F, de Franciscis A, Giaquinto C, Gattinara GC, et al Adherence to antiretroviral therapy and its determinants in children with human immunodeficiency virus infection: a multicentre, national study. Acta Paediatr. 2003;92(12):1398–402. 10.1080/08035250310006737 14971789

[28] Czyzewski D RD, Lopez M, et al Teaching and maintaining pill swallowing in HIV-infected children. AIDS Read. 2000;10(2):88–94.

[29] Garvie PA, Lensing S, Rai SN. Efficacy of a pill-swallowing training intervention to improve antiretroviral medication adherence in pediatric patients with HIV/AIDS. Pediatrics. 2007;119(4):e893–9. 10.1542/peds.2006-1488 17353298

[30] Maggiolo F, Ravasio L, Ripamonti D, Gregis G, Quinzan G, Arici C, et al Similar adherence rates favor different virologic outcomes for patients treated with nonnucleoside analogues or protease inhibitors. Clin Infect Dis. 2005;40(1):158–63. 10.1086/426595 15614706

[31] Kapadia SN, Grant RR, German SB, Singh B, Davidow AL, Swaminathan S, et al HIV virologic response better with single-tablet once daily regimens compared to multiple-tablet daily regimens. SAGE Open Med. 2018;6:2050312118816919 10.1177/2050312118816919 30574301 PMC6295695

[32] Clay PG, Nag S, Graham CM, Narayanan S. Meta-Analysis of Studies Comparing Single and Multi-Tablet Fixed Dose Combination HIV Treatment Regimens. Medicine. 2015;94(42):e1677 10.1097/MD.0000000000001677 26496277 PMC4620781

[33] Nachega JB, Parienti JJ, Uthman OA, Gross R, Dowdy DW, Sax PE, et al Lower pill burden and once-daily antiretroviral treatment regimens for HIV infection: A meta-analysis of randomized controlled trials. Clin Infect Dis. 2014;58(9):1297–307. 10.1093/cid/ciu046 24457345 PMC3982838

[34] Pantuzza LL, Ceccato M, Silveira MR, Junqueira LMR, Reis AMM. Association between medication regimen complexity and pharmacotherapy adherence: a systematic review. Eur J Clin Pharmacol. 2017;73(11):1475–89. 10.1007/s00228-017-2315-2 28779460

[35] Penazzato M, Townsend CL, Rakhmanina N, Cheng Y, Archary M, Cressey TR, et al Prioritising the most needed paediatric antiretroviral formulations: the PADO4 list. Lancet HIV. 2019;6(9):e623–e631. 10.1016/S2352-3018(19)30193-6 31498110

[36] Barro M, Some J, Foulongne V, Diasso Y, Zoure E, Hien H, et al Short-term virological efficacy, immune reconstitution, tolerance, and adherence of once-daily dosing of didanosine, lamivudine, and efavirenz in HIV-1-infected African children: ANRS 12103 Burkiname. J Acquir Immune Defic Syndr. 2011;57 Suppl 1:S44–9. 21857286 10.1097/QAI.0b013e31821fd64f

[37] Biadgilign S, Deribew A, Amberbir A, Deribe K. Barriers and facilitators to antiretroviral medication adherence among HIV-infected paediatric patients in Ethiopia: A qualitative study. SAHARA J. 2009;6(4):148–54. 10.1080/17290376.2009.9724943 20485854

[38] Brackis-Cott E, Mellins CA, Abrams E, Reval T, Dolezal C. Pediatric HIV medication adherence: the views of medical providers from two primary care programs. J Pediatr Health Care. 2003;17(5):252–60. 10.1016/s0891-5245(02)88313-4 14576630

[39] Buscher A, Hartman C, Kallen MA, Giordano TP. Impact of antiretroviral dosing frequency and pill burden on adherence among newly diagnosed, antiretroviral-naive HIV patients. Int J STD AIDS. 2012;23(5):351–5. 10.1258/ijsa.2011.011292 22648890 PMC3642979

[40] Fortuin-de Smidt M, de Waal R, Cohen K, Technau KG, Stinson K, Maartens G, et al First-line antiretroviral drug discontinuations in children. PLoS One. 2017;12(2):e0169762 10.1371/journal.pone.0169762 28192529 PMC5305232

[41] Bangsberg DR, Hecht FM, Charlebois ED, Zolopa AR, Holodniy M, Sheiner L, et al Adherence to protease inhibitors, HIV-1 viral load, and development of drug resistance in an indigent population. AIDS. 2000;14(4):357–66. 10.1097/00002030-200003100-00008 10770537

[42] Bartlett JA. Addressing the challenges of adherence. J Acquir Immune Defic Syndr. 2002;29 Suppl 1:S2–10. 10.1097/00126334-200202011-00002 11832696

[43] Paterson DL, Swindells S, Mohr J, Brester M, Vergis EN, Squier C, et al Adherence to protease inhibitor therapy and outcomes in patients with HIV infection. Ann Intern Med. 2000;133(1):21–30. 10.7326/0003-4819-133-1-200007040-00004 10877736

[44] Chen TK, Aldrovandi GM. Review of HIV antiretroviral drug resistance. Pediatr Infect Dis J. 2008;27(8):749–52. 10.1097/INF.0b013e3181846e2e 18664987 PMC3909718

[45] Germanaud D, Derache A, Traore M, Madec Y, Toure S, Dicko F, et al Level of viral load and antiretroviral resistance after 6 months of non-nucleoside reverse transcriptase inhibitor first-line treatment in HIV-1-infected children in Mali. J Antimicrob Chemother. 2010;65(1):118–24. 10.1093/jac/dkp412 19933171

[46] Patten G, Schomaker M, Davies MA, Rabie H, van Zyl G, Technau K, et al What Should We Do When HIV-positive Children Fail First-line Combination Antiretroviral Therapy? A Comparison of 4 ART Management Strategies. Pediatr Infect Dis J. 2019;38(4):400–5. 10.1097/INF.0000000000002156 30882732 PMC6355383

[47] Cohen MS, Chen YQ, McCauley M, Gamble T, Hosseinipour MC, Kumarasamy N, et al Antiretroviral Therapy for the Prevention of HIV-1 Transmission. New Engl J Med. 2016;375(9):830–9. 10.1056/NEJMoa1600693 27424812 PMC5049503

[48] Flynn PM, Rudy BJ, Douglas SD, Lathey J, Spector SA, Martinez J, et al Virologic and immunologic outcomes after 24 weeks in HIV type 1-infected adolescents receiving highly active antiretroviral therapy. J Infect Dis. 2004;190(2):271–9. 10.1086/421521 15216461

[49] Flynn PM, Rudy BJ, Lindsey JC, Douglas SD, Lathey J, Spector SA, et al Long-term observation of adolescents initiating HAART therapy: three-year follow-up. AIDS Res Hum Retroviruses. 2007;23(10):1208–14. 10.1089/aid.2006.0290 17961106

[50] Bangsberg DR, Kroetz DL, Deeks SG. Adherence-resistance relationships to combination HIV antiretroviral therapy. Curr HIV/AIDS Rep. 2007;4(2):65–72. 10.1007/s11904-007-0010-0 17547827

[51] Gardner EM, Burman WJ, Steiner JF, Anderson PL, Bangsberg DR. Antiretroviral medication adherence and the development of class-specific antiretroviral resistance. AIDS. 2009;23(9):1035–46. 10.1097/QAD.0b013e32832ba8ec 19381075 PMC2704206

[52] Bangsberg DR. Less than 95% adherence to nonnucleoside reverse-transcriptase inhibitor therapy can lead to viral suppression. Clin Infect Dis. 2006;43(7):939–41. 10.1086/507526 16941380

[53] Maggiolo F, Airoldi M, Kleinloog HD, Callegaro A, Ravasio V, Arici C, et al Effect of adherence to HAART on virologic outcome and on the selection of resistance-conferring mutations in NNRTI- or PI-treated patients. HIV Clin Trials. 2007;8(5):282–92. 10.1310/hct0805-282 17956829

[54] Golin CE, Liu H, Hays RD, Miller LG, Beck CK, Ickovics J, et al A prospective study of predictors of adherence to combination antiretroviral medication. J Gen Intern Med. 2002;17(10):756–65. 10.1046/j.1525-1497.2002.11214.x 12390551 PMC1495120

[55] Bangsberg DR, Deeks SG. Is average adherence to HIV antiretroviral therapy enough? J Gen Intern Med. 2002;17(10):812–3. 10.1046/j.1525-1497.2002.20812.x 12390559 PMC1495121

[56] Gross R, Bilker WB, Friedman HM, Strom BL. Effect of adherence to newly initiated antiretroviral therapy on plasma viral load. AIDS. 2001;15(16):2109–17. 10.1097/00002030-200111090-00006 11684930

[57] De Rossi A. Virological and immunological response to antiretroviral therapy in HIV-1 infected children: genotypic and phenotypic assays in monitoring virological failure. New Microbiol. 2004;27(2 Suppl 1):45–50. 15646064

[58] Filho LF, Nogueira SA, Machado ES, Abreu TF, de Oliveira RH, Evangelista L, et al Factors associated with lack of antiretroviral adherence among adolescents in a reference centre in Rio de Janeiro, Brazil. Int J STD AIDS. 2008;19(10):685–8. 10.1258/ijsa.2008.008017 18824621 PMC2857528

[59] Foster C, Fidler S. Optimizing antiretroviral therapy in adolescents with perinatally acquired HIV-1 infection. Expert Rev Anti Infect Ther. 2010;8(12):1403–16. 10.1586/eri.10.129 21133665

[60] Kacanek D, Huo Y, Malee K, Mellins CA, Smith R, Garvie PA, et al Nonadherence and unsuppressed viral load across adolescence among US youth with perinatally acquired HIV. AIDS. 2019;33(12):1923–34. 10.1097/QAD.0000000000002301 31274538 PMC6776473

[61] Kekitiinwa A, Musiime V, Thomason MJ, Mirembe G, Lallemant M, Nakalanzi S, et al Acceptability of lopinavir/r pellets (minitabs), tablets and syrups in HIV-infected children. Antivir Ther. 2016;21(7):579–585. 10.3851/IMP3054 27128199 PMC6029664

[62] Pasipanodya B, Kuwenga R, Prust ML, Stewart B, Chakanyuka C, Murimwa T, et al Assessing the adoption of lopinavir/ritonavir oral pellets for HIV-positive children in Zimbabwe. J Int AIDS Soc. 2018;21(12):e25214 10.1002/jia2.25214 30549217 PMC6293134

[63] Giralt AN, Nöstlinger C, Lee J, Salami O, Lallemant M, Onyango-Ouma W, et al Understanding acceptance of and adherence to a new formulation of paediatric antiretroviral treatment in the form of pellets (LPV/r)-A realist evaluation. PLoS One. 2019;14(8):e0220408 10.1371/journal.pone.0220408 31433803 PMC6703671

[64] Pham K, Li D, Guo S, Penzak S, Dong X. Development and in vivo evaluation of child-friendly lopinavir/ritonavir pediatric granules utilizing novel in situ self-assembly nanoparticles. J Control Release. 2016;226:88–97. 10.1016/j.jconrel.2016.02.001 26849919

[65] Mennella JA, Mathew PS, Lowenthal ED. Use of Adult Sensory Panel to Study Individual Differences in the Palatability of a Pediatric HIV Treatment Drug. Clin Ther. 2017;39(10):2038–48. 10.1016/j.clinthera.2017.08.012 28923290 PMC5654675

[66] Lennox JL, DeJesus E, Lazzarin A, Pollard RB, Madruga JV, Berger DS, et al Safety and efficacy of raltegravir-based versus efavirenz-based combination therapy in treatment-naive patients with HIV-1 infection: a multicentre, double-blind randomised controlled trial. Lancet. 2009;374(9692):796–806. 10.1016/S0140-6736(09)60918-1 19647866

[67] Molina JM, Clotet B, van Lunzen J, Lazzarin A, Cavassini M, Henry K, et al Once-daily dolutegravir is superior to once-daily darunavir/ritonavir in treatment-naive HIV-1-positive individuals: 96 week results from FLAMINGO. J Int AIDS Soc. 2014;17(4 Suppl 3):19490 10.7448/IAS.17.4.19490 25393999 PMC4224885

[68] Snedecor SJ, Radford M, Kratochvil D, Grove R, Punekar YS. Comparative efficacy and safety of dolutegravir relative to common core agents in treatment-naive patients infected with HIV-1: a systematic review and network meta-analysis. BMC Infect Dis. 2019;19(1):484 10.1186/s12879-019-3975-6 31146698 PMC6543679

[69] Panel on Antiretrviral Therapy and Medical Management of Children Living with HIV. Guidelines for the Use of Antiretroviral Agents in Pediatric HIV Infection [cited 2019 Nov 27]. Available from: http://aidsinfo.nih.gov/contentfiles/lvguidelines/pediatricguidelines.pdf.

[70] Coovadia A, Abrams EJ, Stehlau R, Meyers T, Martens L, Sherman G, et al Reuse of nevirapine in exposed HIV-infected children after protease inhibitor-based viral suppression: a randomized controlled trial. JAMA. 2010;304(10):1082–90. 10.1001/jama.2010.1278 20823434 PMC4540068

[71] Violari A, Lindsey JC, Hughes MD, Mujuru HA, Barlow-Mosha L, Kamthunzi P, et al Nevirapine versus ritonavir-boosted lopinavir for HIV-infected children. N Engl J Med. 2012;366(25):2380–9. 10.1056/NEJMoa1113249 22716976 PMC3443859

[72] Ruel TD, Kakuru A, Ikilezi G, Mwangwa F, Dorsey G, Rosenthal PJ, et al Virologic and immunologic outcomes of HIV-infected Ugandan children randomized to lopinavir/ritonavir or nonnucleoside reverse transcriptase inhibitor therapy. J Acquir Immune Defic Syndr. 2014;65(5):535–41. 10.1097/QAI.0000000000000071 24326597 PMC3999287

[73] Barlow-Mosha L, Angelidou K, Lindsey J, Archary M, Cotton M, Dittmer S, et al Nevirapine- Versus Lopinavir/Ritonavir-Based Antiretroviral Therapy in HIV-Infected Infants and Young Children: Long-term Follow-up of the IMPAACT P1060 Randomized Trial. Clin Infect Dis. 2016;63(8):1113–21. 10.1093/cid/ciw488 27439527 PMC5036919

[74] Murnane PM, Strehlau R, Shiau S, Patel F, Mbete N, Hunt G, et al Switching to Efavirenz Versus Remaining on Ritonavir-boosted Lopinavir in Human Immunodeficiency Virus-infected Children Exposed to Nevirapine: Long-term Outcomes of a Randomized Trial. Clin Infect Dis. 2017;65(3):477–85. 10.1093/cid/cix335 28419200 PMC5849095

[75] Byakika-Tusiime J, Crane J, Oyugi JH, Ragland K, Kawuma A, Musoke P, et al Longitudinal antiretroviral adherence in HIV+ Ugandan parents and their children initiating HAART in the MTCT-Plus family treatment model: role of depression in declining adherence over time. AIDS Behav. 2009;13 Suppl 1:82–91. 10.1007/s10461-009-9546-x 19301113

[76] Giannattasio A, Albano F, Giacomet V, Guarino A. The changing pattern of adherence to antiretroviral therapy assessed at two time points, 12 months apart, in a cohort of HIV-infected children. Expert Opin Pharmacother. 2009;10(17):2773–8. 10.1517/14656560903376178 19929700

