# Supplementary material for: Time to treatment disruption in children with HIV-1 randomized to initial antiretroviral therapy with protease inhibitors versus non-nucleoside reverse transcriptase inhibitors
Source: PLoS One. 2020 Nov 23;15(11):e0242405. doi: 10.1371/journal.pone.0242405 (PMC7682873; doi:10.1371/journal.pone.0242405)
Supplement: S1 Protocol — (PDF) [file pone.0242405.s002.pdf]

PENPACT 1
(PENTA 9/PACTG 390)

A PHASE II/III RANDOMIZED, OPEN-LABEL STUDY OF COMBINATION ANTIRETROVIRAL REGIMENS AND TREATMENT-SWITCHING STRATEGIES IN **HIV-1-INFECTED** ANTIRETROVIRAL NAIVE CHILDREN >30 DAYS AND <18 YEARS OF AGE

An International, Multicenter Trial of the
Pediatric AIDS Clinical Trials Group (PACTG) and the
Paediatric European Network for Treatment of AIDS (PENTA)

Funded by:

The National Institute of Allergy and Infectious Diseases (NIAID),
The National Institute of Child Health and Human Development (NICHD),
and the European Commission

Food and Drug Administration (FDA) IND # 64,535
for PACTG 390

PACTG Primary Therapy Research Agenda Committee Chair: Ram Yogev, M.D.

PACTG Protocol Co-Chair: Ross E. McKinney, Jr., M.D.

PACTG Protocol Co-Chair: Ann J. Melvin, M.D.

Division of AIDS Medical Officer: Elizabeth Smith, M.D.

Clinical Trials Specialist: **Jennifer L. Gardella, B.S.**

PENTA Executive Committee: Carlo Giaquinto, M.D. (Chair)
Jean Pierre Aboulker, Ph.D.
Abdel Babiker, Ph.D.
Janet Darbyshire, OBE, FRCP, MSc.
Diana Gibb, M.D.
Gareth Tudor-Williams, M.D.

VERSION **3.1**
FINAL
November 7th 2007

FOREWORD

The PACTG and the PENTA teams wrote this protocol document with the idea of establishing the first of many possible joint studies between these two organizations. PENPACT 1 offers the opportunity of one document for actually two different studies, PACTG 390 **at PACTG affiliated domestic and international sites**, and PENTA 9 in Europe. PENPACT 1 will combine the data from these two studies, for a larger sample size and to achieve higher statistical power. Although this document will be used by both PACTG and PENTA as the official protocol, each group will manage its own approval process through its own scientific and regulatory committees.

The FDA will be the regulatory agency granting the IND for the PACTG 390 study **and will cover those children enrolled at domestic and international sites affiliated with the PACTG**. The PENTA 9 study will be submitted for regulatory and ethical approval in each PENTA country involved**, including Brazil where the protocol will be submitted for regulatory and ethical approval at the local and national levels.** Additionally, scientific review of the PENTA 9 will be conducted by the Medical Research Council (MRC) in the UK and the Agence Nationale de Recherches sur le SIDA (ANRS) in France. The PENPACT 1 Team believes that the number of children, who will enroll in PACTG 390 will be approximately 128 or half of the targeted accrual number.

Throughout the body of this document, both the common and unique items for the two research organizations are clearly explained. All appendices are labeled as either applicable to both organizations or to one of the two groups.

Both groups will always follow the current version of the protocol document; any amendment requested by either one of the two organizations will affect the other **organization**. Amendments will follow the established approval procedures for each organization and will not be implemented until both organizations have finalized their approvals.

All the study medications will be given by prescription. The children, their parent(s) or legally **authorized** representative **(LAR)**, the children's health insurance, and/or, in Europe, the healthcare provider, are responsible for purchasing the study medications. However, only the medicines, doses, and regimens described in this document will be allowed as the children's antiretroviral therapy. Any new medication, dose, or regimen will be considered for incorporation into PENPACT 1; however, an amendment will be necessary before prescription of the new medicine, dose, or regimen is allowed.

The PENPACT 1 Team thoroughly discussed during protocol development the existence of some variations in the dosing guidelines for some of the drugs between the USA and Europe. Therefore, the protocol team developed a single appendix to describe all the allowed drugs and dosing for the protocol, Appendix IX *"ALLOWED ANTIRETROVIRAL THERAPY*

BACKGROUND INFORMATION, SUGGESTED DOSING AND MAJOR TOXICITIES FOR PACTG AND PENTA."

The PENPACT 1 Team understands that maintaining one protocol document for both groups is a difficult and confusing task. However, in this four-year study having only one protocol document may be essential in maintaining the integrity of the objectives of the study.

PENPACT 1 PROTOCOL TEAM ROSTER

All questions concerning this protocol should be sent via e-mail to the PACTG at actg.penpact1@fstrf.org , which includes the Medical Research Council (MRC) at PENTA@ctu.mrc.ac.uk , the Institut National de la Santé et de la Recherche Médicale (INSERM)-France coordinated sites at a.compagnucci@vjf.inserm.fr ,and the Dipartimento di Pediatria, Università di Padova at carlog@child.pedi.unipd.it. Remember to include the child's PID or trial number when applicable. The appropriate person from PENPACT 1 Team will respond to your questions via e-mail with a "cc" to actg.penpact1@fstrf.org. A response should generally be received within 24 hours (Monday - Friday). **For** PACTG protocol registration questions, e-mail protocol@tech-res.com . For PACTG **EAE** questions, **e-mail RCCSafetyOffice@tech-res.com; or call 1-800-537-9979 or 301-897-1709; or fax 1-800-275-7619 or 301-897-1710.** To contact any of the team members directly via fax or telephone, from the USA and Canada: dial 1 + Code + phone number (Codes: UK=44, France=33, Italy=39, Spain=34, all other international dial numbers can be found at http://dial-a-code.com/).

PACTG

Protocol Co-Chairs

Ross E. McKinney, Jr., M.D.
Vice Dean for Research
Box 3461
Duke University Medical Center
Room 117A Dean's Suite, Davison Building
Durham, NC 27710
PHONE: (919) 684-0831
FAX: (919) 668-5239
E-mail: ross.mckinney@duke.edu

Ann J. Melvin, M.D.
Assistant Professor Pediatrics
Division of Infectious Diseases Children's Hospital and Medical Center
Mailstop CH-32
4800 Sand Point Way, N. E.
Seattle, WA 98105-0371
PHONE: (206) 987-2535
FAX: (206) 987-3890
E-mail: ann.melvin@seattlechildrens.org

PENTA

Executive Committee Chair[1]

Carlo Giaquinto, M.D.
Assistente
Dipartimento di Pediatria
Università di Padova
Via Giustiniani 3
35128 Padova, ITALY.
PHONE: 39 049 821 3585
FAX: 39 049 875 3865
E-mail: carlog@child.pedi.unipd.it

Medical Officers[2]

Diana Gibb, M.D. MRCP M.Sc.
Professor in Epidemiology/Hon Consultant Paediatrician
HIV Division
MRC Clinical Trials Unit, 222 Euston Road
LONDON NW1 2DA, UK
PHONE: 44 20 7670 4709
FAX: 44 20 7670 4818
E-mail: Di.Gibb@ctu.mrc.ac.uk

---

[1] Also Medical Officer in Italy.
[2] These Medical Officers will be the Medical Safety Monitors for PENTA for SAE related issues.

PENPACT 1 PROTOCOL TEAM ROSTER

PACTG

DAIDS Medical Officer

Elizabeth Smith, M.D.
Medical Monitor
NIH, NIAID, DAIDS, TRP, PMB
Rm. 5105
6700-B Rockledge Dr., MSC 7624
Bethesda, MD, 20892-7624
PHONE: (301) 402-3226
Fax: (301) 402-3171
E-mail: bs161v@nih.gov

NICHD Medical Officer

Lynne Mofenson, M.D.
Chief, Pediatric, Adolescent and Maternal AIDS Branch
CRMC, NICHD, NIH
6100 Executive Branch
Room 4B11
Rockville, MD 20852
PHONE: (301) **435-6870**
FAX: (301) 496-8678
E-mail: lm65d@nih.gov

Lead Statistician

Michael Hughes, Ph.D.
Director
Statistical & Data Analysis Center
Harvard School of Public Health
Building 2, Room 407
655 Huntington Avenue
Boston MA 02115-6017
PHONE: (617) 432-3161
FAX: (617) 432-2832
E-mail: mhughes@sdac.harvard.edu

PENTA

Medical Officers (Cont.)

Alexandra Compagnucci, M.D.
INSERM SC10, HIV Clinical Trial Centre
16 Avenue Paul Vaillant Couturier
Villejuif Cedex, 94807, France
PHONE: 33-14-559-5290
FAX: 33-14-559-5180
E-mail: a.compagnucci@vjf.inserm.fr

Lead Statistician

Abdel Babiker, Ph.D.
Head, HIV Division
MRC Clinical Trials Unit
222 Euston Road
LONDON NW1 2DA, UK
PHONE: 44 20 7670 47019
FAX: 44 20 7670 4818
E-mail: Abdel.Babiker@ctu.mrc.ac.uk

Protocol Statisticians

Sarah Walker, M.Sc., Ph.D.
Senior Statistician HIV Division
MRC Clinical Trials Unit
222 Euston Road
LONDON NW1 2DA, UK
PHONE: 44 20 7670 4726
FAX: 44 20 7670 4815
E-mail: Sarah.Walker@ctu.mrc.ac.uk

Hannah Green
Statistician
MRC Clinical Trials Unit
222 Euston Road
LONDON NW1 2DA, UK
PHONE: 44 20 7670 4733
FAX: 44 20 7670 4815
E-mail: Hannah.Green@ctu.mrc.ac.uk

PENPACT 1 PROTOCOL TEAM ROSTER

PACTG

Protocol Statistician

Jie Chen, M.S.
Statistician
Statistical and Data Analysis Center
Harvard School of Public Health, FXB 545
651 Huntington Avenue
Boston MA 02115
PHONE: (617) 432-0430
FAX: (617) 432-3163
E-mail: chen@sdac.harvard.edu

Clinical Trials Specialist

Jennifer L. Gardella, B.S.
Senior Clinical Trials Specialist
PACTG Operations Center
8757 Georgia Avenue
Silver Spring, MD 20910
PHONE: (301) 628-3341
FAX: (301) 628-3304
E-mail: jgardella@s-3.com

Protocol Data Manager

Janice Hodge, RN, B.S.
Data Manager
Frontier Science and Technology
Research Foundation
4033 Maple Road
Buffalo, NY 14226-1056
PHONE: (716) 834-0900 (ext. 269)
FAX: (716) 834-8675
E-mail: hodge.janice@fstrf.org

PENTA

Protocol **Trial** Management

Lynda Harper, M.Sc.
Trial Manager
MRC Clinical Trials Unit
HIV Division
222 Euston Rd.
London, NW1 2DA, United Kingdom
PHONE: 44-20-7670-4791
FAX: 44-20-7670-4814
E-mail: Lynda.harper@ctu.mrc.ac.uk

Laura Farrelly
Trial Manager
MRC Clinical Trials Unit
HIV Division
222 Euston Rd.
London, NW1 2DA, United Kingdom
PHONE: 44-20-7670-4789
FAX: 44-20-7670-4814
E-mail: Laura.farrelly@ctu.mrc.ac.uk

Protocol Data Manager

Cathy Taylor, Ph.D.
Data Manager
MRC Clinical Trials Unit
HIV Division
222 Euston Rd.
London, NW1 2DA, United Kingdom
PHONE: 44-20-7670-4825
FAX: 44-20-7670-4814
E-mail: cjt@ctu.mrc.ac.uk

PENPACT 1 PROTOCOL TEAM ROSTER

PACTG

Protocol Field Representative

MariPat Toye, R.N., M.S.
Study Coordinator/Data Manager
Baystate Medical Center/Peds UMASS
Main 3 -SHU-Dept of Pediatrics
759 Chestnut Street
Springfield MA 01199
PHONE: (413) 794-5399
FAX: (413) 794-3207
E-mail: maripat.toye@bhs.org

Protocol Pharmacist

Elaine Ferguson, R.Ph., M.S.
Pharmacist
NIH, NIAID, DAIDS, PAB
6700-B Rockledge Dr., Room 5239
MSC 7620
Bethesda, MD 20892-7620
PHONE: (301) 435-3742
FAX: (301) 402-1506
E-mail: eferguson@niaid.nih.gov

Protocol Neuropsychologist

Pim Brouwers, Ph.D.
Director, Clinical Neuroscience/Prof. of Pediatrics
Texas Children's Hospital
Baylor College of Medicine/Pediatrics
6621 Fannin Street (MC3-3320)
Houston, TX 77002
PHONE: (832) 822-4746
FAX: (832) 825-1503
E-mail: brouwers@bcm.tmc.edu

PENTA

Yacine Saidi, Ph.D.
Head Data Management
INSERM SC10, HIV Clinical Trial Centre
16 Avenue Paul Vaillant Couturier
Villejuif Cedex, 94807, France
PHONE: 33-14-559-5184
FAX: 33-14-559-5180
E-mail: y.saidi@vjf.inserm.fr

Other Core Investigators

Jean Pierre Aboulker, M.D., M.Sc.
Director
INSERM SC 10, HIV Clinical Trial Centre
16 Avenue Paul Vaillant Couturier
94807 Villejuif Cedex, France.
PHONE: 33 1 4559 5107
FAX: 33 1 4559 5180
E-mail: jp.aboulker@vjf.inserm.fr

Janet Darbyshire, OBE, FRCP, M.Sc.
Director
MRC Clinical Trials Unit
222 Euston Road
London
NW1 2DA, UK
PHONE: 44 20 7670 4701
FAX: 44 20 7670 4818
E-mail: Janet.Darbyshire@ctu.mrc.ac.uk

PENPACT 1 PROTOCOL TEAM ROSTER

PACTG

Protocol Immunologist

E. Richard Stiehm, M.D.
Professor, Department of Pediatrics
UCLA School of Medicine
22-404 MDCC
10833 Le Conte Avenue
Los Angeles, CA 90095-1752
PHONE: (310) 825-6481
FAX: (310) 206-5843
E-mail:
estiehm@pediatrics.medsch.ucla.edu

Protocol Virologists

Susan Fiscus, Ph.D.
Professor/Director of the Retrovirology Core Laboratory
University of North Carolina School of Medicine
Department of Microbiology & Immunology
709 Mary Ellen Jones Building
Box # CB#7140
Chapel Hill, NC 27599-7140
PHONE: (919) 966-6872
FAX: (919) 966-9873
E-mail: fiscussa@med.unc.edu

Stephen Spector, A., M.D.
University of California, San Diego
Department of Pediatrics
Division of Infectious Diseases
Stein Clinical Research Bldg., Room 430
9500 Gilman Dr., Mail Code 0672
La Jolla, CA 92093-0672
PHONE: (858) 534-7170
FAX: (858) 534-7411
E-mail: saspector@ucsd.edu

PENTA

Other Core Investigators (Cont.)

Gareth Tudor-Williams, M.D.
Senior Lecturer in Paediatric Infectious Diseases
Division of Paediatrics, **Obstetrics and Gynecology**
Faculty of Medicine Imperial College
St. Mary's **Campus**
Norfolk Place
LONDON W2 **1PG**, UK
PHONE: 44 **(0) 20 7594/3697**
FAX: 44 **(0)** 20 **3984**
E-mail: g.tudor-williams@imperial.ac.uk

The PENTA Executive Committee will appoint, as needed, any other PENTA scientists, e.g. virologists, immunologists, pharmacists, pharmacologists, to represent PENTA 9 in PENPACT 1. Furthermore, each PENTA Scientific Committee (e.g. Virology or Immunology Committees) may designate representatives from participating countries in PENTA 9 for any scientific discussions held by PENPACT 1.

The PENTA Medical Officers will act as Medical Safety Monitors for SAEs for PENTA sites.

PENPACT 1 PROTOCOL TEAM ROSTER

PACTG
Protocol Laboratory Technologist

Cheryl Jennings
Lab Manager, VQA
Rush Presbyterian St. Luke's Medical Center
Retrovirology Laboratory
Jelke Bldg., 1181
1750 West Harrison Street
Chicago IL 60612-3824
PHONE: (312) 942-3446
FAX: (312) 942-6787
E-mail: cjenning@rush.edu

Medical Safety Monitor (SAE)

Elizabeth Smith, M.D.
Medical Monitor
NIH, NIAID, DAIDS, TRP, PMB
Rm. 5105
6700-B Rockledge Dr., MSC 7624
Bethesda, MD, 20892-7624
PHONE: (301) 402-3226
Fax: (301) 402-3171
E-mail: bs161v@nih.gov

<u>SUMMARY OF CHANGES FOR PENPACT1 VERSION 3.0, DATED 06/28/05</u>

A PHASE II/III RANDOMIZED, OPEN-LABEL STUDY OF COMBINATION ANTIRETROVIRAL REGIMENS AND TREATMENT-SWITCHING STRATEGIES IN HIV-1-INFECTED ANTIRETROVIRAL NAIVE CHILDREN >30 DAYS AND <18 YEARS OF AGE

All changes in this version appear in boldface type. Editorial changes, including corrections of typographical errors and other changes required to update information that does not affect regulatory issues or patient consent may also be included.

Please note that the following items are not relevant to PENTA sites: 3, 9, 14a, 14d, 14f, 17, 18 and 19.

The main purposes of this amendment are to (a) clarify definitions of first-line therapy and second-line therapy, (b) clarify criteria for changes in or discontinuation of first or second line therapy, and (c) clarify criteria for switching from first to second line therapy. Related revisions appear in the Schema and in Sections 3.0, 4.4, 5.1-5.4, Appendix II,

Other changes are as follows:

1. **Information regarding regulatory requirements in Brazil has been added to the Forward.**
2. **The protocol team roster has been updated.**
3. **The RCC Safety Office should be contacted for any questions concerning expedited adverse event reporting.**
4. **Primary objective 2.11 was clarified to note that "failure" refers to <u>virologic</u> failure. This is also reflected in the Trial Summary.**
5. **Section 3.15 has been revised to note that prior exposure to NVP is exclusionary, and a new exclusion criterion was added as Section 3.21: Infants or maternal peripartum NVP exposure for prevention of mother-to-child HIV transmission. This revision is also reflected in the Trial Summary.**
6. **Section 3.24 was revised to read "[i.e. impossibility to identify <u>both</u> a 2 NRTI + PI regimen <u>and</u> a 2 NRTI + NNRTI regimen that the child can take]".**
7. **The list of allowable antiretrovirals in Section 4.4 has been updated. New additions include Emtriva®, Truvada®, and Viread®.**
8. **The criteria for changing from first-line to second-line therapy in Section 5.2 were revised. If poor adherence is suspected as a possible reason for failure to achieve the randomized HIV-1 RNA value by Week 24, sites should try to improve adherence within a 5 week window period, and subsequently obtain the confirmatory HIV-1 RNA value.**
9. **Version 3.0 of PACTG 390 will follow the new DAIDS Expedited Adverse Event reporting requirements. Specific requirements have been added to Section 6.1.**

10. Section 6.1 has been revised to note that PENTA sites must also follow the EU Clinical Trials Directive 2001/20/EC (20) in reporting SAEs.
11. Grade 3 and 4 toxicity management guidelines have been revised; refer to Sections 6.223 and 6.225.
12. Section 10.2 has been updated to include a new requirement for sites to have a plan that detects and addresses any change in guardianship occurring in pediatric subjects and determines when a study subject must have a consent process which involves a legally authorized representative (LAR) other than a family member with guardianship.
13. Throughout the protocol, minor revisions have been made to allow for the participation of non-U.S. PACTG sites.
14. Revisions to Appendix I include:
   a. The neurological examination is only required for subjects enrolled at domestic <u>and international</u> PACTG sites at indicated time points. Additionally, for those subjects who are off study treatment but on study follow-up, the neurological examination only needs to be completed whenever there is a neurological problem, not every 12 weeks.
   b. For those subjects who are off study treatment but on study follow-up, blood for plasma and PBMC storage will only be collected every 24 weeks.
   c. A Week 204 plasma and PBMC specimen has been added.
   d. For subjects enrolled at PACTG sites, RNA PCRs will be run locally at a DAIDS VQA certified laboratory. Only aliquots from specimens collected at Screening, Entry, Week 24, time of therapy switch, Week 324, and end of study will be batched and shipped to UNC. Processing and shipping instructions have been revised accordingly in Appendix VII.
   e. For PENTA sites, processing instructions for HIV RNA PCR specimens and stored specimens have been revised.
   f. The neuropsychological assessment is required for subjects on first-line therapy as well as for subjects on second-line therapy; the testing schedule is based on enrollment date and does not change if therapy is switched. A 30 day window has been added for completion of the assessment at Entry. (Refer to Appendix I footnotes for specific details.)
15. Revisions to Appendix IX include:
   a. New ABC dosing information.
   b. Addition of ABC/3TC preparation.
   c. Addition of Emtriva®, Truvada®, and Viread®.
   d. Addition of FDA-approved generic ddI.
   e. Change in ZDV dosing recommendations.
16. In Appendix XIV, the requirement that subjects be weighed barefoot wearing only a gown was changed to "wearing minimal clothing and no shoes".
17. The PACTG 390 Patient Information Handout (formerly Appendix XVII) has been removed; this document is available on the PACTG web site and will be maintained separate from the protocol document.
18. Revisions to the DAIDS Sample Informed Consent (Appendix XVII) include:

a. Under *"What Do I Have To Do If I Am In This Study"*, the list of antiretrovirals has been updated to match that in Section 4.4.
b. Under *"What Do I Have To Do If I Am In This Study"*, a segment was added regarding the neuropsychological testing.
c. Under *"Why Would The Doctor Take Me Off This Study Early?*, pregnancy was added as a reason for study drug discontinuation.
d. Risks related to study drugs, switching drug regimens and resistance have been updated.
e. Under *"What About Confidentiality"*, the second sentence has been revised to read *"The study teams from both organizations wrote one study called PENPACT 1, which includes actually two different studies, PACTG 390 (the one you/your child/your baby are/is enrolling) at U.S. and international sites connected with the PACTG, and PENTA 9 in Europe."*
f. The *"What About Stored Samples"* section has been updated according to current DAIDS requirements.

19. The fact sheet and template consent form for NICHD sites have been added as Appendix XVIII.

SUMMARY OF CHANGES FOR PENPACT1 VERSION 3.1, DATED 07/11/07

All changes appear in green font and can be found on pages 4 to 6 (Protocol Team Roster) and Appendix IX (in bold type).

APPENDIX IX Allowed Antiretroviral Therapy Background Information, Suggested Dosing and Major Toxicities

This appendix establishes the allowed antiretrovirals that could be prescribed for PENPACT 1 children, and was written to assist clinicians in selecting each child's regimen by using FDA/EMEA approved dosing or the PENPACT1 dosing recommendations. New drugs that have been developed since the protocol was written have been added to the appendix. Some of the dosing included in this section is not FDA/EMEA approved but has been used in clinical research under investigational new drugs, with adequate efficacy and safety observed, and is commonly used in the clinical setting. Some drugs licensed for adults have also been included since some of the young people in the trial have reached the age of 18.

New drugs added to Appendix IX are as follows:

ATAZANAVIR (ATV, REYATAZ®)
DARUNAVIR (DRV, PREZISTA®)
EFAVIRENZ/EMTRICITABINE/TENOFOVIR DISOPROXIL FUMARATE (ATRIPLA®)
FOSAMPRENAVIR CALCIUM (FPV, LEXIVA®, TELZIR®)
TIPRANAVIR (TPV, APTIVUS®)

Drugs for which the existing information has been modified are:

EFAVIRENZ (EFV, SUSTIVA®)

EMTRICITABINE (FTC, EMTRIVA®)

INDINAVIR (IDV, CRIXIVAN®)

LOPINAVIR/RITONAVIR (LPV/r, Kaletra®)

NELFINAVIR (NFV, VIRACEPT®)

RITONAVIR (RTV, NORVIR®)

SAQUINAVIR (SQV, INVIRASE®, FORTOVASE®)

TENOFOVIR DISOPROXIL FUMURATE (TDF, VIREAD®)

ZIDOVUDINE (ZDV, RETROVIR®, FDA-APPROVED GENERIC ZIDOVUDINE)

Team Roster: Contact details also amended (page 4, 5 and 6).

TABLE OF CONTENTS

Page

FOREWORD 2
SUMMARY OF CHANGES FOR PENPACT1 VERSION 3.0, dated 06/28/05 10
SUMMARY OF CHANGES FOR PENPACT1 VERSION 3.1, dated 07/11/07 12
LIST OF APPENDICES 16
TRIAL SUMMARY 18
1.0 BACKGROUND AND RATIONALE 21
1.1 General Background 21
1.2 Study Rationale 22
2.0 STUDY OBJECTIVES 23
2.1 Primary Objectives 23
2.2 Secondary Objectives 24
3.0 STUDY DESIGN 24
3.1 Inclusion Criteria 25
3.2 Exclusion Criteria 26
3.3 Co-Enrollment Guidelines 27
4.0 Trial Management 27
4.1 Information and Informed Consent Forms 27
4.2 Eligibility 27
4.3 Randomization 28
4.4 Study Treatment for **First-Line** Therapy: 28
4.5 Study Treatment for Second-Line Therapy 29
5.0 PATIENT MANAGEMENT (HIV-1 RNA CRITERIA) 30
5.1 **Changes to First-Line** Therapy 30
5.2 **Criteria for Changing from First-Line Therapy to Second-Line Therapy** 30
5.3 **Criteria for Discontinuing First-Line Therapy** 32
5.4 **Criteria for Discontinuing** Second-Line Therapy: 32
6.0 ADVERSE EVENT REPORTING AND MANAGEMENT 33
6.1 Serious Adverse Experience (SAE)**/Expedited Adverse Event** Reporting 33
6.2 Criteria for Management of Adverse Events/Toxicity and Dose Modification 34
7.0 COORDINATION OF THE TRIAL, DATA COLLECTION, AND MONITORING 37
7.1 Trial Coordination 38
7.2 Regional Monitoring 38
8.0 Drug Accountability and Adherence 39
8.1 Accountability 39
8.2 Adherence 39
9.0 STATISTICAL CONSIDERATIONS 40
9.1 General Design Issues 40
9.2 Primary Outcome Measure 40
9.3 Secondary Outcome Measures 40
9.4 Randomization and Stratification 41
9.5 Sample Size and Accrual 41

TABLE OF CONTENTS

9.6 Monitoring 42
9.7 Analysis........ 43
10.0 HUMAN SUBJECTS 45
10.1 Declaration of Helsinki........ 45
10.2 PACTG Institutional Review Board (IRB) Review and Informed Consent........ 45
10.3 PENTA Ethics and Regulatory Approval Committee 46
10.4 Confidentiality 46
10.5 PACTG Study Discontinuation: 46
10.6 PENTA Study Discontinuation:........ 46
11.0 PUBLICATION OF RESEARCH FINDINGS 46
12.0 SAMPLES CLARIFICATION........ 47
13.0 BIOHAZARD CONTAINMENT........ 47
14.0 PENTA CENTERS LIABILITY/INSURANCE 47
15.0 REFERENCES 48
Background........ 5
1.1 Introduction........ 5
1.2 Rationale 6
Objectives 6
2.1 Primary objectives 6
2.2 Secondary objectives 6
Sub-study Participants 6
Assessments and Procedures........ 8
4.1 Flow chart/ Schedule for follow-up 8
4.2 Key measures........ 9
4.3 Fasting........ 9
Quality Assurance and Quality Control........ 10
Regulatory and Ethics Approval........ 10
Finance........ 10
Statistical analyses 10
References........ 11

LIST OF APPENDICES

I. SCHEDULE OF EVENTS FOR PACTG AND PENTA FOR BOTH, INITIAL AND SECOND-LINE THERAPIES

II. GUIDELINES FOR SWITCHING THERAPY FOR PACTG AND PENTA

III. PENTA 9--PENPACT 1-B SUB-STUDY ON LIPODYSTROPHY SYNDROME AND METABOLIC ABNORMALITIES

IV. DIVISION OF AIDS TOXICITY TABLE FOR GRADING SEVERITY OF PEDIATRIC (≤3 MONTHS OF AGE) ADVERSE EXPERIENCES. APRIL-1994 (TO BE USED BY PACTG AND PENTA)

V. DIVISION OF AIDS TOXICITY TABLE FOR GRADING SEVERITY OF PEDIATRIC (>3 MONTHS OF AGE) ADVERSE EXPERIENCES. APRIL-1994 (TO BE USED BY PACTG AND PENTA)

VI. SUPPLEMENTAL TOXICITY TABLE FOR GRADING SEVERITY OF ADULT AND PEDIATRIC CUTANEOUS/SKIN RASH/DERMATITIS ADVERSE EXPERIENCES (TO BE USED BY PACTG AND PENTA)

VII. DETERMINATION OF PLASMA HIV-1 RNA - PACTG SITES

VIII. DETERMINATION OF PLASMA HIV-1 RNA - PENTA SITES

IX. ALLOWED ANTIRETROVIRAL THERAPY BACKGROUND INFORMATION, SUGGESTED DOSING AND MAJOR TOXICITIES FOR PACTG AND PENTA

X. MANAGEMENT OF SPECIFIC ADVERSE EVENTS FOR PACTG AND PENTA

XI. MEDICATIONS WITH KNOWN SERIOUS INTERACTIONS (INFORMATION TO BE CONSIDERED BY PACTG AND PENTA)

XII. CDC CLASSIFICATION. PEDIATRIC HIV CLASSIFICATION. CDC CLASSIFICATION SYSTEM FOR HIV-INFECTION IN CHILDREN LESS THAN 13 YEARS OF AGE (MMWR 1994; 43 [RR-12];1-10) (INFORMATION TO BE USED BY PACTG AND PENTA FOR ALL CHILDREN IN THE STUDY REGARDLESS OF THEIR AGE)

XIII. NEURODEVELOPMENTAL ASSESSMENTS (ONLY FOR U.S. PACTG DOMESTIC SITES)

XIV. MEASUREMENT OF HEIGHT, LENGTH, WEIGHT, AND HEAD CIRCUMFERENCE (PROCEDURES TO BE FOLLOWED BY PACTG AND PENTA)

XV. TANNER SCALES (PROCEDURES TO BE FOLLOWED BY PACTG AND PENTA)

XVI. SAMPLE INFORMED CONSENT FORM FOR PENTA SITES

XVII. SAMPLE INFORMED CONSENT FORM FOR PACTG SITES

XVIII. **FACT SHEET AND TEMPLATE CONSENT FORM (NICHD)**

TRIAL SUMMARY

PENPACT 1
(PENTA 9/PACTG 390)
A PHASE II/III RANDOMIZED, OPEN-LABEL STUDY OF COMBINATION ANTIRETROVIRAL REGIMENS AND TREATMENT-SWITCHING STRATEGIES IN **HIV-1-INFECTED** ANTIRETROVIRAL NAIVE CHILDREN **>**30 DAYS AND **<**18 YEARS OF AGE

PRIMARY OBJECTIVES

- To compare the combination of 2 NRTIs plus a protease inhibitor (PI) versus 2 NRTIs plus a non-nucleoside reverse transcriptase inhibitor (NNRTI) as initial therapy, followed by second-line therapy if **virologic** failure occurs, in terms of their effects on a long-term virologic endpoint.

- To compare two different viral load criteria for switching from first-line to second-line therapy.

SECONDARY OBJECTIVES

- To evaluate and compare the safety and tolerability of each drug combination (including first- and second-line therapies).

- To compare the long-term clinical and immunologic outcomes (by the initial randomization).

- To compare the proportions of children who have undergone one regimen switch or reached study end-point (by the initial randomization).

- To compare time from randomization to virologic failure (RNA $\geq$400 copies/mL at or after Week 24) of the first-line therapy analyzed by initial randomization to either protease inhibitor (PI) or NNRTI containing regimens.

- To compare time from randomization to virologic failure of the second line therapy (RNA $\geq$30,000 copies/mL) analyzed by the initial randomization.

- To compare the proportion of children with plasma HIV-1 RNA <400 copies/mL at 4 years (by the initial randomization).

- To describe resistance patterns at four years (by the initial randomization).

VIROLOGIC ENDPOINT DEFINITION

The virologic endpoint is change in HIV-1 RNA viral load between baseline and four years post randomization. It is likely that by four years post-randomization, nearly all children will have

switched from first to second-line therapy.

STUDY DESIGN

This is an international multicenter Phase II/III, randomized, open label, and factorial (2x2) trial.

SAMPLE SIZE

256 children, approximately 50% from PACTG sites and 50% from PENTA sites.

POPULATION

HIV-1 infected **children who are** antiretroviral naïve or who have received less than 56 days of antiretroviral drugs used on consecutive days after birth to prevent mother-to-infant **HIV** transmission. **Prior exposure to NVP, including for prevention of mother-to-child transmission, is exclusionary.**

RANDOMIZATION AND STRATIFICATION

Children will be randomized to **one of** four groups **and** stratified by age (<3 years versus ≥3 years), origin (PACTG site or PENTA site), and exposure versus no exposure to antiretroviral therapy perinatally.

FIRST-LINE THERAPY

All study medications will be given by prescription (the child, child's parent**(s)**/legally **authorized** representative **(LAR)**, the child's health insurance, and/or, in Europe, the healthcare provider, are responsible for purchasing the study medications).

Children are randomized to one of four groups that are defined by the initial therapy to be given to a child as well as the virologic criterion for switching from first-line therapy (defined below) to second-line therapy:

- Group 1(A): **Initial therapy is** 2 NRTIs + PI
  (switch to second-line **therapy** when HIV-1 RNA is ≥1,000 copies/mL)
- Group 1(B): **Initial therapy is** 2 NRTIs + PI
  (switch to second-line **therapy** when HIV-1 RNA is ≥ 30,000 copies/mL)
- Group 2(A): **Initial therapy is** 2 NRTIs + NNRTI
  (switch to second-line **therapy** when HIV-1 RNA is ≥1,000 copies/mL)
- Group 2(B): **Initial therapy is** 2 NRTIs + NNRTI
  (switch to second-line **therapy** when HIV-1 RNA is ≥30,000 copies/mL)

First-line therapy includes the initial therapy to which a child is randomized as well as any

antiretroviral therapies to which the child changes due to non-virologic reasons (e.g. toxicity, intolerability, request of child or child's parent**(s)/LAR**, etc.) prior to reaching the HIV-1 RNA switch criterion (≥1,000 copies/mL or ≥30,000 copies/mL, depending on the initial randomization). **Whenever possible, changes within first-line therapy should involve substitutions of one or more drugs in the initial therapy by drugs from the same class or classes.**

It is important to emphasize that the protocol will allow low doses of ritonavir as a boosting agent, creating drug combinations that will be counted as a single PI.

SECOND-LINE THERAPY

Second-line therapy will be initiated when the HIV-1 RNA switch criterion (≥1,000 copies/mL or ≥30,000 copies/mL, depending on the initial randomization) is reached. The following suggested drug regimens will be strongly encouraged as a second-line therapy for all children failing **first-line therapy** (when HIV-1 RNA is ≥1,000 copies/mL or when HIV-1 RNA is ≥30,000 copies/mL, depending on the initial randomization)**, particularly those children who have remained on their initial (randomized) therapy**:

- For PI-containing Groups **1(A) and 1(B)**: two new NRTIs and an NNRTI
- For NNRTI-containing Groups **2(A) and 2(B)**: two new NRTIs and a PI

However, these regimens do not constitute the only options. Current clinical care guidelines will prevail over protocol **guidelines**, **(e.g.** a fourth drug as part of second-line therapy).

The protocol will allow low doses of ritonavir as a boosting agent, creating drug combinations that will be counted as a single PI.

PENPACT 1 FOLLOW-UP PERIOD

All children will **be followed** until the last child enrolled has reached 204 weeks on **study** treatment from his/her original randomization. This last patient could be from a PACTG or PENTA site.

After Week 204 of treatment until the study ends, children will continue on study follow-up with regular study visits every 12 weeks as described in Appendix 1. This follow-up period will be used to address long-term time-to-event secondary objectives of the study.

PENPACT 1 COMPARISON FOR ALL CHILDREN IN THE STUDY

- PI versus NNRTI as part of the initial therapy
- Switching to second-line therapy at an HIV-1 RNA level of ≥1,000 copies/mL versus switching at HIV-1 RNA level of ≥30,000 copies/mL

1.0 BACKGROUND AND RATIONALE

1.1 General Background

Studies of viral dynamics, and the knowledge of the high mutation rate of the HIV virus, coupled with clinical experience, have all confirmed that combination antiretroviral therapy is needed to achieve inhibition of viral replication. Several published studies in children have demonstrated the advantages of dual therapy compared with monotherapy. **However,** it has also become clear that dual therapies achieve long-term viral suppression in only a very small proportion of individuals (1-5).

There are no data defining a particular highly active antiretroviral therapy (HAART) strategy as being optimal first-line therapy for children or adults. Thus, numerous adaptations exist that fall broadly into three HAART therapy groups:

(1) Two nucleoside reverse transcriptase inhibitors (NRTIs) + one protease inhibitor (PI)

(2) Two NRTIs + one non-nucleoside reverse transcriptase inhibitor (NNRTI)

(3) Three NRTIs

Each strategy has strengths and weaknesses. The first therapy group was the first to achieve complete and sustained viral inhibition in adults, and initially was viewed as the most potent HAART cocktail (6). However, there is increasing recognition of the longer term adverse effects of PIs on lipid metabolism and body fat distribution in a significant proportion of individuals (7). The PIs as a class are not easily formulated in solution and taste extremely bitter. Thus, for children who cannot swallow capsules, these drugs can be difficult for parents and caregivers to administer long-term.

The second therapy group has been perceived until recently as less potent, and most drugs in the NNRTI class are associated with skin rash in up to 20% of individuals. However, for children particularly, provided they do not experience an early drug allergy, palatable formulations exist that can be given once daily and that appear to be very well tolerated long-term. Initial data from adult trials have suggested comparable reductions in viral load in adults randomized to triple regimens including either an NNRTI or a PI (8,9).

The third group of therapy, because it consists of NRTIs only, has the distinct advantage of sparing the two other classes of drugs, allowing room for maneuver in subsequent treatment options. There is more limited long-term experience with this HAART option compared to the other two groups of therapy. **However,** equivalent efficacy is being reported (8,10). The NRTIs are available as palatable formulations for children, and many have been in use for longer than either NNRTIs or PIs. Thus, their individual side effect profiles are more familiar. Cross-class resistance may mean that a child failing a regimen containing only NRTIs will have little chance of benefit from any other drugs of this class. Severe hypersensitivity reactions to abacavir have been reported in up to 3%

of individuals. In a recently reported study of abacavir in treatment-experienced children, two of 205 children were withdrawn because of suspected hypersensitivity (11).

1.2 Study Rationale

PENPACT 1 is designed to evaluate the long-term efficacy, as measured by HIV-1 RNA over four years, of different initial HAART combinations in children and different strategies for switching therapy.

It is recognized that there are insufficient numbers of antiretroviral-naïve HIV-infected children **at participating study sites** to investigate all three of the HAART groups outlined above. Therefore, PENPACT 1's primary objective is the comparison of the combination of two NRTIs + one PI versus two NRTIs + one NNRTI as initial therapy followed by second-line therapy if failure occurs as defined by a long-term virologic endpoint.

It is not known which of the above combinations will have the most sustained impact on reducing viral load. Adult studies have shown similar or even improved efficacy with initial regimens utilizing two NRTIs and one NNRTI versus two NRTIs and one PI. A study with antiretroviral naïve adults treated with efavirenz plus zidovudine and lamivudine showed an improvement in virologic outcome, compared to those treated with indinavir plus zidovudine and lamivudine (14). The baseline viral load did not affect the virologic outcome. Another study comparing triple NRTI therapy to two NRTIs plus one PI showed decreased virologic efficacy in adults whose baseline plasma RNA levels were >100,000 copies/mL (15). As children generally have higher baseline plasma RNA levels than adults, it is possible that a regimen with two NRTIs and one NNRTI may show less efficacy particularly in younger children. However, NNRTI medications are generally better tolerated than PI medications by both adults and children. The potential decrease in potency may be offset by an increase in tolerability and thus adherence.

Additionally, it is unknown if the order in which antiretroviral agents are utilized will have an effect on long-term virologic efficacy. For example, there are data to suggest that d4T has decreased efficacy when a patient has been previously treated with zidovudine (16). The goal of antiretroviral therapy in children is to prolong clinical and immunologic health until adulthood. Therefore, the antiretroviral medications chosen for the initial regimen should be selected with a long-term plan in mind. PENPACT 1 will investigate if treatment with one NNRTI versus one PI in the initial therapy affects the subsequent potency of the second-line therapy. The first objective of PENPACT 1 therefore, is to compare the long-term efficacy of a combination of two NRTIs plus one PI versus two NRTIs plus one NNRTI as initial therapy, followed by second-line therapy if **virologic** failure occurs.

The optimal criteria for switching therapy have yet to be defined. Continued viral replication while under antiretroviral drug pressure results in the accumulation of mutations associated with antiretroviral drug resistance. Therefore, there is a logical

argument in favor of strict control of viral replication. Strict control dictates that as soon as virus becomes detectable in the circulation (or if plasma viral load fails to reach undetectable limits), then a switch should be made to a different, and probably more intensive, regimen. The problem with this approach, given the limitations of drugs available to children, is that this policy may rapidly exhaust all available options for therapy. As children with plasma HIV-1 RNA levels less than 100,000 copies/mL are at relatively low risk for disease progression (17, 18, 19)**, an** alternative approach, which may be equally valid over a long period of follow-up, would be to try to maximize the benefit of each regimen and switch only when the viral load is consistently above a higher threshold. This strategy may preserve options longer. Although with this strategy, the virus could continue to accumulate resistance mutations, there is some evidence that virus replicating in the presence of HAART may be less pathogenic *in-vivo* (12,13).

Therefore, the second primary objective of PENPACT 1 is to investigate the long-term efficacy of utilizing a strategy of "tight" virologic control (switching **first-line** therapy when viral load rises above 1,000 copies/mL) versus "looser" virologic control (switching therapy when the viral load rises above 30,000 copies/mL). The long-term nature of this study should clarify whether early switching improves immunologic and virologic outcomes or results in a more rapid exhaustion of treatment options.

Unfortunately, there are few clinical data on which to base the two threshold RNA values selected by the PENPACT 1 Team to mandate therapeutic changes. However, after much deliberation, the lower threshold was chosen to be 1,000 copies/mL and the higher threshold **was chosen to be** 30,000 copies/mL. The lower of the two thresholds, 1,000 copies/mL (3**.0** logs), represents clear escape from complete virologic suppression. However, it is still at a limited level of replication. Thus, a second-line therapy may have a higher likelihood of achieving complete suppression. The upper threshold, 30,000 copies/mL (4.5 logs), was chosen because it is distant enough from 1,000 copies/mL (3.0 logs) to be considered a clinically and statistically significant change, yet at this RNA level, the risk of disease progression remains relatively low (19).

2.0 STUDY OBJECTIVES

2.1 Primary Objectives

2.11 To compare the combination of 2 NRTIs plus a protease inhibitor (PI) versus 2 NRTIs plus a non-nucleoside reverse transcriptase inhibitor (NNRTI) as initial therapy, followed by second-line therapy if **virologic** failure occurs, in terms of their effects on a long-term virologic endpoint.

2.12 To compare two different viral load criteria for switching from first-line to second-line therapy.

2.2 Secondary Objectives

2.21 To evaluate and compare the safety and tolerability of each drug/combination (including first- and second-line therapies).

2.22 To compare the long-term clinical and immunologic outcomes (by the initial randomization).

2.23 To compare the proportions of children who have undergone one regimen switch or reached study end-point (by the initial randomization).

2.24 To compare time from randomization to virologic failure (RNA >400 copies/mL at or after Week 24) of the first-line therapy analyzed by initial randomization to either protease inhibitor (PI) or NNRTI containing regimens.

2.25 To compare time from randomization to virologic failure of the second-line therapy (RNA >30,000 copies/mL) analyzed by the initial randomization.

2.26 To compare the proportion of children with plasma HIV-1 RNA <400 copies/mL at 4 years (by the initial randomization).

2.27 To describe resistance patterns at four years (by the initial randomization).

3.0 STUDY DESIGN

This is an international, multi-center, Phase II/III, randomized, open-label, factorial (2x2) trial. This study will enroll 256 HIV-1 infected children who are antiretroviral naïve or who have received less than 56 days of antiretroviral drugs used on consecutive days after birth to prevent mother-to-infant **HIV** transmission. Approximately 50 % of the children will be from PACTG sites and 50 % **will be** from PENTA sites.

Children will be randomized to one of four groups stratified by age (<3 years versus ≥3 years), origin (PACTG site or PENTA site), and exposure versus no exposure to antiretroviral therapy perinatally. **The groups** are defined by the initial therapy to be given to a child as well as the virologic criterion for switching from first-line therapy (defined below) to second-line therapy.

- Group 1(A): **Initial therapy is** 2 NRTIs + PI
  (switch to second-line **therapy** when HIV-1 RNA is ≥1,000 copies/mL)
- Group 1(B): **Initial therapy is** 2 NRTIs + PI
  (switch to second-line **therapy** when HIV-1 RNA is ≥30,000 copies/mL)
- Group 2(A): **Initial therapy is** 2 NRTIs + NNRTI
  (switch to second-line **therapy** when HIV-1 RNA is ≥1,000 copies/mL)
- Group 2(B): **Initial therapy is** 2 NRTIs + NNRTI
  (switch to second-line **therapy** when HIV-1 RNA is ≥30,000 copies/mL)

First-line therapy includes the initial therapy to which a child is randomized as well as any antiretroviral therapies to which the child changes due to non-virologic reasons (e.g. toxicity, intolerability, **request of child or child's parent(s)/legally authorized representative (LAR), etc.**) prior to reaching the HIV-1 RNA switch criterion (≥1,000 copies/mL or ≥30,000 copies/mL, depending on the initial randomization). Whenever possible, changes **within** first-line therapy should involve substitutions of one or more drugs in the initial therapy by drugs from the same class or classes.

The following suggested drug regimens will be strongly encouraged as a second-line therapy for all children failing first-line therapy (when HIV-1 RNA is ≥1,000 copies/mL or when HIV-1 RNA is ≥ 30,000 copies/mL, depending on the initial randomization), particularly those children who have remained on their initial (randomized) therapy:

- For PI-containing Groups 1(A) and 1(B):
  The second-line therapy will be two new NRTIs + NNRTI
- For-NNRTI containing Groups 2(A) and 2(B):
  The second-line therapy will be two new NRTIs + PI

All children will be followed **until the last child enrolled has reached 204 weeks on study treatment from his/her original randomization.**

There will be two pair wise comparisons for all children: PI-**containing** versus NNRTI-**containing therapy as initial therapy**, and switching therapy at an HIV-1 RNA level of ≥1,000 copies/mL versus ≥30,000 copies/mL.

3.1 Inclusion Criteria

3.11 Children >30 days and <18 years of age. Children may be randomized up to the day before their 18th birthday.

3.12 A confirmed diagnosis of HIV infection **according to** the current definition of the PACTG Virology Core Laboratory Committee. The current PACTG definition (April **17,** 2000)[3] requires two separate peripheral blood specimens from different days, and each specimen must be positive. The two positive results may be obtained in any combination of the following:

- at any age: HIV culture, HIV-DNA PCR, or plasma HIV RNA value ≥10,000 copies/mL
- age >4 weeks: neutralizable HIV p24 antigen (regular or ICD)
- age >18 months: licensed ELISA with confirmatory Western Blot

PENPACT 1 will follow the above definition for all subjects at PACTG and PENTA sites. The PACTG Virology Core Laboratory Committee may update the above definition at any time. Thus, PENPACT 1 will follow the current definition for both

[3] Since this is an international collaborative study of two different organizations, the restriction in the definition that one of the tests must be done in an ACTG-certified laboratory does not apply, even for children enrolling into PACTG 390.

PACTG and PENTA sites.

However, the PENTA Executive Committee has the right to question any of the possible changes/updates before their implementation in PENPACT 1 at PACTG and PENTA sites. **If this occurs,** PENPACT 1 will continue **to accrue** at both PACTG and PENTA **sites**, **using** the latest accepted definition by the PENPACT 1 Team until a new specific definition is adopted for all PACTG and PENTA sites.

3.13 Female subjects who are sexually active and able to become pregnant must **agree** to use the approved birth control methods for the assigned drug regimen under PENPACT 1. In most cases, drug regimens mandate the use **of** two methods of birth control. In these instances, hormonal birth control alone would not be considered adequate or effective. A medically accepted barrier method of contraception (e.g., condom) must also be used during the study. The interaction between study drugs and hormonal birth control has not been studied.

3.14 Parent/legally **authorized** representative and child, where appropriate, must be able to provide written informed consent, and assent.

3.15 Antiretroviral naïve (or have received less than 56 consecutive days after birth of antiviral drugs used to prevent mother-to-infant transmission) infants, children, and adolescents.

NOTE: Prior exposure to NVP is exclusionary; refer to Section 3.21.

3.2 <u>Exclusion Criteria</u>

3.21 **Infant or maternal peripartum NVP exposure for prevention of mother-to-child HIV transmission.**

3.22 Current Grade 3 or 4 clinical or laboratory toxicity as defined by age appropriate toxicity tables **in** Appendices IV and V (Grade 3 and 4 thrombocytopenia will be allowed only if it is of immunological origin).

3.23 Active opportunistic infection and/or serious bacterial infection at the time of study entry. (Children may be enrolled after the acute phase).

3.24 History of clinical pancreatitis, peripheral neuropathy, or other clinical, hematologic, hepatic, or renal contraindications to receiving the trial therapies (i.e. impossibility to identify **both** a 2 NRTI + PI regimen **and** a 2 NRTI + NNRTI regimen that the child can take).

3.25 Current treatment with any medication known to be contraindicated with any of the drugs to be prescribed for **the** patient's **initial therapy** (one of the NNRTIs or the selected PI; **refer to** Appendix **IX**).

3.26 Receipt of any cytotoxic therapy for malignancy.

3.27 Pregnancy or breastfeeding.

3.3 Co-Enrollment Guidelines

Children from PACTG sites may co-enroll in PACTG opportunistic infection, pharmacokinetics, or quality of life protocols, **in which** the study drugs offered are not contraindicated by PENPACT 1.

Co-enrollments require the assent of the Protocol Chairs of PENPACT 1 and the **Chairs of the** protocol(s) in which the child is co-enrolling. Co-enrollment in PACTG 219 is encouraged.

Co-enrollment in PENPACT 1-B Sub-Study will be only for PENTA children as part of **the** PENTA 9 study (see Appendix III). PACTG 390 children will not participate in this sub-study.

4.0 TRIAL MANAGEMENT

4.1 Information and Informed Consent Forms

Separate sample informed consent forms will be designed for PACTG sites and PENTA sites. These sample consent forms will comply with all the applicable regulations in each country. All parents/guardians and children, where appropriate, will be given information about the intent and rationale of the study (Appendices XVI and XVII).

Written informed consent must be obtained from all participants **and/or their** parent(s)/guardian(s) (including **LAR**). Assent **must be obtained** from children, if appropriate, after explanation of the aims, methods, benefits, and potential hazards of the trial, and before any trial specific procedures are performed.

It must be made completely and unambiguously clear to parents that they are free to refuse to allow their children to participate in the trial. Moreover, that they are free to withdraw their consent at any time and for any reason, without incurring any penalty or affecting the treatment of their children.

For both PACTG and PENTA, signed informed consent forms must be kept by the **site** investigators and documented in the case report forms. A copy of the consent form will be given to the parent**(s)** or **LAR** (and child, **if** appropriate).

4.2 Eligibility

Potentially eligible patients should be seen for a screening visit (Week -2) within the two weeks before trial entry (Week 0). Eligibility should be confirmed and clinical and laboratory assessments undertaken (see Appendix I).

4.3 Randomization

Randomization will be performed through the PACTG for all **PACTG**-funded sites. For PENTA sites, randomization will be done by the Medical Research Council (MRC) Clinical Trials Unit (London) or the INSERM Trials Center (Paris), after the Trials Center has received the pretrial assessment form and has checked the child's eligibility.

Eligible children will be randomized at Week 0, according to a computer-generated randomization list. The randomization code will be held by a statistician and a systems analyst at PACTG and at each European Trials Center.

4.4 Study Treatment for **First-Line** Therapy:

Children will be randomized to one of four groups that are defined by the initial therapy to be given to a child as well as the virologic criterion for switching from first-line therapy (defined below) to second-line therapy:

- Group 1(A): **Initial therapy is** 2 NRTIs + PI
  (switch to second-line **therapy** when HIV-1 RNA is ≥1,000 copies/mL)
- Group 1(B): **Initial therapy is** 2 NRTIs + PI
  (switch to second-line **therapy** when HIV-1 RNA is ≥30,000 copies/mL)
- Group 2(A): **Initial therapy is** 2 NRTIs + NNRTI
  (switch to second-line **therapy** when HIV-1 RNA is ≥1,000 copies/mL)
- Group 2(B): **Initial therapy is** 2 NRTIs + NNRTI
  (switch to second-line **therapy** when HIV-1 RNA is ≥30,000 copies/mL)

First-line therapy includes the initial therapy to which a child is randomized as well as any antiretroviral therapies to which the child changes due to non-virologic reasons (e.g. toxicity, intolerability, request of child or child's parent(s) or LAR, etc.) prior to reaching the HIV-1 RNA switch criterion (≥1,000 copies/mL or ≥30,000 copies/mL, depending on the initial randomization). Whenever possible, changes within first-line therapy should involve substitutions of one or more drugs in the initial therapy by drugs from the same class or classes.

It is important to emphasize that the protocol will allow low doses of ritonavir as a boosting agent, creating drug combinations that will be counted as a single PI.

Below is **a** list of **antiretrovirals allowed in PENPACT 1. All antiretrovirals will be obtained** by prescription. The child, child's parent**(s)/LAR**, the child's health insurance, and/or, in Europe, the healthcare provider, are responsible for purchasing the study medications.

NRTIs:

- Abacavir **sulfate**: ABC, Ziagen®
- Didanosine: ddI, Videx®, **Videx®EC, FDA-approved generic ddI**
- **Emtricitabine: FTC, Emtriva®**

- **Emtricitabine/Tenofovir disoproxil fumarate: FTC/TDF, Truvada®**
- Lamivudine: 3TC, Epivir®
- Lamivudine/Zidovudine: **3TC/ZDV,** Combivir®
- Stavudine: d4T, Zerit®
- **Tenofovir disoproxil fumarate: TDF, Viread®**
- Zalcitabine: ddC, Hivid®
- Zidovudine: AZT, **ZDV**, Retrovir®

NNRTIs:

- Efavirenz: **EFV,** Sustiva®
- Nevirapine: **NVP,** Viramune®

PIs:

- Amprenavir: **APV,** Agenerase®
- Indinavir **sulfate**: **IDV,** Crixivan®
- Lopinavir/Ritonavir: **LPV/r,** Kaletra®
- Nelfinavir **mesylate**: **NFV,** Viracept®
- Saquinavir: **SQV,** Fortovase®; Invirase®
- Ritonavir: **RTV**; Norvir®

Appendix IX serves as a treatment guideline for clinicians, and gives background and dosing information for all the allowed medications, doses, and regimens. All the information in this appendix is to be used as a guideline for prescribing the child's regimen. However, complete and detailed prescribing and toxicity information, on all antiretroviral drugs, is available from the drug manufacturer in the USA and in Europe. Clinicians must review manufacturer's product information before prescribing any of these drugs, and contact the protocol team at actg.penpact1@fstrf.org with any discrepancies or queries.

The PENPACT 1 Team will **review and** update Appendix IX whenever new drugs, drug combinations, drug dosing, and/or drug-regimens are approved by the FDA and/or EMEA to **ensure** that clinicians have the most current options **available** for prescribing children's ARV regimens.

In general, dosage based on weight or **body** surface area will be recalculated at least every 12 weeks. When the weight or body surface area (BSA) changes so that the dose (in mg) differs by ≥10% from the previously dispensed dose, the dose must be adjusted. Doses may be changed at <10% difference based on site preference.

4.5 Study Treatment for Second-Line Therapy

Second-line therapy will be **initiated** when **the HIV-1 RNA switch criterion (≥1,000 copies/mL or ≥30,000 copies/mL, depending on the initial randomization) is reached.** Section 5.0 describes the patient management criteria for **therapy** switch.

The following suggested drug regimens will be strongly encouraged as second-line therapy for all children failing **first-line therapy** (when HIV-1 RNA is ≥1,000 copies/mL or when HIV-1 RNA is ≥30,000 copies/mL, depending on the initial randomization)**, particularly those children who have remained on their initial (randomized) therapy**. **Those** antiretrovirals listed **in** Section 4.4 and Appendix IX will be used **in selecting** second-line therapy for each child.

- For PI containing Groups **1(A) and 1(B)**: two new NRTIs and an NNRTI
- For NNRTI containing Groups **2(A) and 2(B)**: two new NRTIs and a PI

However, these regimens do not constitute the only options. Current clinical care guidelines will prevail over protocol **guidelines**, (e.g. a fourth drug as part of second-line therapy). The protocol will allow low doses of ritonavir as a boosting agent, creating drug combinations that will be counted as a single PI.

5.0 PATIENT MANAGEMENT (HIV-1 RNA CRITERIA)

5.1 **Changes to First-Line** Therapy

First-line therapy includes the initial therapy to which a child is randomized as well as any antiretroviral therapies to which the child changes due to non-virologic reasons (e.g. toxicity, intolerability, request of child or child's parent(s) or LAR, etc.) prior to reaching the HIV-1 RNA switch criterion (≥1,000 copies/mL or ≥30,000 copies/mL, depending on the initial randomization). Whenever possible, changes within first-line therapy should involve substitutions of one or more drugs in the initial therapy by drugs from the same class or classes.

If a child does not start their initial therapy as randomized, then any antiretroviral therapy that he/she starts constitutes his/her first-line therapy.

Regardless of the specific drugs being taken as part of the first-line therapy (including any changes from the initial therapy), a child should be followed on first-line therapy until he/she reaches the HIV-1 RNA criterion for switching to second-line therapy (as randomized).

5.2 **Criteria for Changing from First-Line Therapy to Second-Line Therapy**

While on first-line therapy, a child who:

- fails to achieve his/her randomized **HIV-1** RNA **level for switching therapy** by Week 24 (**i.e. to <1,000 or to <30,000 copies/mL**), or
- experiences an initial decline in plasma HIV-1 RNA levels by Week 24 or later, and subsequently has an HIV-1 RNA level at or above his/her randomized **level** for switching **therapy** (i.e. at HIV-1 RNA either ≥1,000 or at ≥30,000 copies/mL),

must have a **confirmatory** HIV-1 RNA **determination obtained between** 14 **and** 35 days post initial HIV-1 RNA determination. If the **confirmatory** value is below the randomized **HIV-1 RNA** level for switching therapy, **first-line therapy and the** schedule **of evaluations for first-line therapy** should be continued as per Appendix I. If the **confirmatory** value is **greater than or equal to** the randomized **HIV-1 RNA level** for switching therapy, **the child should switch to second-line therapy. However, if poor adherence is suspected as a possible reason for an increase in HIV-1 RNA above the randomized HIV-1 RNA level for switching therapy, then the site should try to improve adherence and subsequently obtain the confirmatory HIV-1 RNA value. This should be accomplished within a five week time frame.**

Additionally, if at or after 24 weeks of first-line therapy a child experiences clinical disease progression (i.e. a new CDC Category C diagnosis) or experiences other clinical progression (such that the treating clinician believes that changing therapy is required prior to reaching the randomized HIV RNA viral load switch point), then the child should switch to second-line therapy.

PACTG sites should contact one of the **PACTG 390** Protocol Co-Chairs to discuss and request authorization for switching therapy**, including intentions to try to improve adherence before making the decision to switch therapy**. PENTA sites should contact the appropriate Trials Center prior to switching therapy. Sites should e-mail or fax this request to the **PENPACT 1 Team** log-on (actg.penpact1@fstrf.org) **and** copy **either** the **PACTG 390** Protocol Co-Chairs or the appropriate PENTA **Trials** Center.

This request for switching therapy must clearly describe the patient's clinical evaluation and clinician's choice(s) for second-line therapy. All supportive clinical, laboratory, and medical history records must be included in this request. The **PACTG 390 Protocol** Co-Chairs or the PENTA **Trials** Center will respond to this request upon receipt. A telephone conversation may be needed to finalize the choice for second-line therapy. However, the **PACTG 390 Protocol** Co-Chairs or the PENTA **Trials** Center **must send an e-mail** to the patient's clinician **with a copy to** the PENPACT 1 Team log-on (actg.penpact1@fstrf.org) **stating** the final choice **of and rationale for** second-line therapy. This notification/consultation is required by the protocol to enable the PENPACT 1 Team **to have** better control of the study (e.g., double checking that the switching point has indeed been reached and that second-line **therapy** has been selected correctly).

For children starting second-line therapy, restart the schedule of evaluations from the Entry visit (Week 0). Note that certain evaluations may not be required on second-line therapy; please pay special attention to all Appendix I footnotes.

5.3 **Criteria for Discontinuing First-Line Therapy**

It is intended that children will be followed on their first-line therapy until they meet the criteria for switching to second-line therapy, regardless of any changes in ARVs constituting their initial therapy. If, at the time the child meets the virologic switch criterion, and the child, child's parent(s) or LAR choose not to switch to second-line therapy, then the subject will be considered to be off study treatment but on study, and will continue to be followed according to the schedule of evaluations.

5.4 **Criteria for Discontinuing** Second-Line Therapy:

Second-line therapy includes any antiretroviral therapy that the child receives after failing first-line therapy (according to the criteria defined in Section 5.2) until the child next experiences virologic failure or disease progression as defined below. Wherever possible, changes in second-line therapy should involve substitutions of one or more drugs from the same class or classes as the child is already taking.

While on second-line therapy, a child who:

- fails to achieve <30,000 copies/mL HIV-1 RNA by Week 24 of second-line **therapy**, or
- experiences initial decline in plasma HIV-1 RNA by Week 24 **of second-line therapy**, and subsequently has an HIV-1 RNA level ≥30,000 copies/mL

must have a confirmatory HIV-1 RNA **determination** obtained **between** 14 and 35 days post initial HIV-1 RNA determination. **If the confirmatory value is also ≥30,000 copies/mL, then the child will be considered to have failed second-line therapy.**

Additionally, if at or after 24 weeks of second-line therapy, a child experiences clinical disease progression (i.e. a new CDC Category C diagnosis) or experiences other clinical progression (such that the treating clinician believes that changing therapy is required prior to reaching the randomized HIV-1 RNA viral load switch point), then the child will also be considered to have failed second-line therapy.

When a child fails second-line therapy (except as defined below) the child will discontinue study treatment, but continue on study follow-up. The child should then be offered the best available, individualized therapy at the discretion of the child's clinician and parent(s) or LAR. This new therapy will not be managed as part of PENPACT 1. However, if in the clinician's opinion the second-line therapy is the best available therapy (despite an HIV-1 RNA value ≥30,000 copies/mL), the patient will be allowed to continue on study treatment for as long as the clinician deems appropriate.

Children who discontinue second-line therapy for any reason should, if possible,

continue to have assessments undertaken every 12 weeks throughout follow-up. The Protocol Co-Chairs (PACTG sites) or the appropriate Trials Center (PENTA sites) should be contacted before discontinuation, and the appropriate CRFs stating reasons for switching or stopping second-line therapy should be completed.

6.0 ADVERSE EVENT REPORTING AND MANAGEMENT

6.1 Serious Adverse Experience (SAE)**/Expedited Adverse Event** Reporting

PACTG 390

PACTG 390 sites will follow **DAIDS expedited adverse event (EAE) reporting requirements. The EAE reporting requirements and definitions for this study and the methods for expedited reporting of adverse events (AEs) to the DAIDS Regulatory Compliance Center (RCC) Safety Office are defined in "The Manual for Expedited Reporting of Adverse Events to DAIDS" (DAIDS EAE Manual) dated May 6, 2004. The DAIDS EAE Manual is available on the RCC web site (http://rcc.tech-res-intl.com/eae.htm). PACTG 390 sites will continue to use Appendices IV and V for grading toxicities.**

AEs reported on an expedited basis must be documented on the DAIDS Expedited Adverse Event Reporting Form (EAE Reporting Form) available on the RCC web site. DAIDS EAE forms should be submitted to DAIDS through the Regulatory Compliance Center (RCC) Safety Office (RCCSafetyOffice@tech-res.com). In addition, the site investigator is required to submit AE information as required by local regulatory or other local authority.

This study uses the Intensive Level of expedited AE reporting as defined in the DAIDS EAE Manual. The study agents that must be considered in determining relationships of AEs requiring expedited reporting to DAIDS are the ARVs comprising the subject's regimen at the time of the AE. AEs must be reported on an expedited basis at the Intensive Level during the protocol-defined EAE Reporting Period, which is the entire study duration for an individual subject (from study enrollment until study completion or discontinuation of the subject from study participation for any reason) and for a period of 8 weeks after the subject's last study visit has been completed. After the end of the protocol-defined EAE Reporting Period stated above, sites must report serious, unexpected, clinical suspected adverse drug reactions if the study site staff becomes aware of the event on a passive basis, i.e., from publicly available information.

PENTA 9

PENTA 9 sites (the European portion of PENPACT 1) will follow the relevant clinical trials guidelines for reporting of SAEs, in accordance with the International Conference

for Harmonization (ICH) requirements **and the EU Clinical Trials Directive 2001/20/EC (20)**. Any serious adverse experience must be notified to the relevant Trials Center immediately via telephone or fax, and a SAE form must be completed and sent to the trials center.

The PACTG and PENTA teams will exchange and review data regarding serious adverse events every three months. The data will include, at minimum, event description, start and resolution dates, grade of the clinical or laboratory abnormality, classification of the **AE** for PACTG and PENTA, and action taken. These data exchanges will be coordinated by the PENPACT 1 protocol statisticians at SDAC (Statistical and Data Analysis Center) for PACTG sites and MRC and INSERM for PENTA sites.

6.2 Criteria for Management of Adverse Events/Toxicity and Dose Modification

Both PACTG and PENTA **sites** will manage any adverse event/toxicity following the guidelines described in this section and in Appendix X "Management of Specific Adverse Events for PACTG and PENTA". Furthermore, both PACTG and PENTA **sites** will refer to **the Division of AIDS Toxicity Tables for Grading Severity of Adverse Experiences in** Appendices IV and V for severity grades associated with toxicity management and dose modification and/or discontinuation.

Only Grade ≥2 toxicities or higher will be collected on PENPACT 1 (PACTG 390 or PENTA 9) study forms.

For all questions regarding any toxicity, consult the **PACTG 390 P**rotocol Co-Chairs (PACTG sites) or Trials Center (PENTA sites). Send an e-mail message to actg.penpact1@fstrf.org, including the PID, the SID, the question, and a brief, relevant history **and** the appropriate team member will answer.

6.21 Management of Specific Adverse Events:

The following specific adverse events must be managed as per Appendix X:

- Hypersensitivity reaction to abacavir (ABC)
- Skin rash/cutaneous dermatitis (other than ABC associated)
- Clinical pancreatitis
- Hyperlipasemia
- Hyperamylasemia
- Increase in values of liver function tests (LFTs)
- CNS symptoms
- Hyperglycemia/glycosuria
- Elevated cholesterol or triglycerides
- Hematologic toxicities
- Neutropenia and anemia
- Lactic acidosis

6.22 General Management Adverse Events:

All antiretrovirals prescribed for first-**line** and second-line therapies will be considered study **drugs**. Alternate explanations for clinical or laboratory abnormalities that may at first appear to be related to study treatment must be sought. The use of reduced doses of the study medications is discouraged and should be avoided.

Management of any other adverse event/toxicity that is not covered by Appendix X must be done as per the following criteria. For abnormal clinical or laboratory observations of:

6.221 Grade 1

- Continue study drugs.
- Routine monitoring.

6.222 Grade 2

- Continue study drugs.
- Monitor closely with more frequent visits (i.e. every two weeks).
- Work-up to exclude other causes.

6.223 Grade 3 (See Clarification Note at the end of **Section** 6.225)*

- Repeat observation within 72 hours for confirmation.
- Subjects should continue taking study drugs pending receipt of the confirmatory laboratory tests.
- Work-up to exclude other causes.
- Clinician has the option of immediately stopping the study drugs if a repeat confirmatory laboratory test cannot be performed within 72 hours, or if the clinician determines that the continuation of study drugs is unsafe while awaiting test results.
- Subjects will be allowed to interrupt study treatment for up to 14 days.
- If toxicity persists at Grade 3 for more than 14 days, recurs on re-challenge, or after replacing a specific drug, all study drugs must be permanently discontinued. **If the child was on first-line therapy at the time of drug discontinuation, then a new first-line regimen may be initiated and the child should continue to be followed on first-line therapy. The selection of the new regimen will be done**

while the child is off study drugs. Clinicians must discuss regimen options with the PACTG 390 Protocol Co-Chairs (PACTG sites) or Trials Center (PENTA sites). Send an e-mail message to actg.penpact1@fstrf.org including the new drug options. The appropriate PENPACT 1 team member will respond. This change in therapy due to toxicity will not constitute a "switch" from first-line to second-line therapy. If the child was on second-line therapy at the time of drug discontinuation, then a new second-line regimen may be initiated and the child should continue to be followed on second-line therapy.

6.224 Specific for All Confirmed Grade 3 Toxicities

- That can not be attributable to only one of the study drugs, stop all study drugs until toxicity resolves to ≤Grade 2, then re-start therapy (all study medications).
- That can be clearly attributable to a specific drug in one class (NRTI, PI or NNRTI), stop all study drugs until toxicity resolves to ≤Grade 2. Then, restart therapy but discontinue the implicated drug permanently and continue all other medications. Replace the specific drug with another in the same class.

  The selection of the new drug for this replacement will be done while the child is off study medications. Clinicians must discuss the drug options with the study Co-Chairs (PACTG sites) or Trials Center (PENTA sites). Send an E-mail message to actg.penpact1@fstrf.org establishing the new drug options. The appropriate PENPACT 1 Team member will respond. This change in therapy due to toxicity will not constitute a **discontinuation of first-line or second-line therapy.**

6.225 Grade 4 (See Clarification Note)*

- Hold study drugs and notify study team to determine course of action
- Obtain confirmatory laboratory results within 72 hours and notify study team of those results
- Work-up to exclude other causes
- For all confirmed Grade 4 toxicities stop all study drugs until toxicity resolves to ≤Grade 2
- For all confirmed Grade 4 toxicities that can be undeniably and clearly attributable to a specific drug in one class (NRTI, PI or NNRTI), stop all study drugs until toxicity resolves to ≤Grade 2. Then, restart therapy but discontinue the implicated drug permanently and continue

all other medications. Replace the specific drug with another in the same class. The selection of the new drug for this replacement will be done while the child is off study medications. Clinicians must discuss the drug options with the study Co-Chairs (PACTG sites) or Trials Center (PENTA sites). Send an E-mail message to actg.penpact1@fstrf.org establishing the new drug options. The appropriate PENPACT 1 Team member will respond. This change in therapy due to toxicity will not constitute a **discontinuation of first-line or second-line therapy.**

- Subjects will be allowed to interrupt study treatment for up to 14 days

- If toxicity persists at Grade 4 for more than 14 days, recurs on re-challenge, or recurs after replacing a specific drug, all study drugs must be permanently discontinued. **If the child was on first-line therapy at the time of drug discontinuation, then a new first-line regimen may be initiated, and the child should continue to be followed on first-line therapy. The selection of the new regimen will be done while the child is off study drugs. Clinicians must discuss regimen options with the PACTG 390 Co-Chairs (PACTG sites) or Trials Center (PENTA sites). Send an e-mail message to actg.penpact1@fstrf.org including the new drug options. The appropriate PENPACT 1 team member will respond. This change in therapy due to toxicity will not constitute a "switch" from first-line to second-line therapy. If the child was on second-line therapy at the time of drug discontinuation, then a new second-line regimen may be initiated and the child should continue to be followed on second-line therapy.**

*Clarification Note:

For all Grade 3 and 4 toxicities, send an e-mail to the Protocol Team at actg.penpact1@fstrf.org (this address already includes penta@ctu.mrc.ac.uk and a.compagnucci@vjf.inserm.fr) within 48 hours of the event. The appropriate team member from the PACTG or PENTA group will respond. Please provide the team with all confirmatory laboratory results for Grade 3 and Grade 4 toxicities.

7.0 COORDINATION OF THE TRIAL, DATA COLLECTION, AND MONITORING

The PENPACT 1 Team, as described at the beginning of this document, will be responsible for the integrity of the two studies, PACTG 390 and PENTA 9, as equal parts of PENPACT 1. The coordination, data collection, and monitoring of the PACTG 390 study will be a direct

responsibility of the PACTG 390 Team in the USA, and likewise for the PENTA 9 team in Europe.

Case Report Forms (CRF) will be provided for each child. Children must not be identified by name on any study documents, but by trial number for PENTA sites and by the PID and SID provided by the ACTG Data Management Center for the PACTG sites **at** randomization.

All data on the CRFs must be legibly recorded in black ink or typed. A correction should be made by striking through the incorrect entry with a single line and entering the correct information adjacent to it. The correction must be initialed and dated by the investigator or a designated, qualified individual. Any requested information that is not obtained as specified in the protocol should have an explanation noted on the CRF as to why the required information was not obtained.

Instructions concerning the recording of study data or the entry of such data into the computerized data base will be provided by the ACTG Data Management Center (PACTG sites) and by the Trials Centers (PENTA sites).

7.1 Trial Coordination

At sites affiliated with the PACTG, the trial will be coordinated by Frontier Science and Technology Research Foundation.

In Europe, the trial will be coordinated jointly by two coordinating Trials Centers at the MRC Clinical Trials Unit, London, and at INSERM, SC10, Paris, under the auspices of PENTA. The liaison between the coordinating trial centers and centers in each country for running the study will be similar to that organized for other PENTA trials, with a combination of direct liaison and liaison via local coordinating centers.

The lead study statisticians from both PACTG and PENTA will be jointly responsible for defining the structure of the merged data (PENPACT 1) and the contents of the interim and final analysis and statistical reports.

The responsibility for finalizing the merged data, undertaking the statistical analysis, and preparing and distributing the report for each interim analysis to the DSMB will alternate between the PACTG and PENTA statistical centers. The lead statisticians from both organizations will attend all DSMB meetings.

7.2 Regional Monitoring

Monitors under contract to NIAID or NICHD will visit **PACTG-affiliated** clinical sites.

In Europe, for PENTA sites, monitoring will be undertaken by each clinical center under the guidelines and supervision of the MRC or INSERM depending on the country.

PACTG and PENTA monitors will review the research records for accuracy, completeness, and legibility. They will inspect sites' regulatory files and pharmacies to ensure that regulatory requirements are being met, and will review all research records for the status of new enrollments to determine if any improvements are needed at a specific site. For confirmation of the study data, site investigators must make study documents (e.g., consent forms, drug distribution forms, and CRFs), and pertinent hospital or clinic records readily available for inspection by the site monitors, the FDA (**PACTG affiliated sites)**, and the EMEA (European **sites)**.

Site visits will be made at main units and the larger sub-units at regular intervals or more frequently as directed by NIAID, NICHD or PENTA.

8.0 DRUG ACCOUNTABILITY AND ADHERENCE

8.1 Accountability

For PACTG sites accountability records will not be needed. Study medications will not be provided by the study but by prescription (the child, child’s parent**(s)/LAR**, the child’s health insurance, and/or, in Europe, the healthcare provider, are responsible for purchasing the study medications).

For PENTA sites, the trial pharmacist is required to maintain complete records of all study medication dispensed. The procedures to be followed will be sent directly to the trial pharmacist and will adhere to the Good Clinical Practices (GCP) guidelines on drug accountability.

8.2 Adherence

To date, there is no gold standard for the assessment of adherence. Adherence assessment strategies commonly used are self-report, pill counts, electronic monitoring, urine and serum assays, and the addition of tracers to liquid medications. Each of these methods is imperfect and subject to error.

The more accurate methods are more expensive and labor intensive, as are combinations of these methods. However, due to the critical nature of the problem, the PACTG Adherence Subcommittee has piloted two measures of self-reported adherence: Adherence Modules 1 and 2. The PENTA group has also piloted an adherence questionnaire for self-completion.

Self-reported data are clearly subject to a number of biases, but, within the context of large clinical trials, self-reporting provides pragmatic means for estimating adherence rates.

In PENPACT 1, **the** parent**(s)/LAR** will receive instructions as to the appropriate administration of study drugs and must demonstrate the ability to dispense the study drugs prior to receiving the child's drug supply.

PENPACT 1 will collect adherence information using Adherence Modules 1 and 2 at PACTG sites, and the Self-Completion Questionnaire at PENTA sites.

9.0 STATISTICAL CONSIDERATIONS

9.1 General Design Issues

This is an international, multi-center Phase II/III, randomized, open-label, clinical trial. It uses a factorial (2x2) design to allow separate comparisons, specified in the primary and secondary objectives, to be evaluated.

9.2 Primary Outcome Measure

The primary outcome measure to be used in addressing the two primary objectives is change in viral load measured in $\log_{10}$ HIV**-1** RNA copies/mL between baseline and four years post-randomization.

The average of the pre-entry and entry values will be used for baseline, and the average of the values obtained at Weeks 192 and 204 will be used for the concluding viral load. Missing values will be handled as defined later in this statistical section.

9.3 Secondary Outcome Measures

The following outcome measures are those required to evaluate each of the secondary objectives:

- Number of Grade 3 or higher signs, symptoms, or laboratory abnormalities experienced.
- Change in immunologic outcome will be defined as the change in CD4% from baseline (mean of pre-entry and entry values) to four years (mean of values at Weeks 192 and 204).
- Time to a significant HIV-related clinical event will be defined as the time to first new CDC Category C diagnosis (except for re-current bacterial infections).
- Whether or not a child switched regimens.
- Time to HIV-1 RNA >400 copies/mL during first line-therapy or permanent discontinuation of first-line therapy. Defined as time from randomization to first of two consecutive evaluations after Week 24 with measurement of >400 copies/mL while on first-**line** therapy, or to permanent discontinuation of the first-line therapy for any reason, whichever event comes first.
- Time to HIV-1 RNA ≥ 30,000 copies/mL during second line-therapy or permanent discontinuation of second-line therapy. Defined as time from randomization to the first of two consecutive evaluations with measurement >30,000 copies/mL while on the second-line therapy, or to permanent discontinuation of the second-line therapy

for any reason, whichever event comes first.

- Whether or not a child has an HIV-1 RNA level <400 copies/mL at Week 24 and has not permanently discontinued first-line therapy prior to that week.
- Whether or not a child has an HIV-1 RNA level <400 copies/mL at Week 204 regardless of therapy at that time.

There will be a continued statistical follow-up beyond 204 weeks of treatment, which will be used to address time-to-event secondary objectives of the study.

9.4 Randomization and Stratification

Children will be randomized to four groups stratified by age (<3 years versus ≥3 years), origin (PACTG sites or PENTA sites), and exposure versus no exposure to antiretroviral therapy perinatally.

Thus, PENPACT 1 children will be randomized to one of four groups, in a 2x2 factorial design. The randomization will be stratified by:

(1) age (prior to 3rd birthday versus on or after 3rd birthday),

(2) site (PACTG versus PENTA), and

(3) perinatal exposure to antiretroviral therapy (exposed, defined as less than 56 consecutive days after birth of antiviral drugs used to prevent mother-to-infant transmission) versus naïve or not exposed.

There will be no limits on the number accrued to each stratum.

9.5 Sample Size and Accrual

The study is designed to accrue a total of 256 children. Each of the pairwise comparisons of randomized groups in the factorial design will therefore involve comparisons between two groups each of size 128 children.

The power to detect a difference in mean change in HIV-1 RNA from baseline to four years will depend on the proportion of children who have HIV-1 RNA levels below the limit of quantification of the assay (400 copies/mL) at that time. If no child has a level below this limit, then it is estimated that there will be at least 90% power to detect a difference between groups of 0.3 $\log_{10}$ HIV-1 RNA copies/mL using an 0.05 level of significance, and assuming up to 10% of children will have no HIV-1 RNA measurements at 4 years.

This estimate of power is based upon a standard deviation of 0.7 $\log_{10}$ HIV-1 RNA copies/mL at four years post-randomization, after adjusting for baseline viral load. This standard deviation was obtained in the Delta study among 330 adults at 96 weeks, and in PACTG 152 among 47 children at 144 weeks, treated with NRTIs. If the standard

deviation is larger than 0.7 $\log_{10}$ copies/ml, then there will still be good power to detect small but clinically relevant differences in pair-wise comparisons. For example, if the standard deviation is 0.8, 0.9 or 1.0 $\log_{10}$ copies/ml, then there will be at least 90% power to detect differences of 0.34, 0.38 and 0.43 $\log_{10}$ copies/ml, respectively.

With moderate proportions of children in either or both groups with HIV-1 RNA levels below the limit of quantification of the assay, the power will be reduced modestly. For example, using results presented by Hughes (**21**) with 40% of children with censored measurements (HIV RNA below the lower limit of detection or above the upper limit of detection), there will be at least 90% power to detect a difference between groups of about 0.5 $\log_{10}$ copies/mL.

This number of children will also allow detection of a minimal difference of 3.2% for change in CD4% from baseline to 4 years post-randomization between each of the pair wise comparisons with the same power and alpha levels, assuming the standard deviation of the change in CD4% from baseline to 4 years is 7.5% (the standard deviation of the change in CD4% from baseline to Week 96 in PACTG 338).

9.6 Monitoring

9.61 Routine Monitoring

Routine monitoring will be conducted to keep the study team informed about progress of the study without revealing information on efficacy outcomes.

During the accrual phase of the study, routine reports summarizing accrual, losses to followup, and completeness of data collection pooled across all randomized Groups will be reviewed by the study team on a monthly basis.

When accrual is complete, the frequency of reporting will be reduced to every three months.

In the event that the team is notified of any serious adverse experiences or deaths that cause concern to the team, then a review by the Data and Safety Monitoring Board will be requested.

9.62 Interim Analyses

An independent Data and Safety Monitoring Board (DSMB) will be established, PENPACT 1-DSMB. There will be equal representation of PACTG and PENTA nominees on the DSMB. PENTA will provide the names of three PENTA representatives to the DSMB, and DAIDS will name three representatives from the current DAIDS Therapeutic DSMB (TDSMB).

PENPACT 1-DSMB will review the study data at least once a year and may request

more frequent interim safety or efficacy reviews as needed. The PENPACT 1 Team may request additional DSMB safety reviews if needed. The PENPACT 1-DSMB will follow the SOP of the DAIDS Therapeutic DSMB (TDSMB).

No member of the U.S./European executive committee for the trial, and no clinician (investigator) responsible for the clinical care of trial patients, involved in PENPACT 1 will be a member of the PENPACT 1-DSMB.

The PENPACT 1-DSMB will review study data every 12 months (or as often as needed), in strict confidence, on the activity, efficacy, and toxicity measures by treatment allocation and will advise the PENPACT 1 Team on whether the number of individuals to be recruited is appropriate.

The PENPACT 1-DSMB may initiate interim analyses for activity, efficacy, and toxicity. One detailed interim analysis will be undertaken when half the children have been followed for two years.

The PENPACT 1-DSMB will also consider the findings from other relevant studies and will advise the executive committee if, in their view, the data have provided both:

- Proof beyond reasonable doubt[4] that one of the randomized factors is better in terms of the primary outcome, and
- Evidence that might be reasonably expected to materially alter the uncertainties of clinicians, who are already aware of the results of other trials.

9.7 Analysis

All analyses will be based on intention to treat except for toxicity analyses, when follow-up will be censored eight weeks after treatment is stopped.

9.71 Primary Analysis

Primary comparisons (NNRTI versus PI; 1,000 versus 30,000 HIV-1 RNA copies/mL for change of treatment) with respect to change in HIV-1 RNA from baseline to four years will be performed using analysis of covariance. The analysis will adjust for baseline viral load and the three stratification factors. As values below the limit of quantification of the assay will be considered as censored measurements, the analysis will use likelihood-based methods for censored data.

[4] Appropriate criteria for proof beyond reasonable doubt cannot be specified precisely; but, for example, a difference of at least three standard deviations in an interim analysis of a major outcome measure may be needed to justify closing or modifying this study prematurely. This criterion has the practical advantage that the exact number of interim analyses would be of little importance.

The above analysis will include children who die from HIV-related causes before completing four years in the study taking their baseline HIV-1 RNA level as their measurement at four years, but otherwise will exclude subjects who have missing values for HIV-1 RNA at four years, and may be sensitive to the handling of censored measurements if the proportion of censored measurements is high. However, the sensitivity of the conclusions of the study to these two issues will be evaluated using two methods:

- For a child with missing viral load at 4 years, let T be the time from randomization to the last available HIV-1 RNA measurement. The extrapolated value of viral load at 4 years can be taken as the viral load predicted from the linear regression model for viral load measurements from baseline to time T extrapolated out to 4 years, using data from all children in the same treatment group.

  These extrapolations, which assume missing at random but not necessarily missing completely at random, will lead to a slight underestimation of the standard error of the treatment effect, which can be ignored if the proportion of children dead or lost to followup is small. Alternatively, multiple imputation methods can be used to adjust standard errors.

- A rank based analysis will be undertaken. Children will be ranked according to their HIV-1 RNA level, including censored measurements, at four years. Children who died prior to four years will be ranked as worse outcomes than any child who survives to four years, with a worse ranking for deaths closer to the date of randomization.

  The sensitivity of results to children who are lost to followup will then be investigated by three different approaches: ranking loss to followup at time T as equivalent to death at time T (this is a severe approach), using the average of the last two observations carried forward, and using the linear regression model of a subject's measurements over the period baseline to time T to give an extrapolation to time T.

9.72 Secondary Analyses

Prior to any formal interim analysis and to the final analysis, a detailed analysis plan will be prepared. The following summarizes the general approach to analysis that will be used for the different types of secondary outcome measures.

For continuous outcomes (e.g. CD4%), analysis of covariance in a similar way to that described above for changes in HIV-1 RNA will be used. For repeated measurements over time, the area under the curve will also be calculated and compared between randomized Groups.

For binary outcome measures (e.g. whether or not a child switched regimens), chi-squared tests (stratified by factors used in the randomization) will be used to compare randomized Groups, and logistic regression adjusted for these stratification variables will be used to provide estimates of effect.

For counts of events (e.g. number of adverse experiences, or number of regimen switches), stratified chi-square tests or Wilcoxon tests will be used to compare randomized Groups, and Poisson regression adjusted for stratification variables and duration of followup will be used to provide estimates of effect.

For time-to-event outcome measures (e.g. time to HIV-1 RNA ≥400 copies/mL or treatment discontinuation while on first-line therapy), stratified log-rank tests will be used to compare randomized Groups, and Kaplan-Meier estimates and stratified proportional hazards modeling will be used to provide estimates of effects.

10.0 HUMAN SUBJECTS

10.1 Declaration of Helsinki

The PENTA 9 trial (the European portion of PENPACT 1) will conducted in full conformance with the principles of the Declaration of Helsinki (as amended in Tokyo, Venice, Hong Kong and Edinburgh), and with the local laws and regulations concerning clinical trials.

10.2 PACTG Institutional Review Board (IRB) Review and Informed Consent

This protocol document and the PACTG sample informed consent documents (Appendices XVII **and XVIII**), as well as any subsequent modifications, will be reviewed and approved by the IRBs or ethics committees responsible for oversight of the study.

Each site which receives US HHS funding and follows the United States Code of Federal Regulations Title 45 – Public Welfare, Part 46 – Protection of Human Subjects (also known as the Common Rule) should have on record at the site a plan that detects and addresses any change in guardianship occurring in pediatric subjects and determines when a study subject must have a consent process which involves a LAR other than a family member with guardianship. The plan will include how the site determines when a LAR is initially or no longer needed and how frequently the LAR resigns the consent. The plan should follow all IRB, local, and state guidelines. Confirmation of such a plan at a site should be submitted with protocol registration materials.

10.3 PENTA Ethics and Regulatory Approval Committee

At each center, one pediatrician (the investigator) will take on overall responsibility for the conduct of the trial. He/she will submit the protocol and any subsequent amendments to the ethics committee.

This protocol, the informed consent documents and adherence questionnaire will be formally approved by the relevant ethics committee of each clinical center. Before the study can start, the center must send a signed copy of the Investigator's Agreement to Participate. The study will not commence in any country until appropriate approval has been obtained from the appropriate Regulatory Approval.

10.4 Confidentiality

All laboratory specimens, evaluation forms, reports, and other records will be identified only by a coded number to maintain confidentiality. All records will be kept in locked file cabinets. All computer entry and networking programs will be done with coded numbers only. Clinical information will not be released without written permission, except as necessary for monitoring by the trial monitors, the FDA, the NIAID or the EMEA.

10.5 PACTG Study Discontinuation:

The PACTG 390 portion of PENPACT 1 study may be discontinued at any time by the NIAID, the FDA, or the PACTG Protocol

10.6 PENTA Study Discontinuation:

The PENTA 9 portion of PENPACT 1 study may be discontinued at any time by the EMEA or the PENTA Executive Committee.

11.0 PUBLICATION OF RESEARCH FINDINGS

The PENPACT 1 Team will be responsible for preparing the manuscript for rapid publication. The authorship will be under PENPACT 1 Team. No other publications, either written or verbal, will be made before the definitive manuscript has been accepted for publication without approval of the PENPACT 1 Team.

PACTG and PENTA will share all the data from PACTG 390 and PENTA 9. The merged data will be the property of both organizations, and this policy will be reflected in all publications deriving from these data. Publication of unmerged PENPACT 1 data, data from PACTG 390, PENTA 9, or PENPACT 1 manuscript, will be allowed independently from the main PENPACT 1 manuscript, but only after approval review by the PENPACT 1 Team.

The data from PENTA 9--PENPACT 1-B Sub-study of lipodystrophy will be the property of PENTA, but will be shared with the whole PENPACT 1 Team before publication.

12.0 SAMPLES CLARIFICATION

All laboratory samples collected at PACTG sites will be managed and/or stored as per PACTG guidelines.

All laboratory samples collected at PENTA sites will be managed and/or stored as per PENTA guidelines following the corresponding country laws and regulations.

There will be no exchange, aliquoting, or storing of PACTG samples with PENTA or vice-versa. Samples will be used for tests described in the samples informed consent for each organization. Testing of samples for non-authorized assays other than what is signed in the informed consent will not be allowed. Both informed consents clearly state that testing of stored samples will be for future PACTG or PENTA approved AIDS related research; no other testing will be allowed.

13.0 BIOHAZARD CONTAINMENT

Transmission of HIV and other blood-borne pathogens can occur through contact with contaminated needles, blood, and blood products. Appropriate blood and secretion precautions must be employed by all personnel when drawing blood, shipping and/or handling of all specimens for this study, as currently recommended by the CDC.

For PACTG sites, since the International Air Transportation Association (IATA) regulations for the shipment of HIV containing specimens are currently being updated (March 2003), please refer to the each individual carrier gudelines (e.g. FedEx, Airborne) and the ACTG Website for specific instructions and shipping guidelines for these specimens.

14.0 PENTA CENTERS LIABILITY/INSURANCE

In consideration of the agreement by the Principal Investigator at each site to supervise the trial, the PENTA Group undertake to indemnify the Principal Investigator at each site and the institutions which participate in the trial and their servants and agents in respect of any claims made against them by any third party which arises out of or as a result of the supervision or conduct of the trial (including any claim arising in respect of the technical procedures described in the protocol which patients/subjects would not have been exposed but for their participation in the trial). Full details of the Indemnity agreement are given in a separate document.

15.0 REFERENCES

1. Englund JA, Baker CJ, Raskino C *et al.* Zidovudine, didanosine, or both as the initial treatment for symptomatic HIV-infected children. *N Engl J Med* 1997, 336, 1704.

2. McKinney RE, for the PACTG 300 Protocol Team. Pediatric ACTG Trial 300: Clinical efficacy of ZDV/3TC vs. ddI vs. ZDV/ddI in symptomatic, HIV-infected children. *35th Annual Meeting of the Infectious Diseases Society of America*, San Francisco, 1997, Abstract 768.

3. Gibb D. The safety and tolerability of zidovudine (ZDV) and zalcitabine (ddC) in children with symptomatic HIV infection – PENTA 3. *6th European Conference on Clinical Aspects and Treatment of HIV Infection*, Hamburg, 1997, Abstract A469/B15.

4. PENTA 4. A randomized double-blind trial of the addition of lamivudine or matching placebo to current nucleoside analogue reverse transcriptase inhibitor therapy in HIV-infected children: PENTA-4 trial. AIDS 1998, 12:F151-F160.

5. Kline MW, Van Dyke RB, Lindsey JC *et al.* Combination therapy with stavudine (d4T) plus didanosine (ddI) in children with human immunodeficiency virus infection (PACTG 327). *Pediatrics*, 1999, 103, 62.

6. Palella FJ, Delaney KM, Moorman AC, *et al.* Declining morbidity and mortality among patients with advanced human immunodeficiency virus infection. *N Engl J Med*, 1998, 338, 853-60.

7. Gharakhanian S, Salhi Y, Nguyen TH, *et al.* Frequency and lipodystrophy and factors associated with glucose/lipid abnormalities in a cohort of 650 patients treated by protease inhibitors. *6th Conference on Retroviruses and Opportunistic Infections*, Chicago, 1999, Abstract 642.

8. Katlama C, Murphy R, Johnson V, *et al.* The Atlantic Study: A randomized open-label study comparing two PI-sparing antiretroviral strategies versus a standard PI-containing regime. *6th Conference on Retroviruses and Opportunistic Infections*, Chicago, 1999, Abstract 18.

9. Staszewski S, Morales_Ramirez J, Tashima KT et al. Efavirenz plus zidovudine and lamivudine, efavirenz plus indinavir, and indinavir plus zidovudine and lamivudine in the treatment of HIV-1 infection in adults. Study 006 Team. N Engl J Med. 1999; 341 (25):1865-73.

10. Staszewski S, Keiser P, Gathe J, *et al.* Ziagen/Combivir is equivalent to indinavir/Combivir in antiretroviral therapy naïve adults at 24 weeks. *6th Conference on Retroviruses and Opportunistic Infections*, Chicago, 1999, Abstract 20.

11. Van Dyke R, Saez-Llorens X, Nelson RP, *et al.* Antiretroviral activity and safety of abacavir (1592, ABC) with 3TC/ZDV in therapy experienced children. *12th World AIDS Conference*, Geneva 1998, Abstract 12255.

12. Stoddart C, Mammano F, Moreno M, *et al.* Lack of fitness of protease inhibitor-resistant HIV-1 in vivo. *6th Conference on Retroviruses and Opportunistic Infections*, Chicago, 1999, Abstract 4.

13. Faye A, Race E, Obry V et al. Viral Fitness in Patients with Discordant CD4 and Plasma HIV RNA following Protease Inhibitor Failure. 6th Conference on Retroviruses and Opportunistic Infections, Chicago 1999, Abstract 331.

14. Staszewski S et al. Efavirenz plus zidovudine and lamivudine, efavirenz plus indinavir, and indinavir plus zidovudine and lamivudine in the treatment of HIV-1 infection in adults. Study 006 team. N Engl J Med 1999;341:1865-73.

15. Vibhagool, A et al. Abstract 63, First IAS Conf, 2001.

16. Shulman NS et al. Genotypic correlates of a virologic response to stavudine after zidovudine monotherapy. JAIDS 2001;27:377-80.

17. Valentine ME et al. Evaluation of surrogate markers and clinical outcomes in two-year follow-up of eighty-six human immunodeficiency virus-infected pediatric patients. Pediatr infect Dis J, 1998;17:18-23.

18. Mofenson LM et al. The relationship between serum human immunodeficiency virus type 1 (HIV-1) RNA level, CD4 lymphocyte percent and long-term mortality risk in Hiv-1-infected children. J Inf Dis 1997;175:1029-38.

19. Palumbo et al. Disease progression in HIV-infected infants and children: predictive value of quantitative plasma HIV RNA and CD4 lymphocyte count. JAMA 1998;279:756-61.

20. **Directive 2001/20/EC of the European Parliament and of the Council of 4 April 2001 on the approximation of the laws, regulations and administrative provisions of the Member States relating to the implementation of good clinical practice in the conduct of clinical trials on medicinal products for human use. Available at http://www.eortc.be/Services/Doc/clinical-EU-directive-04-April-01.pdf.**

21. Hughes MD. Analysis and design issues for studies using censored biomarker measurements with and example of viral load measurements in HIV clinical trials. Statistics in Medicine 2000; 19:3171-3191.

APPENDIX I

SCHEDULE OF EVENTS FOR PACTG AND PENTA FOR BOTH **FIRST-LINE** AND SECOND-LINE THERAPIES

EVENTS	Initial Therapy	SUBJECT'S WEEK OF TREATMENT **FOR BOTH FIRST-LINE AND** SECOND-LINE THERAPIES[22]																					
	Screening [19] Week -2	Entry [20] Week 0	2	4[21]	8	12	16	24	36	48	60	72	84	96	108	120	132	144	156	168	180	192	204
Signed Consent	X																						
History & Physical[1]	X	X	X	X	X	X	X	X	X	X	X	X	X	X	X	X	X	X	X	X	X	X	X
Tanner Scales[2]		X					X		X	X	X	X	X	X	X	X	X	X	X	X	X	X	X
Neurologic Exam [3]		X																					
Pregnancy Test[4]	X	X			X		X		X	X	X	X	X	X	X	X	X	X	X	X	X	X	X
Hematology [5]	1.5	1.5	1.5	1.5	1.5	1.5	1.5	1.5	1.5	1.5	1.5	1.5	1.5	1.5	1.5	1.5	1.5	1.5	1.5	1.5	1.5	1.5	1.5
Chemistries[6]	1.0	1.0		1.0	1.0		1.0	1.0	1.0	1.0	1.0	1.0	1.0	1.0	1.0	1.0	1.0	1.0	1.0	1.0	1.0	1.0	1.0
Lymphocyte Subsets[7]	1.0	1.0			1.0		1.0	1.0	1.0	1.0	1.0	1.0	1.0	1.0	1.0	1.0	1.0	1.0	1.0	1.0	1.0	1.0	1.0
HIV-RNA [8,9]	3.0	3.0			3.0		3.0	3.0	3.0	3.0	3.0	3.0	3.0	3.0	3.0	3.0	3.0	3.0	3.0	3.0	3.0	3.0	3.0
Plasma and PBMC for storage[10,11]	4.0	4.0						4.0		4.0		4.0		4.0		4.0		4.0		4.0		4.0	**4.0**
Urinalysis[12]		X				X		X		X	X	X	X	X	X	X	X	X	X	X	X	X	X
Adherence Determination[13]								X		X		X		X		X		X		X		X	
Neuropsych Assessment[14,15, 16, 17,18]		X						X		X				X				X				X	
Max. Blood (mL)	10.5	10.5	1.5	2.5	6.5	1.5	6.5	10.5	6.5	10.5	.6.5	10.5	6.5	10.5	6.5	10.5	6.5	10.5	6.5	10.5	6.5	10.5	**10.5**

APPENDIX I (Cont)

EVENTS	SUBJECT'S WEEK OF TREATMENT **FOR BOTH FIRST-LINE AND** SECOND-LINE THERAPIES[22]										SWITCHING THERAPY/STUDY TERMINATION	EVALUATIONS FOR OFF STUDY TREATMENT BUT ON STUDY FOR BOTH FIRST-LINE AND SECOND-LINE THERAPIES (every 12 weeks, **unless indicated otherwise**)
	216	228	240	252	264[22]	276	288	300	312	324[22]		
History & Physical[1]	X	X	X	X	X	X	X	X	X	X	X	X
Tanner Scales[2]	X	X	X	X	X	X	X	X	X	X	X	X
Neurologic Exam [3]											X	X **(see footnote 3)**
Pregnancy Test[4]	X	X	X	X	X	X	X	X	X	X	X	X
Hematology[5]	1.5	1.5	1.5	1.5	1.5	1.5	1.5	1.5	1.5	1.5	1.5	1.5
Chemistries[6]	1.0	1.0	1.0	1.0	1.0	1.0	1.0	1.0	1.0	1.0	1.0	1.0
Lymphocyte Subsets[7]	1.0	1.0	1.0	1.0	1.0	1.0	1.0	1.0	1.0	1.0	1.0	1.0
HIV-RNA [8,9]	3.0	3.0	3.0	3.0	3.0	3.0	3.0	3.0	3.0	3.0	3.0	3.0
Plasma and PBMC for storage[10,11]	4.0		4.0		4.0		4.0		4.0		9.0	4.0 **(every 24 weeks)**
Urinalysis[12]	X	X	X	X	X	X	X	X	X	X	X	
Adherence Determination[13]	X		X		X		X		X		X	
Neuropsych Assessment[14,15, 16,17, 18]			X				X					X **(see footnote 18)**
Max. Blood (mL)	10.5	6.5	10.5	6.5	10.5	6.5	10.5	6.5	10.5	6.5	15.5	10.5

(1) **The** physical exam includes height and weight determinations (for PACTG sites, follow instructions in Appendix XIV; for PENTA sites, follow procedures described in Appendix III), head circumference (only for children <3 years of age, and should be done for both PACTG and PENTA sites, as per Appendix XIV), and HIV assessment.

(2) The Tanner Scale evaluation will be applied to any child **(a)** weighing ≥30 kg or **(b)** >9 years of age (see Appendix XV).

(3) A neurological exam will be performed for children at **domestic and international PACTG sites only at indicated time points** and whenever there is a neurological problem. **For those subjects who are off study treatment but on study follow-up, the neurological examination only needs to be completed whenever there is a neurological problem.**

(4) The pregnancy test **can** be either a urine sample or blood sample test (HCG). This test will be performed for all females of childbearing

APPENDIX I (Cont)

potential at **indicated** time points. The initial pregnancy test must be done within 72 hours of enrollment and the results must be received before starting study medications.

(5) The hematology blood samples should be collected in EDTA tubes. These samples will be analyzed for complete blood counts, cell differential, and platelet counts.

(6) The chemistry blood samples should be collected in anticoagulant free tubes. These samples will be analyzed for creatinine, total bilirubin, ALT (SGPT), AST (SGOT), triglyceride, cholesterol, glucose, and total amylase levels.

(7) The lymphocyte subset blood samples should be collected in EDTA, Hep, or ACD tubes. These samples will be analyzed for CD3+4, +CD3+8, and CD19**, if possible**.

(8) For all virology samples, follow Appendices VII and VIII for collection, **processing,** and shipping instructions.

(9) HIV-RNA blood samples should be collected in EDTA tubes.
- For PACTG sites: **Patient management HIV-1 RNA PCR will be run locally at a DAIDS VQA certified laboratory. Plasma aliquots from specimens collected at Screening, Entry, Week 24, time of therapy switch, Week 192, Week 204, and end of study must be reserved; these aliquots will be batched and shipped to the University of North Carolina prior to study completion.**
- For PENTA sites: P**atient management HIV-1 RNA PCR will be run locally.**
- For both PACTG and PENTA sites: **The average of the Screening and Entry** HIV RNA values will be used as **the** baseline value; **the average of the Week 192 and 204 HIV RNA values will be used for the concluding viral load.**

(10) For PENTA sites: An additional 3.0 mL of blood (7.0 mL total) must be collected at **each indicated time point.** These extra 3.0 mL samples will be used for HIV-1 RNA PCR determinations at central laboratories, **particularly at** Screening, Entry, **Week 24, time of therapy switch,** Week 192, Week 204**, and end of study**.
For both PACTG and PENTA sites: The stored plasma and PBMC specimens will be used for PACTG and PENTA-approved AIDS-related assays. Follow Appendices VII and VIII for **collection, processing, and shipping instructions.**

(11) An extra 5 mL **(9 mL total)** EDTA blood sample will be collected at Switching Therapy/Study Termination. These samples will be used for genotyping and pharmacokinetic assays for retrospective analysis, as determined by the PENPACT 1 Team. These samples will be processed to collect plasma and PBMC for storage.

(12) The urinalysis test will be performed using the reagent strip method.

(13) For PACTG sites, adherence will be determined by Adherence Modules 1 and 2. For PENTA sites, adherence will be determined by the Self-Completion Adherence Questionnaire.

(14) Neuropsych assessments **are only for** children **enrolled** at domestic PACTG sites. **Assessments are required at indicated time points for subjects on both first-line and second-line therapy. The neuropsychological testing schedule is based on enrollment date and does not change if therapy is switched.**

(15) For children <3 years of age at study entry, any assessment that included the Bayley, and was done under clinical care within 20 weeks before the Entry visit, will be acceptable. For children ≥ 3 years of age at study entry, any assessment that included the WPPSI-III, WISC-III, or WAIS-III, and was done under clinical care within 20 weeks before the Entry visit, will be acceptable. However, if the test results found the child neurologically unstable, a new neuropsych assessment must be performed at the Entry visit. **If a new neuropsych assessment is necessary based on the above criteria, it must be completed within +/- 30 days of the Entry visit.**

APPENDIX I (Cont)

(16) The neuropsych assessment will be repeated at Week 24 for children who are <6 years of age at the initial assessment. Children who are ≥6 years of age at the time of assessment will have neurodevelopment testing every 48 weeks. Children who are ≥6 years of age, after week 48 assessment, will have neurodevelopment assessment testing every 96 weeks. (See Appendix XIII). For children <6 years of age at the time of the actual study visit, any neuropsych testing performed within 8 weeks **of** the actual visit will be acceptable. For children ≥ 6 years of age at the time of the actual study visit, any neuropsych testing performed within 16 weeks **of** the actual visit will be acceptable. The interval between any two consecutive neuropsych evaluations should be at least 20 weeks.

(17) Any child enrolled in the study under Version 1.0 who was assessed with the WPPSI-R, the CPRS-48, or the CES-D test should continue being evaluated with same test for the rest of the study, as long as it is age appropriate. Otherwise, the new guidelines should be used. All other tests should be changed to the evaluations in Appendix XIII.

(18) Yearly testing for children ≤ 3 years age and every other year for children >3 years of age **at the time of testing**.

(19) The screening visit should be completed within 14 days before randomization (samples can be taken as early as 24 hours prior to study entry visit).

(20) For PACTG sites, the Entry visit for initial therapy must be completed within 72 hours of randomization, and the study regimen must also **be started** within 72 hours of randomization. For PENTA sites, the **Entry** visit for initial therapy must be completed within 72 hours of randomization; however, the study regimen may start within two weeks of randomization.

(21) From **the** Week 4 study visit on, a 2 week window (+/-1 week) for the study visit will be allowed (e.g. Week 4 visit could be at Week 3, 4, or 5).

(22) All children will remain in the study until the last child enrolled into PENPACT 1 has reached 204 weeks on treatment from his/her original randomization. This last patient could be from a PACTG or PENTA site. Thus, the study visit may be longer than 360 weeks for the early enrollees. After subjects have initiated second-line therapy, the schedule for study visits re-starts at the Entry visit as per **first-line** therapy.

NOTES:

- For Screening and Entry visits, all necessary blood samples must be collected for all laboratory tests and for storage.
- For insufficient blood draws at all other study visits, priorities are as follows: (1) RNA **(particularly aliquots at key time points noted in footnote 9),** (2) hematology, (3) chemistries, (4) plasma for storage, and (5) lymphocyte subsets.
- When subjects stop initial or second-line therapy, all clinical, virology, and immunology evaluations should be performed. Furthermore, when subjects start second-line therapy after more than two weeks of stopping initial therapy, all of these evaluations should be performed again.

APPENDIX II

GUIDELINES FOR SWITCHING THERAPY FOR PACTG AND PENTA
FLOWCHART 1

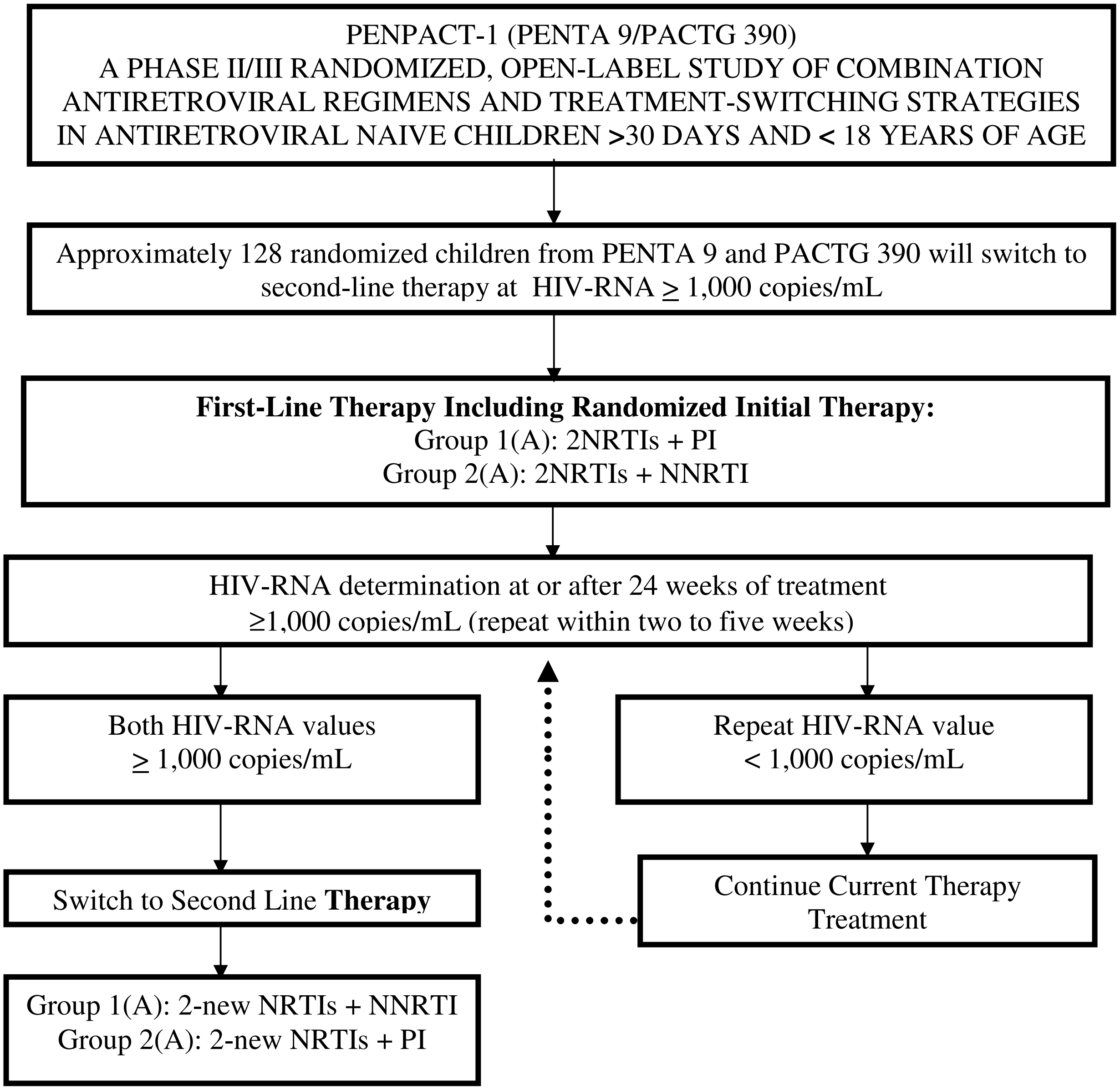

APPENDIX II (Cont)

GUIDELINES FOR SWITCHING THERAPY FOR PACTG AND PENTA
FLOWCHART 2

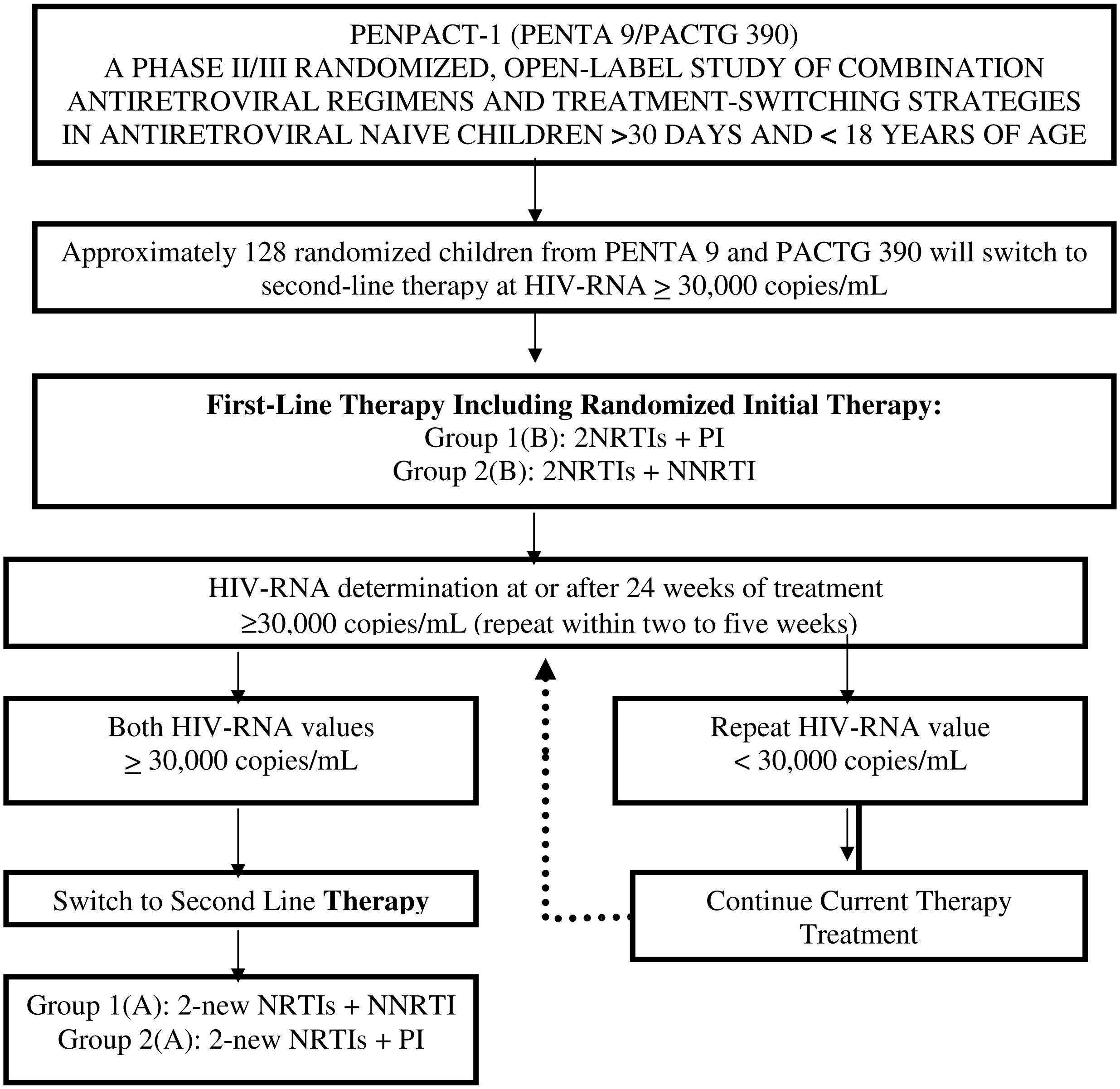

APPENDIX III
PENTA 9--PENPACT 1-B SUB-STUDY ON LIPODYSTROPHY SYNDROME AND METABOLIC ABNORMALITIES

APPENDIX III CONSISTS OF THE
FOLLOWING SELF-CONTAINED
30-PAGE DOCUMENT

APPENDIX III (Cont.)

PENPACT-1B Sub-Study:

A RANDOMISED, OPEN-LABEL STUDY
OF COMBINATION ANTIRETROVIRAL REGIMENS
AND TREATMENT-SWITCHING STRATEGIES
IN ANTIRETROVIRAL NAIVE CHILDREN ≤ 18 YEARS OF AGE

Sub-study on the lipodystrophy syndrome and metabolic abnormalities

APPENDIX III (Cont.)

PENPACT 1-B: Sub-study on the lipodystrophy syndrome and metabolic abnormalities

General Information:

This document describes a PENTA sub-study of PENPACT1 and provides information about procedures to be performed for children entered into it. Clinical problems relating to this study should be referred to the Investigators below.

Investigators responsible for the sub-study

Dr Claire Levy Marchal	clairelm@idf.inserm.fr
Hôpital Robert Debré	
48 Boulevard Sérurier	
75019 Paris	Tel: +33 1 4003 1901
France	Fax: +33 1 4003 1925
Dr Alessandra Viganò	alevig@mailserver.unimi.it
Clinica Pediatrica	
via GB Grassi, 72	
20157 Milano	Tel: +39 02 3904 2254
Italy	Fax: +39 02 3904 2253

National Trials Centres

Dr Alex Compagnucci	a.compagnucci@vjf.inserm.fr
INSERM SC10, HIV Clinical Trials Centre	
16 Avenue Paul Vaillant Couturier	
Villejuif Cedex, 94807	Tel: +33 1 4 559 5290
France	Fax: +33 1 4 559 5180
Lynda Harper	lynda.harper@ctu.mrc.ac.uk
MRC Clinical Trials Unit	
222 Euston Road	
London, NW1 2DA	Tel: +44 20 7670 4791
UK	Fax: +44 20 7670 4818

Statistician

Dr Sarah Walker	sarah.walker@ctu.mrc.ac.uk
MRC Clinical Trials Unit	
222 Euston Road	
London, NW1 2DA	Tel: +44 20 7670 4726
UK	Fax: +44 20 7670 4818

APPENDIX III (Cont.)

CONTENTS

1. Background 5
  1.1 Introduction 5
  1.2 Rationale 6
2. Objectives 6
  2.1 Primary objectives 6
  2.2 Secondary objectives 6
3. Sub-study Participants 6
4. Assessments and Procedures 8
  4.1 Flow chart/ Schedule for follow-up 8
  4.2 Key measures 9
  4.3 Fasting 9
5. Quality Assurance and Quality Control 10
6. Regulatory and Ethics Approval 10
7. Finance 10
8. Statistical analyses 10
9. References 11

Appendices

APPENDIX III/1 PATIENT INFORMATION SHEET
APPENDIX III/2 INFORMED CONSENT
APPENDIX III/3 MEASUREMENT OF BODY CIRCUMFERENCES
APPENDIX III/4 MEASUREMENT OF SKINFOLD THICKNESSES
APPENDIX III/5 STANDARDISED DEXA AND MRI ASSESSMENT
APPENDIX III/6 MEASUREMENT OF HEIGHT AND WEIGHT
APPENDIX III/7 PHOTOGRAPHS
APPENDIX III/8 CASE REPORT FORMS

APPENDIX III (Cont.)

Background

1.1 Introduction

Since 1998 a newly described syndrome has emerged in HIV-infected patients treated by antiretroviral therapy (ART) [1-3]. Although a case definition has not been formally agreed, the lipodystrophy syndrome encompasses a variety of abnormalities including:

- abnormal distribution of adipose subcutaneous tissue with the development of abdominal adiposity and peripheral wasting
- changes in glucose tolerance with insulin-resistance and severe dyslipidaemia.

The role of the different antiretroviral drug classes is still a matter of debate. Although it seems likely that treatment strategies such as 3 or 4 drug Antiretroviral Therapy (ART) contribute to the development of the syndrome, in adults non drug related host and disease factors have also been identified to be important in both fat accumulation and fat loss [4,5]. Whilst a number of studies have reported the use and duration of either protease inhibitors (PI) and/or nucleoside analogue reverse transcriptase inhibitors (NRTI) to be associated with lipodystrophy [2,6,7], whether these drugs should be regarded as promoting factors or direct toxic agents remains to be clarified.

One of the difficulties is assessing causal factors is that the lipodystrophy syndrome has been mostly studied through cross-sectional studies of patients with different duration of infection and treatment regimens. Prevalence of fat redistribution of up to 60% of adults on 3 or 4 DRUG ART has been reported, although the frequencies vary enormously with the study population. In adults under treatment for at least 2 years prevalences of around 40-50% have been reported in adults receiving PI-based ART compared with around 25-30% of those not receiving non-PI based ART, and around 5% in naive persons. Clearly, the lack of a validated case definition hampers comparison across different studies. Further, assessment of determinants of lipodystrophy is complicated by the different methodologies and scales of severity used to assess its components across studies.

In children, the European Collaborative Study estimated a 28% prevalence of fat redistribution in 374 children based on clinical report [8]. Similar prevalences have been reported in 49 children in Spain based on dual X-ray absorptiometry (DEXA) (24%) [9]. The systematic observation of 128 children at Hôpital Necker suggested that the lipodystrophy syndrome occurred as frequently in children as in adults (50%), the major difference being that all symptoms were observed with a lesser degree of severity (Levy Marchal, personal communication). Both clinical lipodystrophy and metabolic abnormalities, primarily insulin-resistance, have been reported to worsen at puberty.

The first lipodystrophy case definition study has recently been completed (the HIV LD Case Definition Study) [10]. It identified 10 parameters (measured locally) which could diagnose lipodystrophy in adults with 79% sensitivity and 80% specificity; age, gender, HIV duration, CDC stage, waist:hip ratio, anion gap, HDL cholesterol, trunk:peripheral fat ratio and leg fat percentage on DEXA, and visceral:subcutaneous abdominal fat ratio (VAT:SAT) by CT at the L4 level. Waist:hip ratio is not thought useful in children because of differing body shapes. MRI is preferred to CT in children due to the radiation dose.

APPENDIX III (Cont.)

1.2 Rationale

Within PENPACT-1, the longitudinal observation of a group of naïve HIV-infected children for 4 years after initiation of ART represents a powerful and unique opportunity to describe the development of the lipodystrophy syndrome in children and to clarify the role of antiretroviral drug classes.

Although the proposed adult case definition includes measurements based on both DEXA and CT [10], work refining a clinical definition is continuing. Systematic documentation of clinical features of paediatric lipodystrophy will enable comparisons to be made with future clinical adult definitions.

Objectives

2.1 Primary objectives

The primary objective of the sub-study is to document the development and the progression of the lipodystrophy syndrome in the European children included in PENPACT-1, considering body fat redistribution, dyslipidaemia and insulin-resistance.

The specific objectives are

- to calculate the incidence of lipodystrophy syndrome in ART-naïve children initiating 3 or 4 drug ART
- to compare incidence rates between children receiving PI versus NNRTI based ART as their first regimen (both on a dual NRTI backbone)

2.2 Secondary objectives

Secondary objectives are

- to determine the host and disease-related factors influencing the progression rate of fat redistribution and metabolic changes jointly and separately in children, with particular interest in the role of puberty
- to assess the role of different approaches (questionnaires, circumferences, skinfolds and clinical investigations) in the evaluation of lipodystrophy in children

Sub-study Participants

All children aged 30 days to 18 years enrolled in the European centres of PENPACT-1 are eligible for this sub-study.

Clinicians are strongly encouraged to enrol all children entering PENPACT-1 into the minimal investigations part of the lipodystrophy sub-study (see sections 4.1 and 4.2). At baseline and then every 24 weeks, this will involve a questionnaire for the carer and physician; waist, mid-arm and mid-thigh circumferences; and lipids (including fractions wherever possible) and glucose. Metabolic measurements should be taken fasted wherever possible.

APPENDIX III (Cont.)

Where a clinical centre is able to commit to more intensive investigations, all children from this centre enrolled in PENPACT-1 should be included in the maximal investigations part of the lipodystrophy sub-study; which, in addition to the measurements detailed above, includes 4 skinfold measurements at baseline and each 24-weekly visit (see sections 4.1 and 4.2). Centres should ensure that there is one or more members of staff with an active interest in performing these skinfold thicknesses, with the intention of carrying out all longitudinal measurements in this PENPACT-1 substudy.

Finally, L4 MRI and/or DEXA should be performed at baseline and then annually where feasible on an individual child basis, for children enrolled in either minimal or maximal parts of the sub-study. Baseline scans should be obtained if at all possible.

APPENDIX III (Cont.)

Assessments and Procedures

4.1 Flow chart/ Schedule for follow-up

Evaluation (week)	Screening (-2) or Entry (0)	24	48	72	96	then every 24 weeks	switch to 2nd line*
Key assessment stages (annual)	X		X		X		X
MINIMAL (ALL CHILDREN)							
Medical history[a]	X						
Fasting lipid profile[b]	X	X	X	X	X	X	X
Fasting plasma glucose	X	X	X	X	X	X	X
Fasting serum insulin (where possible)	X	X	X	X	X	X	X
Fasting serum storage[c]	X	X	X	X	X	X	X
Weight, height, Tanner stage[d]	X	X	X	X	X	X	X
Carer and physician assessment[e]	X	X	X	X	X	X	X
Body circumferences[f]	X	X	X	X	X	X	X
MAXIMAL (SOME CENTRES)							
Skinfold thicknesses[g]	X	X	X	X	X	X	X
WHERE POSSIBLE							
MRI L4	X		X		X	(every 48	X
DEXA	X		X		X	weeks)	X
Photographs[h]	X	X	X	X	X	X	X

* Repeat measurements at switch to second line if more than 8 weeks elapsed since last measured (repeat MRI and DEXA if more than 24 weeks since last measured).

[a] birthweight; history of diabetes (Type I and II), hypertension and cardiovascular disease in biological parents and grand-parents; presence of lipodystrophy in parents
[b] total cholesterol and triglycerides; directly measured LDL and HDL where possible
[c] 1ml serum storage where possible (3ml of whole blood in empty tube). This is in addition to plasma stored in the main PENPACT-1 trial.
[d] collected as part of the main PENPACT-1 trial
[e] including specific aspects of fat redistribution such as sunken cheeks, buffalo hump, flat buttocks, lipoatrophy in the legs and abdominal hypertrophy (Appendix 8)
[f] waist, mid-arm, mid-thigh (Appendix 3)
[g] triceps, subscapular, supra-iliac, mid-thigh (Appendix 4)
[h] photographs of lower arm, thigh, face and side profile (Appendix 7)

APPENDIX III (Cont.)

4.2 Key measures

Clinical assessments are obviously useful, but tend to be operator-dependent. In contrast, investigations such as MRI provide a quantitative assessment of both subcutaneous and visceral adipose tissue. All assessments should be performed at baseline if at all possible, particularly clinical investigations (MRI and DEXA) since any subsequent investigations should ideally be compared to pre-therapy. Photographs may provide a reference to compare against a clinical diagnosis of lipodystrophy or reported signs and symptoms, although care must be taken to standardise conditions such as lighting and distance.

As many of the individual procedures should be performed as possible, according to the order below if there are constraints of time or other resources. See Appendix 3 and 4 for details of circumference and skinfold thickness measurement respectively. Appendix 5 provides guidelines for standardised MRI and DEXA assessment. Whilst height and weight are recorded as part of the main study, standardised methods of measurement should be used wherever possible (Appendix 6). CRFs with carer and physician assessment and details of other procedures required are in Appendix 8.

1. Fasting lipid profile (total cholesterol, triglycerides, directly measured LDL and HDL) (see section 4.3)
2. Fasting plasma glucose and serum insulin (see section 4.3)
3. Waist, mid-arm and mid-thigh circumferences (Appendix 3)
4. Skinfold thicknesses (triceps, subscapular, supra-iliac, mid-thigh; Appendix 4)
5. Carer assessment (Appendix 8)
6. Physician assessment (Appendix 8)
7. MRI L4 (Appendix 5)
8. DEXA (Appendix 5)
9. Photographs (Appendix 7)

On both baseline and follow-up forms, the carer should complete their assessment of the child's body shape before all other information is completed.

Photographs should be stored in the clinic notes and not sent to the National Trials Centres with forms. If they are subsequently required for blinded central review, they must be anonymised before sending.

Demographic data is collected as part of the main PENPACT-1 trial data.

4.3 Fasting

Fasting is defined as no intake other than water for the last 6 hours (4 hours for children under 2 years). If possible, given dosing schedules, antiretroviral medication should be taken after fasting measurements have been performed. Regardless, the time of last dose of antiretroviral drugs should be recorded.

If the child is not fasted, the lipid profile and other metabolic measurements should still be performed, with time of last intake recorded.

APPENDIX III (Cont.)

Quality Assurance and Quality Control

As clinical investigations will be performed locally, it is important to record make and models of scanners and wherever possible to ensure the same machine is used at each assessment visit to ensure valid comparisons over time. Copies of electronic scan data should be retained by the clinic. Please contact Dr Alessandra Viganò if interpretation of electronic scan data is required. The necessity for centralised reading of all scan data will be considered at the end of PENPACT-1.

Regulatory and Ethics Approval

This substudy will be reviewed and approved by the relevant Ethics Committee for each participating centre before children are enroled. The patient information sheet is attached (Appendix 1). Written informed consent will be obtained from each child or their representative according to age and knowledge of HIV status (Appendix 2).

Finance

As this sub-study has not received additional funding, there is no reimbursement for any of the procedures.

Statistical analyses

There is currently no consensus definition of the lipodystrophy syndrome: however, case definition studies are ongoing [10], and any validated definition based on measurements taken in this substudy will be used in the final analysis. Otherwise, each anomaly will initially be described separately; subsequently each component of lipodystrophy (fat redistribution, metabolic abnormalities) will be considered separately with any abnormality in each component regarded as evidence of lipodystrophy.

Anthropometric measurements will be expressed in percentiles of the reference distribution for age and gender [11,12]. These values will be regarded as abnormal if $\geq 95^{th}$ percentile. Lipid measurements will be considered abnormal according to standard toxicity tables used in PENPACT-1. Insulin resistance will be estimated from fasting insulin and glucose measurements by using a homeostasis model assessment (HOMA) [13].

The effect of the following risk factors at trial entry on the incidence of lipodystrophy will be tested using standard time to event methods (logrank and Cox proportional hazards models): gender, age, BMI, family history, percentage and absolute CD4, and HIV-1 RNA. The effect of initial response to ART on subsequent development of lipodystrophy will also be considered.

APPENDIX III (Cont.)

Assuming a cumulative proportion of 40% of children in the PI containing arm develop 1 or more signs of lipodystrophy by 3 years, there would be at least 80% power to detect a proportion below 18% in the NNRTI containing arm if all 128 children were included in the sub-study. If only 80 children were included there would be at least 80% power to detect a proportion below 14% in the NNRTI containing arm.

References

1. Carr A, Samaras K, Burton S, et al. A syndrome of peripheral lipodystrophy, hyperlipidaemia and insulin resistance in patients receiving HIV protease inhibitors. *AIDS* 1998; 12:F51-8.
2. Miller KD, Jones E, Yanovski JA et al. Visceral abdominal fat accumulation with the use of indinavir. *Lancet* 1998; 351:871-875.
3. Lo JC, Mulligan K, Tai VW et al. "Buffalo hump" in men with HIV-1 infection. Lancet 1998; 351:867-70.
4. Lichtenstein KA, Ward DJ, et al. Clinical assessment of HIV-associated lipodystrophy in an ambulatory population. *AIDS* 2001;15:1389-98.
5. Lichtenstein KA, Delaney KM, Armon C, et al. Incidence of and risk factors for lipoatrophy (abnormal fat loss) in ambulatory HIV-1-infected patients. *JAIDS* 2003;32:48-56.
6. Gervasconi C, Ridolfo AL, Trifiro G, et al. Redistribution of body fat in HIV-infected women undergoing combined antiretroviral therapy. *AIDS* 1999;13:465-71.
7. Saint-Marc T, Partisani M, Poizot-Martin I, et al. Fat distribution evaluated by computed tomography and metabolic abnormalities in patients undergoing antiretroviral therapy: preliminary results of the LIPOCO study. *AIDS* 2000;14:37-49.
8. Vigano A, Thorne C for the Italian Register on HIV in Children, European Collaborative Study. Fat redistribution and metabolic abnormalities in HIV-infected children and adolescents in Europe. 10th CROI, Boston February 2003, Abstract 774.
9. Ramos JT, Garcia L, Rojo P, et al. High prevalence of metabolic abnormalities in children treated with HAART. 10th CROI, Boston February 2003, Abstract 772.
10. HIV Lipodystrophy Case Definition Study Group. An objective case definition of lipodystrophy in HIV-infected adults: a case-control study. *Lancet* 2003; 361:726-35.
11. RollandCachera MF, Brambilla P, Manzoni P, Akrout M, Sironi S, DelMaschio A, Chiumello G. Body composition assessed on the basis of arm circumference and triceps skinfold thickness: A new index validated in children by magnetic resonance imaging. Am J Clin Nutr 1997; 65:1709-13.
12. Moreno LA, Fleta J, Mur L, Rodriguez G, Sarria A, Bueno M. Waist circumference values in Spanish children - Gender related differences. Eur J Clin Nutr 1999; 53:429-433.
13. Haffner SM, Miettinen H, Stern MP. The homeostasis model in the San Antonio Heart Study. Diabetes Care 1997; 20:1087-92.

APPENDIX III (Cont.)

Appendix III/1Patient Information Sheet

THE PENPACT 1 TRIAL – LIPODYSTROPHY SUBSTUDY
An Information Sheet For Parents

WHAT IS LIPODYSTROPHY ?

Since 1998 doctors, and patients themselves, have started noting that some adults and children with HIV infection have a side-effect called lipodystrophy after being on antiretroviral treatment for a long time. This can cause them to have

- extra fat in some parts of their bodies
- loss of fat in some parts
- changes in blood results which may be linked to risk of heart disease in the future.

It is unclear which antiretroviral drugs or combination of antiretroviral drugs are causing these changes. It might be that only certain people are likely to experience them. It is also possible that puberty might make these changes worse.

WHAT ARE THE AIMS OF THE PENPACT 1 SUBSTUDY ?

The PENPACT 1 trial plans to include 256 HIV infected children who are starting taking antiretroviral therapy for the first time. Children enrolled in PENPACT 1 will be followed for at least 4 years from when they start therapy. It is extremely to important to monitor for the development of symptoms of lipodystrophy in these children. It is hoped that most of the children who join the study through PENTA (Paediatric European Network for Treatment of AIDS) will also join this lipodystrophy substudy so it will help us understand whether some drugs are more likely to cause lipodystrophy and whether certain children are more likely to suffer from it. This will help doctors know which are the safest antiretroviral drugs to give children in the future.

Most of the children enrolled in PENPACT 1 in the USA will also join a similar, but separate, substudy.

WHAT WILL JOINING THE SUBSTUDY MEAN FOR MY CHILD ?

As it is not yet clear which are the best ways of detecting lipodystrophy, a number of extra tests need to be performed. We will measure weight and height and the circumference of arms, waist and thighs on all children at the time of joining the PENPACT 1 trial and then every 6 months. Your child's doctor will ask you a few questions about any changes you might have noticed. We will also take a little extra blood (3ml) for further investigations to understand lipodystrophy.

It is easier to interpret the results of these blood tests when no food has been taken (fasting) so if possible your child should not eat or drink anything other than water in the 6 hours before coming up to clinic. If your child is under 2, they only need to fast for 4 hours.

We would also like to be able to ask you whether any of your child's family have suffered from diabetes or heart disease to see if this increases the chances of your child getting lipodystrophy.

APPENDIX III (Cont.)

In some clinics we are able to perform some further investigations and your child's doctor may discuss the possibility of performing these investigations with you. These are:

Measurement of skinfold thickness: this is a way of assessing body fat. Calipers are used to measure skinfold thickness in the top part of the arm, the thigh and above and below the shoulder blade.
Some younger children may be a little wary of their caliper measurements, however we do not expect them to cause any discomfort. Your doctor will not continue with any measurements if your child becomes distressed.

Photographs: as lipodystrophy can be difficult to diagnose from clinical symptoms, it will be very useful to have photographs for reference. Your child will not be identifiable in the photograph.

Imaging tests such as DEXA scan or MRI provide the best ways of looking at fat under the skin. These investigations will only be performed at the start of the trial then every 12 months. Your doctor will give you the information sheet from the radiology department in your hospital if these tests are available.

CONFIDENTIALITY

All information collected about your child during PENPACT 1 will be confidential. Names will not be used on any data collection forms, samples or photographs. Your child will be identified by a study number only.

PARTICIPATION

Participation in this substudy is entirely voluntary. If you decide that you do not wish your child to take part in the substudy, or in certain parts of the substudy, that is entirely your right. Your decision will in no way affect any present or future treatment for your child.
You can withdraw your child from the substudy at any time without giving a reason. This will not affect your child's medical care.

WHAT ELSE DO I NEED TO KNOW ?

The Ethics Committee of your hospital has given approval for this substudy to be conducted at your clinic. PENTA, which is funded by the European Union, has made agreements for compensation should your child come to any harm during the trial. Your doctor will be able to tell you about this.

Thank you for taking time to consider this substudy for your child. Please ask any questions and let us know if there are things that you do not understand, or would like more information about.

APPENDIX III (Cont.)

Appendix III/2 **Informed Consent**

ISRCTN 73318385

PENPACT 1 LIPODYSTROPHY SUBSTUDY – CONSENT

Form L0
September 2003

Please give all dates as dd/mm/yy

Initials: |||	Date of Birth:	Trial Number:

Please initial (or mark) box if you agree:

1. I have read the information sheet for the PENPACT1 lipodystrophy substudy dated ____________ and I understand what will be required if my child participates in this study. ☐

2. I agree for my child to be enrolled in the PENPACT 1 substudy and for an additional sample of blood (3ml) to be stored each 6 months. Yes ~ No ~

Consent for the following is ***not required*** *for entry into the lipodystrophy substudy: a decision not to consent will not have an adverse effect on your child's care.*

3. I agree that information on presence or absence of lipodystrophy in the child's biological parents may be requested and stored as part of the lipodystrophy substudy. Yes ~ No ~

Your doctor will let you know which of the following are possible, with your consent, in this clinic.

4. I agree for skinfold thicknesses to be measured on my child. Yes ~ No ~

5. I agree that photographs of my child's lower arm, thigh, face and side profile may be taken during the lipodystrophy substudy. I agree to subsequent storage of these photographs identified only by the PenPact1 trial number. Yes ~ No ~

6. I have read the information sheet for MRI and DEXA scans, and I agree that MRI scans may be performed on my child during the lipodystrophy substudy. Yes ~ No ~

7. I have read the information sheet for MRI and DEXA scans, and I agree that DEXA scans may be performed on my child during the lipodystrophy substudy. Yes ~ No ~

PARENT or LEGAL GUARDIAN

SIGNED: ________________________ (PRINT NAME): ________________ DATE: ___/___/___

INVESTIGATOR

SIGNED: ________________________ POSITION: ________________ DATE: ___/___/___

(PRINT NAME): ____________________ CENTRE: ______________________________

IMPORTANT: One signed original to be given to parent/legal guardian
One signed original to be kept in the PENPACT 1 file
One signed original to be kept in the clinic notes

Appendix III/3Measurement of Body Circumferences

APPENDIX III (Cont.)

In order to reduce inter-operator and inter-visit variability, a standardised procedure with pre-specified positions for measurement and multiple measurements should be followed.

1. Child should be dressed in underwear, socks, and a hospital gown; all outer clothing should be removed.
2. Use nonstretchable, cloth or vinyl measuring tape that measures in centimeters or millimeters and is at least one half inch in width.
3. Make sure the tape does not compress the tissues during the measurement.
4. Measuring tape should always be read at eye level.
5. All measurements should be made in triplicate and all values recorded.

Waist Circumference:

6. The child should be standing erect but relaxed.
7. Ask the child not to try to hold in their stomach during the measurements.
8. All measurements should be made after child has exhaled.
9. The usual method is to measure the smallest circumference around the waist. However, this measurement is not sufficient in individuals with increased abdominal girth. Therefore, place the measuring tape around the child at the level of the navel, holding the tape horizontal to the floor (the umbilicus waist).
10. Record measurement in cm to the nearest mm.
11. Repeat the circumference measurement twice more and record. In all, the measurement should be made 3 times.

Midarm Circumference:

12. All measurements should be performed on the right arm unless there is a specific reason why this is not possible. At the time of the first measurement, note the side used in the notes and use the same side for all subsequent measurements. To correctly locate the midarm region, upper arm length should first be measured.
13. Ask the child to bend the arm at right angle with the palm facing upward.
14. Locate the acromial process on shoulder blade. It may help to slide your fingers along the clavicle to find the acromial process.
15. Locate the olecranon process, which is the tip of the elbow.
16. Using a measuring tape, measure down the posterior aspect of the arm between these two points, being careful to keep the tape straight by holding it slightly away from the two end points if necessary.
17. Divide the length by 2, and mark this midpoint on the arm with a pen.
18. Ask the child to relax the arm at his/her side with the palm facing inward. Make certain that the child is not flexing the muscles in the arm.
19. Place the measuring tape around the arm at this midpoint, holding the tape horizontal to the floor (and, therefore, perpendicular to the length of the arm).
20. The tape should be touching the skin continuously and should follow the contours of the tissue (i.e., no gaps), but it should not compress the skin or tissue.
21. Record measurement in cm to the nearest mm.
22. Repeat the circumference measurement twice more and record. In all, the measurement should be made 3 times.

APPENDIX III (Cont.)

Midthigh Circumference:

23. As is the case with the midarm circumference, this measurement should always be made on the right side, unless doing so is impossible. To correctly locate the midthigh region, upper leg length should first be measured.
24. Ask the child to sit on a chair, exam table, or bed, with the knee bent at a 90° angle.
25. Locate the midpoint of the upper border of the patella (kneecap).
26. Locate the inguinal crease, just below the anterior superior iliac spine. An easily identifiable landmark is the tendon that moves when the leg is flexed upward slightly.
27. Measure the length between these two marks.
28. Divide the length by 2, and mark this midpoint on the top of the thigh, making sure the tape measure remains straight.
29. Ask the child to stand, with the foot of the right leg slightly forward from that of the left leg. The knee of the right leg should be flexed slightly, and all of the weight should be on the left leg.
30. Ask the child not to flex the muscles in the thigh.
31. Measure circumference across the midpoint, holding the tape perpendicular to the length of the thigh.
32. Record measurement in cm to the nearest mm.
33. Repeat the circumference measurement twice more and record. In all, the measurement should be made 3 times.

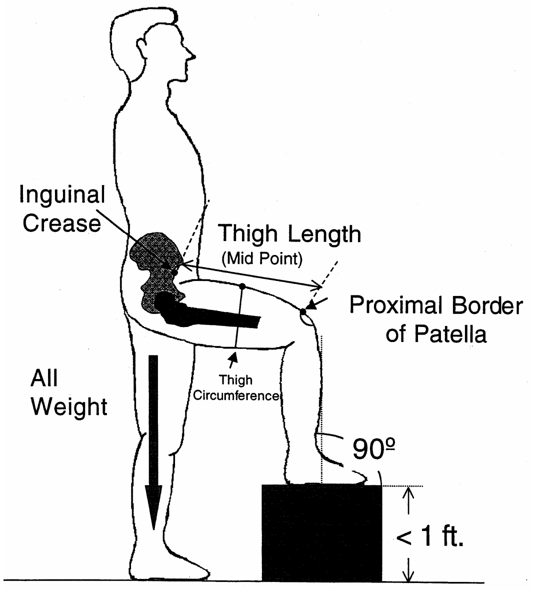

APPENDIX III (Cont.)

Appendix III/4Measurement of Skinfold Thicknesses

In order to reduce inter-operator and inter-visit variability, a standardised procedure following Loeman et al in the *Anthropometric Standardization Manual* with pre-specified positions for callipers and multiple measurements should be followed. Holtain callipers provided by PENTA for PenPact-1 should be used.

1. Child should be dressed in underwear, socks, and a hospital gown where appropriate; all outer clothing should be removed.
2. All measurements should be performed on the right side unless there is a specific reason why this is not possible. At the time of the first measurement, note the side used in the source document and use the same side for all subsequent measurements.
3. All measurements should be made in triplicate in the order below and all values recorded.

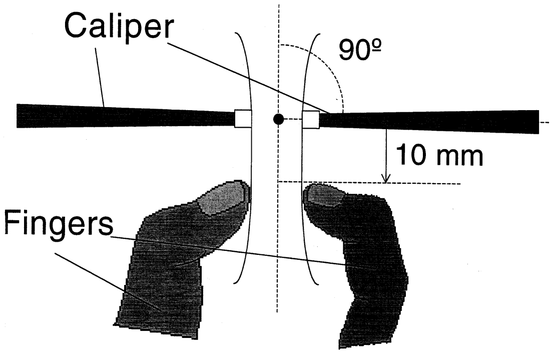

Triceps:

4. The child should be standing erect but relaxed.
5. The site of the triceps skinfold is located in the midline of the posterior aspect of the arm while the elbow is flexed to 90° and the palm is facing upwards, over the triceps muscle, at a point midway between the lateral projection of the acromion process of the scapula and the inferior margin of the oclecranon process of the ulna (see below).
6. The tape is placed with its zero mark on the acromion and stretched along the upper arm, extending below the elbow.
7. The midpoint is marked on the lateral side.
8. The skinfold is measured with the arm hanging loosely and comfortably at the child's side and palm facing the thigh. The site of measurement must be in the midline posteriorly.
9. The measurer stands behind the child and places the palm of his/her hand on the child's arm proximal to the marked level, with the thumb and finger directed inferiorly.
10. The triceps skinfold is picked up with the thumb and index finger, approximately 1cm proximal to the marked level, and the tips of the calipers are applied to the skinfolds at the marked level. The skinfold must be parallel to the long axis of the upper arm and the caliper must be applied at the mark 90° to the long axis of the upper arm (see below).
11. Record the measurement in cm to the nearest mm.
12. Repeat the measurement twice more, pausing each time to prevent sustained compression of the tissue and record all three measurements.

APPENDIX III (Cont.)

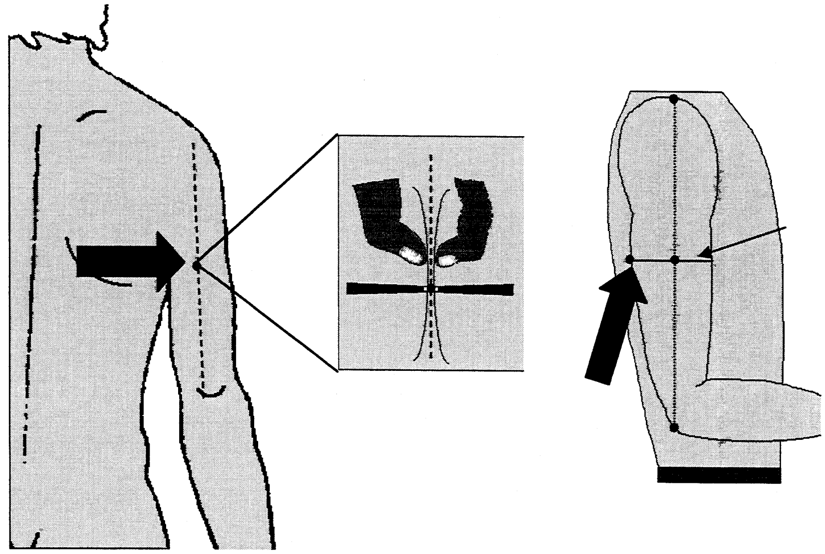

Subscapular:

13. The child should be standing erect with upper extremities relaxed at the side of the body.
14. The subscapular skinfold is picked up on a diagonal, inclined infero-laterally approximately 45° to the spine in the natural cleavage lines of the skin (see above). The site is just inferior to the inferior angle of the scapula.
15. To locate the site, the measurer palpates the scapular (shoulder blade), running the fingers inferiorly and laterally along its vertebral border until the inferior angle is identified.
16. The caliper jaws are applied 1cm infero-lateral to the thumb and finger raising the fold.
17. Record the measurement in cm to the nearest mm.
18. Repeat the measurement twice more, pausing each time to prevent sustained compression of the tissue and record all three measurements.

APPENDIX III (Cont.)

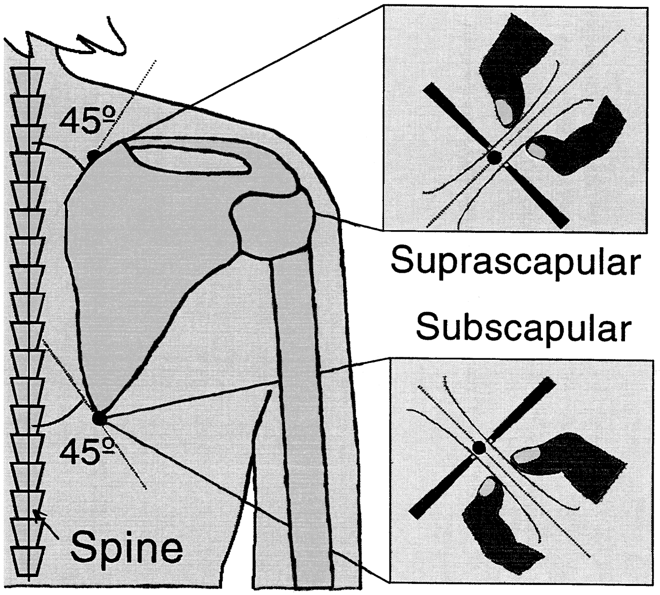

APPENDIX III (Cont.)

Supra-iliac:

19. The child should be standing erect with body weight evenly distributed on both feet.
20. The child should relax the abdominal wall musculature as much as possible during the procedure and breath normally.
21. The child may be asked to hold their breath near the end of expiration if there is substantial movement of the abdominal wall with normal respiration.
22. Select a site 5cm below the midpoint of the umbilicus and 3cm to the right (see below).
23. Raise a horizontal skinfold with one hand and measure its thickness.
24. Record the measurement in cm to the nearest mm.
25. Repeat the measurement twice more, pausing each time to prevent sustained compression of the tissue and record all three measurements.

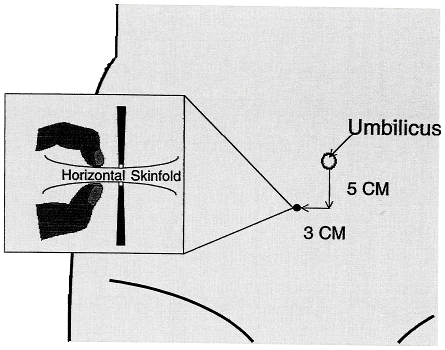

Thigh:

26. The child should be standing with the body weight shifted to the left foot while the right leg is relaxed with the knee flexed and the right foot is flat on a stool or bench less than 30cm in height.
27. If maintaining balance is a problem, the child holds the top of the measurer's shoulder, a countertop, or a high-backed chair.
28. The child flexes the hip and knee to assist location of the inguinal crease and the proximal border of the patella.
29. The thigh skinfold is located in the midline of the anterior aspect of the thigh, midway between the inguinal crease and the proximal border of the patella.
30. The thickness of a vertical fold is measured while the participant is still in the position above.
31. The caliper jaws are applied about 1cm distal to the fingers holding the fold (see below).
32. Record the measurement in cm to the nearest mm.
33. Repeat the measurement twice more, pausing each time to prevent sustained compression of the tissue and record all three measurements.

APPENDIX III (Cont.)

APPENDIX III/5 Standardised DEXA and MRI assessment

Procedures for Performing DEXA Scans

The following is a summary of procedures. Please follow the manufacturer's instructions for operating your instrument. Total body scans should be done using standard operating procedures defined by the manufacturer in order to allow comparability between study sites as well as generalisability to children outside of the study conditions.

The child should be questioned and examined for metal that could be in the scan path. Typical things to look for are earrings, spectacles, wristwatches, coins, rings, buttons, buckles, zippers, and support braces. The child should remove shoes and it may be necessary to remove skirts, trousers, etc. If in doubt, it is best to remove the object in question. If clothes are removed, a child gown will be provided and a sheet available to place over the child during the scan. Rings that cannot be removed can be left on, but should be noted and should always be left on in subsequent scans. Small objects removed from the child should be placed in a small box to keep them together and the box left in the room with the child. After the scan is performed the objects should be returned to the child and the child queried to make sure s/he has received back all objects.

Child Positioning Techniques and Whole Body Scan Acquisition

For any system, the calibration procedure and quality control checks should be performed daily. Use the recommended "whole body scan" setting of the particular DEXA machine available, depending on the size of the child.

1. When prompted by the program, ask the child to lie down on the scan table.
2. The centerline on the table pad should be down the middle of the child's body (to divide the child's body in half). Use the lines on the table pad to ensure the child is lying straight on the table. The child's head should be directly below (about 1") the horizontal line located along the top of the table pad.
3. Have the child tilt his/her head back slightly during the scan. This makes placement of the head region cut line easier to place.
4. A foam wedge (or pillow) should not be used under the child's head, as it will affect the body composition results.
5. To measure the child's thickness (sagittal diameter) while he/she is lying down, identify the umbilicus, and measure the distance from the plane of the top of the abdomen to the table using a 1-meter ruler. Measurement should be recorded to the nearest millimeter. Place a flat Plexiglas sheet on the abdomen, parallel to the table, and place the ruler at the edge of the Plexiglas. This will facilitate identification of the plane of the umbilicus. The distance between the Plexiglas and the table is then read directly from the ruler. Be sure the ruler is straight perpendicular to the table, and that the Plexiglas sheet is flat and parallel with the table.
6. Have the child place his/her hands palms down alongside the body with fingers together. DO NOT OVERLAP HANDS AND LEGS. The child's entire body should fit within the scan lines on the table pad.
7. The child's feet should be held together using a Velcro strap, and the child asked not to move until directed to do so. Feet should be held maximally dorsiflexed for consistency.
9. Continuing the scan will cause the x-ray tube to ramp up to the appropriate current and voltage. The operator should check to make sure the orange "X-Ray On" light is lit, and remain in the room to check the progress of the scan acquisition as it appears on the screen.

APPENDIX III (Cont.)

10. The child's head should appear on the screen with a few blank scan lines above it. As the scan proceeds, the total-body image should be in a straight line vertically on the screen. If these conditions are not met, the scan should be stopped. The scan arm will move to the original start position and the localizer light will come on. The child should be repositioned as needed.
11. When the detector goes past the child's feet, the auto stop feature will interrupt the scan and close the shutter. A message will appear on the screen, allowing the operator to continue the scan or shut down the system. After the scan ends the shutter will close, the voltage and current ramp down, and a messages appear for the operator to wait. The scan arm will move to the home position and a screen message will appear to inform the operator that the scan is over and to remove the child from the table. The technician should go to the Total-Body Scan Options screen and save the scan file.
12. The operator may either analyze the scan file or exit the Total-Body portion of the program to do another type of scan.

IMPORTANT NOTE: MAKE SURE TO SAVE THE SCAN FILE TO THE HARD DRIVE AND/OR A DISC.

Analysis of DEXA Scan

Positioning of cuts is the same regardless of the guidelines for scan analysis for different machines. Note that the cuts used are not the same as those utilised for bone density. When drawing regions try to ensure correct allocation of soft tissue. It is easier to position the cuts to include soft tissue if the grey scale is adjusted and a combined bone and soft tissue display is used. Soft tissue cuts are as follows

1. Arm: The cut should be positioned so it passes through the centre of the arm socket. It should be as close to the body as possible, without touching the ribs, pelvis or greater trochanter. The arm should be included in the cut.
2. Rib: Position the cut as close to the spine as possible, without including spine data in the rib region.
3. Centre: divide the body lengthways and position the cut such that it passes though the centre of the body.
4. Pelvis: position the cut such that the top horizontal line is just above the top of the pelvis without touching it. The two angled lines bisect the femoral necks.
5. Dorsal: position the cut in T12-L1 disc space. T12 may be identified by the rib extending from it.

The following data are required for the each of the total body, the arms, legs and trunk: fat kg and percentage, lean mass kg and percentage.

Procedures for Performing L4 MRI Scans

Single cut abdominal MRI at the mid L4 level must be performed using a manual tracing method to estimate visceral abdominal (cm^2) and subcutaneous abdominal fat (cm^2).

1. Place the child in a supine position with their head straight, shoulders relaxed and arms raised above the head. Movement of the child should be minimal throughout the procedure. All soft tissue must be included in the MRI field of view.
2. Place a metallic marker at the level of the mid L4 and take a scout film of the area.

APPENDIX III (Cont.)

3. Using the scout film as a guide, take a single 10mm thick axial slice of the abdomen through the point of the marker, using a normal setting.
4. Use the trace cursor to trace out the intra-abdominal fat and obtain the area. This may require 3 or 4 attempts to ensure all the fat "pools" are included. Add these areas together to obtain the visceral abdominal fat measurement (VAT) in cm^2.
5. Magnify the image to an appropriate size on the monitor, and using the cursor, measure the area of subcutaneous fat (SAT) in cm^2.

IMPORTANT NOTE: MAKE SURE TO SAVE THE SCAN FILE TO THE HARD DRIVE AND/OR A DISC.

APPENDIX III (Cont.)

APPENDIX III/6 Measurement of height and weight

Height

1. This measurement should be performed carefully using a stadiometer, a measuring rod that is attached to many physicians' scales, or other device that is carefully mounted and maintained throughout the study. If no designated equipment is available for measuring height, a tape measure or series of yardsticks could be carefully attached to a wall, with the zero end just touching the floor.
2. Shoes should be removed before height is measured.
3. For wall-mounted measuring devices, the child should stand with his/her back to the device. The child should be standing with the device centered down the middle of the body and should be standing with heels together and heels, buttocks, and shoulders touching the wall. The child should tuck his/her chin down into the chest and stand as tall as possible. If the measuring device has a horizontal bar to assist with the measurement, the bar should be raised above the child's head and lowered until it just touches the head (the skull; not just the hair). It is important to make certain that the bar is completely horizontal. If the bar is at an angle greater or less than 90° to the wall, the measurement of height will be inaccurate.
4. If the measuring device does not have a horizontal bar, an alternative device should be constructed to make certain that height is recorded from the point on the measuring device that is exactly horizontal to the top of the center of the head. A plastic right triangle or two pieces of wood attached at a 90_ angle could serve this purpose.
5. Height should be reported in cm to the nearest mm.

Weight:

NOTE: The same scale should be used for all measurements performed for this substudy. The scale should be calibrated monthly. (Instructions for calibration are listed below).

1. Children should be weighed while wearing only a hospital gown, underwear, and socks. All other clothing, including shoes, should be removed.
2. Children should be asked to void before weight is measured.
3. Children should not engage in strenuous exercise for 8 hours preceding the measurements, because of its potential effect on hydration status.
4. Before the child is weighed, make certain that the scale is in balance if it is a beam-balance scale or reads zero if it is an electronic scale.
5. Instruct the child to stand with both feet centered on the scale with arms at the sides. The child should not move or hold onto anything during the measurement.
6. Allow the scale to stabilize and record the weight in kg to the nearest 0.1kg.

Standardisation of Weight Scales

1. The scales should be standardised monthly. Procedures for standardisation are described below.
2. Scales should be standardised before the first child is screened and monthly throughout the study. To standardise, select an object or objects whose combined weight is at least 50 lbs. or 23 kg and that will be available, unaltered, throughout the study. (Example of objects might be standard weights, which might be available through your institution’s engineering facility, weights used with exercise equipment, or simply gallon bottles filled with water and well-sealed.

APPENDIX III (Cont.)

3. Zero or balance the scale.
4. Place the object or objects to be weighed on the center of the scale and record the reading.
5. Remove the object or objects; re-zero or rebalance the scale and repeat the weighing procedure two more times. Total trials: three.
6. If the weights differ by more than 0.5 lb. or 0.2 kg from each other or from baseline values, notify the protocol team and arrange for a technician to service the scale.
7. No other adjustments should be made to the scale during the study period.

APPENDIX III (Cont.)

Appendix III/7Photographs

In order to reduce inter-visit variability, a standardised procedure with pre-specified positions for photographs should be followed. Ideally all photographs should be taken under the same lighting conditions (for example, in the same clinic rooms).

Lower arm

1. All photographs should be taken of the right arm unless there is a specific reason why this is not possible. At the time of the first photograph, note the side used in the notes and use the same side for all subsequent photograph.
2. The child should sit at a table or desk and extend their lower arm palm down on the table, with their upper arm at a 90º angle.
3. Photograph the child's arm looking straight down through the camera from a distance of approximately 70cm, with the arm from wrist to elbow in centre of view.
4. The child should then turn their arm over, palm up, for a second photograph at the same distance.

Thigh

5. All photographs should be taken of the right thigh unless there is a specific reason why this is not possible. At the time of the first photograph, note the side used in the notes and use the same side for all subsequent photograph.
6. The child should stand straight with their back against a wall.
7. Photograph the child's thigh looking horizontally across through the camera from a distance of approximately 1m, with the leg from knee upwards in centre of view.

Face

8. The child should remain standing straight with their back against a wall.
9. Photograph the child's face looking horizontally across through the camera from a distance of approximately 1m, with nose in centre of view.

Side profile

10. Whilst remaining by the wall, the child should turn to their left so that their left shoulder and left leg is against the wall, standing straight.
11. Photograph the child's side profile looking horizontally across through the camera to their waist from a distance of approximately 3m, with waist in centre of view.

APPENDIX III (Cont.)

Appendix III/8Case Report Forms

On both baseline and follow-up forms, the carer should complete their assessment of the child's body shape before all other information is completed.

Form 1	Baseline
Form 2	Follow-up

APPENDIX III (Cont.)

PENPACT1 PENTA9 PENPACT1 PENTA9 PENPACT1 PENTA9

ISRCTN 73318385

Week no. ______

PENPACT 1 LIPODYSTROPHY SUBSTUDY – BASELINE (WEEK –2 OR 0)

Form L1
March 2003

Please give all dates as dd/mm/yy

**Clinic Number:**	**Date of Birth:**	**Trial Number:**
**Initials:** ☐ ☐ ☐	**Date of Assessment:**	

1. **What was the child's birthweight** (kg) ____________ **and gestational age** (weeks) ____________

2. **Has consent been given for information on family medical history to be collected? Yes** ☐ **No** ☐
If Yes, **have either of the child's parents, or any of the child's grandparents suffered from the following conditions?**

**Condition**	**Biological Mother** No	Yes	Not known	**Biological Father** No	Yes	Not known	**Grandparents** No	Yes	Not known	If yes, specify which grandparent:
Type I diabetes	☐	☐	☐	☐	☐	☐	☐	☐	☐	
Type II diabetes	☐	☐	☐	☐	☐	☐	☐	☐	☐	
Hypertension	☐	☐	☐	☐	☐	☐	☐	☐	☐	
Stroke	☐	☐	☐	☐	☐	☐	☐	☐	☐	
Coronary artery disease*	☐	☐	☐	☐	☐	☐	☐	☐	☐	
Diagnosed with/treated for high cholesterol/triglycerides	☐	☐	☐	☐	☐	☐	☐	☐	☐	
Lipodystrophy: fat accumulation	☐	☐	☐	☐	☐	☐				
fat wasting	☐	☐	☐	☐	☐	☐				

* prompt for heart attack, coronary death, sudden death, coronary artery by-pass grafting, coronary angioplasty, carotid endarterectomy

3. **Lipid profile** *Please specify units used and any normal ranges which have changed*

Date of specimen___/___/___ Time of last intake (other than water): Date: ___/___/___ Time (24:00): ___ : ___
Time of draw (24h) ___ : ___ Time of last ART dose: Date: ___/___/___ Time (24:00): ___ : ___

HDL cholesterol (mmol/l mg/dl) __________ **Normal range** (__________________)
LDL cholesterol (mmol/l mg/dl) __________ **Normal range** (__________________)
Serum insulin (mmol/l mg/dl) __________ **Normal range** (__________________)

If the following results have already been reported on main PenPact-1 forms, they need not be reported again

Total cholesterol (mmol/l mg/dl) __________ **Normal range** (__________________)
Triglycerides (mmol/l mg/dl) __________ **Normal range** (__________________)
Plasma glucose (mmol/l mg/dl) __________ **Normal range** (__________________)

4. **Whole blood sent for serum storage** 3ml ☐ None ☐ Sample ID: __________ Time of draw (24h) ___ : ___

5. **Body circumferences** Date of measurement ___/___/___

Waist	(cm)		1: ________	2: ________	3: ________
Mid-arm	(cm)	R / L*	1: ________	2: ________	3: ________
Mid-thigh	(cm)	R / L*	1: ________	2: ________	3: ________

* delete as appropriate

6. **Skinfold thicknesses** Date of measurement ___/___/___ Not done ☐

Triceps	(cm)	R / L*	1: ________	2: ________	3: ________
Subscapular	(cm)		1: ________	2: ________	3: ________
Supra-iliac	(cm)		1: ________	2: ________	3: ________
Mid-thigh	(cm)	R / L*	1: ________	2: ________	3: ________

* delete as appropriate

7. **MRI** Date of scan ___/___/___ Not done ☐

Model of scanner ______________ Make ______________ Performed by ______________
L4 (cm^2) SAT: ________ VAT: ________ TAT: ________

8. **DEXA** Date of scan ___/___/___ Not done ☐

Model of scanner ______________ Make ______________ Performed by ______________

Total body	fat (kg): ______	fat (%): ______	lean tissue (%): ______	lean tissue (kg): ______
Arm	fat (kg): ______	fat (%): ______	lean tissue (%): ______	lean tissue (kg): ______
Leg	fat (kg): ______	fat (%): ______	lean tissue (%): ______	lean tissue (kg): ______
Trunk	fat (kg): ______	fat (%): ______	lean tissue (%): ______	lean tissue (kg): ______

PENPACT1 PENTA9 PENPACT1 PENTA9 PENPACT1 PENTA9

APPENDIX III (Cont.)

9. Photographs Date of photographs ___/___/___ Not done ☐

Lower arm R / L* ☐ Thigh R / L* ☐ Face ☐ Side profile ☐

* delete as appropriate

10. CARER assessment of change in body shape in the last 6 months (tick one)

Mild – the change is only noticeable when specifically inspected; Moderate – the change is readily obvious to the child/carer;
Severe – the change is obvious to a casual observer

**In the last 6 months has there been a change in**	Severe fat loss	Moderate fat loss	Mild fat loss	No change	Mild fat gain	Moderate fat gain	Severe fat gain
the appearance of your child's face?	☐	☐	☐	☐	☐	☐	☐
the shape of the front or sides of your child's neck?	☐	☐	☐	☐	☐	☐	☐
the shape of the back or base of your child's neck?	☐	☐	☐	☐	☐	☐	☐
the appearance of your child's back or spine?	☐	☐	☐	☐	☐	☐	☐
the appearance of your child's arms?	☐	☐	☐	☐	☐	☐	☐
the appearance of your child's breasts?	☐	☐	☐	☐	☐	☐	☐
the size of your child's waist (belly, abdomen)?	☐	☐	☐	☐	☐	☐	☐
the appearance of your child's legs?	☐	☐	☐	☐	☐	☐	☐
the shape of your child's buttocks (bottom)?	☐	☐	☐	☐	☐	☐	☐
the appearance of your child's thighs?	☐	☐	☐	☐	☐	☐	☐
the number of any lipomas or fat lumps?	☐	☐	☐	☐	☐	☐	☐
the size of any fat lumps (lipomas)?	☐	☐	☐	☐	☐	☐	☐

In the last 6 months has there been a change in the appearance of the veins in your child's
arms? ☐ more visible ☐ no change ☐ less visible
legs? ☐ more visible ☐ no change ☐ less visible

11. PHYSICIAN assessment of change in body shape in the last 6 months

Mild – the change is only noticeable when specifically inspected; Moderate – the change is readily obvious to the child/carer;
Severe – the change is obvious to a casual observer

**In the last 6 months has there been a change in**	Severe fat loss	Moderate fat loss	Mild fat loss	No change	Mild fat gain	Moderate fat gain	Severe fat gain
the appearance of the child's face?	☐	☐	☐	☐	☐	☐	☐
the shape of the front or sides of the child's neck?	☐	☐	☐	☐	☐	☐	☐
the shape of the back or base of the child's neck?	☐	☐	☐	☐	☐	☐	☐
the appearance of the child's back or spine?	☐	☐	☐	☐	☐	☐	☐
the appearance of the child's arms?	☐	☐	☐	☐	☐	☐	☐
the size of the child's breasts?	☐	☐	☐	☐	☐	☐	☐
the size of the child's waist (belly, abdomen)?	☐	☐	☐	☐	☐	☐	☐
the appearance of the child's legs?	☐	☐	☐	☐	☐	☐	☐
the shape of the child's buttocks (bottom)?	☐	☐	☐	☐	☐	☐	☐
the appearance of the child's thighs?	☐	☐	☐	☐	☐	☐	☐
the number of any lipomas or fat lumps?	☐	☐	☐	☐	☐	☐	☐
the size of any fat lumps (lipomas)?	☐	☐	☐	☐	☐	☐	☐

In the last 6 months has there been a change in the appearance of the veins in the child's
arms? ☐ more visible ☐ no change ☐ less visible
legs? ☐ more visible ☐ no change ☐ less visible

12. Comments ____

SIGNED: ________________________ **POSITION:** __________________ **DATE:** ___/___/___

(PRINT NAME): __________________ **CENTRE:** ______________________________

APPENDIX III (Cont.)

PENPACT 1 PENTA9 PENPACT 1 PENTA9 PENPACT 1 PENTA9 PENPACT 1 PENTA9

ISRCTN 73318385

Week no. ______

PENPACT 1 LIPODYSTROPHY SUBSTUDY – FOLLOW UP

Form L2
March 2003

Please give all dates as dd/mm/yy

**Clinic Number:**	**Date of Birth:**	**Trial Number:**
**Initials:** ☐ ☐ ☐	**Date of Assessment:**	

1. **Lipid profile** *Please specify units used and any normal ranges which have changed*

   Date of specimen___/___/___ Time of last intake (other than water): Date: ___/___/___ Time (24:00): ___ : ___

   Time of draw (24h) ___ : ___ Time of last ART dose: Date: ___/___/___ Time (24:00): ___ : ___

   HDL cholesterol (mmol/l mg/dl) __________ **Normal range** (____________________)

   LDL cholesterol (mmol/l mg/dl) __________ **Normal range** (____________________)

   Serum insulin (mmol/l mg/dl) __________ **Normal range** (____________________)

   If the following results have already been reported on main PenPact-1 forms, they need not be reported again

   Total cholesterol (mmol/l mg/dl) __________ **Normal range** (____________________)

   Triglycerides (mmol/l mg/dl) __________ **Normal range** (____________________)

   Plasma glucose (mmol/l mg/dl) __________ **Normal range** (____________________)

2. **Whole blood sent for serum storage** 3ml ☐ None ☐ Sample ID: __________ Time of draw (24h) ___ : ___

3. **Body circumferences** Date of measurement ___/___/___

   Waist (cm) 1: __________ 2: __________ 3: __________

   Mid-arm (cm) R / L* 1: __________ 2: __________ 3: __________

   Mid-thigh (cm) R / L* 1: __________ 2: __________ 3: __________

   * delete as appropriate

4. **Skinfold thicknesses** Date of measurement ___/___/___ Not done ☐

   Triceps (cm) R / L* 1: __________ 2: __________ 3: __________

   Subscapular (cm) 1: __________ 2: __________ 3: __________

   Supra-iliac (cm) 1: __________ 2: __________ 3: __________

   Mid-thigh (cm) R / L* 1: __________ 2: __________ 3: __________

   * delete as appropriate

5. **MRI** Date of scan ___/___/___ Not done ☐

   Model of scanner ______________ Make ______________ Performed by ______________

   L4 (cm^2) SAT: __________ VAT: __________ TAT: __________

6. **DEXA** Date of scan ___/___/___ Not done ☐

   Model of scanner ______________ Make ______________ Performed by ______________

   Total body fat (kg): ______ fat (%): ______ lean tissue (%): ______ lean tissue (kg): ______

   Arm fat (kg): ______ fat (%): ______ lean tissue (%): ______ lean tissue (kg): ______

   Leg fat (kg): ______ fat (%): ______ lean tissue (%): ______ lean tissue (kg): ______

   Trunk fat (kg): ______ fat (%): ______ lean tissue (%): ______ lean tissue (kg): ______

7. **Have either of the child's parents suffered from lipodystrophy?** Yes ☐ No ☐ Not known ☐

   If yes, specify below

	Biological Mother			Biological Father		
**Lipodystrophy**	No	Yes	Not known	No	Yes	Not known
fat accumulation	☐	☐	☐	☐	☐	☐
fat wasting	☐	☐	☐	☐	☐	☐

8. **Photographs** Date of photographs ___/___/___ Not done ☐

   Lower arm R / L* ☐ Thigh R / L* ☐ Face ☐ Side profile ☐

   * delete as appropriate

PENPACT1 PENTA9 PENPACT1 PENTA9 PENPACT1 PENTA9

APPENDIX III (Cont.)

9. CARER assessment of change in body shape since initiation of ART (tick one)

Mild – the change is only noticeable when specifically inspected; Moderate – the change is readily obvious to the child/carer; Severe – the change is obvious to a casual observer

**Since initiating ART has there been a change in**	Severe fat loss	Moderate fat loss	Mild fat loss	No change	Mild fat gain	Moderate fat gain	Severe fat gain
the appearance of your child's face?	☐	☐	☐	☐	☐	☐	☐
the shape of the front or sides of your child's neck?	☐	☐	☐	☐	☐	☐	☐
the shape of the back or base of your child's neck?	☐	☐	☐	☐	☐	☐	☐
the appearance of your child's back or spine?	☐	☐	☐	☐	☐	☐	☐
the appearance of your child's arms?	☐	☐	☐	☐	☐	☐	☐
the appearance of your child's breasts?	☐	☐	☐	☐	☐	☐	☐
the size of your child's waist (belly, abdomen)?	☐	☐	☐	☐	☐	☐	☐
the appearance of your child's legs?	☐	☐	☐	☐	☐	☐	☐
the shape of your child's buttocks (bottom)?	☐	☐	☐	☐	☐	☐	☐
the appearance of your child's thighs?	☐	☐	☐	☐	☐	☐	☐
the number of any lipomas or fat lumps?	☐	☐	☐	☐	☐	☐	☐
the size of any fat lumps (lipomas)?	☐	☐	☐	☐	☐	☐	☐

Since initiating ART has there been a change in the appearance of the veins in your child's

arms? ☐ more visible ☐ no change ☐ less visible
legs? ☐ more visible ☐ no change ☐ less visible

10. PHYSICIAN assessment of change in body shape since initiation of ART

Mild – the change is only noticeable when specifically inspected; Moderate – the change is readily obvious to the child/carer; Severe – the change is obvious to a casual observer

**Since initiating ART has there been a change in**	Severe fat loss	Moderate fat loss	Mild fat loss	No change	Mild fat gain	Moderate fat gain	Severe fat gain
the appearance of the child's face?	☐	☐	☐	☐	☐	☐	☐
the shape of the front or sides of the child's neck?	☐	☐	☐	☐	☐	☐	☐
the shape of the back or base of the child's neck?	☐	☐	☐	☐	☐	☐	☐
the appearance of the child's back or spine?	☐	☐	☐	☐	☐	☐	☐
the appearance of the child's arms?	☐	☐	☐	☐	☐	☐	☐
the size of the child's breasts?	☐	☐	☐	☐	☐	☐	☐
the size of the child's waist (belly, abdomen)?	☐	☐	☐	☐	☐	☐	☐
the appearance of the child's legs?	☐	☐	☐	☐	☐	☐	☐
the shape of the child's buttocks (bottom)?	☐	☐	☐	☐	☐	☐	☐
the appearance of the child's thighs?	☐	☐	☐	☐	☐	☐	☐
the number of any lipomas or fat lumps?	☐	☐	☐	☐	☐	☐	☐
the size of any fat lumps (lipomas)?	☐	☐	☐	☐	☐	☐	☐

Since initiating ART has there been a change in the appearance of the veins in the child's

arms? ☐ more visible ☐ no change ☐ less visible
legs? ☐ more visible ☐ no change ☐ less visible

11. Comments ____
____
____

SIGNED: ____________________ **POSITION:** ________________ **DATE:** ___/___/___

(PRINT NAME): ____________________ **CENTRE:** ______________________________

Please return this form to: PENTA trials, 222 Euston Road, London NW1 2DA, UK
Tel: 00 44 (0) 20 7670 4791 Fax: 00 44 (0) 20 7670 4814 email: PENTA @ctu.mrc.ac.uk

APPENDIX IV

DIVISION OF AIDS
TOXICITY TABLE FOR GRADING SEVERITY of PEDIATRIC
(≤3 MONTHS OF AGE) ADVERSE EXPERIENCES. April-1994
(TO BE USED BY PACTG AND PENTA)

The PENPACT 1 (PENTA 9/PACTG 390) Protocol Team added the units for laboratory test results to facilitate the table's use at PENTA sites.

For other findings, the Toxicity Table for children ≥3 months of age (April, 1994) is applicable. All values here are for term newborns. Preterm infants should be judged by a comparison of local normal ranges and the newborn ranges identified here.

PARAMETER	GRADE 1	GRADE 2	GRADE 3	GRADE 4
HEMATOLOGY				
HEMOGLOBIN (g/dL)				
1-7 days old	13.0-14.0	12.0-12.9	<12	Cardiac Failure 2ndary to Anemia
8-21 days old	12.0-13.0	10.0-11.9	<10.0	Cardiac Failure 2ndary to Anemia
22-35 days old	9.5-10.5	8.0-9.4	<8.0	Cardiac Failure 2ndary to Anemia
36-56 days old	8.5-9.4	7.0-8.4	<7.0	Cardiac Failure 2ndary to Anemia
57-90 days old	9.0-9.9	7.0-8.9	<7.0	Cardiac Failure 2ndary to Anemia
ABS NEUTROPHIL CT/mL				
1 day old	5000-7000	3000-4999	1500-2999	<1500
2-7 days old	1750-2500	1250-1749	750-1249	<750
8-56 days old	1200-1800	900-1199	500-899	<500
57-90 days old	750-1200	400-749	250-399	<250

APPENDIX IV (Cont)

PARAMETER	GRADE 1	GRADE 2	GRADE 3	GRADE 4
BILIRUBIN (mg/dL)				
<7 days old	.	20-25	26-30	>30
7-60 days old	1.1-1.9xN	2.0-2.9xN	3.0-7.5xN	>7.5xN
61-90 days old	1.1-1.9xN	2.0-2.9xN	3.0-7.5xN	>7.5xN
CREATININE (mg/dL)				
<7 days old	1.0-1.7	1.8-2.4	2.5-3.0	>3.0
7-60 days old	0.5-0.9	1.0-1.4	1.5-2.0	>2.0
61-90 days old	0.6-0.8	0.9-1.1	1.2-1.5	>1.5
CR CLEARANCE (cc/min/1.73 m2)				
<7 days old	35-40	30-34	25-29	<25
7-60 days old	45-50	40-44	35-39	<35
61-90 days old	60-75	50-59	35-49	<35
Low Calcium (mg/dL)				
<7 days old	6.5-6.9	6.0-6.4	5.5-5.9	<5.5
7-60 days old	7.6-8.0	7.0-7.5	6.0-6.9	<6.0
61-90 days old	7.8-8.4	7.0-7.7	6.0-6.9	<6.0
High Calcium (mg/dL)				
<7 days old	12.0-12.4	12.5-12.9	13.0-13.5	>13.5
7-60 days old	10.5-11.2	11.3-11.9	12.0-13.0	>13.0
61-90 days old	10.5-11.2	11.3-11.9	12.0-12.9	>= 13.0

APPENDIX V

DIVISION OF AIDS
TOXICITY TABLE for GRADING SEVERITY of
PEDIATRIC (> 3 MONTHS OF AGE) ADVERSE EXPERIENCES April, 1994
(TO BE USED BY PACTG AND PENTA)

THE PENPACT 1 (PENTA 9/PACTG 390) PROTOCOL TEAM ADDED THE UNITS FOR LABORATORY TEST RESULTS TO FACILITATE THE TABLE'S USE AT PENTA SITES

PARAMETER	GRADE 1	GRADE 2	GRADE 3	GRADE 4
HEMATOLOGY				
Hemoglobin (g/dL)<br>> 3 mo.- < 2 y.o.	9.0-9.9	7.0-8.9	<7.0	Cardiac Failure<br>2ndary to anemia
Hemoglobin (g/dL)<br>> = 2 y.o.	10-10.9	7.0-9.9	<7.0	Cardiac Failure<br>2ndary to anemia
Abs Neutrophil CT/µL	750-1200	400-749	250-399	<250
Platelets/µL		50,000-75,000	25,000-49,999	<25,000 or bleeding
PT (seconds)	1.1-1.25xN	1.26-1.5xN	1.51-3.0xN	>3xN
PTT (seconds)	1.1-1.66xN	1.67-2.33xN	2.34-3.0xN	>3xN
GASTROINTESTINAL				
Bilirubin (mg/dL)	1.1-1.9xN	2.0-2.9xN	3.0-7.5xN	>7.5xN
AST (SGOT)	1.1-4.9xN U/L	5.0-9.9xN U/L	10.0-15.0xN U/L	>15.0xN U/L
ALT (SGPT)	1.1-4.9xN U/L	5.0-9.9xN U/L	10.0-15.0xN U/L	>15.0xN U/L
GGT	1.1-4.9xN U/L	5.0-9.9xN U/L	10.0-15.0xN U/L	>15.0xN U/L
Pancreatic Amylase	1.1-1.4xN U/L	1.5-1.9xN U/L	2.0-3.0xN U/L	>3.0xN U/L
Total Amylase + Lipase*	1.1-1.4xN U/L	1.5-2.4xN U/L	2.5-5.0xN U/L	>5.0xN U/L
Uric Acid (mg/dL)	7.5-9.9	10-12.4	12.5-15.0	>15.0 or Gout
CPK	See Neuromuscular Toxicity			
Abdominal Pain	Mild	Moderate-<br>No Rx Needed	Moderate-<br>Rx Needed	Severe-<br>Hospital and Rx
Diarrhea	Soft stools	Liquid stools	Liquid Stools &<br>Mild Dehydration<br>Bloody stools	Dehydration requiring IV therapy or Hypotensive Shock
Constipation	Mild	Moderate	Severe	Distention and Vomiting
Nausea	Mild	Moderate-<br>Decreased po intake	Severe-Little<br>po intake	Unable to ingest food<br>or fluid for >24 hours
Vomiting	<1 episode/day	1-3 episodes/day or<br>duration >3d	>3 episodes/day or<br>duration >7d	Intractable Vomiting

Comments:
*Both amylase and lipase must be elevated to the same grade or higher (i.e. if total amylase is Grade 4, but lipase is only Grade 1, the Toxicity Grade is 1. In pediatric HIV patients, the most common source of serum amylase is the salivary glands. Salivary amylase elevations are generally not clinically significant. When amylase is released from damaged pancreatic cells, it can be a marker of pancreatitis. In most cases of clinical pancreatitis, lipase will also be elevated. However, lipase is also a non-specific marker. Combined elevation of amylase and lipase (each >5 x normal) often indicates pancreatic disease and requires evaluation. However, in the absence of pancreatic disease, drug can be resumed even at Grade 3 and 4 toxicities.

APPENDIX V (Cont.)

PARAMETER	GRADE 1	GRADE 2	GRADE 3	GRADE 4
RENAL AND ELECTROLYTES				
CREATININE (mg/dL)				
2 Month-2 Years	0.6-0.8	0.9-1.1	1.2-1.5	>1.5
2 Years-Adolescent	0.7-1.0	1.1-1.6	1.7-2.0	>2.0
Adolescents	1.0-1.7	1.8-2.4	2.5-3.5	>3.5
Creatinine Clearance (cc/min/1.73 m^2)	60-75	50-59	35-49	<35
ELECTROLYTES				
High Sodium (mmo/L)	145-149		150-155	>155 or mental status changes
Low Sodium (mmo/L)	130-135		129-124	<124 or mental status changes
High Potassium (mmo/L)	5.0-5.9	6.0-6.4	6.5-7.0	>7.0 or Cardiac arrhythmias
Low Potassium (mmo/L)	3.0-3.5	2.5-2.9	2.0-2.4	<2.0
High Calcium (mg/dL)	10.5-11.2	11.3-11.9	12.0-12.9	>=13.0
Low Calcium (mg/dL)	7.8-8.4	7.0-7.7	6.0-6.9	<6.0
Low Magnesium (mg/dL)	1.2-1.4	0.9-1.1	0.6-0.8	<0.6 or Cardiac arrhythmias
Hypoglycemia (mg/dL)	55-65	40-54	30-39	<30 or Mental status changes
Hyperglycemia (mg/dL)	116-159	160-249	250-400	>400 or Ketoacidosis
Proteinuria (mg/dL)	Tr-1+<br><150 mg/day	2+<br>150-499 mg/day	3+<br>500-1000 mg/day	4+, or nephrotic syndrome<br>>1000 mg/day
Hematuria	Microscopic<br><25 cells/hpf	Microscopic<br>>=25 cells/hpf	Gross	Obstruction or Transfusion requirement
Comments<br>Calcium values are corrected for albumin concentration. CrCl values do not apply to infants <2 months old.				
OTHER				
Allergy	Pruritis without Rash	Pruritic Rash	Mild Urticaria	Severe Urticaria<br>Anaphylaxis, Angioedema
Drug Fever (Rectal)		38.5-40 °C	>40 °C	Sustained Fever:<br>>40 °C, >5 days
Cutaneous		Diffuse maculo-papular rash, dry desquamation	Vesiculation, ulcers	Exfoliative dermatitis, Stevens-Johnson or Erythema multiforme, Moist desquamation
Stomatitis	Mild discomfort	Painful, difficulty swallowing, but able to eat and drink	Painful: unable to swallow solids	Painful: requires IV fluids

APPENDIX V (Cont.)

SYMPTOM	GRADE 1	GRADE 2	GRADE 3	GRADE 4
CENTRAL NERVOUS SYSTEM				
Seizures	None	1 Uncomplicated Sz<br>+/- Temp Elevation	1 Sz/Month for<br>>=2 Consecutive Months<br>Or 3 Sz over 6 Months; No Temp Elevation	≥1 Sz/Month;<br>No Temp Elevation;<br>No Decrease in Sz Frequency<br>Despite dose reduction
Seizures are a ubiquitous symptom of numerous systemic or CNS disturbances; alternative explanations should be vigorously sought and eliminated. Status epilepticus represents a severe end of the seizure spectrum, but should be considered as a single seizure event. The need for chronic or acute anticonvulsant medication should be made on a clinical basis. Seizures as a manifestation of drug toxicity are usually primarily generalized. Focal (partial onset) seizures are suggestive of focal central nervous system pathology and should be appropriately investigated, although they may be a manifestation of drug toxicity. Beware of focal seizures which secondarily generalize; these should be approached diagnostically as partial onset seizures. Children with underlying epileptic conditions who experience persistent breakthrough seizures despite maximal anticonvulsant therapy coincident with beginning the trial medication should be considered Grade 4.				
Headache	<=1/Month<br><2 Hrs duration<br>Mild	>1/Month<br>>2 Hrs Duration<br>Moderate to Severe<br>Responds to non-narcotic analgesia or prophylaxis	>2/Month<br>>2 Hrs Duration<br>Moderate to Severe<br>Responds to narcotic analgesia, or<br>does not respond<br>to prophylaxis	>4/Month;<br>>2 Hrs Duration;<br>Moderate to Severe;<br>Non-Responsive to narcotic Analgesia; or persistently Recurrent despite prophylaxis<br>No decrease in frequency or Severity despite dose reduction
Headache is a non-specific symptom, but may be a symptom of CNS/intracranial pathology. Appropriate diagnostic measures should be pursued. Duration refers to the waxing and peak phases, not to the resolution/waning phases of the headache. Mild refers to a grade of headache pain which does not affect function or activity. Moderate to severe refers to a grade of headache which affects function or activity.				
Mental Status<br>And Behavior	Changes which do not Affect Function	Changes requiring pharmacologic or other therapy; or mild lethargy, sedation or somnolence which resolves with rest	Changes not improved by drugs or other therapies; or onset of confusion, memory impairment, lethargy, sedation, or somnolence which does not respond to rest	Onset of delirium, obtundation, coma, or psychosis, or Grade 3 toxicity which does not respond to dose reduction
Behavior refers to the development of attention deficits with or without hyperactivity, depression, mania, agitation, sleep disorders, phobias, obsessive-compulsive behaviors, or anxiety. Mental status refers to the level of consciousness, memory function, language and analytical operations, and non-dominant hemisphere functioning. Alternative explanations should be sought.				
Balance & Posture	None	None	Ataxia, dizziness, vertigo, tremor, impaired postural balance	Onset of movement disorder; or Grade 3 toxicity which does not respond to dosage adjustment
"Ataxia" can be mistakenly diagnosed in the face of central weakness or peripheral neuropathy, which should not be considered a drug toxicity of this category. Movement disorders refer to tardive or other dyskinesias, dystonias, chorea, or ballismus. Alternative explanations should be sought.				

APPENDIX V (Cont.)

SYMPTOM	GRADE 1	GRADE 2	GRADE 3	GRADE 4
Visual	None	Blurriness, diplopia, or horizontal nystagmus of < 1 hour duration, with spontaneous resolution	> = 1 episode of Grade 2 symptoms per week, or an episode of Grade 2 Sx lasting 1 hour with spontaneous resolution by 4 hours or vertical nystagmus	Decrease in visual acuity, visual field deficit, or oculogyric crisis, or Grade 3 Sx which persist after dose reduction
Many of the symptoms in this category can be the result of CNS pathology, or alternatively can be an external (i.e., non-CNS) neuro-ophthalmologic disorder. Appropriate diagnostic investigations should be pursued.				
Myelopathy	None	None	None	Myelopathic/spinal cord symptoms, such as: Pyramidal tract weakness and disinhibition, sensory level, loss of proprioception, bladder/bowel dysfunction
HIV can cause spinal cord syndromes rarely in children. Other infectious agents can cause myelopathies as well. Alternative explanations should be sought.				
PERIPHERAL NERVOUS SYSTEM				
Neuropathy/ Lower Motor Neuronopathy	None	Mild transient Paresthesia only	Persistent or progressive paresthesias, burning sensation in feet, or mild dysesthesia; no weakness; mild to moderate deep tendon reflex changes; no sensory loss	Onset of significant weakness, decrease or loss of DTRs, sensory loss in "stocking glove" distribution, radicular sensory loss, multiple cranial nerve involvement; bladder or bowel dysfunction, fasciculations, respiratory embarrassment from chest wall weakness. Grade 3 symptoms which do not resolve with dose reduction
Infectious agents other than HIV can precipitate a neuropathy and should be considered, especially CMV. Neuropathies which do not resolve after dose reduction or discontinuation should be pursued for alternative infectious or non-infectious etiologies, since drug-related neuropathies will usually resolve after dose reduction or drug discontinuation. It should be borne in mind that many subjects will worsen for up to one month after drug discontinuation prior to improvement ("coasting"). Abnormalities should be confirmed by nerve conduction studies (NCS) +/- electromyographic studies (EMG).				
Myopathy or Neuromuscular Junction Impairment	Normal or mild (<2 x N) CPK elevation	Mild proximal weakness and/or atrophy not affecting gross motor function. Mild myalgias, +/- mild CPK elevation (<2 x N)	Proximal muscle weakness and/or atrophy affecting motor function +/- CPK elevation; or severe myalgias with CPK >2 x N; Consider confirmatory EMG and/or muscle bx	Onset of myasthenia-like symptoms (fatiguable weakness with external, variable ophthalmoplegia and/or ptosis), or neuromuscular junction blockade (acute paralysis) symptoms (confirm with EMG); or Grade 3 symptoms which do not resolve on dose adjustment; confirm with muscle bx
HIV can produce a myopathy, and should be differentiated. Drug-induced myopathy can be accompanied by normal CPK levels. On occasion, neuropathic or central weakness can mimic myopathic weakness.				

APPENDIX V (Cont.)

SYMPTOM	GRADE 1	GRADE 2	GRADE 3	GRADE 4
Clinical symptoms *not otherwise specified* in this table	No therapy; monitor condition	May require minimal intervention and monitoring	Requires medical care and possible hospitalization	Requires active medical intervention, hospitalization, or hospice care
Laboratory values *not otherwise specified* in this table	Abnormal, but requiring no immediate intervention; follow	Sufficiently abnormal to require evaluation as to causality and perhaps mild therapeutic intervention, but not of sufficient severity to warrant immediate changes in study drug	Sufficiently severe to require evaluation and treatment, including at least temporary suspension of study drug	Life-threatening severity. Requires immediate evaluation, treatment, and usually hospitalization. Study drug must be stopped immediately and should not be restarted until the abnormality is clearly felt to be caused by some other mechanism that study drug.

APPENDIX VI

SUPPLEMENTAL TOXICITY TABLE FOR GRADING SEVERITY OF ADULT AND PEDIATRIC CUTANEOUS/SKIN RASH/DERMATITIS ADVERSE EXPERIENCES

(TO BE USED BY PACTG AND PENTA)

GRADE 1	GRADE 2	GRADE 3*	GRADE 4*
CUTANEOUS/SKIN RASH/DERMATITIS			
Erythema, with or without pruritis	A. Diffuse erythematous macular or maculopapular cutaneous eruption or dry desquamation with or without pruritis (without the presence of any additional constitutional findings as described for Grade 3); OR typical target lesions without blistering, vesicles, or ulcerations in the lesions.<br><br>B. Urticaria	A. Diffuse erythematous macular or maculopapular cutaneous eruption or moist desquamation with or without pruritis together with any of the following constitutional findings considered related to study drug:<br>1. 5 x ULN AST, ALT or 2 x baseline if baseline > ULN.<br>2. fever, >39°C<br>3. blistering and/or vesiculation of cutaneous eruptions<br>4. any site of mucosal lesions; OR<br><br>B. angioedema; OR<br><br>C. exfoliative dermatitis defined as severe widespread erythema and dry scaling of the skin, with generalized superficial lymphadenopathy, and with other constitutional findings such as fever, weight loss, hypoproteinemia possibly related to study drug; OR<br><br>D. diffuse rash and serum sickness-like reactions defined as a clinical symptom complex manifested as fever, lymphadenopathy, edema, myalgia, and/or arthralgia; OR<br><br>E. diffuse cutaneous eruptions, usually starting on the face, trunk or back, often with prodromal symptoms plus one of the following:<br>1. cutaneous bullae, sometimes confluent with widespread sheet-like detachment of skin (<10% body surface area), (Nikolski's sign)(Stevens Johnson Syndrome, SJS)<br>2. two or more anatomically distinct sites of mucosal erosion or ulceration not due to another cause.	Diffuse cutaneous eruptions, usually starting on the face, trunk or back, often with prodromal symptoms plus cutaneous bullae with widespread sheet-like detachment of skin (>10% of body surface area), (Nikolski's sign), (SJS/Toxic Epidermal Necrolysis (TEN) overlap syndrome; TEN)
*When a Grade 3 or 4 cutaneous/skin rash/dermatitis adverse experience is suspected, a Dermatology consult for photographs and biopsies is required.			

APPENDIX VII

DETERMINATION OF PLASMA HIV-1 RNA-PACTG SITES

VIROLOGY

ASSAY	SPECIMEN	COLLECTION CONTAINER	IMMEDIATE SPECIMEN HANDLING
HIV-1 RNA PCR and plasma for storage<br>(Roche 1.5 UltraSensitive assay,)	3.0 mL blood	**k3 EDTA-Tubes**	• Gently invert tubes several times to mix. Do not shake.<br>• Specimen should be identified as to patient ID# (PID), study ID# (SID), site ID#, visit ID#, date and time of collection, and specimen type.<br>• Specimen should be kept at room temperature (18°-24°C) and processed as quickly as possible, preferably within 4 to 6 hours of collection.
PBMC for storage<br>(resistance analysis and analysis of proviral DNA)	7.0 mL blood<br>5.0 mL blood		

SPECIMEN PROCESSING:

PLASMA FOR HIV-1 RNA PCR – UltraSensitive Roche Monitor Test (v1.5). Please follow the consensus methods on the ACTG website at http://aactg.s-3.com/pub/download/SpecimenProcessingGuide.doc.

1. Specimens must be logged into the LDMS and labeled with the LDMS specimen #, PID, date, time of collection and derivative information.
2. Aliquot clarified plasma into a minimum of 2 x 0.6mL volumes (LDMS code: BLD/EDT/PL2)
3. Freeze immediately at -70ºC.
4. **Patient management HIV RNA PCR can be performed locally at a DAIDS VQA certified laboratory.**
5. **Plasma aliquots from specimens collected at Screening, Entry, Week 24, time of therapy switch, Week 192, Week 204, and end of study must be reserved; these aliquots will be batched and shipped to the University of North Carolina prior to study completion according to the instructions below.**
6. **If there is no local DAIDS VQA certified laboratory available for HIV RNA testing, specimens can be sent real-time to the University of North Carolina.**

PLASMA AND PBMC FOR STORAGE- Please follow the consensus methods on the ACTG website at http://aactg.s-3.com/pub/download/SpecimenProcessingGuide.doc and http://aactg.s-3.com/pub/download/vir/freezingprotocol.doc

1. Specimens must be logged into the LDMS and labeled with the LDMS specimen #, PID, date, time of collection and derivative information.
2. Aliquot clarified plasma into a minimum of 5-7 x 0.6mL volumes (LDMS code: BLD/EDT/PL2)
3. Freeze immediately at -70ºC.
4. Freeze cells viably at 2.5 x 10^6 cells per vial (BLD/EDT/CEL/DMS) and freeze according to the consensus method for cryopreservation.

SHIPPING INSTRUCTIONS: Sites should ship 2 x 0.6mL PL2 to University of North Carolina for **batch** RNA testing. All specimens should be packaged according to the ACTG Virology Manual (http://aactg.s-3.com/specship.htm) with strict attention to Federal and carrier-specific regulations for the shipment of diagnostic specimens. Include sufficient dry ice to keep the specimens frozen. Ship via overnight carrier (using the before 10:30 AM option) on the following preferred days: Monday, Tuesday and Wednesday. PBMCs will be stored at each site until requested by protocol team.
DO NOT SHIP SAMPLES ON FRIDAY OR WHEN THEY WOULD BE RECEIVED ON A HOLIDAY. HOLIDAYS INCLUDE: New Year's Day, M.L. King's Birthday, Good Friday, Memorial Day, July 4, Labor Day, Thanksgiving and the day after Thanksgiving, and several days around Christmas.

Notify the lab by FAX # prior to shipment with the airbill number. This is a Federal regulation.Notify the lab by FAX # prior to shipment with the airbill number. This is a Federal regulation.

DESIGNATED LABORATORY/CONTACT PERSON:
Melissa Kerkau
University of North Carolina
School of Medicine
Retrovirology Core Laboratory
709 Mary Ellen Jones Building, CB#7140
Chapel Hill, NC 27599-7140
TEL: (919)-966-6867
FAX: (919)-966-9873

NOTE: The Roche 1.5 UltraSensitive assay will be used for all HIV-1 RNA determinations. When a specimen has a result with >100,000 cp/mL, the specimen should be diluted 1:100 (performed as two serial 10-fold dilutions) and re-run on the UltraSensitive assay.

APPENDIX VIII

DETERMINATION OF PLASMA HIV-1 RNA- PENTA SITES

VIROLOGY

ASSAY	SPECIMEN	COLLECTION CONTAINER	IMMEDIATE SPECIMEN HANDLING
HIV-1 RNA PCR<br>(ultrasensitive assay)	3.0 mL blood	EDTA tubes	• Gently invert tubes several times to mix. Do not shake.<br>• Add trial number, date and time of collection, and specimen type to PENTA ID labels. Complete details on laboratory request form.<br>• Specimen should be kept at room temperature (18°-24°C) and processed as quickly as possible, preferably within 4 to 6 hours of collection.
Plasma and PBMC for storage<br>(resistance analysis and analysis of proviral DNA)	**7.0** mL blood<br>5.0 mL blood		

SPECIMEN PROCESSING:

PLASMA FOR LOCAL HIV-1 RNA PCR: Weeks –2, 0, 8, 16, 24, then every 12 weeks, for each regimen.
HIV-1 RNA should be measured as per local protocol. The Roche assay should be used if possible.

PLASMA and PBMC for storage: Weeks -2, 0, 24, 48, 72, 96, 120, 144, 168, 192 every 24 weeks there after, for each regimen.

The following procedures are suggested; however, variations may be needed. Please contact your trial center if you have any doubts.

1. Pellet the whole blood at 1000 x g for 10 minutes at 30°C.
2. Remove the plasma by pipetting down to within 0.5 cm of the buffy coat.
3. Remove the remaining plasma and buffy coat, being careful to remove as few RBCs as possible.
4. Dilute the buffy coat up to 10 mL in a 15-mL conical centrifuge tube with warmed (37°C) PBS.
5. Add 2 mL Ficoll-paque solution to each required number of 15-mL conical centrifuge tubes by gently underlaying the diluted blood.
6. Centrifuge at 500 x g for 25 minutes at 30°C.
7. Aspirate the plasma fraction down to within 2 cm of the PBMC band at the interface between the plasma fraction and the Ficoll-paque solution.
8. Carefully remove the PBMCs with a pipette, taking care not to aspirate any more Ficoll-paque solution than necessary.
9. Transfer the PBMCs to a 50-mL conical centrifuge tube and dilute up to 50 mL with warmed PBS.
10. Pellet the PBMCs at 1000 x g for 10 minutes at 30°C.
11. Wash the cell pellet (pellets from the same blood draw may now be pooled if desired) twice with 50 mL of warmed PBS and twice with serum free RPMI-1640. Count cell number. Usual yields per 10 mL whole blood equal 1-2 x 10^7 PBMCs total.
12. Prepare freezing medium (90% fetal bovine serum and 10% DMSO).
13. Pellet counted PBMCs at 500 x g, then re-suspend the pellet in freezing medium at 10^6 PBMCs per mL.
14. Label two or three 1·5 ml cryotubes with date and trial number. (note need 5 x 10^6 cells per tube).
15. Transfer cells to cryotubes.
16. Freeze the cryotubes slowly overnight until the temp. -70 ºC is reached : if your center does not have a controlled rate freezer, the best alternative is to obtain a "Mr Frosty" cryobox which will ensure slow freezing (details available from the trial center - please contact in advance of children entering the trial). Alternatively, place cryotubes in a thick-walled plastic box at room temperature and place the box in the -70ºC freezer. Transfer to liquid Nitrogen the next day (which is preferable) or if not available, store at -70 ºC.

SHIPPING INSTRUCTIONS:

Stored plasma and cells should stay in the local laboratory until the end of the trial. Shipping will then be arranged by the Trials Centres.

NOTES: The ultrasensitive assay will be used for all HIV-1 RNA determinations. When a specimen has a result with >100,000 cp/mL, the specimen should be diluted 1:100 (performed as two serial 10-fold dilutions) and re-run on the ultrasensitive assay.

APPENDIX IX

ALLOWED ANTIRETROVIRAL THERAPY BACKGROUND INFORMATION, SUGGESTED DOSING AND MAJOR TOXICITIES FOR **IMPAACT** AND PENTA

This appendix establishes the allowed antiretrovirals that could be prescribed for PENPACT 1 children, and was written to assist clinicians in selecting each child's regimen by using FDA/EMEA approved dosing or the PENPACT1 dosing recommendations.

All the information in this appendix is to be used as a guideline for prescribing the child's regimen. However complete and detailed prescribing and toxicity information, on all antiretroviral drugs, is available from the drug manufacturer's in the USA and in Europe. Clinicians must review manufacturer's product information before prescribing any of these drugs, and contact the protocol team at actg.penpact1@fstrf.org with any discrepancies or queries.

The PENPACT1 Team will update this appendix, whenever new drugs, drug combinations, drug dosing, and/or drug-regimens, are/is approved by the FDA and/or EMEA. Thus, clinicians in both **IMPAACT**/PACTG and PENTA sites must follow the approved version of this appendix when prescribing. The PENPACT1 Team will review and update this appendix as needed to guarantee that clinicians have the most current options for prescribing the children's ART-regimens.

For PENPACT1, adolescents in early puberty (Tanner I-II - Appendix XV) should be dosed using pediatric schedules, and adolescents in late puberty (Tanner V - Appendix XV) should be dosed using adults schedule. Children who are in the midst of their growth spurt (Tanner III females, Tanner IV males - Appendix) should be closely monitored for medication efficacy and toxicity when choosing either adult or pediatric dosing guidelines. Clinicians should alert the protocol team of the selected dosing schedule when reporting virologic failure or toxicities in these children.

The appendix is divided into one to three sections for each drug, and the drugs are listed in alphabetical order, rather than by class. These three sections are:

(1) Current Preparations and Dosages Including FDA/EMEA Approved Dosing Guidelines

This section summarizes the current preparations and dosage for all antiretroviral drugs allowed in PENPACT 1, among these dosing recommendations the PENPACT 1 Team included the FDA/EMEA approved dosing guidelines. Most of these dosing recommendations can be found in the *"Guidelines for the Use of Antiretroviral Agents in Pediatric HIV Infection", Appendix "Characteristics of Available Antiretroviral Drugs",* dated **October 26, 2006**. Some of the dosing included in this section is not FDA/EMEA approved but **has been used in clinical research under IND, with adequate efficacy and safety observed, and is** commonly used in the clinical setting.

APPENDIX IX

These dosing recommendations can be followed by all sites participating in the study, **IMPAACT**/PACTG and PENTA sites. However, clinicians may opt to prescribe only the approved dosing guidelines issued by their own regulatory agency, either FDA or EMEA. Thus, the current FDA approved dosing guidelines for **IMPAACT**/PACTG sites are for all USA sites participating in **IMPAACT** 390, and the current EMEA approved dosing guidelines for PENTA sites are for all European PENTA countries participating in PENTA 9.

In Germany and Switzerland when a drug, a drug dose, or a drug-formulation is not approved by the EMEA but it is approved by the FDA, this drug dose or drug formulation can be prescribed based on the FDA's approval.

(2) <u>PENPACT 1 Dosing Recommendations</u>

The PENPACT 1 Team includes here dosing recommendations that are not reported in the previous section. These dosing recommendations are based on published scientific papers, or ongoing approved clinical trials or studies, and can be followed by both **IMPAACT** 390 and PENTA 9 sites

(3) <u>Major Toxicities</u>

This section informs site personnel of **some of** the well-known side effects of a given drug. A complete list of risks is available for each drug in its **package** insert. These **risks** must be explained and made available to the children and/or care-givers during the informed consent process.

<u>IMPORTANT NOTES</u>

- Dosing of children with hepatic and/or renal insufficiency, as well as children receiving special dosing due to toxicity, must be done as per drug manufacturer's **package** insert information and/or toxicity management guidelines in this document. Clinicians must contact the protocol team at actg.penpact1@fstrf.org with any dosing concerns regarding special requirements of a given child.
- For both initial- and second-line therapies, it is important to emphasize that PENPACT 1 will allow low doses of ritonavir as a boosting agent, creating drug-combinations that will be counted as a single protease inhibitor.
- Combivir (300 mg zidovudine + 150 mg lamivudine) dosing can be found under lamivudine and zidovudine sections. Major toxicities for this drug combination should be reviewed as per individual drug components.

APPENDIX IX

1.0 ABACAVIR (**ABC**, ZIAGEN®)

1.1 Current Preparations and Dosages Including FDA/EMEA Approved Dosing Guidelines

Antiretroviral Class: NRTI

Preparations:

- Pediatric oral solution: 20 mg/mL
- Tablets: 300 mg
- Tablets in combination with lamivudine: Epzicom®/Kivexa: 600 mg abacavir, 300 mg lamivudine

Dosages:

- Neonatal dose: Not approved for infants less than three months of age.
- FDA/EMEA pediatric/adolescent dose: 3 months to 16 years of age: 8 mg/kg of body weight twice daily, maximum dose 300 mg twice daily.
- FDA/EMEA adult dose (>12 years): 300 mg twice daily or 600 mg once daily.

1.2 Non FDA/EMEA-Approved PENPACT 1 Dosing Recommendations

Children < 3 months of age: Abacavir has been studied in children ≥ 30 days and children ≤ 2 years of age through PACTG 356. In this study, abacavir dosage was 8 mg/kg po q12h. PACTG 356 is an ongoing study that is evaluating antiretroviral activity and the durability of viral suppression using early intensive antiretroviral combination therapy in HIV-1-infected infants and children. In cohorts 3 and 4, infants ≥ 30 days and children ≤ 2 years of age received quadruple therapy; abacavir, zidovudine, lamivudine and nevirapine.

Once daily dosing: Once daily dosing of abacavir was studied in children aged 2-12 in PENTA 13. In this cross-over PK study, the AUC0-24 and C_{max} of abacavir given 1 6mg/kg po q24h were not inferior to q12h dosing. Virological data did not indicate a marked difference in antiviral activity between q12h and q24h regimens.

Once daily dosing of abacavir can be considered for children enrolled in PENPACT 1 who have reached a stable nadir in viral load (ideally <50 copies/ml). We would not recommend starting therapy with once daily abacavir in children, particularly where the initial viral load is high. It is important to ensure that once daily doses are given at approximately the same time each day (optimally within an hour either side of a given time).

APPENDIX IX

1.3 Major Toxicities

In ongoing clinical trials, hypersensitivity reactions have been reported in approximately 5% of pediatric patients receiving abacavir. Fatal hypersensitivity reactions have been associated with therapy with abacavir in adults. Refer to the manufacturer's product information for the management of subjects, and to Appendix X for management of any hypersensitivity reaction. Lactic acidosis and severe hepatomegaly with steatosis, including fatal cases, have been reported with the use of nucleoside analogues alone or in combination, including abacavir and other antiretrovirals. The caregiver (subject) should receive the warning card and medication guide that are included in the manufacturers packaging. As the subject is enrolled the study the site must ensure that:

1) the caregiver (subject) receives the abacavir warning card,
2) the designated health care provider reviews the signs and symptoms of hypersensitivity reaction with the caregiver (subject),
3) the caregiver (subject) verbalizes an understanding of the steps to take in the event of a suspected hypersensitivity reaction, including when and how to contact the study site.

References:

- **Luzuriaga K, McManus M, Mofenson L, Britto P, Graham B, Sullivan JL; PACTG 356 Investigators. A trial of three antiretroviral regimens in HIV-1-infected children. N Engl J Med. 2004 Jun 10;350(24):2471-80.**
- Luzuriaga K, McManus M, Catalina M, Mayack S, Sharkey M, Stevenson M, Sullivan JL. Early therapy of vertical human immunodeficiency virus type 1 (HIV-1) infection: control of viral replication and absence of persistent HIV-1-specific immune responses. J Virol. 2000 Aug;74(15):6984-91.
- **Bergshoeff A, Burger D, Verweij C, Farrelly L, Flynn J, Le Prevost M, Walker S, Novelli V, Lyall H, Khoo S, Gibb D; PENTA-13 Study Group. Plasma pharmacokinetics of once- versus twice-daily lamivudine and abacavir: simplification of combination treatment in HIV-1-infected children (PENTA-13). Antivir Ther. 2005;10(2):239-46.**

APPENDIX IX

2.0 AMPRENAVIR (**APV,** AGENERASE®)

2.1 Current Preparations and Dosages Including FDA/EMEA Approved Dosing Guidelines

Antiretroviral Class: PI

Preparations:

- Pediatric oral solution: 15 mg/mL
- Capsules: 50 and 150 mg

Dosages:

- Neonatal dose: There are currently no data for <3 years of age.
- FDA pediatric/adolescent dose (<50 kg): For children 4 to 12 years of age or 13 to 16 years old weighing less than 50 kg: Oral solution: 22.5 mg/kg twice daily or 17 mg/kg three times daily (maximum daily dose 2800 mg). Capsules: 20 mg/kg twice daily or 15 mg/kg three times daily (maximum daily dose 2400 mg).
- FDA adolescent dose (≥50 kg): For children 13 to 16 years of age weighing more than 50 kg: Oral solution: 1400 mg bid.
- FDA/EMEA adult dose: 1200 mg (eight 150 mg capsules) bid.

2.2 Major Toxicities

Amprenavir has been generally well tolerated in clinical studies. Vomiting, nausea, diarrhea, perioral paresthesias, and rash have been reported. Life-threatening rash, including Stevens-Johnson syndrome has been seen in 1% of patients. Other less common side effects have included increased cholesterol levels, new onset diabetes mellitus, hyperglycemia, exacerbation of pre-existing diabetes mellitus, hemolytic anemia, and spontaneous bleeding in hemophiliacs. The FDA approved oral solution formulation of amprenavir contains significant amounts of propylene glycol and therefore is not suitable for use in infants.

APPENDIX IX

3.0 ATAZANAVIR (ATV, REYATAZ®)

3.1 Current Preparations and Dosages Including FDA/EMEA Approved Dosing Guidelines

Antiretroviral Class: PI

Preparations:

- **Capsules: 100 mg, 150 mg, 200 mg and 300 mg**

Dosages:

FDA dosing recommendations:

- **Adolescent (≥ 16 years)/adult dose:**

  Antiretroviral-naïve patients: 400 mg once daily. Low-dose ritonavir boosting may be considered for adolescents because adequacy of the unboosted atazanavir adult dose for adolescents has not been established.

  Antiretroviral-experienced patients: 300 mg atazanavir with 100 mg ritonavir once daily.

- **Atazanavir in combination with efavirenz (adults): 300 mg atazanavir with 100 mg ritonavir and 600 mg efavirenz, all once daily. Only atazanavir boosted with ritonavir should be used in combination with efavirenz.**
- **Atazanavir in combination with tenofovir DF (adults): 300 mg atazanavir with 100 mg ritonavir and 300 mg tenofovir DF, all once daily. Only atazanavir boosted with ritonavir should be used in combination with tenofovir DF.**

EMEA dosing recommendations:

- **Adults: 300 mg once daily taken with ritonavir 100 mg once daily and with food. Ritonavir is used as a booster of atazanavir pharmacokinetics.**
- **Atazanavir in combination with efavirenz (adults): 400 mg atazanavir with 100 mg ritonavir and 600 mg efavirenz, all once daily.**
- **If Reyataz® with ritonavir is co-administered with didanosine, it is recommended that didanosine be taken 2 hours after Reyataz® with ritonavir taken with food.**

APPENDIX IX

3.2 Non FDA/EMEA-Approved PENPACT 1 Dosing Recommendations

Pediatric/adolescent dose (>2 years to ≤21 years) recommendation from P1020A: 205 mg/m^2 atazanavir plus 100 mg/m^2 ritonavir (maximum 100 mg).

3.3 Major Toxicities

More common: Asymptomatic elevations in indirect bilirubin (30% of patients), jaundice (10% of patients), headache, fever, arthralgia, depression, insomnia, dizziness, nausea, vomiting, diarrhea, and paresthesias. Less common (more severe): Prolongation of PR interval of electrocardiogram. Abnormalities in AV conduction, generally limited to first-degree AV block, but with rare reports of second-degree AV block. Rash, generally mild to moderate, but in rare cases includes life-threatening Stevens-Johnson syndrome. Fat redistribution and lipid abnormalities may be less common than with other PIs. Rare: New onset diabetes mellitus, hyperglycemia, ketoacidosis, exacerbation of pre-existing diabetes mellitus, spontaneous bleeding in hemophiliacs, and elevation in serum transaminases.

References:

- **R Rutstein, P Samson, J Kiser, C Fletcher, B Graham, S Schnittman, M Smith, L Mofenson, T Fenton, G Aldrovandi and the PACTG P1020A Study Team. The PACTG 1020A Protocol: Atazanavir with or without ritonavir in HIV-infected infants, children and adolescents. 14th Conference on Retroviruses and Opportunistic Infections, February 2007. Abstract #715.**
- **Rutstein R, Samson P, Fenton T, Schnittman S, Fletcher C, Kiser J, Smith ME. Effect of atazanavir on serum cholesterol and triglyceride levels in HIV-infected infants, children and adolescents: Pediatric AIDS Clinical Trials Group 1020A. AIDS 2007. In Press.**
- **P1020A "A Phase I/II Open Label Pharmacokinetic and Safety Study of A Novel Protease Inhibitor (BMS 232632) in Combination Regimens in ART Naive and Experienced HIV-infected Infants, Children and Adolescents, Version 5.0, September 23, 2003 available at http://impaact.s-3.com/members/ps/1020a/ps1020a.htm (username *impaact*, password *cure*)**

APPENDIX IX

4.0 **DARUNAVIR (DRV, PREZISTA®)**

4.1 **Current Preparations and Dosages Including FDA/EMEA Approved Dosing Guidelines**

Antiretroviral Class: PI

Preparations:

- **Tablet: 300 mg**

Dosages:

- **Pediatric dose: The safety and efficacy of darunavir in pediatric patients has not been established.**
- **Adolescent (≥18 years)/adult dose: 600 mg twice daily taken with ritonavir 100 mg twice daily and taken with food.**

4.2 **Major Toxicities**

More common: Diarrhea, nausea, vomiting, abdominal pain, headache, and fatigue. Less common: Skin rash, including erythema multiforme and Stevens-Johnson syndrome, has been reported. Fever and elevated hepatic transaminases have been reported. Lipid abnormalities have been reported.

5.0 DIDANOSINE (**DDI**, VIDEX® AND VIDEX EC®, FDA-APPROVED GENERIC DIDANOSINE)

5.1 Current Preparations and Dosages Including FDA/EMEA Approved Dosing Guidelines

Antiretroviral Class: NRTI

Preparations:

- Pediatric powder for oral solution (when reconstituted as solution containing antacid, 2 g and 4 g bottles): 10 mg/mL (not licensed in Europe).
- Chewable tablets with buffers: 100 mg
- Delayed-release capsules (enteric-coated beadlets): Videx EC®: 125, 200, 250, and 400 mg.

APPENDIX IX

Dosages:

- Neonatal dose (infants aged <90 days): 50 mg per m^2 of body surface area every 12 hours.
- FDA pediatric usual dose (Accepted for use in Europe by recognition of FDA approval): In combination with other antiretrovirals, 120 mg per m^2 of body surface area every 12 hours.
- Pediatric dosage range use in clinical setting: 90 to 150 mg per m^2 of body surface area every 12 hours.
- Note: May need higher dose in patients with central nervous system disease.
- FDA adolescent/adult dose for chewable tablets with buffers (Accepted for use in Europe by recognition of FDA approval): Body weight ≥60 kg: 200 mg twice daily. Body weight <60 kg: 125 mg twice daily.

Note: May be administered once daily in adolescents/adults to improve compliance or for other therapeutic reasons; however, twice daily dosing provides better therapeutic response than once daily dosing.

- FDA adult dose for didanosine as delayed release capsules (Accepted for use in Europe by recognition of FDA approval): Body weight ≥60 kg; 400 mg once daily for capsules. Body weight <60 kg; 250 mg once daily for capsules.
- FDA adult dose for didanosine as buffered powder for oral solution (This dosing is accepted for use in Europe by recognition of FDA approval): Body weight ≥60 kg; 250 mg BID for buffered powder for oral solution. Body weight <60 kg; 167 mg BID for buffered powder for oral solution.

5.2 Major Toxicities

Didanosine can cause diarrhea, abdominal pain, nausea, and vomiting.
Other reported side effects include peripheral neuropathy (dose related), electrolyte abnormalities, and hyperuricemia. Rarely, pancreatitis (dose related, less common in children than adults), increased liver enzymes, and retinal de-pigmentation and optic neuritis have been reported.

APPENDIX IX

Reference

Working Group on Antiretroviral Therapy and Medical Management of HIV-Infected Children. Guidelines for the Use of Antiretroviral Agents in Pediatric HIV Infection. October 26, 2006 1-126. Available at http://aidsinfo.nih.gov/ContentFiles/PediatricGuidelines.pdf.

6.0 EFAVIRENZ (**EFV,** SUSTIVA®)

6.1 Current Preparations and Dosages Including FDA/EMEA Approved Dosing Guidelines

Antiretroviral Class: NNRTI

Preparations:

- Capsules: 50, 100 and 200 mg
- Tablet: 600 mg
- Oral solution (EMEA approval): **30 mg/mL**
- **Tablets in combination with emtricitabine and tenofovir DF (FDA approval): Atripla®: 600 mg efavirenz/200 mg emtricitabine/300 mg tenofovir DF**

Dosage:

- FDA/EMEA neonatal dose: Unknown.
- FDA/EMEA pediatric **(> 3 years)** dose: There are currently no data available on the appropriate dosage for children under age three years. For children 3 years of age and older, it should be administered once daily for body weight, as follows:

  FDA 10 to <15 kg: 200 mg
  EMEA 13 to <15 kg: 200 mg
  15 to <20 kg: 250 mg
  20 to 25 kg: 300 mg
  25 to <32.5 kg: 350 mg
  32.5 to <40 kg: 400 mg
  ≥40 kg: 600 mg

APPENDIX IX

- EMEA dose: Oral solution for children 3 years of age and older, it should be administered once daily for body weight, as follows:

Body Weight (kg)	Children ≥3 and < 5 years of age	Children ≥5 years of age
13 to <15	12mL od (360mg)	9mL (270mg)
15 to <20	13 mL (390 mg)	10mL (300 mg)
20 to < 25	15 mL (450 mg)	12mL (360 mg)
25 to < 32.5	17 mL (510 mg)	15 mL (450 mg)
32.5 to <40	-	17 mL (510 mg)
40 -	-	24 mL (720 mg)

- FDA/EMEA adult dose: 600 mg once daily (EFV as a 600 mg tablet can be taken once daily, in combination with a PI and/or NRTIs).
- FDA adult (**≥18 years**) dose: **Atripla® one tablet once daily. Dosing at bedtime may improve the tolerability of nervous system symptoms.**

6.2 Major Toxicities

Treatment with efavirenz has been associated with skin rash and increased aminotransferase levels. In some patients, central nervous system effects (somnolence, insomnia, abnormal dreams, confusion, abnormal thinking, impaired concentration, amnesia, agitation, depersonalization, hallucinations, and euphoria), have been reported. The central nervous system effects have been seen primarily in adults. Efavirenz is teratogenic in primates (use in pregnancy should be avoided and women of childbearing potential should undergo pregnancy testing before initiating therapy).

Because Atripla® is a fixed-dose combination, it should not be prescribed for patients requiring dosage adjustment or those with creatinine clearance <50 mL/min.

7.0 EFAVIRENZ/EMTRICITABINE/TENOFOVIR DISOPROXIL FUMARATE (ATRIPLA®)

5.1 Current Preparations and Dosages Including FDA Approved Dosing Guidelines

Atripla® is not EMEA licensed.

Antiretroviral Class: NNRTI, NRTI, NtRTI

Preparations:

- **Tablets: 600 mg efavirenz/200 mg emtricitabine/300 mg tenofovir DF**

Dosages

- **FDA adult (≥18 years) dose: Atripla® one tablet once daily. Dosing at bedtime may improve the tolerability of nervous system symptoms.**

5.2 **Major Toxicities:**

Refer to entries for efavirenz, emtricitabine and tenofovir DF. Because Atripla® is a fixed-dose combination, it should not be prescribed for patients requiring dosage adjustment or those with creatinine clearance <50 mL/min.

8.0 EMTRICITABINE (FTC, EMTRIVA®)

8.1 Current Preparations and Dosages Including FDA/EMEA Approved Dosing Guidelines

Antiretroviral Class: NRTI

Preparations:

- Capsules: 200 mg
- Oral suspension: 10 mg/ml
- Tablets in combination with tenofovir disoproxil fumarate: Truvada®: 200 mg emtricitabine, 300mg tenofovir disoproxil fumarate
- **Tablet in combination with efavirenz and tenofovir DF (FDA approval): Atripla®: 600 mg efavirenz/200 mg emtricitabine/300 mg tenofovir DF**

Dosages

- FDA/EMEA adult dose: 200 mg once daily.
- FDA/EMEA adult dose of Truvada®: one tablet daily
- **FDA adult (≥18 years) dose: Atripla® one tablet once daily. Dosing at bedtime may improve the tolerability of nervous system symptoms.**

8.2 Non FDA/EMEA Approved PENPACT 1 Dosing Recommendations

Suggested pediatric dose 6 mg/kg (maximum dose 200 mg).

8.3 Major Toxicities:

APPENDIX IX

Adverse effects in patients treated with emtricitabine have included headache, dizziness, tiredness, inability to sleep, unusual dreams, loose or watery stools, nausea or vomiting, abdominal pain, rash, itching, allergic reactions, skin darkening of the palms and/or soles, and increased LFTs, amylase, triglycerides or CPK. Lactic acidosis and severe hepatomegaly with steatosis, including fatal cases, have been reported with the use of nucleoside analogues alone or in combination, including emtricitabine and other antiretrovirals in adults.

Because Atripla® is a fixed-dose combination, it should not be prescribed for patients requiring dosage adjustment or those with creatinine clearance <50 mL/min.

References:

- **McKinney RE Jr, Rodman J, Hu C, Britto P, Hughes M, Smith ME, Serchuck LK, Kraimer J, Ortiz AA, Flynn P, Yogev R, Spector S, Draper L, Tran P, Scites M, Dickover R, Weinberg A, Cunningham C, Abrams E, Blum MR, Chittick GE, Reynolds L, Rathore M, Pediatric AIDS Clinical Trials Group Protocol P1021 Study Team. Long-term safety and efficacy of a once-daily regimen of emtricitabine, didanosine and efavirenz in HIV-infected, therapy-naïve children and adolescents: Pediatric AIDS Clinical Trials Group Protocol P1021. Pediatrics. 2007 Aug;120(2):e416-23. Epub 2007 Jul 23.**
- P1021 "An Open-Label Study to Evaluate the Safety, Tolerance, Anti-Viral-Activity and Pharmacokinetics of Emtricitabine in Combination with Efavirenz and Didanosine in a Once Daily Regimen in HIV Infected Antiretroviral Therapy Naïve or Very Limited Antiretroviral Exposed Pediatric Subjects", **Version 2.0, December 22, 2004 available at http://impaact.s-3.com/MEMBERS/ps/1021/ps1021.htm** (username *impaact*, password *cure*)

APPENDIX IX

9.0 FOSAMPRENAVIR CALCIUM (FPV, LEXIVA®, TELZIR®)

9.1 Current Preparations and Dosages Including FDA/EMEA Approved Dosing Guidelines

Antiretroviral Class: PI

Preparations:

- **Oral suspension: 50 mg/mL**
- **Tablet: 700 mg**

Dosages:

The Protocol Team recommends that fosamprenavir be boosted with ritonavir.

FDA dosing recommendations:

- **Adolescent (≥ 18 years)/therapy-naive adult dose:**

  Fosamprenavir 1400 mg twice daily (without ritonavir); fosamprenavir 1400 mg once daily plus ritonavir 200 mg once daily; fosamprenavir 700 mg twice daily plus ritonavir 100 mg twice daily.

- **Protease inhibitor-experienced adult dose: Fosamprenavir 700 mg twice daily plus ritonavir 100 mg twice daily.**

- **Pediatric Patients (2 to 18 years of age): The recommended dosage of fosamprenavir calcium in patients > 2 years of age should be calculated based on body weight (kg) and should not exceed the recommended adult dose. The data are insufficient to recommend: (1) once-daily dosing of fosamprenavir calcium alone or in combination with ritonavir, and (2) any dosing of fosamprenavir calcium in therapy-experienced patients 2 to 5 years of age.**

  Therapy-naïve 2 to 5 years of age: Fosamprenavir calcium oral suspension 30 mg/kg twice daily, not to exceed the adult dose of fosamprenavir calcium 1,400 mg twice daily.

  Therapy-naïve ≥ 6 years of age: Either fosamprenavir calcium oral suspension 30 mg/kg twice daily not to exceed the adult dose of fosamprenavir calcium 1,400 mg twice daily or fosamprenavir calcium oral suspension 18 mg/kg plus ritonavir 3 mg/kg twice daily not to exceed the adult dose of fosamprenavir calcium 700 mg plus ritonavir 100 mg twice daily.

  Therapy-experienced ≥ 6 years of age: Fosamprenavir calcium oral

suspension 18 mg/kg plus ritonavir 3 mg/kg administered twice daily not to exceed the adult dose of fosamprenavir calcium 700 mg plus ritonavir 100 mg twice daily.

When administered without ritonavir, the adult regimen of fosamprenavir tablets 1400 mg twice daily may be used for pediatric patients weighing at least 47 kg.

When administered in combination with ritonavir, fosamprenavir tablets may be used for pediatric patients weighing at least 39 kg; ritonavir capsules may be used for pediatric patients weighing at least 33 kg.

Patients with Hepatic Impairment:

- **Mild hepatic impairment (Child-Pugh score ranging from 5 to 6): Fosamprenavir calcium should be used with caution at a reduced dosage of 700 mg twice daily without ritonavir (therapy-naïve) or 700 mg twice daily plus ritonavir 100 mg once daily (therapy-naïve or PI-experienced).**

- **Moderate hepatic impairment (Child-Pugh score ranging from 7 to 9): Fosamprenavir calcium should be used with caution at a reduced dosage of 700 mg twice daily (therapy-naïve) without ritonavir, or 450 mg twice daily plus ritonavir 100 mg once daily (therapy-naïve or PI-experienced).**
- **Severe hepatic impairment (Child-Pugh score ranging from 10 to 12): Fosamprenavir calcium should be used with caution at a reduced dosage of 350 mg twice daily without ritonavir (therapy-naïve). There are no data on the use of fosamprenavir calcium in combination with ritonavir in patients with severe hepatic impairment.**

EMEA dosing recommendations:

- **Adults ($\geq$18 years): For antiretroviral naïve and experienced patients the recommended dose is 700 mg fosamprenavir twice daily with 100 mg ritonavir twice daily, in combination with other antiretroviral medicinal products. Caution is advised if the recommended doses of fosamprenavir with ritonavir detailed above are exceeded.**
- **Children (<12 years) and adolescents (12 to 17 years): Fosamprenavir with ritonavir is not recommended for use in children below age 12 and adolescents age 12 to 17 due to lack of data on safety and efficacy.**

APPENDIX IX

9.2 **Major Toxicities**

More common: Vomiting, nausea, diarrhea, perioral paresthesias, headache, rash, and lipid abnormalities. Less common (more severe): Life-threatening rash, including Stevens-Johnson syndrome, in < 1% of patients. Fat redistribution, neutropenia, and elevated serum creatinine kinase levels. Rare: New onset diabetes mellitus, hyperglycemia, ketoacidosis, exacerbation of pre-existing diabetes mellitus, spontaneous bleeding in hemophiliacs, hemolytic anemia, and elevation in serum transaminases.

10.0 INDINAVIR (**IDV,** CRIXIVAN®)

10.1 Current Preparations and Dosages Including FDA/EMEA Approved Dosing Guidelines

Antiretroviral Class: PI

Preparations: Capsules: 100, 200, 333 and 400 mg

Dosages:

- Neonatal dose: Unknown. Due to side effect of hyperbilirubinemia, should not be given to neonates until further information is available.
- Routinely used pediatric dose in clinical setting: 500 mg per m^2 of body surface area every eight hours. However, children with small body surface areas: 300-400 mg/m^2 every 8 hours.
- FDA adult dose: 800 mg every eight hours.
- The EMEA recommended dosage for children and adolescents (4 to 17 years of age) is 500 mg per m^2 (dose adjusted by BSA) every eight hours (maximum dose of 800 mg every eight hours).
- EMEA adult dose: 800 mg every eight hours.

10.2 PENPACT 1 Dosing Recommendations

Dosing recommendations for children: The indinavir dose used in PACTG 395 Version 2.0 **was** 500 mg/m^2/dose given orally q8h. An oral formulation for administration to younger children and infants, particularly those not able to swallow capsules and who require doses less than 100 mg, is not available at this time.

10.3 PENPACT 1 Dosing Recommendations of Indinavir (Crixivan®) given with

APPENDIX IX

Ritonavir (Norvir®)

Ritonavir increases indinavir and reduces the intersubject variability of indinavir plasma concentrations.

Dosing for adults and adolescents: Indinavir in combination with ritonavir 800 mg IDV + 200 mg RTV twice daily.

10.4 Major Toxicities

Adverse effects include changes in hematologic and hepatic function tests, nephrolithiasis (kidney stones), abdominal pain, asthenia/fatigue, diarrhea, headache, dizziness, insomnia, nausea, back and side pain, rash, vomiting, acid regurgitation (backflow of stomach acid to the esophagus), and taste perversion. Indirect hyperbilirubinemia has occurred frequently with indinavir administration and has sometimes been associated with increases in serum transaminases. Both hyperbilirubinemia and nephrolithiasis occurred more frequently at doses exceeding 2.4 g/day compared to doses = 2.4 g/day. Adequate hydration (comparable to at least 1.5 liters/adult/24 hours) is required in all patients treated with indinavir.

References:

- Hsu A, Granneman GR, Cao G, Carothers L, Japour A, El-Shourbagy T, Dennis S, Berg J, Erdman K, Leonard JM, Sun E. Pharmacokinetic interaction between ritonavir and indinavir in healthy volunteers. Antimicrob Agents Chemother. 1998 Nov;42(11):2784-91.
- Saah AJ, Winchell GA, Nessly ML, Seniuk MA, Rhodes RR, Deutsch PJ. Pharmacokinetic profile and tolerability of indinavir-ritonavir combinations in healthy volunteers. Antimicrob Agents Chemother. 2001 Oct;45(10):2710-5.
- EG Chadwick E, JH Rodman, P Sampson, T Fenton, EA Abrams, B Nowak, SI Pelton, S Lavoie, K Knapp, M Bambji, R Yogev for the PACTG 1013 Team. Antiviral activity, tolerance and pharmacokinetics of indinavir with two doses of ritonavir as salvage therapy in children. 10th Conference on Retroviruses and Opportunistic Infections, Boston, Massachusetts, February 2003. Abstract # 875.

APPENDIX IX

11.0 LAMIVUDINE (**3TC,** EPIVIR®)

11.1 Current Preparations and Dosages Including FDA/EMEA Approved Dosing Guidelines

Antiretroviral Class: NRTI

Preparations:

- Solution: 10 mg/mL
- Tablets: 150 and 300 mg
- Tablets in combination with zidovudine: Combivir®: 300 mg zidovudine, 150 mg lamivudine
- Tablets in combination with abacavir: Epzicom®/Kivexa: 600 mg abacavir, 300 mg lamivudine

Dosages:

- Routinely used neonatal dose in clinical setting (infants aged <30 days): 2 mg per kg of body weight twice daily.
- EMEA adolescents aged >12 years: 30 mL or 300 mg once a day, or 15 mL or 150 mg BID.
- FDA adolescent/adult aged > 16 years of age dose: 300 mg once daily or 150 mg twice daily. Maximum dose is 150 mg twice daily.
- FDA/EMEA pediatric/adolescent dose: 3 months to 16 years of age: 4 mg per kg of body weight twice daily. Maximum dose is 150 mg twice daily.
- FDA/EMEA adolescent/adult dose of Combivir®: one tablet twice daily.

11.2 PENPACT 1 Dosing Recommendations

Dosing in infants <3 months of age: Lamivudine at 4mg/kg po q12h has been used in PACTG 356 and PACTG 345 for infants >29 days of life to 2 years of age.

Once daily dosing in children under 13: once daily dosing of lamivudine was studied in children aged 2 to 12 **years** in PENTA 13. In this cross-over PK study, the AUC_{0-24} and C_{max} of lamivudine given 8mg/kg po q24h were not inferior to q12h dosing. Virological data did not indicate a marked difference in antiviral activity between q12h and q24h regimens.

Once daily dosing of lamivudine can be considered for children enrolled in

PENPACT 1 who have reached a stable nadir in viral load (ideally <50 copies/mL). We would not recommend starting therapy with once daily lamivudine in children, particularly where the initial viral load is high. It is important to ensure that once daily doses are given at approximately the same time each day (optimally within an hour either side of a given time).

11.3 Major Toxicities

Pancreatitis, which has been fatal in some cases has been observed in antiretroviral nucleoside-experienced pediatric patients receiving lamivudine alone or in combination with other antiretroviral agents. In pediatric studies paresthesias and peripheral neuropathies were reported in <1% to 15% of patients. Lactic acidosis and severe hepatomegaly with steatosis, including fatal cases, have been reported with the use of nucleoside analogues alone or in combination, including lamivudine and other antiretrovirals in adults.

References:

- **Chadwick EG, Rodman JH, Britto P, Powell C, Palumbo P, Luzuriaga K, Hughes M, Abrams EJ, Flynn PM, Borkowsky W, Yogev R; PACTG Protocol 345 Team. Ritonavir-based highly active antiretroviral therapy in human immunodeficiency virus type 1-infected infants younger than 24 months of age. Pediatr Infect Dis J. 2005 Sep;24(9):793-800.**
- **Luzuriaga K, McManus M, Mofenson L, Britto P, Graham B, Sullivan JL; PACTG 356 Investigators. A trial of three antiretroviral regimens in HIV-1-infected children. N Engl J Med. 2004 Jun 10;350(24):2471-80.**
- Luzuriaga K, McManus M, Catalina M, et al. Early therapy of vertical human immunodeficiency virus type 1 (HIV-1) infection: control of viral replication and absence of persistent HIV-1-specific immune responses. J Virol (United States), Aug 2000, 74(15) p6984-91.
- **Bergshoeff A, Burger D, Verweij C, Farrelly L, Flynn J, Le Prevost M, Walker S, Novelli V, Lyall H, Khoo S, Gibb D; PENTA-13 Study Group. Plasma pharmacokinetics of once- versus twice-daily lamivudine and abacavir: simplification of combination treatment in HIV-1-infected children (PENTA-13). Antivir Ther. 2005;10(2):239-46.**

APPENDIX IX

12.0 LOPINAVIR/RITONAVIR (**LPV/R,** KALETRA®)

12.1 Current Preparations and Dosages Including FDA/EMEA Approved Dosing Guidelines

Co-formulation of lopinavir and ritonavir: RTV acts as a pharmacokinetic enhancer, not as an antiretroviral agent. It does this by inhibiting the metabolism of lopinavir and increasing lopinavir plasma concentrations.

Antiretroviral Class: PI

Preparations:

- Pediatric oral solution: 80 mg lopinavir and 20 mg ritonavir per mL
- **Film-coated tablets**: **200 mg** lopinavir/**50** mg ritonavir

Dosages:

- Neonatal dose: **300 mg/m^2 LPV/r twice daily has been studied in PACTG P1030.**
- FDA pediatric dose for individuals not receiving concomitant nevirapine or efavirenz: 230 mg per m^2 lopinavir/57.5 mg per m^2 ritonavir twice daily with food, up to a maximum of 400 mg lopinavir/100 mg ritonavir. These subjects can also be dosed as per the following table:

Six months to 12 years of age (Dosing without NVP or EFV)	
7 to <15 kg	12 mg per kg lopinavir/3 mg per kg ritonavir twice daily with food.
15 to 40 kg	10 mg per kg lopinavir/2.5 mg per kg ritonavir twice daily with food.
>40 kg	400 mg lopinavir/100 mg ritonavir (**two tablets** or 5 mL **solution**) twice daily with food (same as adult dose).

- FDA adult/adolescent dose not receiving concomitant nevirapine or efavirenz: 400 mg lopinavir/100 mg ritonavir (**two tablets** or 5 mL **solution**) twice daily with food.
- FDA pediatric dose for individuals receiving concomitant nevirapine or efavirenz **(which induces lopinavir metabolism, reduces plasma levels and requires increased lopinavir/ritonavir dosing)** and/or treatment experienced patients where reduced susceptibility to lopinavir is suspected **(such as those with prior treatment with other PIs):** 300 mg per m^2 lopinavir/75 mg per m^2 ritonavir twice daily with food up to a maximum of **600** mg lopinavir/**150** mg ritonavir (three **tablets** or 6.5 mL **solution**) twice daily with food. These

APPENDIX IX

subjects can also be dosed as per the following table:

Six months to 12 years of age (Dosing giving with NVP or EFV)	
7 to <15 kg	13 mg per kg lopinavir/3.25 mg per kg ritonavir twice daily with food.
15 to 50 kg	11 mg per kg lopinavir/2.75 mg per kg ritonavir twice daily with food.
>50 kg	**600** mg lopinavir/**150** mg ritonavir (three **tablets** or 6.5 mL **solution**) twice daily with food (same as adult dose).

- The EMEA does not recommend Kaletra® for use in children less than 2 years of age because of limited efficacy and safety data.
- FDA adult/adolescent dose receiving concomitant nevirapine or efavirenz: 533 mg lopinavir/133 mg ritonavir (4 capsules or 6.5 mL **solution**) twice daily with food.
- EMEA pediatric dose (2 years of age and above) (covers adolescent/adult dosing): the recommended dosage of Kaletra® for children with a body surface area of 1.3 m^2 or greater, is 3 capsules twice daily taken with food (maximum 400 mg lopinavir/100 mg ritonavir).

Notes:
- For children with a body surface area of less than 1.3 m^2, Kaletra® oral solution is recommended.
- Body surface area can be calculated with the following equation:

$$BSA\ (m^2) = \sqrt{(\text{Height (cm)} \times \text{Weight (kg)} / 3600)}$$

Clarification Notes:
- Although pediatric clinical trials utilized the mg per m^2 body surface area dosing, the FDA-approved doses are based on a mg per kg body weight dosage. The 230 mg per m^2 lopinavir/57.5 mg per m^2 ritonavir twice daily regimen without nevirapine or efavirenz and the 300 mg per m^2 lopinavir/75 mg per m^2 ritonavir twice daily regimen with concomitant nevirapine or efavirenz resulted in lopinavir concentrations similar to those obtained in adults receiving the 400 mg lopinavir/100 mg ritonavir twice daily regimen (without concomitant nevirapine or efavirenz).
- The pediatric trials were done in NNRTI-naïve patients and there is little data in heavily pretreated pediatric patients. In treatment-experienced patients where reduced susceptibility to lopinavir is suspected, higher doses may be required but there is little data to make definitive dosing recommendations at this time.
- Kaletra® should be prescribed by physicians who are experienced in the treatment of HIV infection.

APPENDIX IX

12.2 Non-FDA Approved PENPACT 1 Dosing Recommendations

Infants < 6 months of age: 300 mg/m^2 LPV/r twice daily.

12.3 Non-EMEA Approved PENPACT 1 Dosing Recommendations

The FDA approved dose (230 mg/m^2 lopinavir + 57.5 mg ritonavir/m^2) for children six months to two years of age should be prescribed.

12.4 Major Toxicities

Adverse effects in patients treated with Kaletra® have included pancreatitis, elevated levels of total cholesterol and triglycerides, nausea, diarrhea, abdominal pain, rash, headache, asthenia, and ECG changes (heart block).

References:

- **J Pinto, B Robbins, J Chen, L Serchuck, E Capparelli, R Yogev, P Palumbo, J Rodman, B Heckman, E Chadwick, and PACTG P1030 Team of Investigators. Pharmacokinetics and 24-Week Efficacy and Safety of Lopinavir/Ritonavir in HIV-1-infected Infants <6 Weeks of Age. 14th Conference on Retroviruses and Opportunistic Infections, Los Angeles, California, February 2007. Abstract # 716.**
- **EG Chadwick, J Rodman, P Palumbo, D Persaud, J Chen, J Gardella, K Luzuriaga, R Yogev, P Emmanuel, M Rathore and PACTG P1030 Study Team. A Prospective Evaluation of Pharmacologic, Virologic and Immunologic Parameters of Lopinavir/Ritonavir for HIV-1-Infected Infants <6 Months of Age. 12th Conference on Retroviruses and Opportunistic Infections, February 2005. Poster #766.**
- Sáez-Llorens X, Violari A, Deetz CO, Rode RA, Gomez P, Handelsman E, Pelton S, Ramilo O, Cahn P, Chadwick E, Allen U, Arpadi S, Castrejón MM, Heuser RS, Kempf DJ, Bertz RJ, Hsu AF, Bernstein B, Renz CL, Sun E. Forty-eight-week evaluation of lopinavir/ritonavir, a new protease inhibitor, in human immunodeficiency virus-infected children. Pediatr Infect Dis J. 2003 Mar;22(3):216-24.

APPENDIX IX

13.0 NELFINAVIR (**NFV,** VIRACEPT®)

13.1 Current Preparations and Dosages Including FDA/EMEA Approved Dosing Guidelines

Any new treatment plan with nelfinavir must be reviewed with and approved by the Protocol Team before starting.

Antiretroviral Class: PI

Preparations:

- Powder for oral suspension: 50 mg per one level gram scoop full (200 mg per one level teaspoon)
- Tablets: 250 **and 625** mg

Dosages:

- FDA pediatric dose (children 2 to 13 years of age): 20 to 30 mg per kg of body weight three times a day.
- Neonatal dose routinely used in clinical setting: doses as high as 45 mg/kg every 8 hours.
- FDA adolescent/adult dose: 1250 mg (5 tablets) twice daily or 750 mg (3 tablets) three times daily.
- EMEA dose is licensed as for children ≥3 years of age: 25 to 30 mg/kg every 8 hours.

13.2 PENPACT 1 Dosing Recommendations

Alternate recommended dosing: Nelfinavir 110 mg/kg/**day** divided q12 hours was shown in PACTG 377 to provide improved serum levels in children greater than age 2.

- Dosing recommendations for children 1 to 2 years of age: A dose between 120 –150 mg/kg/day (q12) in the second year of life is advised.
- Dosing in infants < 3 months to 1 year: Infants less than 3 to 4 months old need higher doses (130-150 mg/kg/day, q12) of nelfinavir compared to older children and adults to achieve therapeutic concentrations (PENTA 7).

APPENDIX IX

13.3 Major Toxicities

Diarrhea is commonly associated with nelfinavir usage. The diarrhea generally improves after several weeks of therapy. Less common side effects include asthenia, abdominal pain, rash, and exacerbation of chronic liver disease. Rarely, nelfinavir treatment has been associated with spontaneous bleeding episodes in hemophiliacs, hyperglycemia, keto-acidosis, and diabetes.

References:

- Wiznia A, Stanley K, Krogstad P, Johnson G, Lee S, McNamara J, Moye J, Jackson JB, Mendez H, Aguayo R, Dieudonne A, Kovacs A, Bamji M, Abrams E, Rana S, Sever J, Nachman S. Combination nucleoside analog reverse transcriptase inhibitor(s) plus nevirapine, nelfinavir, or ritonavir in stable antiretroviral therapy-experienced HIV-infected children: week 24 results of a randomized controlled trial--PACTG 377. Pediatric AIDS Clinical Trials Group 377 Study Team. AIDS Res Hum Retroviruses. 2000 Aug 10;16(12):1113-21.
- Capparelli EV, Sullivan JL, Mofenson L, Smith E, Graham B, Britto P, Becker MI, Holland D, Connor JD, Luzuriaga K; Pediatric ACTG 356 Investigators. Pharmacokinetics of nelfinavir in human immunodeficiency virus-infected infants. Pediatr Infect Dis J. 2001 Aug;20(8):746-51.
- C. Litalien, C. Giaquinto, A. Faye, F. Mechinaud, I. Grosch, A. Compagnucci, E. Jacqz-Aigrain. Nelfinavir doses should be increased in infants less than 3 months. XIII International AIDS Conference, Durban, South Africa, July 2000. Abstract MoPeB2213.
- Luzuriaga K, McManus M, Catalina M, Mayack S, Sharkey M, Stevenson M, Sullivan JL. Early therapy of vertical human immunodeficiency virus type 1 (HIV-1) infection: control of viral replication and absence of persistent HIV-1-specific immune responses. J Virol. 2000 Aug;74(15):6984-91.
- Floren LC, Wiznia A, Hayashi S, Jayewardene A, Stanley K, Johnson G, Nachman S, Krogstad P, Aweeka FT; Pediatric AIDS Clinical Trials Group 377 Protocol Team. Nelfinavir pharmacokinetics in stable human immunodeficiency virus-positive children: Pediatric AIDS Clinical Trials Group Protocol 377. Pediatrics. 2003 Sep;112(3 Pt 1):e220-7.

APPENDIX IX

14.0 NEVIRAPINE (**NVP,** VIRAMUNE®, **FDA-APPROVED, GENERIC NEVIRAPINE**)

14.1 Current Preparations and Dosages Including FDA/EMEA Approved Dosing Guidelines

Antiretroviral Class: NNRTI

Preparations:

- Suspension: 10 mg/mL
- Tablet: 200 mg

Dosages:

NVP is initiated at a lower dose and increased in a step-wise fashion. This allows induction of cytochrome P450 3A that results in increased clearance of drug. The occurrence of rash may be diminished by the stepwise increase in dosage. The following suggested incremental increases in dose are given for days on treatment (not age).

- Neonatal dose (through age two months): Under study in PACTG 356: 5 mg/kg of body weight or 120 mg/m^2 of body surface area once daily for 14 days, followed by 120 mg/m^2 of body surface area every 12 hours for 14 days, followed by 200 mg/m^2 of body surface area every 12 hours.
- Pediatric dose (age ≥2 months): 120-200 mg/m^2 every 12 hours. Initiate therapy with 120 mg/m^2 (maximum 200 mg) administered once daily for 14 days. Increased to full dose (120-200 mg/m^2) administered every 12 hours (maximum 200 mg every 12 hours) if no rash or other untoward effects. These subjects can also be dosed as per the following FDA/EMEA approved dosing table:

7 mg/kg every 12 hours for ages ≥2 months to < 8 years<br>4 mg/kg every 12 hours for ages > 8 years<br><br>Note: Initiate therapy with dose of 4 mg/kg once daily for 14 days and increase to full dose if no rash or other untoward effects.

- Adolescent/adult dose: 200 mg every 12 hours. Initiate therapy with 200 mg given once daily for the first 14 days. Increase to full dose administered every 12 hours if there is no rash or other untoward effects.

APPENDIX IX

Clarification Notes:

- The majority of clinical trials involving infants and children utilized the 120 - 200 mg/m^2 dosing regimen. The new FDA approved regimen, which uses mg/kg dosing, is based on pharmacokinetic modeling designed to achieve similar plasma concentrations as dosing of 150 mg/m^2.
- NVP clearance is highest during the first two years of life, decreasing gradually after eight to 12 years of age and approaching adult clearance rates. The new dosing regimen accounts for the changes in clearance that occurs after eight years of age. However, the changes in clearance are gradual and the new mg/kg dosing regimen results in an abrupt 43% decrease in dose size when the 8th birthday is reached.
- Some clinicians may prefer the mg/m^2 dosing that was utilized in clinical trials.

14.2 PENPACT 1 Dosing Recommendations

Dosing recommendations for <2 months: Nevirapine at 120 mg/m^2 once every day for 14 days, followed by an increase to full dose nevirapine 200 mg/m^2 every 12 hours if there were no rashes of other untoward effects. was used in PACTG 356 for infants $\geq$30 days to $\leq$2 years receiving triple or quadruple therapy including nevirapine.

14.3 Major Toxicities

Severe, life-threatening, and in some cases fatal hepatotoxicity, including fulminant and cholestatic hepatitis, hepatic necrosis and hepatic failure, has been reported in patients treated with nevirapine. In some cases, patients presented with non-specific prodromal signs or symptoms of hepatitis and progressed to hepatic failure. Some events occurred after short-term exposure to nevirapine. Patients with signs or symptoms of hepatitis must seek medical evaluation immediately and should be advised to discontinue nevirapine.

Severe, life threatening skin reactions, including fatal cases, have occurred in patients treated with nevirapine. These have included cases of Stevens-Johnson syndrome, toxic epidermal necrolysis, and hypersensitivity reactions characterized by rash, constitutional findings, and organ dysfunction. Patients developing signs or symptoms of severe skin reactions or hypersensitivity reactions must discontinue nevirapine as soon as possible.

The first 12 weeks of therapy with nevirapine are a critical period during which intensive monitoring of patients is required to detect potentially life-threatening

hepatic events and skin reactions.

Nevirapine-attributable rash occurred in 16% of patients on combination regimens in Phase II/III controlled studies. Thirty-five percent of patients treated with the nevirapine-containing regimen experienced rash compared to 19% of control group patients treated with either zidovudine plus didanosine or zidovudine alone. Severe or life-threatening rash occurred in 6.6% of nevirapine-treated patients compared with 1.3% of patients treated in the control groups.

Rashes are usually mild to moderate, maculopapular erythematous cutaneous eruptions, with or without pruritis, located on the trunk, face and extremities. In adult Phase II/III clinical trials, the majority of rashes occurred within the first 6 weeks of therapy. Severe rashes occurred most frequently within the first 28 days of treatment; 25% of the patients with severe rashes required hospitalization; and one patient required surgical intervention. Overall, 7% of patients discontinued NVP due to rash.

In one clinical trial, concomitant use of prednisone to prevent nevirapine-associated rash increased the incidence and severity of rash during the first 6 weeks of NVP therapy. The use of prednisone to prevent nevirapine-associated rash is not recommended.

Subjects should be advised to promptly notify their health care provider if they develop any rash or signs and symptoms of a hypersensitivity reaction. Signs and symptoms of hypersensitivity include, but are not limited to severe rash or rash accompanied by fever, general malaise, fatigue, muscle or joint aches, blisters, oral lesions, conjunctivitis, facial edema, and/or hepatitis, eosinophilia, granulocytopenia, and renal dysfunction.

As always, please see the Viramune® package insert for additional information regarding the use of nevirapine.

References:

- Luzuriaga K, McManus M, Catalina M, Mayack S, Sharkey M, Stevenson M, Sullivan JL. Early therapy of vertical human immunodeficiency virus type 1 (HIV-1) infection: control of viral replication and absence of persistent HIV-1-specific immune responses. J Virol. 2000 Aug;74(15):6984-91.

- Verweel G, Sharland M, Lyall H, Novelli V, Gibb DM, Dumont G, Ball C, Wilkins E, Walters S, Tudor-Williams G. Nevirapine use in HIV-1-infected children. AIDS. 2003 Jul 25;17(11):1639-47.

15.0 RITONAVIR (**RTV,** NORVIR®)

15.1 Current Preparations and Dosages Including FDA/EMEA Approved Dosing Guidelines

Antiretroviral Class: PI

Preparations:

- Oral solution: 80 mg/mL
- Capsule: 100 mg

Dosages:

- FDA pediatric dose: 2 years of age and older, 400 mg/m^2 of body surface area every 12 hours with a maximum of 600 mg bid.

Note:<br>To minimize nausea/vomiting, initiate therapy starting at 250 mg/m^2 of body surface area every 12 hours and increase stepwise to full dose over five days as tolerated.

- EMEA pediatric dose: 350 mg/m^2 of body surface area every 12 hours with a maximum of 600 mg bid.
- FDA/EMEA pediatric dose range: 250 to 400 mg/m^2 of body surface area every 12 hours.
- FDA/EMEA adolescent/adult dose: 600 mg twice daily.

15.2 PENPACT 1 Dosing Recommendations

Dosing recommendations for children < 2 years: **350-450 mg/m^2 of body surface area twice daily (not to exceed 600 mg per dose). To minimize nausea/vomiting, initiate therapy starting at 250 mg/m^2 of body surface area every 12 hours and increase at 2- to 3-day intervals by 50 mg/m^2 of body surface area twice daily to full dose as tolerated. If patient does not tolerate 400 mg/m^2 of body surface area twice daily due to adverse effects, the highest tolerated dose may be used for maintenance therapy in combination with other antiretroviral agents; however, an alternative PI should be considered.**

15.3 Major Toxicities

APPENDIX IX

Most frequent are nausea, vomiting, diarrhea, headache, abdominal pain, and anorexia. Less common are circumoral paresthesias and increase in liver enzyme. Rare include spontaneous bleeding episodes in hemophiliacs, pancreatitis, increased levels of triglycerides and cholesterol hyperglycemia, ketoacidosis, diabetes and hepatitis.

References:

- Chadwick EG, Rodman JH, Britto P, Powell C, Palumbo P, Luzuriaga K, Hughes M, Abrams EJ, Flynn PM, Borkowsky W, Yogev R; PACTG Protocol 345 Team. Ritonavir-based highly active antiretroviral therapy in human immunodeficiency virus type 1-infected infants younger than 24 months of age. Pediatr Infect Dis J. 2005 Sep;24(9):793-800.

- **Working Group on Antiretroviral Therapy and Medical Management of HIV-Infected Children. Guidelines for the Use of Antiretroviral Agents in Pediatric HIV Infection. October 26, 2006. Available at http://aidsinfo.nih.gov/ContentFiles/PediatricGuidelines.pdf.**

16.0 SAQUINAVIR (**SQV,** INVIRASE®, FORTOVASE®)

16.1 Current Preparations and Dosages Including FDA/EMEA Approved Dosing Guidelines

Antiretroviral Class: PI

Preparations:

- Hard gel capsules: 200 mg
- **Film-coated tablet: 500 mg**

Dosages:

- Neonatal dose: **Not approved for use in neonates/infants.**
- **Adolescent (>16 years)/adult dose:**

  Saquinavir in combination with ritonavir 1,000 mg SQV + 100 mg RTV, both given twice daily. Should be taken within 2 hours of a meal.

  Saquinavir in combination with lopinavir/ritonavir: 1,000 mg AQV + 400 mg LPV/100 mg RTV, both given twice daily.

  Note: Saquinavir should only be used in combination with RTV or LPV/r (never unboosted).

APPENDIX IX

16.2 PENPACT 1 Dosing Recommendations

Dosing recommendations for children <16 years: ACTG 397 is a trial that evaluated two saquinavir containing regimens in children 3 to 16 years of age who weigh at least 8 kg and can swallow capsules. Saquinavir dosing is 50 mg/kg po bid up to a maximum of 1200 mg po bid when given with ritonavir (75-150 mg/m^2 BID) plus one or two NRTIs.

16.3 Major Toxicities

Saquinavir appears to be well tolerated. The most frequently reported adverse events among patients receiving saquinavir were diarrhea, abdominal discomfort and nausea. Other reactions reported in trial of saquinavir, alone and with zidovudine and zalcitabine, include the following: dyspepsia, mucosa damage, headache, paresthesia, extremity numbness, dizziness, peripheral neuropathy, musculoskeletal pain, myalgia, asthenia, appetite disturbances, rash, and pruritis.

References:

- R. C. Brundage, M. W. Kline, J. Lindsey, T. Fenton, and C. V. Fletcher for the PACTG 397 Team. Pharmacokinetics of squinavir (SQV) with nlfinavir (NFV) or rtonavir (RTV) in HIV-infected children. 8th Conference on Retroviruses and Opportunistic Infections, Chicago, Illinois, February 2001. Abstract #728.

- **Panel on Antiretroviral Guidelines for Adult and Adolescents. Guidelines for the use of antiretroviral agents in HIV-infected adults and adolescents. Department of Health and Human Services. October 10, 2006. Available at http://www.aidsinfo.nih.gov/ContentFiles/AdultandAdolescentsGL.pdf.**

APPENDIX IX

17.0 STAVUDINE (**ZDV,** ZERIT®, **ZERIT XR**®, **FDA-APPROVED GENERIC STAVUDINE)**

17.1 Current Preparations and Dosage Including the FDA/EMEA Approved Dosing Guidelines

Antiretroviral Class: NRTI

Preparations:

- Solution: 1 mg/mL
- Capsules: 15 mg, 20 mg, 30 mg, and 40 mg
- Extended release capsules: 37.5 mg, 50 mg, 75 mg, and 100 mg

Dosages:

- FDA/EMEA pediatric dose: 1 mg per kg of body weight every 12 hours (up to weight of 30 kg). Pediatric patients weighing 30 kg or greater should receive the recommended adult dosage.
- FDA/EMEA adolescent/adult dose: Body weight ≥60 kg: 40 mg twice daily, body weight <60 kg: 30 mg twice daily.
- FDA dose for extended release capsule formulation: 100 mg once daily for individuals weighing at least 60 kg and 75 mg once daily for individuals weighing less than 60 kg.

17.2 Major Toxicities:

Peripheral neuropathy, manifested by numbness, tingling or pain in hand or feet, that has been reported in patients receiving stavudine and cases of lactic acidosis/ severe hepatomegaly with steatosis (a majority of these cases have been in women) have been reported, some cases were fatal. Fatal and nonfatal pancreatitis has occurred during therapy when stavudine was part of a combination regimen that included didanosine, with or without hydroxyurea, in both treatment naïve and treatment experienced patients regardless of degree of immunosuppression.

Cases of lactic acidosis and Gullian-Barre syndrome (sometime fatal) have been associated with regimens containing stavudine.

APPENDIX IX

Reference:

Wade NA, Unadkat JD, Huang S, Shapiro DE, Mathias A, Yasin S, Ciupak G, Watts DH, Delke I, Rathore M, Hitti J, Frenkel L, Samelson R, Smith ME, Mofenson L, Burchett SK. Pharmacokinetics and safety of stavudine in HIV-infected pregnant women and their infants: Pediatric AIDS Clinical Trials Group protocol 332. J Infect Dis. 2004 Dec 15;190(12):2167-74.

18.0 TENOFOVIR DISOPROXIL FUMURATE (**TDF,** VIREAD®)

18.1 Current Preparations and Dosages Including FDA/EMEA Approved Dosing Guidelines

Antiretroviral Class: NtRTI

Preparations:

- Tablets: 300 mg (245mg of tenofovir)
- Tablets in combination with tenofovir disoproxil fumarate: Truvada®, 200 mg emtricitabine, 300mg tenofovir disoproxil fumarate
- **Tablet in combination with efavirenz and tenofovir DF: Atripla®: 600 mg efavirenz/200 mg emtricitabine/300 mg tenofovir DF**

Dosages:

- FDA/EMEA adult dose: 300 mg once daily.
- FDA/EMEA adult dose of Truvada®: one tablet daily.
- **FDA adult (≥18 years) dose of Atripla®: One tablet once daily. Dosing at bedtime may improve the tolerability of nervous system symptoms. Renal impairment: Because Atripla® is a fixed-dose combination, it should not be prescribed for patients requiring dosage adjustment such as those with creatinine clearance <50 mL/min.**

18.2 Non FDA/EMEA Approved PENPACT 1 Dosing Recommendations

Children aged 2 to 8 years: 8 mg/kg of body weight once daily. Children aged >8 years: Median dose of 210 mg/m^2 of body surface area once daily, maximum dose of 300 mg once daily.

APPENDIX IX

18.3 Major Toxicities:

More common: Nausea, diarrhea, vomiting, and flatulence. Less common (more severe): Lactic acidosis and severe hepatomegaly with steatosis, including fatal cases, have been reported. TDF caused bone toxicity (osteomalacia and reduced bone density) in animals when given in high doses. Decreases in bone mineral density have been shown in both adults and children taking TDF for 48 weeks; the clinical significance of these changes is not yet known. Evidence of renal toxicity, including increases in serum creatinine, blood urea nitrogen, glycosuria, proteinuria, phosphaturia, and/or calciuria and decreases in serum phosphate has been observed in animal studies at high exposure levels. Several cases of renal tubular dysfunction have been reported in patients receiving TDF; patients at increased risk of renal dysfunction should be closely monitored.

References:

- Hazra R, Balis FM, Tullio AN, DeCarlo E, Worrell CJ, Steinberg SM, Flaherty JF, Yale K, Poblenz M, Kearney BP, Zhong L, Coakley DF, Blanche S, Bresson JL, Zuckerman JA, Zeichner SL. Single-dose and steady-state pharmacokinetics of tenofovir disoproxil fumarate in human immunodeficiency virus-infected children. Antimicrob Agents Chemother. 2004 Jan;48(1):124-9.
- **Hazra R, Gafni RI, Maldarelli F, Balis FM, Tullio AN, DeCarlo E, Worrell CJ, Steinberg SM, Flaherty J, Yale K, Kearney BP, Zeichner SL. Tenofovir disoproxil fumarate and an optimized background regimen of antiretroviral agents as salvage therapy for pediatric HIV infection. Pediatrics. 2005 Dec;116(6):e846-54. Epub 2005 Nov 15.**
- **Vigano A, Zuccotti GV, Martelli L, Giacomet V, Cafarelli L, Borgonovo S, Beretta S, Rombola G, Mora S. Renal Safety of Tenofovir in HIV-Infected Children: A Prospective 96-Week Longitudinal Study. Clin Drug Investig. 2007;27(8):573-81.**
- **Working Group on Antiretroviral Therapy and Medical Management of HIV-Infected Children. Guidelines for the Use of Antiretroviral Agents in Pediatric HIV Infection. October 26, 2006 1-126. Available at http://aidsinfo.nih.gov/ContentFiles/PediatricGuidelines.pdf.**

APPENDIX IX

19.0 TIPRANAVIR (TPV, APTIVUS®)

19.1 Current Preparations and Dosages Including FDA/EMEA Approved Dosing Guidelines

Antiretroviral Class: PI

Preparations:

- **Capsule: 250 mg**

Dosages:

- **Adult FDA/EMEA dose: 500 mg twice daily given with 200 mg ritonavir twice daily.**

19.2 Non FDA/EMEA Approved PENPACT 1 Dosing Recommendations

Pediatric/adolescent (>2 to ≤18 years) dose: 375 mg/m^2 tipranavir plus 150 mg/m^2 ritonavir. (Up to a maximum of 500 mg tipranavir twice daily plus 200 mg ritonavir twice daily.)

19.3 Major Toxicities:

Common: Diarrhea, nausea, fatigue, headache, rash, and vomiting. Laboratory abnormalities are elevated liver enzymes, cholesterol, and triglycerides. Less common (more severe): Fat redistribution. Clinical hepatitis and hepatic decompensation, including some fatalities. Patients with chronic hepatitis B or C coinfection or elevations in transaminases are at increased risk for developing further transaminase elevations or hepatic decompensation (approximately 2.5-fold risk). Rare: New onset diabetes mellitus, hyperglycemia, ketoacidosis, exacerbation of pre-existing diabetes mellitus, spontaneous bleeding in hemophiliacs. Possible association with increased risk of intracranial hemorrhage. Should not be used with supplemental Vitamin E.

References:

- **JC Salazar, P Cahn, M Della Negra, G Castelli-Gattinara, C Fortuny, P Flynn, R Yogev, PK Ruan, J Mikl, A Jelaska. Predictors of response to TPV/r in pediatric patients: Results of BI1182.14/PACTG 1051. 14th Conference on Retroviruses and Opportunistic Infections, February 2007. Poster #733.**

- **PACTG P1051 "A Multiple Dose, Open Label, Randomized, Safety and**

APPENDIX IX

Pharmacokinetic Study of Tipranavir in Combination with Low Dose Ritonavir in HIV-Infected Pediatric Patients, Version Date: BI Amendment #8 October 2, 2006 P6 / SIC Version 7 October 2, 2006, available at http://impaact.s-3.com/members/prothtm/P1051.htm (user name *impaact*, password *cure*).

20.0 ZIDOVUDINE (**ZDV,** RETROVIR®, **FDA-APPROVED GENERIC ZIDOVUDINE**)

20.1 Current Preparations and Dosages Including FDA/EMEA Approved Dosing Guidelines

Antiretroviral Class: NRTI

Preparations:

- Syrup: 10 mg/mL
- Capsule: 100 mg
- Tablet: 300 mg
- Concentrate for injection/for intravenous infusion: 10 mg/mL
- Tablets in combination with lamivudine: Combivir®: 300 mg zidovudine and 150 mg lamivudine.

Dosages:

- Dose for premature infants: (Standard neonatal dose may be excessive in premature infants). **1.5 mg/kg of body weight (intravenous) or 2 mg/kg of body weight (oral) every 12 hours, increased to every 8 hours at 2 weeks of age (neonates ≥30 weeks gestational age) or at 4 weeks of age (neonates <30 weeks gestational age).**
- FDA neonatal dose (infants aged <90 days) (accepted in Europe by recognition of FDA approval): Oral: 2 mg per kg of body weight every six hours. Intravenous: 1.5 mg per kg of body weight every six hours.
- FDA pediatric dose (accepted in Europe by recognition of FDA approval) 6 weeks to 12 years of age:
  - Oral: 160 mg per m^2 of body surface area every eight hours.
  - Intravenous (intermittent infusion): 120 mg per m^2 of body surface area to 180 mg per m^2 of body surface area every six to eight hours.

APPENDIX IX

- Intravenous (continuous infusion): 20 mg per m^2 of body surface area per hour.

- Pediatric dosage range routinely used in clinical setting: 360-480 mg per m^2 body surface area per day orally in two or three divided doses
- FDA adult dose (accepted in Europe by recognition of FDA approval): 200 mg three times a day or 300 mg twice daily.
- FDA/EMEA adolescent/adult dose of Combivir®: one tablet twice daily.

20.2 PENPACT 1 Dosing Recommendations

Alternate dosing options: **180 mg/m^2 to 240 mg/m^2 of body surface area every 12 hours.**

20.3 Major Toxicities:

Zidovudine may be associated with hematologic toxicity including granulocytopenia and severe anemia particularly in patients with advanced HIV disease. Prolonged use has been associated with symptomatic myopathy similar to that produced by human immunodeficiency virus. Rare occurrences of potentially fatal lactic acidosis in the absence of hypoxemia, and sever hepatomegaly with steatosis have been reported with the use of certain antiretroviral nucleoside analogues.

Reference:

Luzuriaga K, McManus M, Catalina M, Mayack S, Sharkey M, Stevenson M, Sullivan JL. Early therapy of vertical human immunodeficiency virus type 1 (HIV-1) infection: control of viral replication and absence of persistent HIV-1-specific immune responses. J Virol. 2000 Aug;74(15):6984-91.

APPENDIX X

MANAGEMENT OF SPECIFIC ADVERSE EVENTS FOR PACTG AND PENTA

Note:

Whenever a toxicity grade includes the use of the "ULN", as part of the calculation of the toxicity grade itself, sites must follow:

⇒ "ULN" values reported by the laboratory report for the test, or
⇒ "ULN" values routinely used/established by the site, or
⇒ "ULN" values (and/or calculations) as per the Harriet Lane Handbook.

1.0 Hypersensitivity Reaction to Abacavir (ABC)

1.1 Description of an ABC Hypersensitivity Reaction

In clinical studies, approximately 3-5% of patients receiving abacavir develop a hypersensitivity reaction which in rare cases has proved fatal. This is characterized by the appearance of symptoms indicating multi-organ/body system involvement. Symptoms usually appear within the first six weeks of initiation of treatment with abacavir (median time to onset is 11 days) and most often include fever, rash, gastrointestinal symptoms (nausea, vomiting, diarrhea, or abdominal pain), and lethargy or malaise. Other signs and symptoms may include respiratory symptoms (dyspnea, sore throat, cough), musculoskeletal symptoms (myalgia, rarely myolysis, arthralgia), headache, paresthesia and oedema. Some patients with hypersensitivity reactions were initially thought to have respiratory disease (pneumonia, bronchitis, pharyngitis) or a flu-like illness. This delay in diagnosis of hypersensitivity has resulted in abacavir being continued or re-introduced, leading to more severe hypersensitivity reactions or death. Therefore, the diagnosis of hypersensitivity reaction should be carefully considered for patients presenting with symptoms of these diseases. Renal failure and anaphylaxis have also been reported in association with hypersensitivity reactions.

Physical findings include lymphadenopathy and, occasionally, mucous membrane lesions (conjunctivitis and/or mouth ulceration) and hypotension. The rash is variable and may be absent, but often appears maculopapular or urticarial. Laboratory abnormalities that may accompany abacavir hypersensitivity include elevated liver function tests or creatine phosphokinase, creatinine or lymphopenia.

Symptoms related to this hypersensitivity reaction worsen with continued therapy and usually resolve upon discontinuation of abacavir. Restarting abacavir following a hypersensitivity reaction results in a prompt return of symptoms within hours.

This recurrence of the hypersensitivity reaction may be more severe than on initial

APPENDIX X (Cont.)

presentation and may include life-threatening hypotension and death. Subjects who

Develop a hypersensitivity reaction must discontinue abacavir and must not be re-challenged with abacavir.

Presumed hypersensitivity reactions (HSR) must be reported as serious adverse events and should also be discussed with the study co-chair (PACTG sites) or the appropriate trial center (PENTA sites). Additionally, an e-mail notification to actg.penpact1@fstrf.org for any presumed HSR must be sent from the study site. In the event that ABC is permanently discontinued, it should be replaced by zidovudine (ZDV)

1.2 Information for Patients about ABC hypersensitivity:

Subjects must be informed of the risk of a hypersensitivity reaction to abacavir and provided with information to help them recognized the symptoms and signs associated with possible hypersensitivity reactions. Patients must be advised to contact their doctor immediately if they experience such symptoms.

Warning Card - All subjects receiving abacavir should receive a wallet-size warning card provided by GlaxoSmithKline. As each subject is enrolled, the study site must assure that:

- The subject receives the warning card.
- The designated health care provider (e.g., physician, study nurse-coordinator, or pharmacist) reviews the signs and symptoms of hypersensitivity with the subject.
- The subject verbalizes an understanding of the steps to take in the event of a suspected hypersensitivity, including contacting the study site.

1.3 Medical Management of a Possible ABC Hypersensitivity Reaction:

If a subject reports symptoms suggestive of hypersensitivity, s/he should be instructed not to take any additional doses and should be evaluated at the clinic. The evaluation should consist of a careful history and physical examination. Laboratory studies should be obtained as clinically indicated. There is no diagnostic test available to confirm the clinical diagnosis. If upon evaluation the subject does have a presentation consistent with hypersensitivity, therapy with abacavir must be permanently discontinued. In addition, the event "abacavir hypersensitivity" is to be reported as a serious adverse event on the SAE form and sent to the SAE office. The hematology, chemistry, signs and symptoms, diagnoses, event evaluation and serious event evaluation forms in the CRF should also be completed as indicated.

Symptoms usually start to resolve soon (within 24 hours) after stopping therapy. Symptomatic support, such as intravenous fluids for those who develop hypotension, is advised. There are no clinical data demonstrating the benefit of antihistamines or

APPENDIX X (Cont.)

corticosteroids in the management of hypersensitivity. Nevertheless, symptomatic and/or supportive treatment may be reasonable.

Patients who have had a hypersensitivity reaction must be advised that they should never take abacavir (or Ziagen) again as a life-threatening second hypersensitivity reaction can occur. Patients should be advised to return all unused abacavir at the time of discontinuation for return to the sponsor according to study procedures to reduce the risk of inadvertent rechallenge.

1.4 ABC Rash and Hypersensitivity:

Subjects receiving abacavir who develop rash of any grade should be evaluated for the possibility of hypersensitivity as outlined above. If there is no indication of any other organ system involvement and the subject has no systemic symptoms (fever, malaise, fatigue, headache), abacavir may be continued with the warning to discontinue immediately and permanently if other signs and/or symptoms consistent with hypersensitivity appear.

1.5 Fever and ABC Hypersensitivity

The onset of fever may also herald hypersensitivity. Subjects reporting fever should be evaluated as above. In the event of a clinical presentation consistent with hypersensitivity, abacavir should be discontinued permanently, and the steps taken as stated above.

2.0 Skin Rash/Cutaneous Dermatitis (other than ABC associated)

Rashes which meet the criteria for Grade 2 or higher AE (using the Supplemental Toxicity Table for grading severity of cutaneous/skin rash/dermatitis in Appendix VI) must be reported immediately to the PENPACT1 Team by e-mail at actg.penpact1@fstrf.org

- Study drugs may be continued for Grade 2 (A or B) skin rash following consultation with the PENPACT1 team (Co-Chairs for PACTG sites trial center for PENTA sites).
- For children who develop cutaneous reactions $\geq$ Grade 2 with any of the following signs/symptoms, antiretroviral therapy should be immediately discontinued. Sites should contact the study co-chair (PACTG sites) or the appropriate trial center (PENTA sites) to receive instructions on course of action:
    - Systemic symptoms (such as fever, nausea, or vomiting) if clinically relevant
    - Allergic symptoms
    - Generalized rash or
    - Exfoliation
    - Target lesions
    - Mucosal involvement

APPENDIX X (Cont.)

- All study drugs must be permanently discontinued for any Grade 3 or 4 rash. Notify protocol team at actg.penpact1@fstrf.org.

3.0 Clinical Pancreatitis

If a subject develops nausea, vomiting , or abdominal pain of any grade associated with any elevation of serum fractionated pancreatic amylase or lipase, or develops a clinical syndrome that in the opinion of the subject's clinician is classified as pancreatitis, study medications should be permanently discontinued. Future consideration should be given to avoiding ddI or other drugs potentially affecting the pancreas. Notify protocol team at actg.penpact1@fstrf.org.

4.0 Hyperlipasemia

For elevations of lipase in blood, follow this algorithm:

- For Grade 2 hyperlipasemia (>1.5 ULN), consider holding all study drugs, notify protocol team at actg.penpact1@fstrf.org immediately, schedule followup visits every two weeks if necessary until toxicity resolves to ≤ Grade 1.
- For any Grade 3 hyperlipasemia (>2.5 ULN), hold all study drugs until both lipase and amylase are ≤ Grade 1. Notify protocol team at actg.penpact1@fstrf.org immediately.
- For Grade 4 hyperlipasemia (>5.0 ULN), all study drugs should be held and may be permanently discontinued, do not restart until both lipase and amylase are ≤ Grade 1. Notify protocol team at actg.penpact1@frstf.org immediately to determine course of action. If hyperlipasemia recurs, discontinue all study drugs.

5.0 Hyperamylasemia

Management of this toxicity will be prompted for a Grade 3 or 4 hyperamylasemia.

Upon presentation of Grade 3 or 4, the amylase should be fractionated and the pancreatic fraction should then be used to determine the toxicity management.

Additionally, a lipase should also be obtained and the following algorithm applies: (Remember that this is done as secondary assay to a Grade 3 or 4 hyperamylasemia. Hyperlipasemia alone must be managed as described on the previous section)

- If there is an elevation in lipase, hold all study drugs until both amylase and lipase are ≤ Grade 1. Notify protocol team at actg.penpact1@fstrf.org immediately.
- Pending the results of the fractionated amylase evaluation if the lipase is normal, study medications may be continued. Notify protocol team at actg.penpact@frstf.org.
- Once available, fractionated pancreatic amylase elevations should be managed as per PENPACT 1 protocol Section 6.3 depending on the toxicity grade.

APPENDIX X (Cont.)

6.0 Increase in Values for the Liver Function Tests (LFT's):

The following algorithm for management of this toxicity should be observed:

- For all Grade 2 LFT's, monitor subject every two weeks until values return to Grade 1. Notify protocol team at actg.penpact1@fstrf.org.
- In general all Grade 2 LFT's or higher, should be reported to the team every other week.
- Elevations in LFT's should be managed as per Section 6.3 of the protocol depending on the toxicity grade.

7.0 CNS Symptoms

For grading CNS symptoms follow Appendix V "Division of AIDS Toxicity Table for Grading Severity of Pediatric (>3 months of age) Adverse Experiences."

For management of CNS toxicities follow Section 6.3. Contact the protocol team via e-mail at actg.penpact1@fstrf.org, if you have any doubts on how to proceed after observing specific CNS symptoms.

8.0 Hyperglycemia/glycosuria

If non-fasting blood glucose > 200 or urinary dipstick > 2+ positive, obtain fasting blood glucose. If the fasting blood glucose is ≥ 150 mg/dl or greater, notify protocol team at actg.penpact1@fstrf.org immediately. Consult with an endocrinologist regarding possible new onset diabetes, and relay this information, and any followup information, to the protocol team.

9.0 Elevated Cholesterol or Triglycerides

Initiation of HAART with or without PIs and /or NNRTIs have been associated with elevations in cholesterol and triglyceride levels. These elevations should be managed as follows:

- For cholesterol levels > 500 mg/dL, repeat a fasting cholesterol level.
- For triglycerides > 750 mg/dL, repeat a fasting triglyceride level.

For grading of cholesterol and triglyceride toxicities, use the following guidelines:

- Cholesterol:

APPENDIX X (Cont.)

Grade 0 = 0-170 mg/dL, Grade 1 = 171-499 mg/dL, Grade 2 = 500-749 mg/dL, Grade 3 = > 750 mg/dL. If Grade 2 or 3 toxicity is recorded, sites should contact the study co-chair (PACTG sites) or the appropriate Trials Center (PENTA sites) to determine if treatment should be modified or discontinued.

- Triglycerides:

  Grade 0 = 0-135 mg/dL, Grade 1 = 136-749 mg/dL, Grade 2 = 750-1199 mg/dL, Grade 3 > 1200 mg/dL. If Grade 2 or 3 toxicity is recorded, sites should contact the study co-chair (PACTG sites) or the appropriate Trials Center (PENTA sites) to determine if treatment should be modified or discontinued.

10.0 Hematologic Toxicities

For hematologic toxicities, erythropoietin and/or G-CSF/GM-CSF may be administered as clinically indicated before considering dose reduction.

Only ABC, and d4T should be dose reduced. Only two (2) dose modification cycles are allowed. Following the second cycle, the full dose of the study drug can no longer be resumed.

For other hematologic toxicities where no dose reduction is allowed, follow Section 6.3 of the protocol.

11.0 Neutropenia and Anemia:

These two toxicities are managed as illustrated in Figure 1.

APPENDIX X (Cont.)

FIGURE 1: Management of Neutropenia and Anemia

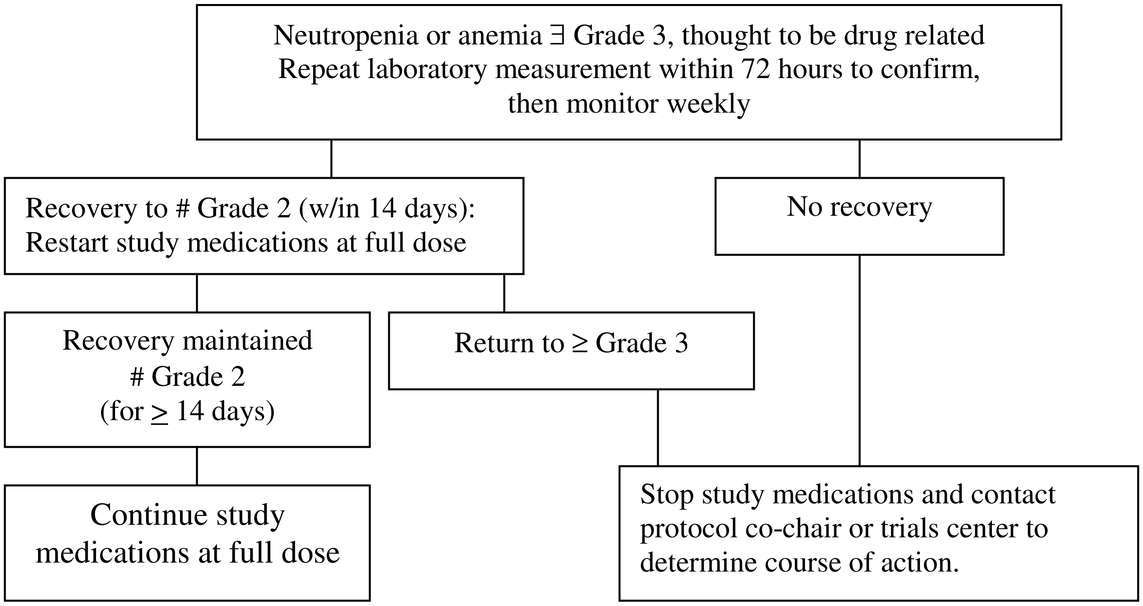

G-CSF/GM-CSF and erythropoietin are allowed and should be used where possible before any drug reduction.

12.0 Lactic Acidosis

Development of lactic acidosis is considered a toxicity endpoint. Lactic acidosis and a liver dysfunction syndrome have been associated with NRTI's mono-and combination therapies. In PENPACT 1 the possibility of the development of these conditions will be monitored. For subjects with ALT and AST values above 2.5 x ULN with no easily discernable etiology (e.g. acute hepatitis A, B, C, or chronic hepatitis B or C), a serum bicarbonate or lactate will be obtained. If persistent abnormal values are obtained by verified correct blood drawing techniques for the specimen, hold all study drugs and contact the protocol Co-chair (PACTG sites) or the appropriate Trials Center (PENTA sites). Also, send an e-mail to team log-on at actg.penpact1@fstrf.org notifying the protocol team. The protocol team will work with the subject's clinician to determine the best course of action for subjects in whom NRTI associated liver dysfunction syndrome and lactic acidosis are confirmed.

APPENDIX XI

MEDICATIONS WITH KNOWN SERIOUS INTERACTIONS

(INFORMATION TO BE CONSIDERED BY PACTG AND PENTA)

Protease inhibitors (PI) are known inhibitors of the cytochrome P450 system, following is a list of medications known to have serious interactions, and in some cases are thus relatively or absolutely contra-indicated for use with a PI.

▪ alprazolam	XANAX®
▪ amiodarone	CORDARONE®
▪ astemizole	HISMANAL®
▪ bepridil	VASCOR®
▪ bupropion	WELLBUTRIN®,ZYBAN®
▪ carbamazepine	TEGRETOL®
▪ cisapride	PROPULSID®
▪ clorazepate	TRANXENE®
▪ clozapine	CLOZARIL®
▪ diazepam	VALIUM®
▪ dexamethasone	DECADRON®
▪ encainide	ENKAID®
▪ estazolam	PROSOM®
▪ ergot alkaloids and derivatives:	
	BELLERGAL®
	CAFERGOT®
	ERGOSTAT®
	WIGRAINE®, and others
▪ flecainide acetate	TAMBOCOR®
▪ flurazepam	DALMANE®
▪ ketoconazole	NIZORAL®
▪ isotretinoin	ACCUTANE®
▪ itraconazole	SPORANOX®
▪ meperidine	DEMEROL®
▪ midazolam	VERSED®
▪ Phenobarbital	________________________
▪ phenytoin	DILANTIN®
▪ pimozide	ORAP®
▪ piroxicam	FELDENE®
▪ propafenone	RYTHMOL®
▪ propoxyphene	DARVON®, DARVOCET®
▪ quinidine	QUINAGLUTE®
▪ rifabutin	MYCOBUTIN®
▪ rifampin	RIFADIN®, RIMACTANE®
▪ terfenadine	SELDANE®
▪ triazolam	HALCION®
▪ zolpidem	AMBIEN®

APPENDIX XII

CDC CLASSIFICATION
PEDIATRIC HIV CLASSIFICATION
CDC 1994 REVISED CLASSIFICATION SYSTEM FOR HIV-INFECTION IN CHILDREN LESS THAN 13 YEARS OF AGE (MMWR 1994; 43 [RR-12]:1-10)

(INFORMATION TO BE USED BY PACTG AND PENTA FOR ALL CHILDREN IN THE STUDY REGARDLESS OF THEIR AGE)

TABLE 1. Pediatric human immunodeficiency virus (HIV) classification*

Clinical Categories				
Immunologic Categories	N: No signs/ symptoms	A: Mild signs/ symptoms	B:** Moderate signs/symptoms	C:** Severe signs/symptoms
1: No evidence of suppression	N1	A1	B1	C1
2: Evidence of moderate suppression	N2	A2	B2	C2
3: Severe suppression	N3	A3	B3	C3

* Children whose HIV infection status is not confirmed are classified by using the above grid with a letter E (for perinatally exposed) placed before the appropriate classification code (e.g., EN2).

** Both Category C and lymphoid interstitial pneumonitis in Category B are reportable to state and local health departments as acquired immunodeficiency syndrome.

TABLE 2. Immunologic categories based on age-specific CD4+ T-lymphocyte counts and percent of total lymphocytes

Immunologic Category	Age of Child		
	< 12 months	1-5 years	6-12 years
	l (%)	l (%)	l (%)
1: No evidence of suppression	≥ 1,500 (≥ 25)	≥ 1,000 (≥ 25)	≥ 500 (≥ 25)
2: Evidence of moderate suppression	750-1,499 (15-24)	500-999 (15-24)	200-499 (15-24)
3: Severe suppression	< 750 (< 15)	< 500 (< 15)	< 200 (< 15)

APPENDIX XII (Cont.)

Box 2. Clinical Categories for Children with Human Immunodeficiency Virus (HIV) Infection

CATEGORY N: NOT SYMPTOMATIC

Children who have no signs or symptoms considered to be the result of HIV infection or who have only one of the conditions listed in Category A.

CATEGORY A: MILDLY SYMPTOMATIC

Children with two or more of the conditions listed below but none of the conditions listed in Categories B and C.

- Lymphadenopathy (≥ 0.5 cm at more than two sites; bilateral = one site)
- Hepatomegaly
- Splenomegaly
- Dermatitis
- Parotitis
- Recurrent or persistent upper respiratory infection, sinusitis, or otitis media

CATEGORY B: MODERATELY SYMPTOMATIC

Children who have symptomatic conditions other than those listed for Category A or C that are attributed to HIV infection. Examples of conditions in clinical Category B include but are not limited to:

- Anemia (< 8 gm/dL), neutropenia (< 1,000/mm3), or thrombocytopenia (<Ê100,000/mm3) persisting ≥ 30 days
- Bacterial meningitis, pneumonia, or sepsis (single episode)
- Candidiasis, oropharyngeal (thrush), persisting (> 2 months) in children > 6 months of age
- Cardiomyopathy
- Cytomegalovirus infection, with onset before 1 month of age
- Diarrhea, recurrent or chronic
- Hepatitis
- Herpes simplex virus (HSV) stomatitis, recurrent (more than two episodes within 1 year)
- HSV bronchitis, pneumonitis, or esophagitis with onset before 1 month of age
- Herpes zoster (shingles) involving at least two distinct episodes or more than one dermatome

APPENDIX XII (Cont.)

- Leiomyosarcoma
- Lymphoid interstitial pneumonia (LIP) or pulmonary lymphoid hyperplasia complex
- Nephropathy
- Nocardiosis
- Persistent fever (lasting > 1 month)
- Toxoplasmosis, onset before 1 month of age
- Varicella, disseminated (complicated chickenpox)

CATEGORY C: SEVERELY SYMPTOMATIC

Children who have any condition listed in the 1987 surveillance case definition for acquired immunodeficiency syndrome (10), with the exception of LIP (Box 3).

Box 3. Conditions included in clinical Category C for children infected with human immunodeficiency virus (HIV)

CATEGORY C: SEVERELY SYMPTOMATIC*

Serious bacterial infections, multiple or recurrent (i.e., any combination of at least two culture-confirmed infections within a 2-year period), of the following types: septicemia, pneumonia, meningitis, bone or joint infection, or abscess of an internal organ or body cavity (excluding otitis media, superficial skin or mucosal abscesses, and indwelling catheter-related infections)

- Candidiasis, esophageal or pulmonary (bronch, trachea, lungs)
- Coccidioidomycosis, disseminated (at site other than or in addition to lungs or cervical or hilar lymph nodes)
- Cryptococcosis, extrapulmonary
- Cryptosporidiosis or isosporiasis with diarrhea persisting > 1 month
- Cytomegalovirus disease with onset of symptoms at age > 1 month (at a site other than liver, spleen, or lymph nodes)
- Encephalopathy (at least one of the following progressive findings present for at least 2 months in the absence of a concurrent illness other than HIV infection that could explain the findings):
  - a) failure to attain or loss of developmental milestones or loss of intellectual ability, verified by standard developmental scale or neuropsychological tests;
  - b) impaired brain growth or acquired microcephaly demonstrated by head

APPENDIX XII (Cont.)

circumference measurements or brain atrophy demonstrated by computerized tomography or magnetic resonance imaging (serial imaging is required for children < 2 years of age);

c) acquired symmetric motor deficit manifested by two or more of the following: paresis, pathologic reflexes, ataxia, or gait disturbance

- Herpes simplex virus infection causing a mucocutaneous ulcer that persists for > 1 month; or bronchitis, pneumonitis, or esophagitis for any duration affecting a child > 1 month of age
- Histoplasmosis, disseminated (at a site other than or in addition to lungs or cervical or hilar lymph nodes)
- Kaposi's sarcoma
- Lymphoma, primary, in brain
- Lymphoma, small, noncleaved cell (Burkitt's), or immunoblastic or large cell lymphoma of B-cell or unknown immunologic phenotype
- Mycobacterium tuberculosis, disseminated or extrapulmonary
- Mycobacterium, other species or unidentified species, disseminated (at a site other than or in addition to lungs, skin, or cervical or hilar lymph nodes)
- Mycobacterium avium complex or Mycobacterium kansasii, disseminated (at site other than or in addition to lungs, skin, or cervical or hilar lymph nodes)
- Pneumocystis carinii pneumonia
- Progressive multifocal leukoencephalopathy
- Salmonella (nontyphoid) septicemia, recurrent
- Toxoplasmosis of the brain with onset at > 1 month of age
- Wasting syndrome in the absence of a concurrent illness other than HIV infection that could explain the following findings:

  a) persistent weight loss > 10% of baseline

  OR

  b) downward crossing of at least two of the following percentile lines on the weight-for-age chart (e.g., 95th, 75th, 50th, 25th, 5th) in a child ≥ 1 year of age

  OR

  c) < 5th percentile on weight-for-height chart on two consecutive measurements, ≥ 30 days apart <u>PLUS</u> a) chronic diarrhea (i.e., at least two loose stools per day for ≥ 30 days) OR b) documented fever (for ≥ 30 days, intermittent or constant)

<u>Immunologic Categories</u>

The three immunologic categories (Table 2) were established to categorize children by the severity of immunosuppression attributable to HIV infection. CD4+ T-lymphocyte depletion is a major consequence of HIV infection and is responsible for many of the severe manifestations of HIV infection in adults. For this reason, CD4+ counts are used in the adult HIV classification system (11). However, several findings complicate the use of CD4+ counts for assessing immunosuppression resulting from HIV infection in children. Normal CD4+ counts are higher

in infants and young children than in adults and decline over the first few years of life (12-16).

APPENDIX XII (Cont.)

In addition, children may develop opportunistic infections at higher CD4+ levels than adults (17-19). Although insufficient data exist to correlate CD4+ levels with disease progression at all age groups, low age-specific CD4+ counts appear to correlate with conditions associated with immunosuppression in children (12,17,20,21). Therefore, despite these complications, classification based on age-specific CD4+ levels appears to be useful for describing the immunologic status of HIV-infected children.

Fewer data are available on age-specific values for CD4+ T-lymphocyte percent of total lymphocytes than for absolute counts. However, the CD4+ T-lymphocyte percent has less measurement variability than the absolute count (22). To establish the age-specific values of CD4+ percent that correlate with the CD4+ count thresholds, CDC compiled data from selected clinical projects in the United States and Europe. The data included > 9,000 CD4+ counts, with the corresponding CD4+ percent determinations, from both HIV-infected and uninfected children < 13 years of age. Nonparametric regression modeling was used to establish the CD4+ percent boundaries that best correlated with the CD4+ count boundaries in the classification system.

The immunologic category classification (Table 2) is based on either the CD4+ T-lymphocyte count or total lymphocytes. If both the CD4+ count and the CD4+ percent indicate different classification categories, the child should be classified into the more severe category. Repeated or followup CD4+ values that result in a change in classification should be confirmed by a second determination. Values thought to be in error should not be used. A child should not be reclassified to a less severe category regardless of subsequent CD4+ determinations.

Clinical Categories

Children infected with HIV or perinatally exposed to HIV may be classified into one of four mutually exclusive clinical categories based on signs, symptoms, or diagnoses related to HIV infection (Box 2). As with the immunologic categories, the clinical categories have been defined to provide a staging classification (e.g., the prognosis for children in the second category would be less favorable than for those in the first category).

Category N, not symptomatic, includes children with no signs or symptoms considered to be the result of HIV infection or with only one of the conditions listed in Category A, mildly symptomatic. Category N was separated from Category A partly because of the substantial amount of time that can elapse before a child manifests the signs or symptoms defined in Category B, moderately symptomatic. Also, more staging information can be obtained during this early stage of disease by separating Categories N and A. In addition, for children who have uncertain HIV-infection status (prefix E), Categories N and A may help to distinguish those children who are more likely to be infected with HIV (23) (i.e., children in Category EA may be more likely to be infected than children in Category EN).
Category B includes all children with signs and symptoms thought to be caused by HIV infection but not specifically outlined under Category A or Category C, severely symptomatic. The conditions listed in Box 2 are examples only; any other HIV-related condition not included in

APPENDIX XII (Cont.)

Category A or C should be included in Category B. Anemia, thrombocytopenia, and lymphopenia have defined thresholds in the new classification system (23).

Category C includes all AIDS-defining conditions except lymphoid interstitial pneumonitis (LIP) (Box 3). Several reports indicate that the prognosis for children with LIP is substantially better than that for children who have other AIDS-defining conditions (21,24,25). Thus, LIP has been separated from the other AIDS-defining conditions in Category C and placed in Category B.

Signs and symptoms related to causes other than HIV infection (e.g., inflammatory or drug-related causes) should not be used to classify children. For example, a child with drug-related hepatitis or anemia should not be classified in Category B solely because these conditions may be associated with HIV infection. In contrast, a child with anemia or hepatitis should be classified in Category B when the condition related to HIV infection. The criteria for diagnosing some conditions and determining whether a child's signs, symptoms, or diagnoses are related to HIV infection may not be clear in all cases, and therefore may require judgment of the clinicians and researchers using the classification system.

Categories in the 1987 pediatric HIV classification system can be translated into categories in the 1994 system in most cases (Box 4). Class PO is now designated by the prefix "E", and Class P1 is now Class N. Children previously classified as P2A are now classified in more than one category, reflecting the different prognoses for children with different conditions included in the P2A category (e.g., children who have wasting syndrome have a worse prognosis than those who have lymphadenopathy).

APPENDIX XIII

NEURODEVELOPMENTAL ASSESSMENT
(ONLY FOR U.S. PACTG DOMESTIC SITES)

Age Group at Entry	Tests
Birth to 30 months	Bayley Scales II (BSID 1993)<br>Including MDI, PDI and BRS
30 months to 75 months	WPPSI-III (2002)<br>BASC-Parent/Caregiver
75 months to 15 years and 11 months	WISC-III (1991)<br>BASC-Parent/Caregiver
16.0 years and above	WAIS-III (1997)<br>BASC-Parent/Caregiver (Through 18.11)<br>Beck Depression Inventory-II (BDI-II, 1996)<br>State-Trait Anxiety Inventory (STAI)

RULES:

- Every child will be assessed with exactly the same instruments at baseline and follow-ups.
- The child age determines the kind of test for evaluation at baseline and study follow-ups until Week 48. Additionally, any child enrolled in the study under Version 1.0 who was assessed with the WPPSI-R, CPRS-48, or CES-D test should continue being evaluated with same test for the rest of the study, as long as it is age-appropriate. All other tests should be changed to new evaluations in the table above.
- Example: a 2 year 4 month old child is evaluated with the Bayley at baseline, Weeks 24 and at 48. Subsequent evaluations after Week 48 should be done with the WPPSI-III.

Considerations for the choice of the battery:

- Circumvent change of test instruments between baseline and up to 1 year follow-ups
- Use tests that have similar structure in the various age groups
- Maximize similarity with other recent or on-going studies
- Use tests that are familiar to most and do not require significant new purchases
- Limit assessment time and number of evaluations
- If possible choose tests that also have Spanish (or other language) forms available

Evaluation time points:

- Baseline
- Week 24 (only for those children less than 6 years of age at the time of enrollment),
- Week 48.
- After the first 48 weeks, below age 6 years at the time of testing every 48 weeks, above 6 years every 96 weeks.

APPENDIX XIV

MEASUREMENT OF HEIGHT, LENGTH, WEIGHT AND HEAD CIRCUMFERENCE
(PROCEDURES TO BE FOLLOWED BY PACTG AND PENTA)

1. Height

- Once a subject is greater than 24 months of age and can stand upright, stature should be measured using a calibrated, wall-mounted stadiometer.

- For best results, the subject is measured wearing a gown that allows the measurer to visualize the subject's body position. The subject stands with bare feet close together, body and legs straight, arms at sides, relaxed shoulders, and head, back, buttocks, and heels against the wall or shaft of the stadiomenter.

- Instruct the subject to look straight ahead and stand tall, keeping heels on the ground.

- Bring the headboard down to the top of the subject's head while at eye-to-eye level with the subject and record the height to the nearest 0.1 cm.

2. Length

- Measure subject's recumbent length up to 24 months of age and, for those unable to stand, up to 36 months of age on a calibrated length board with a stable headboard and a sliding footboard.

- Two people are required to perform an accurate length measurement. One person holds the subject's head in place while the other slides the footboard and takes the reading. The subject's foot should be flat against the footboard with toes pointing straight upward and legs straight at the time of measurement.

Subjects who are longer than standard length boards but cannot stand should be measured on a hospital bed as accurately as possible. Two measurers must be involved. One holds the subject's head in place while the other marks the bed paper where the top of the head meets the bed. The feet need to be flexed with toes pointing upward. Legs and torso need to be straight during the time of marking the bed at the heel. Using a ruler, draw a straight line from the marks to the edge of the bed paper. Measure between the lines without the subject's moving.

3. Weight

- Use an electronic or beam scale with non-detachable weights.

- Zero the scale prior to each measure. Use a calibrated scale.
- Infants and young toddlers should be weighed lying down wearing only a diaper during

APPENDIX XIV (Cont.)

the measurement.

- Weigh children who can stand on a beam scale, preferably one with “handle bars” for support. Calm children down and reduce their movement as much as possible for accurate measurements. Take a child's weight while he or she is **wearing minimal clothing and no shoes.**

- For children who have disabilities that prevent them from standing on a beam scale but are large for an infant scale, use a bed scale. Alternatively, an adult may hold the child and stand on the scale with and without the child. The weight of the adult is subtracted from the combined adult-child weight to determine the child’s weight. This method is only to be used for children who cannot stand alone and are too large for the infant scale.

4. Head Circumference

The tape is placed across the forehead with the lower border of the tape just above the eyebrows, around the head, above the ears and over the occipital prominence at the back of the head. Pull tape firmly to compress the hair and underlying soft tissues. Positioning of the tape over the forehead and occiput should be done to yield the maximum head circumference. Record head circumference in centimeters to the nearest 0.1 cm.

APPENDIX XV

<u>TANNER SCALES</u>

(PROCEDURES TO BE FOLLOWED BY PACTG AND PENTA)

The Five Stages of Female Breast Development

Stage 1

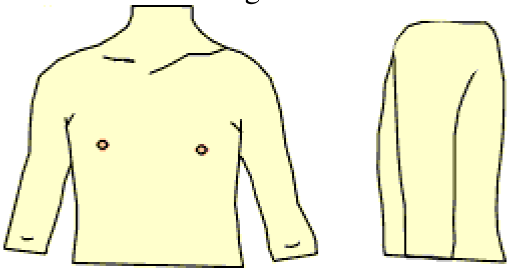

Breasts during childhood. The breasts are flat and show no signs of development.

2

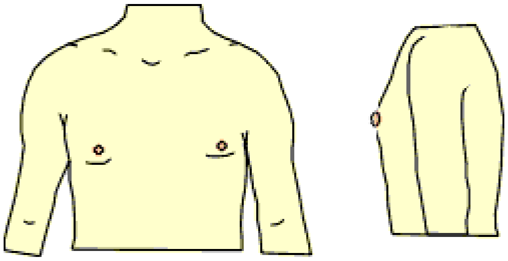

Breast bud stage. Milk ducts and fat tissue forms a small mound.

3

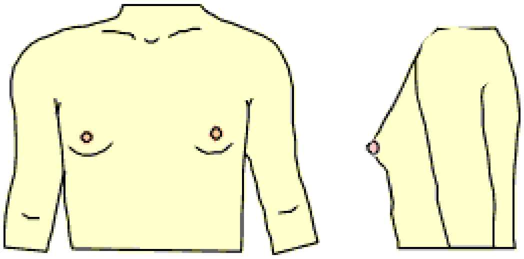

Breasts continue to grow. Breasts become rounder and fuller.

4

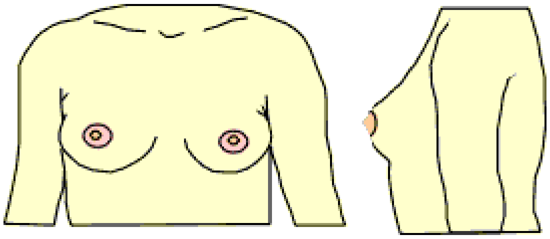

Nipple and areola form separate small mound. Not all girls go through this stage. Some skip stage 4 and go directly to stage 5.

5

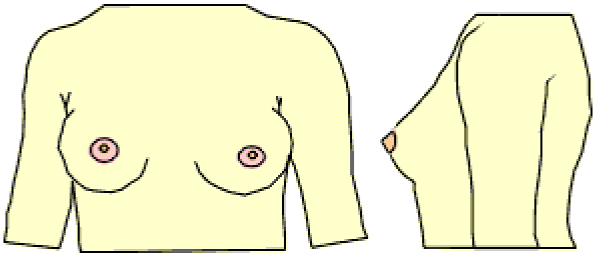

Breast growth enters final stage. Adult breast is full and round shaped.

Copyright© 1998,1999 VirtualKid All Rights Reserved.

APPENDIX XV (Cont.)

The Five Stages of Female Pubic Hair Development

Stage 1

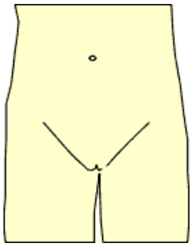

No pubic hair.

2

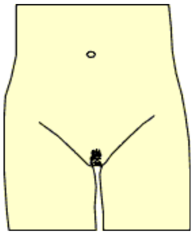

Pubic hair is sparse, lightly pigmented, straight, medial border of labia.

3

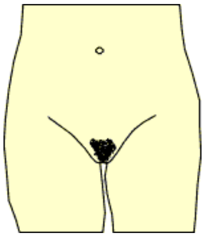

Pubic hair is darker, beginning to curl, and increases in amount.

4

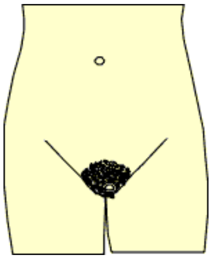

Pubic hair is coarse, curly, abundant but the amount is less that in an adult woman.

5

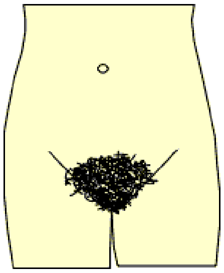

Pubic hair is that of an adult woman forming a triangle spread to medial surface of thighs.

APPENDIX XV (Cont.)

The Five Stages of Male Pubic Hair Development

Stage
1

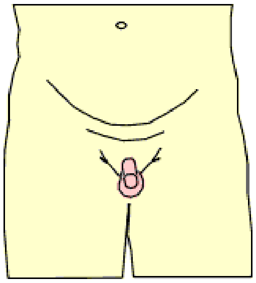

No pubic hair. Penis and testicles of a child. Testicles between 1 and 3 milliliters in volume.

2

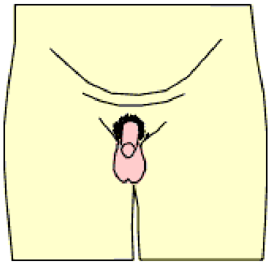

First signs for penis, testicle growth, and pubic hair beginning to grow. Pubic hair appears sparse and downy straight. Testicles become larger. Testicles between 4 and 6 milliliters in volume.

3

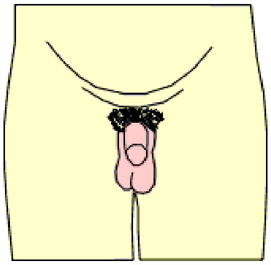

Pubic hair appears curlier and coarser with increased pigmentation. Penis continues to grow getting wider and longer. Testicles continue to grow larger. Testicles between 7 and 16 milliliters in volume.

4

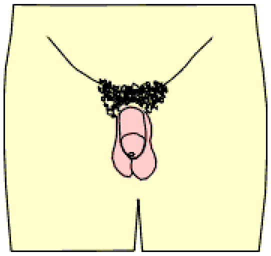

Penis continues to grow getting wider and longer. Pubic hair becomes adult type, but less. Testicles continue to grow larger. Penis gland or head is more developed. Testicles between 12 and 24 milliliters in volume. Testicles are about 1 1/2 inches long.

5

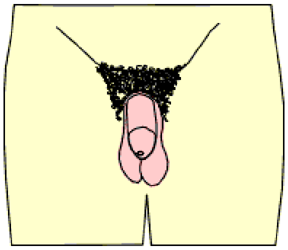

Penis growth enters final stage. Average erect penis length 6 1/4 inches. 90% are 5 - 7 inches. Pubic hair is thick spreading to medial thighs. Glans penis or head is fully developed. Testicles 16 - 27 milliliters in volume. Testicles are about 1-3/4 inches.

APPENDIX XVI

SAMPLE INFORMED CONSENT FOR PENTA SITES

THE PENPACT 1 TRIAL
An Information Sheet For Parents

Your child is being invited to take part in a research study. Before you decide it is important for you to understand why the research is being done and what it will involve. Please take time to read the following information carefully and discuss it with others if you wish. Ask us if there is anything that is not clear or if you would like more information. Take time to decide whether or not you wish your child to take part.

WHAT IS PENPACT 1 ?

PENPACT 1 is a study organised by the Paediatric European Network for Treatment of AIDS (PENTA) in Europe and a similar group in the USA. It is a study for children with HIV infection who need to go on treatment for the first time. Significant progress has been made in the understanding, and treatment of HIV infection and AIDS. Although there are no drugs presently available that can eliminate HIV completely, there are drugs which slow the growth of HIV in the body. These are called anti-HIV drugs.

There are already at least 15 such drugs available for children, and new drugs are being developed. It is known that taking a combination of three drugs together works more effectively than using only one or two drugs. However it is not known which combination of drugs gives the most benefit over time with the least side effects in children.

After starting anti-HIV drugs it is expected that most children will have very low (undetectable) levels of HIV in the blood. However, after some time, the level of virus often starts to rise again, especially if the drugs are not taken very regularly. This may mean that the virus becomes resistant to the drugs.
At the moment we still don't know when the best time is to switch therapy, if the drugs are changed too early that perhaps will mean that we quickly run out of suitable drugs for children. On the other hand, staying on the same drug combination may be more likely to increase resistance of HIV to the drugs. However, there is evidence the virus growing while on treatment with these drug combinations will not produce HIV-related disease as rapidly as when the virus grows in a person without drug treatment.

WHAT ARE THE AIMS OF THE TRIAL ?

The PENPACT 1 plans to include 256 HIV infected children from clinics in Europe and the USA who need to start treatment, (a decision made by your child’s doctor).

APPENDIX XVI (Cont.)

The study has 2 main aims:

1. To compare different combinations of anti-HIV drugs to see which is the best to start with to slow the growth of the virus in the body and which has the least side-effects for children. The drugs are the same ones that your doctor would use even if you decide that you do not want your child to participate.
2. To decide what to do if the HIV level rises again in the blood. We will look at whether it is better to change to a new set of anti-HIV drugs as soon as the virus becomes detectable or if it is better to wait until the amount of virus in the blood is a bit higher.

Children in the trial will be chosen at random by a computer to change their drugs when their viral load (level of the virus) goes over either 1 000 copies/ml or over 30 000 copies/ml.
This will mean that some children may stay on the same therapy for a period of time with detectable viral load levels below 30 000 copies/ml. (In children on no treatment, the viral load is often greater than 100,000 copies/ml).

<u>WHAT DRUGS WILL BE USED ?</u>

It is important that your child takes anti-HIV medicine as prescribed by the doctor, whether they are in the trial or not.

Children will start treatment with 3 drugs:

- two NRTIs and one NNRTI

OR

- two NRTIs and one Protease Inhibitor

NRTIs - Nucleoside Reverse Transcriptase Inhibitors:- Your doctor will select 2 of the following drugs:

- 3TC (also called Lamivudine or Epivir).
- Abacavir (also called Ziagen).
- ddI (also called didanosine or Videx). This should be taken on an empty stomach.
- d4T (also called stavudine or Zerit).
- AZT (also called Zidovudine, Retrovir or ZDV)

These drugs are all available as syrups or as tablets and should be taken twice a day, except for ddI which can be prescribed once or twice a day. (3TC can also be prescribed once a day in adolescents).

NNRTI - Non-Nucleoside Reverse Transcriptase Inhibitors or PI - Protease Inhibitors
A computer will choose at random whether your child receives an NNRTI or a PI.

If your child is allocated to receive an NNRTI, your doctor will prescribe either :

- Nevirapine (also known as Viramune) OR

APPENDIX XVI (Cont.)

- Efavirenz (also known as Sustiva).

If your child is allocated to receive a PI, your doctor will select ONE of the following drugs:

- Nelfinavir (Viracept)
- Lopinavir (Kaletra)
- Ritonavir (Norvir)
- Saquinavir (Fortovase or Invirase)
- Amprenavir (Agenerase)
- Indinavir (Crixivan).

Sometimes a small dose of Ritonavir is given as well to boost one of the other drugs in this group. The decision about which drug will be taken by your doctor.

When the time comes to change drugs, those children who started with a Protease inhibitor should change to an NNRTI and those children who started with an NNRTI drug should change to a Protease Inhibitor. The NRTIs will also be changed. However, if by the time your child needs to change drugs there are newer better drugs available, your doctor may discuss using different drugs.

HOW OFTEN WILL WE NEED TO ATTEND THE CLINIC?

Your child will be seen at the following times for blood tests and an examination:
At the beginning of the trial to ensure that it is appropriate for your child to participate.

- 2 weeks later when the drugs will be started
- 2 weeks after starting drugs
- Every 4 weeks for the next 12 weeks
- Then, once every 12 weeks until all children in the trial have been followed for 4 years.

We hope that you will agree for your doctor to continue to send information about your child, such as routine blood results and general medical progress for long term follow up after the trial period has ended. This information will continue to be confidential and will be used under the study number only.

The level of virus in the blood will be measured at each clinic visit. If it goes over the level allocated to your child (1,000 or 30,000 copies/ml) twice in a row, your doctor will prescribe new drugs for your child. At some clinic visits small quantities of blood may be stored to look at particular ways in which the virus behaves.

WHAT IF MY CHILD BECOMES ILL DURING THE TRIAL?

APPENDIX XVI (Cont.)

If your child becomes ill at any time during the study, and your doctor feels that he or she would benefit from changing drugs, this will be discussed with you.

CONFIDENTIALITY

All information collected about your child during PENPACT 1 will be confidential. Names will not be used on any data collection forms. Your child will be identified by a study number only.

An independent group called the Data and Safety Monitoring Committee will meet regularly to oversee the progress of the trial. They will recommend whether the trial should continue as planned or should stop. Information from these meetings will be summarized in the PENTA Newsletter for families involved in PENTA studies.

PARTICIPATION

Participation in this study is entirely voluntary. If you decide that you do not wish your child to take part that is entirely your right. Your decision will in no way affect any present or future treatment for your child.

You can withdraw your child from the trial at any time without giving a reason. This will not affect your child's medical care. We hope, however, that parents will give a reason why they wish to withdraw their child and will continue to allow follow-up.

WHAT ELSE DO I NEED TO KNOW?

The Ethics Committee of your hospital has given approval for this trial to be conducted at your clinic.
PENTA, which is funded by the European Union, has made agreements for compensation should your child come to any harm during the trial. Your doctor will be able to tell you about this.

SIDE-EFFECTS OF THE DRUGS PRESCRIBED

Your child will be randomised to receive either a PI or an NNRTI. Your doctor will provide you with further information on the individual drugs.
However some general points can be made:

In general, the main side-effects of all Protease Inhibitor drugs are long-term and include concerns about the way the fats in the body are managed (high blood fat levels) and sometimes changes in fat distribution causing a thinning of the face, legs and arms and an increase in fat around the stomach (lipodystrophy). These body fat changes have also been observed with drugs from the NRTI group (especially d4T). The cause of these changes is not clear yet and it seems that not only drugs but other problems like a low CD4 count at start of therapy are also involved in the development of these body fat changes.

APPENDIX XVI (Cont.)

The most common side-effect of the NNRTI drugs is rashes. Adverse effects to Efavirenz in the first days of therapy are often dizziness, insomnia, somnolence, abnormal dreams and hallucinations, and depression (nervous system symptoms). However these generally resolve after 2-4 weeks, although about 7% of patients may continue to have symptoms.

<u>In addition to the PI or NNRTI drug your child will also receive 2 NRTIs.</u> Occasional problems with the liver (hepatitis) or with the body's metabolism (lactic acidosis) or lipodystrophy have been reported with this type of drugs (NRTIs)

The more significant side-effects of specific NRTIs are as follows:
The main side-effect of Abacavir is flu-like symptoms, with or without a rash, up to 4 weeks after your child has started taking this drug. Your doctor or clinic nurse will give you more information about this. This side-effect is not very common, however if your child should develop these symptoms, you should inform your doctor immediately. If your doctor tells you to stop the Abacavir, your child should never start it again.

The most important side-effect of d4T is neuropathy (problems with the nervous system). This has been seen in adults but is rare in children. A sign of neuropathy can be pain or tingling of the feet to touch and your doctor will also check your child's reflexes at each visit.

A rare side-effect of ddI can be an inflammation of the pancreas (pancreatitis) which can be associated with vomiting, diarrhoea and abdominal tenderness and can be checked for with a blood test at clinic visits. It stops when the drug is stopped. ddI can also cause neuropathy.

OTHER POSSIBLE RISKS
It is possible that some combinations of antiretroviral drugs may harm an unborn child. If girls could become pregnant, they must have a pregnancy test before entering the trial and must use effective contraceptives including condoms or other barrier contraception if they are having sex. Any boys having sex must also use condoms.

Thank you for taking time to consider this trial for your child. Please ask any questions and let us know if there are things that you do not understand, or would like more information about.

APPENDIX XVI (Cont.)

PENPACT 1 STUDY CONSENT FORM

I have read and understood the information sheet on the PenPact 1 trial. I understand the benefits and disadvantages of my child participating in this study. The details of this study have been explained by Dr……………………….. who has answered my questions satisfactorily.
YES NO

I agree that my child should take part in this trial which is comparing different drug combinations when starting HIV treatment and also comparing different viral load levels (levels of virus in the blood) as an indicator for changing drugs. YES NO

I understand that my child may be randomised to stay on the same antiretroviral therapy even when the viral load is detectable (but under 30 000 copies/ml) YES NO

I know that my child can be withdrawn from the study at any time without it affecting his/her care. YES NO

I agree to anonymised blood samples being taken and processed during the study and to be stored for studies which will help understand the disease. YES NO

I understand that clinical information may be reviewed by properly authorised individuals as part of the trial but that such information will be treated as strictly confidential. YES NO

I agree to my child's routine blood results and clinical information being included anonymously in continued follow up after the trial has ended. YES NO

Name of Parent/Guardian……………………………………….

Signature of Parent/Guardian.………………………………….. Date………………..

Signature of child (if appropriate) ……………………………… Date………………..

Name of Paediatrician ………………………………………….

Signature of Paediatrician ……………………………………… Date………………..

I would/would not like my GP to be notified about my participation in this trial.

Signature of Parent ……………………………………..…. Date………………..

Name of GP ………………………………………………….

Contact address of GP…………………………………………..

APPENDIX XVII

SAMPLE INFORMED CONSENT FORM FOR PACTG SITES

PACTG SAMPLE INFORMED CONSENT TEMPLATE

DIVISION OF AIDS
PEDIATRIC AIDS CLINICAL TRIALS GROUP (PACTG)
SAMPLE INFORMED CONSENT

PACTG 390 (USA PORTION OF PENPACT 1)

PENPACT 1 (PENTA 9/PACTG 390) "A PHASE II/III RANDOMIZED, OPEN-LABEL STUDY OF COMBINATION ANTIRETROVIRAL REGIMENS AND TREATMENT-SWITCHING STRATEGIES IN **HIV-1-INFECTED** ANTIRETROVIRAL NAIVE CHILDREN **>**30 DAYS AND **<** 18 YEARS OF AGE"

SHORT TITLE FOR THE STUDY: Different combination regimens and treatment switching guidelines in HIV-Infected children **<** 18 years of age. VERSION **3.0, dated 06/28/05**

INTRODUCTION

You are/your child/baby is being asked to take part in this research study because you/your child/baby are/is infected with HIV. This study is sponsored by the National Institutes of Health (NIH). The doctor in charge of this study at this site is _____. Before you decide if you want to be/want your child/baby a part of this study, we want you to know about the study.

This is a consent form. It gives you information about this study. The study staff will talk with you about this information. You are free to ask questions about this study at any time. If you agree to allow your child/baby to take part in this study, you will be asked to sign this consent form. You will get a copy to keep.

WHY IS THIS STUDY BEING DONE?

This study will evaluate two different treatment combinations of anti-HIV drugs, and will use one of two plans for switching from one to the other treatment combinations. One plan will switch treatment combination when the viral load is 1,000 copies of HIV-1 per milliliter of blood in the bloodstream, and the other plan will switch treatment when the viral load is 30,000 copies of HIV-1 per milliliter of blood in the bloodstream. The study will evaluate if there is a difference in benefit for either of the two switching plans in a four-year treatment.

APPENDIX XVII

Currently there are three kinds of anti-HIV drugs. These kinds of drugs have different chemical ways to fight HIV. The three types are Non-nucleoside reverse transcriptase inhibitors (NNRTI), Nucleoside reverse transcriptase inhibitors (NRTI), and Protease Inhibitors (PI).

These names are given based on their ways to lower HIV levels in your bloodstream. The use of different combinations of these kinds of drugs has been proven to be successful in decreasing the amount of HIV in the bloodstream.

This study will include the following drug combinations:

2 NRTIs + 1 PI
2 NRTIs + 1 NNRTI

Although HIV resistance to these drugs and their combinations is expected when the amount of virus circulating in the blood is increasing (giving a higher viral load test), there is evidence the virus growing while on treatment with these drug combinations will not produce HIV-related disease as rapidly as when the virus grows in a person without drug treatment.

There will be a recommended switch from one treatment combination to the other when your/your child's initial treatment combination is not able to control the amount of HIV in the bloodstream. There will be two different viral load values for this switch, 1,000 copies/mL or 30,000 copies/mL. The study will evaluate if switching drugs when the amounts of HIV in the bloodstream are low (1,000 copies/mL) or when they are high (30,000 copies/mL), or starting with a PI or not, makes a difference in the power of the second treatment combination to fight HIV. The study will also evaluate how well you/your child tolerate(s) the drugs over at least a four-year treatment.

Although the study will recommend a second treatment combination switching from PI to non-PI, or vice-versa, your/your baby's/your child's clinician will choose the best available treatment combination, which could be different from the ones recommended by this protocol.

This study will also evaluate and compare the safety and how acceptable is each drug combination regimen used right after enrollment and after switching.

WHAT DO I HAVE TO DO IF I AM IN THIS STUDY?

You/your child/baby will be assigned at the entry visit to one of the drug combination regimens mentioned above. It is important that you/your child/baby take(s) all the drugs properly as prescribed by your doctor. A computer will choose at random whether you/your child/baby will receive 2NRTIs+1NNRTI or 2NRTIs+1PI. This is the best way to compare different drug

APPENDIX XVII

combinations. The computer will also assign the viral load value at which you/your child/baby should switch drugs.

The current anti-HIV drugs available for your/your child's/baby's doctor to be prescribed as part of this study, are as follows: (The choice will depend on what your doctor feels is most suitable for you/your child/baby, and if new drugs/treatment combinations become available. These new options will be discussed with you/your child when it is time for switching)

<u>NRTIs:</u>

1. Abacavir **sulfate**: ABC, Ziagen®
2. Didanosine: ddI, Videx®, **Videx®EC**
3. **Emtricitabine: FTC, Emtriva®**
4. **Emtricitabine/Tenofovir disoproxil fumarate: FTC/TDF, Truvada®**
5. Lamivudine: 3TC, Epivir®
6. Lamivudine/Zidovudine: **3TC/ZDV,** Combivir®
7. Stavudine: d4T, Zerit®
8. **Tenofovir disoproxil fumarate: TDF, Viread®**
9. Zalcitabine: ddC, Hivid®
10. Zidovudine: AZT, **ZDV**, Retrovir®

<u>NNRTIs:</u>

1. Efavirenz: **EFV**, Sustiva®
2. Nevirapine: **NVP,** Viramune®

<u>PIs:</u>

1. Amprenavir: **APV**, Agenerase®
2. Indinavir **sulfate: IDV,** Crixivan®
3. Lopinavir/Ritonavir: **LPV/r,** Kaletra®
4. Nelfinavir **mesylate: NFV,** Viracept®
5. Saquinavir: **SQV**, Fortovase®; Invirase®
6. Ritonavir: **RTV**; Norvir®

<u>Screening Visit</u>

You/your child/baby will have tests to find out if you/your child/baby can enter the study. A complete physical exam will be done at this visit. About 2 teaspoons of blood will be drawn. If you/your child are/is old enough to become pregnant, a urine sample or a blood sample will be collected for a pregnancy test at this visit. The amount of blood for this pregnancy test will be much less than 1 teaspoon.

APPENDIX XVII

The blood collected on this study visit will include routine laboratory tests as with any physical exam and the following specific laboratory tests: viral load (amount of HIV in bloodstream), and CD4+ cell counts (the number of cells in bloodstream that fight HIV).

<u>Entry Visit</u>

You/your child/baby will need to come for an entry visit, which will be on the day you/your child/baby are assigned to one of the treatment regimens. In this visit, about 2 teaspoons of blood will be drawn for similar laboratory tests as described previously.
A urine sample will be collected on this visit. A pregnancy test will also be needed at this visit if you/your child are/is old enough to become pregnant.

On this visit, a neurologic exam will be performed. This exam evaluates the brain and nervous system functions, such as reflexes, hearing, sense of touch, vision, ability to move, and others.

<u>U.S. Domestic Sites</u>

Also on this visit a specialized doctor called neuropsychologist will perform an examination of your/your child's/baby's behavioral and learning development (this exam will include answering questions and doing some tasks).

<u>On Study Visits</u>

You/your child/baby will have a study visit at weeks 2, 4, 8, 12, 16, 24, and every 12 weeks thereafter until the week that your/your child's doctor determines that your/your child's drug regimen should be changed. Then you/your child/baby will come for a new re-entry visit and re-start the schedule of visits for the new treatment combination.

On these visits blood (about 2 teaspoons) and urine samples will be collected for similar laboratory tests as previously described.

To help understand the effect that not taking the study drugs as per doctor's orders has on you/your child/your baby HIV infection, you/your child will be asked several questions at some of these on study visits. There will be two sets of questions:
(1) to determine the amount the of study drugs actually taken within the previous 3 days, and
(2) to determine your/your child's/your baby's special reasons for not taking each study drug.

<u>U.S. Domestic Sites</u>

At weeks 24, 48, and every 48 weeks thereafter, the neuropsychologist will perform an examination of your/your child's/baby's behavioral and learning development (this exam will include answering questions and doing some tasks). These visits may take 1-3 hours.

APPENDIX XVII

HOW MANY PEOPLE WILL TAKE PART IN THIS STUDY?

About 128 children followed by the PACTG and 128 children followed by PENTA (a group like PACTG in Europe) will take part in this study.

HOW LONG WILL I BE IN THIS STUDY?

You/you child/baby will be in this study between 4 years [minimum amount of time] and about 8 years [maximum amount of time] depending on when you join.

WHY WOULD THE DOCTOR TAKE ME OFF THIS STUDY EARLY?

The study doctor may need to take you/your child/baby off the study early without your permission if:

- the study is cancelled by the U.S. Food and Drug Administration (FDA), National Institutes of Health (NIH), the drug companies supporting this study, or the site's Institutional Review Board (IRB). (An IRB is a committee that watches over the safety and rights of research subjects.)
- a Data Safety Monitoring Board (DSMB) recommends that the study be stopped early (A DSMB is an outside group of experts who monitor the study.)
- you are/your child/baby is not able to attend the study visits as required by the study

The study doctor may also need to take you/your child/your baby off the study drug(s) without your permission if:

- continuing the study drug(s) may be harmful to you/your child/baby
- you/your child/baby need(s) a treatment that you/your child/baby may not take while on the study
- you are/your child/baby is not able to take the study drug(s) as required by the study
- **you/your child become(s) pregnant**

If you/your child/baby must stop taking the study drug(s) before the study is over, the study doctor may ask you/your child/baby to continue to be part of the study and return for some study visits and procedures.

WHAT ARE THE RISKS OF THE STUDY?

Treatment combinations of anti-HIV drugs and other drugs may cause increase risk of side effects or death.
There is the risk of serious and/or life threatening side effects when non-study medications are taken with study drugs. For your safety, you must tell your/you child's/your baby's clinician

APPENDIX XVII

and/or the study doctor or nurse about all medications you are taking before you start the study and also before starting any new medications while on the study. In addition, you must tell the study doctor or nurse before enrolling in any other clinical trials while on this study.

Once your/your child's/baby's clinician has decided which antiretroviral drugs will prescribe as your regimen, he/she will inform you of risk and side effects observed for those medications.

Other side effects besides those listed and side effects from taking these drugs together may occur. If any unusual symptoms or changes happen, you should call your/your child's/baby's doctor immediately. It is also important that while participating in the study, you/your child/baby do(es) not take/receive any other prescription drugs or over-the-counter medications without first talking to your/your child's/baby's doctor or study nurse.

<u>For the NRTI group of drugs</u>

Lactic acidosis **(elevated lactic acid levels in the blood)** and severe hepatomegaly (enlarged liver) with steatosis (fatty liver) that may result in liver failure, other complications **or** death have been reported with the use of antiretroviral nucleoside analogues alone or in combination. The liver complications and death have been seen more often in women on these drug regimens. Some nonspecific symptoms that might indicate lactic acidosis include: unexplained weight loss, stomach discomfort, nausea, vomiting, fatigue, **cramps, muscle pain,** weakness, **dizziness,** and shortness of breath.

<u>For the NNRTI group of drugs</u>

Severe liver damage that can result in death may occur and is often associated with a rash. Being female or having a higher CD4 cell count, regardless of gender, increase the risk of developing liver damage. If you are developing liver **damage,** you may **have** one or more of the following: tiredness, general feeling of illness **or flu-like feeling**, loss of appetite, nausea, pale stools, dark urine, yellowing of the skin or **whites of the** eyes, liver tenderness, or abnormal liver function tests. Subjects with active Hepatitis B or C infection or abnormal liver function tests are at higher risk for worsening liver disease.

Rash is the most common side effect. **Rash occurs more often in women. Most rashes occur early during treatment.** The rash may be severe and rarely **may cause** death. One of the risk factors for developing serious skin reactions includes failure to take medications properly during the first 14 days **of treatment**.

Hypersensitivity reactions **(allergic reaction)** may occur. **These reactions are rarely fatal. The symptoms that you may notice are** rash, fever, **tiredness**, muscle or joint aches, **flu-like feeling,** blisters, mouth **sores**, facial swelling, red eyes and irritation of the eyes, general feeling of discomfort, **and/or liver damage described above,** kidney problems, and/or changes in white blood cell levels.

APPENDIX XVII

If you develop **any of the side effects** listed above, no matter how long you have been receiving nevirapine, you must contact **your health care provider right away** and **before you take your next dose. Your health care provider will instruct you on what to do next.** If you and your doctor then decide to stop your treatment because of **liver damage**, hypersensitivity, or severe skin reactions, you should never take nevirapine again.

The use of efavirenz during pregnancy and especially early pregnancy should be avoided. Efavirenz may cause fetal harm when taken during the first three months of pregnancy. Serious birth defects, including those of the central nervous system, have been seen in the offspring of animals and women on efavirenz.

A false-positive urine screening test for marijuana has been seen with one particular test brand and has not been seen when using other screening tests or with tests used to confirm results for marijuana.

For the PI group of drugs

The use of potent antiretroviral drug combinations (which commonly include a protease inhibitor) may be associated with an abnormal placement of body fat and wasting. Some of the body changes include:
- Increase in fat around the waist and stomach area
- Increase in fat on the back of the neck
- Thinning of the face, legs, and arms
- Breast enlargement

The use of protease inhibitors may also be associated with **the following:**
- **Increases in the amount of triglycerides and/or cholesterol in the blood**
- **Development of** diabetes or the worsening of **high** blood sugar

There have been reports of increased bleeding in HIV-infected persons with hemophilia (a bleeding disorder) who were treated with protease inhibitors. It is not known if protease inhibitors were the cause of these bleeding episodes

Blood Drawing Risks

Blood drawing may cause some discomfort, bleeding or bruising where the needle enters the body. A small blood clot may form at the site of venipuncture or there may be swelling in the area. There is a small risk of a minor infection at the blood draw site.

APPENDIX XVII

Risks of Switching Drug Regimens **and Resistance**

Every time you/your child/your baby switch(es) drug regimens because of **increased** HIV levels in the bloodstream, there is a possibility that new drug regimens may be difficult to find. **Furthermore, the HIV in your/your child's/your baby's blood might change – or become resistant to – certain drugs. This means that certain drugs do not work well against your/your child's/your baby's HIV. Even n**ew drugs from the same **group** of drugs as the current regimen may not **work well against your/your child's/your baby's HIV**. Your/your child's/your baby's clinician will explain the importance of taking all study drugs exactly the way and times they are prescribed to **decrease** the chance of **developing drug resistance, and to** keep HIV levels in the bloodstream **low**.

Neuropsychological Testing Risks (U.S. Domestic Sites)

The discomforts of neuropsychological testing are tiredness and difficulty concentrating.

ARE THERE RISKS RELATED TO PREGNANCY?

The drug or drug combinations in this study may be unsafe for unborn babies. The risks to unborn babies for each drug will be explained to you by your/your child's clinician once he/she has determined the antiretroviral drugs of your study regimen. If you are having sex that could lead to pregnancy, you must agree not to become pregnant or make a female pregnant

Because of the risk involved, you and your partner must use two methods of birth control that you discuss with the study staff. You must continue to use both methods until three months after stopping study drugs. You may choose two of the birth control methods listed below:

- Birth control drugs that prevent pregnancy given by pills, shots or placed under the skin
- Male or female condoms with or without a cream or gel that kills sperm
- Diaphragm or cervical cap with a cream or gel that kills sperm
- Intrauterine device (IUD)

If you are assigned to receive study drugs that do not require the use of two birth control methods, the study staff will discuss your options.

If you can become pregnant, you must have a pregnancy test before you enter this study. The test must show that you are not pregnant. If you think you may be pregnant at any time during the study, tell your study staff right away. The study staff will talk to you about your choices.

APPENDIX XVII

ARE THERE BENEFITS TO TAKING PART IN THIS STUDY?

If you/your child/baby take(s) part in this study, there may be a direct benefit to you/your child/baby, but no guarantee can be made. It is also possible that you/your child/baby may receive no benefit from being in this study. Information learned from this study may help others who have HIV.

WHAT OTHER CHOICES DO I/DOES MY CHILD/BABY HAVE BESIDES THIS STUDY?

Instead of being in this study you have the choice of:

- treatment with prescription drugs, including some of the drugs used in this study as well as other drugs not prescribed in the study, are available to you/your child/baby
- treatment with experimental drugs, if you/your child/baby qualify(ies)
- no treatment

Please talk to your doctor about these and other choices that may be available to you/your child/baby. Your doctor will explain the risks and benefits of these choices.

WHAT ABOUT CONFIDENTIALITY?

This study is part of a combined effort of two different organizations, the PACTG group and the PENTA group. The study teams from both organizations wrote one study called PENPACT 1, which includes actually two different studies, PACTG 390 (the one you/your child/your baby are/is enrolling) **at U.S. and international sites connected with the PACTG,** and PENTA 9 in Europe. PENPACT 1 will combine the information from these two studies, for a larger number of subjects.

To help us protect your privacy, we have obtained a Certificate of Confidentiality from the National Institutes of Health. With this Certificate, the researchers cannot be forced to disclose information that may identify you, even by a court subpoena, in any federal, state, or local civil, criminal, administrative, legislative, or other proceedings. The researchers will use the Certificate to resist any demands for information that would identify you, except as explained below. The Certificate cannot be used to resist a demand for information from personnel of the United States Government that is used for auditing or evaluation of federally funded projects or for information that must be disclosed in order to meet the requirements of the federal Food and Drug Administration (FDA).

People who may review your records include: the U.S. Food and Drug Administration (FDA), (insert name of site) IRB, National Institutes of Health (NIH), PACTG (Pediatrics AIDS Clinical

APPENDIX XVII

Trials Group) study staff, PENTA (Pediatric European Network for Treatment of AIDS) staff and study monitors.

You should understand that a Certificate of Confidentiality does not prevent you or a member of your family from voluntarily releasing information about you or your participation in this research. If an insurer, employer, or other person obtains your written consent to receive research information, then the researchers may not use the Certificate of Confidentiality to withhold that information.

[The researchers should include language such as the following if they intend to make voluntary disclosure about things such as child abuse]

The Certificate of Confidentiality does not prevent the researchers from disclosing voluntarily, without your consent, information that would identify you as a participant in the research project under the following circumstances. [The researchers should state here the conditions under which voluntary disclosure will be made]

Your/your child's/baby's records may be reviewed by the U.S. Food and Drug Administration (FDA), <u>(insert name of site)</u> IRB, National Institutes of Health (NIH), PACTG (Pediatrics AIDS Clinical Trials Group) study staff, PENTA (Pediatric European Network for Treatment of AIDS) staff and study monitors.

WHAT ABOUT STORED SAMPLES?

<u>Information for NIAID Sites:</u>

Some of your/your child's blood will be taken and stored (with usual protectors of identity) and used for testing that is required for this study (pharmacokinetic and genotypic) and **future PACTG-approved, HIV-related research**. Less than 1 teaspoon of your/your child's blood will be taken for this purpose.

Your/your child's samples will not be sold or directly used to produce commercial products. All proposed research studies using your/your child's samples will be reviewed by the National Institutes of Health (NIH). There is no time limit on how long your/your child's samples will be stored. The researchers do not plan to contact you or your/your child's regular doctor with the results of future studies done using your/your child's stored samples. This is because research studies are often done with experimental procedures, and results of such studies should not be used to make decisions about your/your child's medical care. If the researchers decide that the result of a certain study provides important information for your/your child's medical care, then your/your child's study doctor will be notified. If you would like to be contacted with this sort of information, you must notify the study staff of any changes in your/your child's address or phone number.

APPENDIX XVII

You may decide that you do not want your/your child's samples stored for future research studies. You/your child can still participate in this study even if you make this decision. You may withdraw your consent for the storage and use of your/your child's samples at any time. If you withdraw your consent, these stored samples will be destroyed.

Please read the following statement carefully and then mark your initials in the appropriate space provided.

I agree to allow my/my child's blood samples to be stored for use in future PACTG-approved, HIV-related research studies.

__________ Yes __________ No __________ Initials __________ Date

<u>Information for NICHD Sites:</u>

Some of your blood specimens collected as part of this study will be stored for testing at a later date as part of this study. There is a separate consent form to explain this and get your/your child's consent.

WHAT ARE THE COSTS TO ME?

Taking part in this study may lead to added costs to you and your insurance company. In some cases it is possible that your insurance company will not pay for these costs because you/your child/baby is/are taking part in a research study.

All the study medications prescribed by your/your child's doctor will not be covered by the study. You/your child and/or your/your child's health insurance will be responsible for purchasing the study medications.

WHAT HAPPENS IF I AM INJURED?

If you/your child/baby is/are injured as a result of being in this study, you/your child/baby will be given immediate treatment for your injuries. The cost for this treatment will be charged to you or your insurance company. There is no program for compensation either through this institution or the National Institutes of Health (NIH). You will not be giving up any of your legal rights by signing this consent form.

WHAT ARE MY RIGHTS AS A RESEARCH SUBJECT?

Taking part in this study is completely voluntary. You may choose not to take part/not to allow your child/baby to take part in this study or leave this study/take your child/baby out of the study at any time. You/your child/baby will be treated the same no matter what you decide.

APPENDIX XVII

We will tell you about new information from this or other studies that may affect your/your child's/baby's health, welfare or willingness to stay in this study. If you want the results of the study, let the study staff know.

WHAT DO I DO IF I HAVE QUESTIONS OR PROBLEMS?

For questions about this study or a research-related injury, contact:

- name of the investigator or other study staff
- telephone number of above

For questions about your/your child's/baby's rights as a research subject, contact:

- name or title of person on the Institutional Review Board (IRB) or other organization appropriate for the site
- telephone number of above

APPENDIX XVII

SIGNATURE PAGE

If you have read this consent form (or had it explained to you), all your questions have been answered and you agree to take part in this study, please sign your name below.

______________________	______________________________
Participant's Name (print)	Participant's Signature and Date
______________________	______________________________
Participant's Legal Guardian (print)<br>(As appropriate)	Legal Guardian's Signature and Date
______________________	______________________________
Study Staff Conducting<br>Consent Discussion (print)	Study Staff Signature and Date
______________________	______________________________
Witness' Name (print)<br>(As appropriate)	Witness's Signature and Date
______________________	______________________________
Father's Name<br>(As appropriate)	Father's Signature and Date

APPENDIX XVIII

FACT SHEET and TEMPLATE CONSENT FORM for
Specimen Storage at Repositories funded by the
National Institute of Child Health and Human Development (NICHD)
PARENT FACT SHEET

When your child joins this NICHD sponsored Study, you will be asked to give permission for having some specimens that the doctor or nurse will take from your child's body saved in a repository. (A repository is a special laboratory with freezers where specimens like blood or tissue cells and body fluids that are taken from you during a study are kept. Your child's name will not be on these specimens, only a special study number. The people who run the repository laboratory will not know your child's name.)

Why have a repository?

Researchers can learn a lot from a study but as time goes by the tests that they used get better or new tests appear, and there is a need to learn more. When study volunteers consent to put specimens in the repository and consent to the researchers doing new tests on the specimens later after their time in the study is ended, these questions can be answered and more can be learned. None of these future studies would happen unless the Institutional Review Board overseeing the repository examines the study and makes sure that your child's rights are being protected.

How will my child's privacy be protected?

The only record that your child participated in this NICHD sponsored study is at the clinic where it is kept separate from your child's health records and locked away.

Your child's specimens in the repository will not have your child's name on them. The specimens will have a special study code. It will be the same code that is on your child's information in the NICHD sponsored Study from your child's interviews and examinations. Again, none of this information will have your child's name on it.

How would a researcher get to use the specimens in the repository?

If a researcher wants to do a test on specimens from the NICHD sponsored repository in the future, he or she will write up the idea and it will have to be approved by a committee to make sure the research is worthwhile. If the idea is approved, then coded specimens and coded information will be given to the researcher. The researcher will not know the names, addresses, or phone numbers of the people who gave the specimens to the repository.

APPENDIX XVIII

Why wouldn't I find out the results of the research using my child's specimens?

You will <u>not</u> receive the results of research done with your child's specimens. This is because research can take a long time and must use specimens from many people before results are known. Results from research using your child's specimens may not be ready for many years. Often when studies are first done, it is not always clear how to use the information from the study to change the health care that people receive. So none of these study results is likely to affect your child's care right now, but they may be helpful to people like your child in the future. Your child's specimens can last in the freezer for many years and there is no time limit to when studies could be done in the future.

Would I ever be contacted in the future about research using my child's specimens?

All of the studies to be done in the future on your child's specimens in the repository will be for the particular reasons that you agreed to. Every study that is planned to use specimens from your child and others from this NICHD sponsored Study has to be reviewed by a special committee of people known as an Institutional Review Board, who are not part of the Study. Their goal is to make sure that what is planned is the same kind of study that you had agreed to. If it is, then the research will go ahead since you would have agreed that these particular tests could be done without anyone contacting you to get your permission in the future.

If the study to be done is not like the kind of tests you agreed could be done, then the committee will decide if you need to be contacted to give permission for the new study.

I gave my permission to testing my child's specimens in the repository, but what if I change my mind?

People always have the right to stop participating in research. So if you decide that you do not want researchers to be able to use the specimens from your child in the repository, you can contact the clinic staff. They will tell the repository that the specimens with the study code number linked to your child's name in the clinic should not be studied. These specimens can be removed from the repository and destroyed if you tell us to do that.

What type of research will be done with my child's specimens?

Many different kinds of studies use specimens. Some researchers may develop new tests to find diseases. Others may develop new ways to treat or even cure diseases. In the future, some of the research may help to develop new products, such as tests and drugs. If this would happen and these tests or drugs make money, there are no plans to share that money with the people who gave the specimens.

APPENDIX XVIII

As part of this study (insert title), your child is being asked to have some (insert specimen source- blood, urine, tissue, genital fluid, saliva, etc.) taken. These specimens will go into the NICHD repository for research to be done at some time in the future so that more information can come from your child's time in this NICHD sponsored Study.

You do not have to agree to store your child's specimens for future tests for your child to take part in this study. Your child will not lose any benefits to which your child is entitled if you decide against storing your child's specimens.

You will also be asked to agree that these particular tests can be done without anyone contacting you to get your permission sometime in the future. No one doing these tests would know that these specimens came from your child and no one would contact you or your doctor or nurse with the results from these tests that might happen in the future.

APPENDIX XVIII

TEMPLATE CONSENT FORM

What are the <u>general</u> HIV-related studies that can be done with the repository specimens?

Researchers would like to store your child's specimens to understand how HIV causes disease and complications, and how best to treat or prevent HIV infection and its complications. They need specimens from people who have HIV and from those who do not. Sometimes, too, the specimens can be used to learn something about new problems that people with HIV have like liver disease, diabetes, and heart disease. These general studies would not include any genetic testing (looking at your child's DNA).

> Benefits: There are no direct benefits to your child. Your child will be helping researchers learn more about how to help people with HIV or at risk of HIV infection.
>
> Risks: The specimens would be collected as part of your child's study visits. (<u>Insert text about collection procedures</u>.) Once in the repository, there are few risks. Your child's name will not be available to the repository or to the scientists who may be doing any future test.

I give permission for the use of my child's stored specimens for the purposes stated in the preceding section (general HIV-related tests).

____________________________ ____________________________ ________

Parent or Legal Guardian Signature   Witness Signature   Date

I give my assent to the use of my stored specimens for the purposes stated in the preceding section (general HIV-related tests).

____________________________ ____________________________ ________

Participant Signature   Witness Signature   Date

APPENDIX XVIII

What are the special HIV-related studies that can be done with the repository specimens?

Researchers in this study would also like to store your child's specimens to understand how HIV causes disease and complications, and how best to treat or prevent HIV infection and its complications through looking at how each person's genetic makeup (your child's DNA) either protects them or puts them at greater risk. It may be that researchers use some of your child's blood to make a "cell line". That means the blood cells can keep dividing and give an endless supply of your child's DNA for tests to be done in the future. This kind of information will be particularly important as scientists work toward a vaccine that could protect people from AIDS. They need specimens from people who have HIV and from those who do not.

Benefits: There are no direct benefits to your child. Your child will be helping researchers learn more about how to help people with HIV or at risk of HIV infection.

Risks: The specimens would be collected as part of your child's study visits. (Insert text about collection procedures.) Once in the repository, there are few risks. Your child's name will not be available to the repository or to the scientists who may be doing any future test. Since there are no plans to give participants the results of the tests performed on their stored specimens, you will not receive any information on your child's genetic makeup.

I give permission for the use of my child's stored specimens for the purposes stated in the preceding section (special HIV-related tests).

____________________________ ____________________________ _________
Parent or Legal Guardian Signature Witness Signature Date

I give my assent to the use of my stored specimens for the purposes stated in the preceding section (special HIV-related tests).

____________________________ ____________________________ _________
Participant Signature Witness Signature Date

APPENDIX XVIII

What if I have more questions?

If you have any questions about the repository, about storage, or the use of your child's samples, contact (<u>Study personnel</u>) at (<u>phone</u>).

If you have questions about giving consent or your child's rights as a research volunteer, contact the (<u>Name of Institution</u>) Institutional Review Board at (<u>phone</u>).

I refuse to have any specimen collected from my child stored in the repository.

______________________________	______________________________	__________
Parent or Legal Guardian Signature	Witness Signature	Date

APPENDIX XVIII

FACT SHEET and TEMPLATE CONSENT FORM for
Specimen Storage at the Repository of the National Institute of Child Health and Human Development (NICHD)
YOUTH FACT SHEET

When you join this NICHD sponsored Study, you will be asked to consent to having some specimens that the doctor or nurse will take from your body saved in a repository. (A repository is a special laboratory with freezers where specimens like blood or tissue cells and body fluids that are taken from you during the study are kept. Your name will not be on these specimens, only a special study number. The people who run the repository laboratory will not know your name.)

Why have a repository?

Researchers can learn a lot from a study but as time goes by the tests that they used get better or new tests appear, and there is a need to learn more. When study volunteers consent to put specimens in the repository and consent to the researchers doing new tests on the specimens later after their time in the study is ended, these questions can be answered and more can be learned. None of these future studies would happen unless the Institutional Review Board overseeing the repository examines the study and makes sure that your rights are being protected.

How will my privacy be protected?

The only record that <u>you</u> participated in this NICHD sponsored Study is at your clinic where it is kept separate from your health records and locked away.

Your specimens in the repository will not have your name on them, only a special study code. It will be the same code that is on your information in the NICHD sponsored Study from your interviews and examinations. Again, none of this information will have your name on it.

How would a researcher get to use the specimens in the repository?

If a researcher wants to do a test on specimens from the NICHD repository in the future, he or she will write up the idea and it will have to be approved by a committee to make sure the research is worthwhile. If the idea is approved, then coded specimens and coded information will be given to the researcher. The researcher will not know the names, addresses, or phone numbers of the people who gave the specimens to the repository.

APPENDIX XVIII

Why wouldn't I find out the results of the research using my specimens?

You will <u>not</u> receive the results of research done with your specimens. This is because research can take a long time and must use specimens from many people before results are known. Results from research using your specimens may not be ready for many years. Often when studies are first done, it is not always clear how to use the information from the study to change the health care that people receive. So none of these study results is likely to affect your care right now, but they may be helpful to people like you in the future. Your specimens can last in the freezer for many years and there is no time limit to when studies could be done in the future.

Would I ever be contacted in the future about research using my specimens?

All of the studies to be done in the future on your specimens in the repository will be for the particular reasons that you agreed to. Every study that is planned to use specimens from you and others from this NICHD sponsored Study has to be reviewed by a special committee of people known as an Institutional Review board, who are not part of the Study. Their goal is to make sure that what is planned is the same kind of study that you agreed to. If it is, then the research will go ahead since you would have agreed that these particular tests could be done without anyone contacting you to get your permission in the future.

If the new study to be done is not like the kind of tests you agreed could be done, then the committee will decide if you need to be contacted to give consent for the new study.

I gave my consent to testing my specimens in the repository, but what if I change my mind?

People always have the right to stop participating in research. So if you decide that you do not want researchers to be able to use the specimens from you in the repository, you can contact the clinic staff. They will tell the repository that the specimens with the study code number linked to your name in the clinic should not be studied. These specimens can be removed from the repository and destroyed if you tell us to do that.

What type of research will be done with my specimens?

Many different kinds of studies use specimens. Some researchers may develop new tests to find diseases. Others may develop new ways to treat or even cure diseases. In the future, some of the research may help to develop new products, such as tests or drugs. If this would happen and these tests or drugs make money, there are no plans to share that money with the people who gave the specimens.

APPENDIX XVIII

As part of this study (insert title), you are being asked to have some (insert specimen source-blood, urine, tissue, genital fluid, saliva, etc.) taken from you. These specimens will go into the NICHD repository for research to be done at some time in the future so that more information can come from your time in this NICHD sponsored Study.

You do not have to agree to store your specimens for future tests to take part in this study. You will not lose any benefits to which you are entitled if you decide against storing your specimens.

APPENDIX XVIII

TEMPLATE CONSENT/ASSENT FORM

What are the general HIV-related studies that can be done with the repository specimens?

Researchers would like to store your specimens to understand how HIV causes disease and complications, and how best to treat or prevent HIV infection and its complications. They need specimens from people who have HIV and from those who do not. Sometimes, too, the specimens can be used to learn something about new problems that people with HIV have like liver disease, diabetes, and heart disease. These general studies would not include any genetic testing (looking at your DNA).

Benefits: There are no direct benefits to you. You will be helping researchers learn more about how to help people with HIV or at risk of HIV infection.

Risks: The specimens would be collected as part of your study visits. (Insert text about collection procedures.) Once in the repository, there are few risks. Your name will not be available to the repository or to the scientists who may be doing any future test.

I consent to the use of my stored specimens for the purposes stated in the preceding section (general HIV-related tests).

______________________________	______________________________	__________
Participant Signature	Witness Signature	Date

What are the special HIV-related studies that can be done with the repository specimens?

Researchers in this study would also like to store your specimens to understand how HIV causes disease and complications, and how best to treat or prevent HIV infection and its complications through looking at how each person's genetic makeup (your DNA) either protects them or puts them at greater risk. It may be that researchers use some of your blood to make a "cell line". That means the blood cells can keep dividing and give an endless supply of your DNA for tests to be done in the future. This kind of information will be particularly important as scientists work toward a vaccine that could protect people from AIDS. They need specimens from people who have HIV and from those who do not.

APPENDIX XVIII

Benefits: There are no direct benefits to you. You will be helping researchers learn more about how to help people with HIV or at risk of HIV infection.

Risks: The specimens would be collected as part of your study visits. (Insert text about collection procedures.) Once in the repository, there are few risks. Your name will not be available to the repository or to the scientists who may be doing any future test. Since there are no plans to give participants the results of the tests performed on their stored specimens, you will not receive any information on your genetic makeup.

I consent to the use of my stored specimens for the purposes stated in the preceding section (special HIV-related tests).<br><br>____________________ ____________________ ________<br>Participant Signature Witness Signature Date

What if I have more questions?

If you have any questions about the repository, about storage, or the use of your samples, contact (Study personnel) at (phone).
If you have questions about giving consent or your rights as a research volunteer, contact the (Name of Institution) Institutional Review Board at (phone).

I refuse to have any specimen collected for storage in the repository.<br><br>____________________ ____________________ ________<br>Participant Signature Witness Signature Date
